# Beyond functional disability: longitudinal joint trajectories of muscle strength and walking speed and their association with new-onset diabetes risk among older adults without ADL impairment

**DOI:** 10.3389/fendo.2026.1935223

**Published:** 2026-10-28

**Authors:** Xiangyang Chen, Xuan Wu, Sizhe Feng

**Affiliations:** The First Affiliated Hospital of Wenzhou Medical University, Wenzhou, China

**Keywords:** ADL, aging cohorts, group-based trajectory modeling, longitudinal trajectories, muscle strength, new-onset diabetes, walking speed

## Abstract

**Background:**

With population aging, diabetes poses a growing threat to older adults. However, whether longitudinal trajectories of muscle strength and walking speed can identify diabetes risk before ADL impairment develops remains unclear.

**Methods:**

This longitudinal cohort study used data from the HRS, ELSA, SHARE, and CHARLS. Group-based trajectory modeling (GBTM) was applied to identify joint longitudinal trajectories of walking speed and muscle strength. Associations between trajectory groups and new-onset diabetes were assessed using inverse probability of treatment weighting (IPTW)-adjusted Cox proportional hazards models. In addition, 129 candidate machine learning models, comprising nine individual algorithms and 120 Stacking combinations, were evaluated for new-onset diabetes prediction, and SHAP (SHapley Additive exPlanations) analysis was used to interpret model predictions. The robustness of the findings was further evaluated using competing risk models, discrete-time logistic regression models, stratified analyses, and cross-cohort machine learning validation.

**Results:**

During median follow-up periods of 10, 12, 6, and 8 years, 341, 265, 170, and 448 new-onset diabetes events occurred in the HRS, ELSA, SHARE, and CHARLS cohorts, respectively. GBTM identified distinct joint trajectories of muscle strength and walking speed across cohorts. Compared with participants maintaining higher functional levels, those with unfavorable trajectories had significantly increased risks of incident diabetes in fully adjusted IPTW-Cox models (HRS: adjusted HR = 4.17, 95% CI: 3.10–5.61; ELSA: adjusted HR = 3.85, 95% CI: 2.55–5.79; SHARE: adjusted HR = 3.38, 95% CI: 2.25–5.06; CHARLS: adjusted HR = 2.60, 95% CI: 1.87–3.61). Sensitivity analyses consistently supported these associations. Machine learning models further evaluated the contribution of joint trajectories within diabetes prediction frameworks, with SHAP analyses indicating that these trajectory features contributed to model predictions.

**Conclusions:**

Unfavorable joint trajectories of muscle strength and walking speed were associated with higher incident diabetes risk among older adults without baseline ADL impairment, highlighting the potential value of longitudinal functional monitoring for early diabetes risk stratification.

## 1 Introduction

With increasing global life expectancy, diabetes has become a major public health challenge, substantially affecting health outcomes, quality of life, and healthcare expenditures among older adults (1). The growing burden of diabetes in aging populations is driven not only by traditional metabolic risk factors but also by geriatric syndromes, including frailty, progressive functional decline, multimorbidity, and increased mortality risk (2, 3). Therefore, identifying early indicators of diabetes susceptibility among older adults is essential for implementing precision prevention strategies and promoting healthy aging (4).

Current clinical approaches for diabetes risk assessment primarily rely on conventional metabolic factors, including chronological age, body mass index (BMI), family history, blood glucose levels, and lifestyle characteristics (5). However, these indicators mainly reflect static metabolic conditions and may not capture subtle risk signals embedded in dynamic physiological changes during aging. This limitation is particularly relevant among older adults without apparent impairment in activities of daily living (ADL), among whom early identification of individuals at elevated risk remains challenging. Consequently, increasing attention has been directed toward physical function-related measures, suggesting that skeletal muscle function may represent an important link between biological aging and metabolic disease development, providing a foundation for investigating the combined effects of muscle strength and walking speed (6).

Declining muscle strength and reduced walking speed are among the most prevalent and clinically meaningful manifestations of age-related functional deterioration and are widely used as core indicators for assessing sarcopenia, frailty, and overall physical health (6). Biologically, skeletal muscle is not merely a mechanical tissue responsible for movement but also a metabolically active endocrine organ. It plays a critical role in maintaining glucose homeostasis through regulation of glucose uptake, insulin sensitivity, inflammatory pathways, and energy metabolism (7, 8). Accordingly, progressive deterioration of muscle function may contribute directly to diabetes development through multiple pathways involving insulin resistance, chronic low-grade inflammation, and metabolic dysfunction (4).

Accumulating epidemiological evidence has demonstrated that lower baseline grip strength and slower walking speed are associated with increased diabetes risk (9, 10). However, most previous studies have relied on single-point measurements, thereby overlooking the dynamic nature of muscle function during aging (11). Furthermore, muscle strength and walking speed represent distinct but complementary dimensions of physical function. Muscle strength primarily reflects neuromuscular capacity and physiological reserve, whereas walking speed integrates lower-extremity function, balance, cardiorespiratory fitness, and complex neuromotor coordination (12). Therefore, evaluating these indicators separately may fail to capture the multidimensional characteristics of functional decline. Compared with isolated measurements, simultaneous assessment of their longitudinal trajectories may provide a more comprehensive representation of functional aging patterns (11). However, whether joint trajectories of muscle strength and walking speed can predict new-onset diabetes among older adults without baseline functional impairment remains unclear.

Previous investigations examining the relationship between longitudinal muscle function changes and diabetes risk have several methodological limitations. First, most studies have been conducted within single cohorts, restricting population diversity and limiting external validity and generalizability (13). Given substantial variations in lifestyle behaviors, healthcare systems, and social environments across populations, large-scale longitudinal studies involving diverse multinational cohorts are required for robust validation. Second, conventional approaches typically evaluate muscle strength or walking speed as independent exposures and fail to capture complex functional phenotypes arising from their interaction over time. Trajectory modeling provides an opportunity to identify subgroups with distinct longitudinal patterns and offers a novel framework for understanding heterogeneity in functional aging. Therefore, joint trajectory analysis may provide valuable insights into how combined changes in muscle strength and walking speed influence diabetes risk.

Third, previous studies investigating functional trajectories and disease outcomes have primarily focused on population-level associations, while the translation of dynamic functional indicators into individualized prediction tools remains limited (14). Recent advances in machine learning and explainable artificial intelligence provide new opportunities to integrate multidimensional health information, improve prediction accuracy, and identify clinically meaningful predictive features (15, 16). Therefore, integrating trajectory modeling, robust causal inference methods, and advanced machine learning approaches is essential to comprehensively characterize the relationship between dynamic muscle function changes and diabetes risk in representative aging populations.

To address these gaps, we analyzed four large longitudinal aging cohorts (HRS, ELSA, SHARE, and CHARLS) among adults without baseline ADL impairment or diabetes. Joint trajectories of muscle strength and walking speed were identified using trajectory modeling, followed by IPTW-adjusted Cox models with multiple sensitivity analyses to assess diabetes risk. Machine learning models with SHAP interpretation and cross-cohort validation were further applied to evaluate the predictive value and generalizability of these trajectories.

## 2 Methods

### 2.1 Study population

The Health and Retirement Study (HRS), conducted by the University of Michigan with support from the National Institute on Aging, is designed to investigate the dynamic relationships among health, economic status, and social factors in non-institutionalized adults aged ≥51 years in the United States.

The English Longitudinal Study of Ageing (ELSA), conducted by a consortium led by University College London and the National Centre for Social Research, aims to longitudinally examine health, economic conditions, and social circumstances among adults aged ≥50 years living in private households in England.

The Survey of Health, Ageing and Retirement in Europe (SHARE), coordinated by the SHARE-ERIC consortium and the Munich Center for the Economics of Aging at the Max Planck Institute for Social Law and Social Policy, is a cross-national longitudinal cohort study that collects harmonized data on health, socioeconomic status, and social and family networks among community-dwelling adults aged ≥50 years across multiple European countries.

The China Health and Retirement Longitudinal Study (CHARLS), led by the National School of Development at Peking University and the China Center for Economic Research, is a nationally representative longitudinal survey that collects harmonized information on health status, socioeconomic conditions, and family characteristics among community-dwelling adults aged ≥45 years across 28 provinces in China.

This longitudinal cohort study was conducted using data from four large-scale aging cohorts: HRS, ELSA, SHARE, and CHARLS. These cohorts are nationally representative, ongoing longitudinal studies involving community-dwelling middle-aged and older adults. Standardized interviews and physical examinations are generally conducted biennially to systematically collect detailed information on sociodemographic characteristics, health status, functional ability, and health-related behaviors.

Baseline waves were selected according to the availability of harmonized longitudinal assessments of walking speed and muscle strength and sufficient subsequent follow-up for incident diabetes ascertainment. Therefore, cohort-specific baseline waves differed due to variations in survey design, assessment schedules, and data availability.

In the HRS cohort, wave 8 was defined as the baseline assessment, and 18,469 participants were initially included. A total of 15,869 participants were excluded according to the following criteria: (1) presence of ADL impairment during the first three waves (n=3629); (2) diagnosis of diabetes during the first three waves (n=2463); (3) missing walking speed measurements during the first three waves (n=2480); (4) missing muscle strength measurements during the first three waves (n=7116); and (5) lack of follow-up information (n=181). Finally, 2600 participants from the HRS cohort were included in the present study (**Figure 1**).

**Figure 1:**
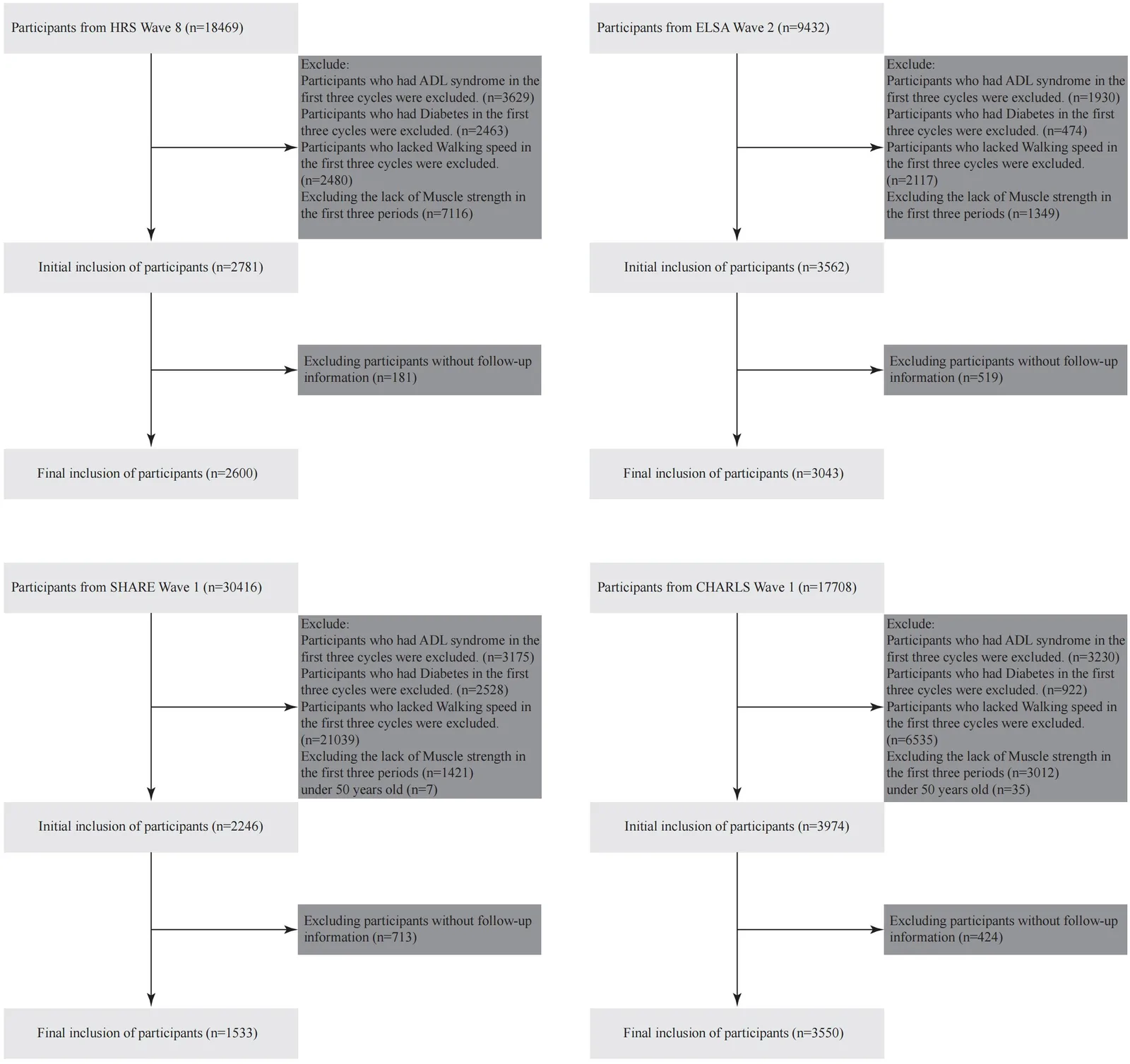
Flowchart of participant selection.

In the ELSA cohort, wave 2 was defined as the baseline assessment, and 9432 participants were initially included. A total of 6389 participants were excluded according to the following criteria: (1) presence of ADL impairment during the first three waves (n=1930); (2) diagnosis of diabetes during the first three waves (n=474); (3) missing walking speed measurements during the first three waves (n=2117); (4) missing muscle strength measurements during the first three waves (n=1349); and (5) lack of follow-up information (n=519). Finally, 3043 participants from the ELSA cohort were included in this study (**Figure 1**).

In the SHARE cohort, wave 1 was defined as the baseline assessment, with 30,416 participants initially included. A total of 28,883 participants were excluded according to the following criteria: (1) presence of ADL impairment during the first three waves (n=3175); (2) diagnosis of diabetes during the first three waves (n=2528); (3) missing walking speed measurements during the first three waves (n=21039); (4) missing muscle strength measurements during the first three waves (n=1421); (5) age <50 years (n=7); and (6) lack of follow-up information (n=713). Finally, 1533 participants from the SHARE cohort were included in this study (**Figure 1**).

In the CHARLS cohort, wave 1 was defined as the baseline assessment, with 17,708 participants initially included. A total of 14,158 participants were excluded according to the following criteria: (1) presence of ADL impairment during the first three waves (n=3230); (2) diagnosis of diabetes during the first three waves (n=922); (3) missing walking speed measurements during the first three waves (n=6535); (4) missing muscle strength measurements during the first three waves (n=3012); (5) age <50 years (n=35); and (6) lack of follow-up information (n=424). Finally, 3550 participants from the CHARLS cohort were included in this study (**Figure 1**).

### 2.2 Assessment of walking speed and muscle strength and construction of dual trajectory models

Walking speed was assessed using standardized physical performance tests available in the HRS, ELSA, SHARE, and CHARLS cohorts. In the HRS, ELSA, and SHARE cohorts, participants were instructed to complete an 8-foot walking test at their usual pace. Walking speed was calculated by dividing the walking distance by the time required to complete the test and was expressed as meters per second (m/s). Each participant completed two trials, and the mean value was used for analysis to improve measurement reliability. In the CHARLS cohort, walking speed was assessed using a standardized 2.5-meter walking test. Participants were instructed to walk 2.5 meters at their usual pace, and the completion time was recorded. Walking speed was calculated as the distance divided by the completion time (m/s). Similarly, two trials were performed for each participant, and the average walking speed was used for subsequent analyses.

Muscle strength was assessed using grip strength measurements collected during each survey wave. In the HRS, ELSA, SHARE, and CHARLS cohorts, grip strength was measured using handheld dynamometers according to cohort-specific standardized protocols. Both hands were assessed three times, and the maximum value recorded for each hand was retained to minimize measurement error and capture maximal voluntary strength. Total grip strength was calculated as the sum of the maximum grip strength values from both hands (kg). This measure has been widely used as an indicator of overall muscle strength in epidemiological studies of older adults.

During longitudinal data preparation, participants with at least three measurements of both walking speed and muscle strength during baseline and follow-up periods were included. Follow-up time (years since baseline) was used as the time variable to construct individual-level repeated-measure datasets.

Subsequently, group-based trajectory modeling (GBTM) was applied to identify long-term patterns of change in walking speed and muscle strength and to characterize their joint longitudinal trajectories. Models with one to four trajectory classes and linear or quadratic terms were fitted. The optimal model was selected based on the Bayesian Information Criterion (BIC), within-group sample proportions, and clinical interpretability. The final trajectory classification was determined by achieving the best balance between statistical model fit and clinical relevance.

### 2.3 Assessment of ADL impairment and new-onset diabetes

Physical functional impairment was assessed using the modified Katz ADL scale in the HRS, ELSA, SHARE, and CHARLS cohorts. Although the number of ADL items varied slightly among cohorts, six core ADL indicators available across all four databases were extracted to ensure consistency and comparability. These indicators included dressing, bathing, eating, transferring in and out of bed, toileting, and indoor walking. At each follow-up assessment, participants were asked whether they experienced difficulty performing these activities without assistance. According to widely accepted definitions in international aging cohort studies, participants were classified as having ADL impairment if they reported difficulty or inability to perform any of the six activities (binary variable). Participants with ADL impairment during any of the first three waves used for trajectory construction were excluded, and only those without ADL impairment throughout this period were included.

New-onset diabetes was assessed using longitudinal follow-up data from the HRS, ELSA, SHARE, and CHARLS cohorts. Participants diagnosed with diabetes during the first three waves were excluded. New-onset diabetes was defined as the first occurrence of physician-diagnosed diabetes or self-reported diabetes diagnosis during follow-up. In each cohort, participants were asked whether they had ever been diagnosed with diabetes by a physician or healthcare professional, and the timing of diagnosis was recorded in subsequent follow-up waves. Participants who first reported diabetes during follow-up were classified as incident cases, whereas those without a diabetes diagnosis throughout follow-up were classified as non-cases. To ensure comparability across cohorts, diabetes status was consistently defined based on physician-diagnosed diabetes during follow-up. Follow-up time was calculated from the completion of the three-wave trajectory-construction period until the first occurrence of diabetes, loss to follow-up, death, or study completion, whichever occurred first.

New-onset diabetes was considered the primary outcome and was used to assess the longitudinal associations between joint trajectories of muscle strength and walking speed and diabetes risk among participants without baseline ADL impairment. To enhance robustness and comparability, outcome variables were independently constructed in each cohort using identical definitions and analytical procedures.

To ensure correct temporal ordering between exposure and outcome, participants with diabetes during any of the first three waves used for trajectory construction were excluded, and incident diabetes follow-up began after this period. This design ensured that changes in walking speed and muscle strength trajectories preceded diabetes onset and reduced the potential influence of reverse causation.

### 2.4 Covariates

Sociodemographic characteristics and lifestyle factors were collected as covariates, including age, sex, race, education, wealth, BMI, hypertension, drinking status, smoking status, cancer, cardiovascular disease (CVD), hip fracture, physical activity, cognitive function, and CESD score. Smoking status was categorized as individuals with no current smoking history and individuals with a history of smoking. Drinking status was categorized as individuals without current alcohol consumption and individuals with a history of alcohol consumption. BMI was calculated as weight (kg) divided by height squared (m²).

### 2.5 Machine learning feature preprocessing and feature selection

Machine learning models were developed as a complementary analysis to evaluate the predictive contribution of longitudinal functional trajectories and other clinical characteristics for new-onset diabetes. Feature selection was performed using a sequential screening strategy rather than independent competing approaches. First, candidate features were evaluated for multicollinearity using VIF and pairwise correlation analyses. Features retained after these steps were subsequently analyzed using both LASSO regression and the BORUTA algorithm. The final feature set for machine learning model development consisted only of variables identified by both LASSO and BORUTA, thereby retaining predictors with consistent evidence of predictive relevance. Features excluded by either method were not incorporated into subsequent machine learning or Stacking model construction. Variance inflation factors (VIFs) and correlation coefficients were calculated for all candidate features. Features with VIF values >3 or correlation coefficients >0.9 were considered to exhibit substantial multicollinearity and were excluded from subsequent analyses.

LASSO regression was applied to further select relevant features after removal of collinear variables. LASSO is an L1 regularization-based feature selection method that introduces a penalty term into the loss function to shrink regression coefficients. Coefficients of irrelevant variables are reduced toward zero, enabling automatic feature selection. The optimal penalty parameter λ was determined using cross-validation. Multiple iterative fitting procedures were performed to identify features with predictive value for new-onset diabetes. Features with non-zero coefficients were considered LASSO-selected and were subsequently intersected with the features selected by Boruta.

The BORUTA algorithm was additionally applied for feature selection after removal of collinear variables. BORUTA is a random forest-based feature selection method that evaluates feature importance by generating corresponding “shadow features” for each original variable. During the selection process, the importance of original features was compared with that of shadow features through 500 iterations until stable importance estimates were achieved. Features classified as “confirmed” by Boruta were considered Boruta-selected; only features selected by both LASSO and Boruta were retained for subsequent model development.

Because the incidence of outcome events was relatively low, substantial class imbalance existed in the training dataset. To minimize the influence of class imbalance on model training and predictive performance, the Synthetic Minority Oversampling Technique (SMOTE) combined with random undersampling was applied to resample the training dataset. Specifically, SMOTE was first used to generate synthetic minority-class samples to enhance model learning of minority patterns. Subsequently, random undersampling was applied to majority-class samples to achieve a more balanced distribution between classes. All resampling procedures and feature selection steps were conducted exclusively within the training dataset to prevent data leakage and ensure reliable model evaluation. To minimize the influence of different feature scales on model training, all continuous variables were standardized, and categorical variables were transformed using one-hot encoding.

### 2.6 Statistical analyses

Prior to statistical analysis, continuous variable distributions were assessed using the Shapiro–Wilk test alongside visual inspections of histograms and density plots (**Supplementary Figure 1**). Because the continuous variables exhibited non-normal distributions, they were expressed as medians with interquartile ranges (IQRs), and between-group comparisons were performed via the Mann–Whitney U test. Categorical variables were expressed as frequencies and percentages and compared using the chi-square test.

To identify distinct developmental patterns in walking speed and muscle strength, GBTM—a finite mixture model approach—was applied. This method classifies individuals into latent subgroups with similar developmental patterns through maximum likelihood estimation. Trajectory models were constructed separately within each cohort using data from the first three interview waves, utilizing follow-up time since baseline as the temporal variable. Models containing one to four trajectory classes were fitted. The optimal number of classes was determined based on the BIC, AIC, log-likelihood values, and mean posterior probabilities. Model selection criteria prioritized the lowest BIC value, a sample proportion greater than 5% for each trajectory class, and a mean posterior probability of 0.70 or higher. The final model was selected based on the best overall balance among statistical fit, classification accuracy, trajectory interpretability, and subgroup stability. Each participant was assigned to a corresponding trajectory group according to the maximum posterior probability principle. Detailed model selection results, including competing trajectory solutions and posterior probabilities, are presented in **Supplementary Table 5**.

Multivariable Cox proportional hazards regression models evaluated the associations between joint trajectories of walking speed and muscle strength and new-onset diabetes risk among participants without ADL impairment. Event time was defined as the interval from the completion of the first three survey waves (used for trajectory construction) to the first occurrence of diabetes, loss to follow-up, death, or the end of follow-up, whichever occurred first. Hazard ratios (HRs) and 95% confidence intervals (CIs) were calculated.

To minimize potential confounding bias caused by baseline differences among trajectory groups, inverse probability of treatment weighting (IPTW) based on propensity scores was applied. Propensity scores were estimated using multinomial logistic regression models incorporating age, sex, race, education, household wealth, BMI, hypertension, drinking status, smoking status, cancer, CVD, hip fracture, physical activity, cognitive function, and Center for Epidemiological Studies Depression (CESD) score as covariates. Stabilized IPTW was used to construct weighted cohorts, and weights were truncated at the 1st and 99th percentiles to mitigate the influence of extreme weights on estimation stability. Covariate balance before and after IPTW weighting was assessed using standardized mean differences (SMDs). Baseline balance results are presented in **Supplementary Tables 22**-**25**. Love plots illustrating covariate balance before and after weighting are shown in **Supplementary Figures 2A–D**, and histograms comparing extreme weights before and after truncation are presented in **Supplementary Figures 2E–H**.

Four sequential Cox regression models were constructed: Model 1 was an unadjusted crude model without IPTW weighting or covariate adjustment; Model 2 was a multivariable-adjusted model without IPTW weighting that controlled for all aforementioned baseline covariates; Model 3 applied an IPTW-weighted Cox framework without further covariate adjustment; and Model 4 was a doubly robust Cox model incorporating both IPTW weighting and additional adjustment for all baseline covariates. Robust sandwich variance estimators were used to obtain robust standard errors for the weighted Cox regression models.

### 2.7 Machine learning framework and benchmarking

The dataset was randomly partitioned into training and validation sets at a 7:3 ratio to prevent model overfitting. Based on the MLR3 framework, nine machine learning algorithms were constructed and compared within each cohort: AdaBoost, Decision Tree (DT), Random Forest, LightGBM, K-Nearest Neighbors (KNN), Naive Bayes, Support Vector Machine (SVM), XGBoost, and CatBoost.

Each algorithm offered distinct analytical advantages: AdaBoost iteratively trains multiple weak learners and dynamically adjusts sample weights to focus on misclassified instances, progressively optimizing predictive performance. The Decision Tree algorithm recursively selects optimal splitting variables to capture complex nonlinear relationships and feature interactions with high interpretability and visualization capability. Random Forest aggregates an ensemble of decision trees to enhance generalization and mitigate overfitting. LightGBM optimizes gradient boosting decision trees for high training efficiency, low memory usage, and parallel learning capabilities. KNN classifies samples based on proximity to neighboring data points, performing effectively with nonlinear data without requiring an explicit training phase. Naive Bayes utilizes Bayes’ theorem under the assumption of feature independence, offering high computational efficiency and robustness to missing data. SVM identifies the optimal separating hyperplane to distinguish complex decision boundaries within high-dimensional spaces. XGBoost provides a rapid, regularized gradient boosting framework highly resilient to missing values in large-scale datasets. Finally, CatBoost utilizes ordered boosting and advanced categorical feature encoding to reduce prediction bias, handle categorical variables directly, and maintain stability across small sample sizes.

Benchmarking analysis systematically compared the performance of these machine learning models on standardized datasets. Given that the machine learning task was a binary classification problem, performance was assessed using seven evaluation metrics: Accuracy, F-beta score, Area under the ROC curve (AUC), Sensitivity, Specificity, Area under the Precision–Recall curve (PR-AUC), and the Brier score. AUC served as the primary metric for identifying the optimal machine learning model, while the remaining measures provided secondary evaluation. Ten-fold cross-validation was applied to improve model stability. Analysis of variance (ANOVA) and the Kruskal–Wallis H test were used to compare differences in evaluation metrics among the machine learning models.

### 2.8 Construction of the stacking ensemble learning model

To further maximize predictive performance, a Stacking ensemble learning model was developed based on the single-model analyses. First, the seven best-performing base learners were selected according to the AUC obtained from each individual model in the validation dataset.

Subsequently, a Stacking ensemble framework was applied to construct the combined model. To maximize complementary information among different base learners, exhaustive combinations of the seven models were generated within each cohort, resulting in 120 possible base learner combinations. For each combination, out-of-fold prediction probabilities of individual base learners were first obtained through cross-validation. These predictions were then used as new meta-features and entered into the meta-learner. Given its structural stability and clinical interpretability, Logistic Regression was selected as the meta-learner to perform secondary learning on outputs from the base learners and generate final predictions.

To comprehensively evaluate the predictive performance of individual models and Stacking models, Accuracy, F-beta score, AUC, Sensitivity, Specificity, PR-AUC, and the Brier score were calculated. The 95% CIs were estimated using 1000 bootstrap resampling iterations. Furthermore, the overall performance of different models was compared using a Normalized Rank Score. Specifically, each evaluation metric was ranked separately: Accuracy, F-beta, AUC, Sensitivity, Specificity, and PR-AUC were ranked in descending order, whereas the Brier Score was ranked in ascending order. The average ranking across all evaluation metrics was calculated as the Normalized Rank Score for each model. A lower Normalized Rank Score indicated superior overall model performance, and the optimal prediction model was ultimately selected based on the lowest score.

The SHAP (SHapley Additive exPlanations) algorithm was applied to interpret the contribution of individual features in the best-performing machine learning model. SHAP is an explainable artificial intelligence approach based on the concept of Shapley values from game theory, which quantifies the marginal contribution of each feature to model predictions across all possible feature combinations, thereby improving the understandability of the model’s decision-making process.

After identifying the best-performing Stacking model in the validation dataset, calibration curves and decision curve analysis (DCA) were applied for further evaluation. Two probability recalibration methods, Platt Scaling and Isotonic Regression, were used to optimize predicted probabilities. Platt Scaling applies logistic regression-based parametric calibration to original predicted probabilities, whereas Isotonic Regression uses a non-parametric monotonic function to model the relationship between predicted probabilities and observed outcomes. After calibration, three models were compared: the original Stacking model (Original Stacking), the Platt-calibrated model (Stacking + Platt), and the Isotonic-calibrated model (Stacking + Isotonic). In addition to comparing individual models, the best-performing Stacking model and the two recalibrated Stacking models were evaluated alongside “Treat All” and “Treat None” strategies via DCA. A higher net benefit across threshold probabilities indicated greater potential clinical utility.

A series of sensitivity analyses were performed to evaluate the robustness of our findings. First, because death may act as a competing risk for the occurrence of diabetes, a competing risk regression analysis was conducted using the Fine–Gray subdistribution hazard model, with death treated as a competing event; subdistribution hazard ratios (sHRs) and 95% CIs were reported. Second, to evaluate associations under an alternative temporal framework, discrete-time logistic regression models were fitted using follow-up waves as the time unit. For this analysis, data were restructured into a person-period format, with each participant contributing one observation at each follow-up wave until the first occurrence of diabetes or censoring. Stratified analyses were conducted to examine whether the associations between joint trajectories of muscle strength and walking speed and new-onset diabetes risk remained consistent across clinically relevant population strata, including age, sex, BMI, hypertension status, and physical activity level. Finally, vertical validation was performed using the HRS and ELSA cohorts as training datasets, the SHARE cohort as the validation dataset, and the CHARLS cohort as the testing dataset to evaluate cross-population model stability.

All data processing and statistical analyses were performed using IBM SPSS Statistics (version 24.0) and R software (version 4.3.0). All statistical tests were two-sided, and a p-value less than 0.05 was considered statistically significant.

## 3 Results

### 3.1 Baseline characteristics of participants by new-onset diabetes status

**Supplementary Tables 1**-**4** present the baseline characteristics and differences between participants with and without new-onset diabetes during follow-up across the four cohorts, focusing exclusively on individuals without baseline ADL impairment.

The HRS cohort included 2,600 baseline diabetes- and ADL-free participants with a median age of 72.00 years; over a median follow-up period of 10 years, 341 individuals developed new-onset diabetes. The ELSA cohort comprised 3,043 participants with a median age of 69.00 years, yielding 265 new-onset diabetes cases over a median follow-up of 12 years. The SHARE cohort tracked 1,533 participants with a median age of 78.00 years, documenting 170 incident cases over a median follow-up of 6 years. Lastly, the CHARLS cohort enrolled 3,550 participants with a median age of 66.00 years, among whom 448 developed new-onset diabetes over a median follow-up of 8 years. To assess potential selection bias resulting from participant exclusion, baseline characteristics were additionally compared between included and excluded participants across the four cohorts (**Supplementary Table 32**). Significant differences were observed for several demographic and health-related characteristics, including age, depressive symptoms, BMI, socioeconomic status, and selected comorbidities. The magnitude and direction of these differences varied across cohorts, reflecting differences in cohort structure and missing data patterns.

Inter-group baseline comparisons revealed distinct cohort-specific profiles. In the HRS cohort, individuals who developed new-onset diabetes during follow-up were significantly younger, more likely to be female, had lower household incomes, and exhibited higher BMIs than their non-diabetic counterparts, with additional significant variations observed in race and hypertension status (**Supplementary Table 1**). In the ELSA cohort, lower income, higher BMI, history of hypertension, and lower physical activity levels were significantly associated with diabetes development (**Supplementary Table 2**). Within the SHARE cohort, new-onset diabetes cases were concentrated among younger participants and those with a higher BMI, alongside significant baseline differences in hypertension and physical activity (**Supplementary Table 3**). In the CHARLS cohort, participants who developed diabetes were predominantly female, younger, and had higher BMIs, with significant disparities also appearing across race, hypertension, smoking status, and Center for Epidemiological Studies Depression (CESD) scores (**Supplementary Table 4**).

### 3.2 Associations between joint trajectories of walking speed and muscle strength and new-onset diabetes risk

**Table 1** outlines the associations between joint walking speed and muscle strength trajectories and new-onset diabetes risk across the four geographic regions using IPTW Cox regression models. GBTM identified distinct joint developmental patterns unique to each cohort. In both the HRS and ELSA cohorts, participants partitioned into two trajectory groups: “High-level decline and low-level stability” versus “Low-level decline and low-level rise” (**Figures 2A, B**). The SHARE cohort similarly yielded two distinct pathways, classified as “High-level decline and low-level stability” and “Low-level decline and low-level rise” (**Figure 2C**). For the CHARLS cohort, the optimal solution identified “High-level decline and low-level decline” and “Low-level decline and high-level decline” groups (**Figure 2D**). Comprehensive evaluation metrics for these structural models are detailed in **Supplementary Table 5**. Although trajectory structures differed across cohorts, unfavorable functional trajectories consistently showed positive associations with incident diabetes risk. The cohort-specific trajectory patterns likely reflect differences in population characteristics, baseline age distribution, functional status, and measurement protocols. Therefore, trajectory classes should be interpreted within each cohort rather than considered as directly equivalent categories across populations. To further examine the contribution of individual functional domains, separate trajectory models based on muscle strength alone and walking speed alone were constructed in each cohort. Associations between these single-domain trajectories and incident diabetes were evaluated using the same IPTW-adjusted Cox models. The results are presented in **Supplementary Figure 14** and **Supplementary Table 33**. Single-domain trajectories showed limited and inconsistent associations across cohorts, whereas joint trajectories demonstrated more consistent associations with diabetes risk.

**Table 1:** Multivariable IPTW-Cox regression analyses examining the association between joint trajectories of muscle strength and walking speed and new-onset diabetes risk among participants without ADL impairment.

Exposure Variable	Model 1		Model 2		Model 3		Model 4	
	HR (95% CI)	P-value	HR (95% CI)	P-value	HR (95% CI)	P-value	HR (95% CI)	P-value
HRS								
Muscle strength and Walking speed trajectory								
High level decline and Low level stability	1.00 (Reference)	-	1.00 (Reference)	-	1.00 (Reference)	-	1.00 (Reference)	-
Low level decline and low level rise	4.68 (3.51 ~ 6.24)	<.001***	4.14 (3.07 ~ 5.58)	<.001***	4.67 (3.53 ~ 6.18)	<.001***	4.17 (3.10 ~ 5.61)	<.001***
ELSA								
Muscle strength and Walking speed trajectory								
High level decline and Low level stability	1.00 (Reference)	-	1.00 (Reference)	-	1.00 (Reference)	-	1.00 (Reference)	-
Low level decline and low level rise	3.70 (2.64 ~ 5.17)	<.001***	3.69 (2.53 ~ 5.38)	<.001***	3.79 (2.71 ~ 5.31)	<.001***	3.85 (2.55 ~ 5.79)	<.001***
SHARE								
Muscle strength and Walking speed trajectory								
High level decline and Low level stability	1.00 (Reference)	-	1.00 (Reference)	-	1.00 (Reference)	-	1.00 (Reference)	-
Low level decline and Low level rise	3.54 (2.43 ~ 5.15)	<.001***	3.33 (2.25 ~ 4.94)	<.001***	3.55 (2.43 ~ 5.19)	<.001***	3.38 (2.25 ~ 5.06)	<.001***
CHARLS								
Muscle strength and Walking speed trajectory								
High level decline and Low level decline	1.00 (Reference)	-	1.00 (Reference)	-	1.00 (Reference)	-	1.00 (Reference)	-
Low level decline and High level decline	2.69 (1.96 ~ 3.70)	<.001***	2.58 (1.86 ~ 3.59)	<.001***	2.70 (1.97 ~ 3.70)	<.001***	2.60 (1.87 ~ 3.61)	<.001***

Model 1: Crude.

Model 2: Adjust: Age, Sex, Race/Native, Education, Wealth (Without SHARE), BMI, Hypertension, Drinking status (Without SHARE), Smoking status, Cancer, Cardiovascular disease, Hip fracture, Physical activity, CESD, Cognitive score (Without ELSA).

Model 3: IPTW-Crude.

Model 4: IPTW- Adjust: Age, Sex, Race/Native, Education, Wealth (Without SHARE), BMI, Hypertension, Drinking status (Without SHARE), Smoking status, Cancer, Cardiovascular disease, Hip fracture, Physical activity, CESD, Cognitive score (Without ELSA).

Significance: *p < 0.05, **p < 0.01, ***p < 0.001.

**Figure 2:**
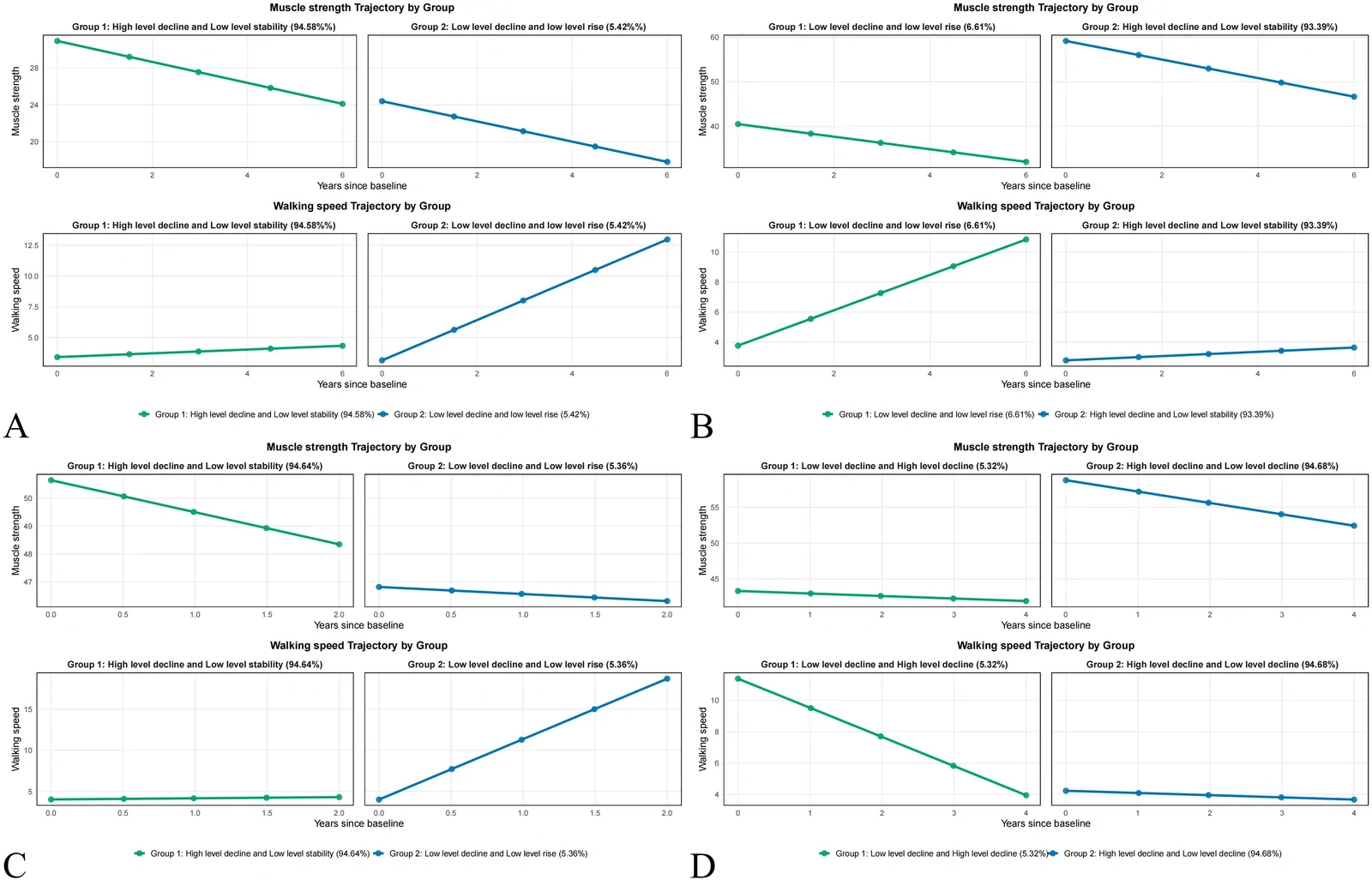
Group-based trajectory modeling (GBTM) identified the joint longitudinal trajectories of muscle strength and walking speed across different cohorts. **(A)** HRS; **(B)** ELSA; **(C)** SHARE; **(D)** CHARLS.

In the HRS cohort, using the “High-level decline and low-level stability” group as the reference category, Model 4 (the fully adjusted, IPTW-weighted model) controlled for potential confounding from age, sex, race/native status, education, wealth (excluding SHARE), BMI, hypertension, drinking status (excluding SHARE), smoking status, cancer, cardiovascular disease, hip fracture, physical activity, CESD score, and cognitive score (excluding ELSA). Participants assigned to the “Low-level decline and low-level rise” group had a significantly higher hazard of incident diabetes (adjusted HR = 4.17, 95% CI: 3.10–5.61, p < 0.001) (**Table 1**). Similar elevated risks were mirrored across the other cohorts using their respective reference groups. Compared to the “High-level decline and low-level stability” reference, the “Low-level decline and low-level rise” trajectory was tied to a significantly higher risk of new-onset diabetes in both the ELSA cohort (adjusted HR = 3.85, 95% CI: 2.55–5.79, p < 0.001) and the SHARE cohort (adjusted HR = 3.38, 95% CI: 2.25–5.06, p < 0.001). Within the CHARLS cohort, using the “High-level decline and low-level decline” group as the reference, individuals in the “Low-level decline and high-level decline” pathway had a significantly higher hazard of incident diabetes (adjusted HR = 2.60, 95% CI: 1.87–3.61, p < 0.001). IPTW-adjusted Kaplan–Meier survival curves confirmed significant differences in cumulative diabetes-free probabilities across trajectory groups for all four cohorts (all log-rank tests, p < 0.001) (**Supplementary Figure 3**).

### 3.3 Feature selection for machine learning models

**Supplementary Figure 4** delineates the feature selection pipelines conducted entirely within the respective training datasets of each cohort. **Supplementary Figures 4A, F, K, P** display the VIFs used to screen for multicollinearity. Utilizing a conservative exclusion threshold of a VIF greater than 3, no features were omitted, as all variables across the cohorts fell safely below this limit. Pairwise correlation analysis (**Supplementary Figures 4B, G, L, Q**) confirmed that all retained features maintained correlation coefficients well below the predefined collinearity exclusion threshold of 0.9.

Following collinearity screening, LASSO regression was applied to optimize variable selection using 10-fold cross-validation. Cross-validation curves demonstrated optimal model execution and minimal mean squared error at specific penalty parameters (λ) for each cohort (HRS: 0.004; ELSA: 0.003; SHARE: 0.001; CHARLS: 0.005), shrinking non-essential regression coefficients to zero (**Supplementary Figures 4C, D, H–I, M–N, R–S**).

The Boruta algorithm was then deployed as a final supervisor to lock in important features from the remaining variables. In the HRS cohort, hip fracture was identified as a “Rejected” feature regarding its predictive contribution to diabetes risk. In the ELSA cohort, race was classified as “Tentative” while hip fracture was “Rejected.” The SHARE cohort pipeline labeled race as a “Rejected” feature, while the CHARLS cohort rejected cancer. Ultimately, only variables consistently selected by both LASSO regression and BORUTA were retained for downstream machine learning model development, whereas features rejected by either method were excluded from subsequent analyses. The sequential feature screening pathway from initial candidate variables to final model inputs is summarized in **Supplementary Figure 15**.

### 3.4 Construction and evaluation of machine learning models

**Supplementary Figure 5** displays performance heatmaps for the nine distinct machine learning architectures built and tested across the training and validation splits of the four cohorts: AdaBoost, DT, Random Forest, LightGBM, KNN, Naive Bayes, SVM, XGBoost, and CatBoost. Model performance was evaluated comprehensively via the area under the receiver operating characteristic curve (AUC-ROC; **Supplementary Figures 6A–H**), area under the precision-recall curve (PR-AUC; **Supplementary Figures 6I–P**), accuracy, F-beta score, sensitivity, specificity, and the Brier score. These models were constructed independently for the HRS, ELSA, SHARE, and CHARLS databases (**Supplementary Tables 6**-**13**).

In the validation datasets, the models were prioritized based on their primary AUC-ROC performance metric. Top-performing individual models varied by cohort: Random Forest achieved optimal discrimination in the HRS dataset, AdaBoost led in the ELSA dataset, Naive Bayes demonstrated peak predictive strength in the SHARE cohort, and CatBoost excelled within the CHARLS cohort (**Supplementary Tables 6**-**13**).

To build upon single-model capabilities, the seven individual models with the highest validation AUCs were grouped into a Stacking ensemble using Logistic Regression as the meta-learner. For the HRS and CHARLS configurations, the optimal base learners selected were AdaBoost, DT, Random Forest, LightGBM, Naive Bayes, SVM, and CatBoost. The ELSA ensemble utilized AdaBoost, DT, Random Forest, LightGBM, SVM, XGBoost, and CatBoost, while the SHARE framework integrated AdaBoost, DT, Random Forest, Naive Bayes, SVM, XGBoost, and CatBoost.

Exhaustive combinatorial testing of these base learners generated 120 candidate Stacking models per cohort (**Supplementary Tables 14**-**21**). The Stacking ensembles consistently outperformed their single-model counterparts across all populations. Multiple ensemble combinations generated noticeable improvements in overall AUC, PR-AUC, and F-beta scores, illustrating that ensemble learning successfully reconciled the complementary strengths of different mathematical algorithms (**Supplementary Figure 7**). Normalized Rank Scores provided a final, holistic performance index across all evaluation metrics. Based on the lowest overall score, the optimal Stacking ensemble for the HRS cohort was AdaBoost+RF+SVM (**Figures 3A, B**); for the ELSA cohort, AdaBoost+DT+LightGBM+CatBoost (**Figures 3C, D**); for the SHARE cohort, AdaBoost+XGBoost (**Figures 3E, F**); and for the CHARLS cohort, RF+LightGBM+NaiveBayes+CatBoost (**Figures 3G, H**). The complete ROC and PR curves for all 129 generated models are compile-mapped in **Supplementary Figure 7**.

**Figure 3:**
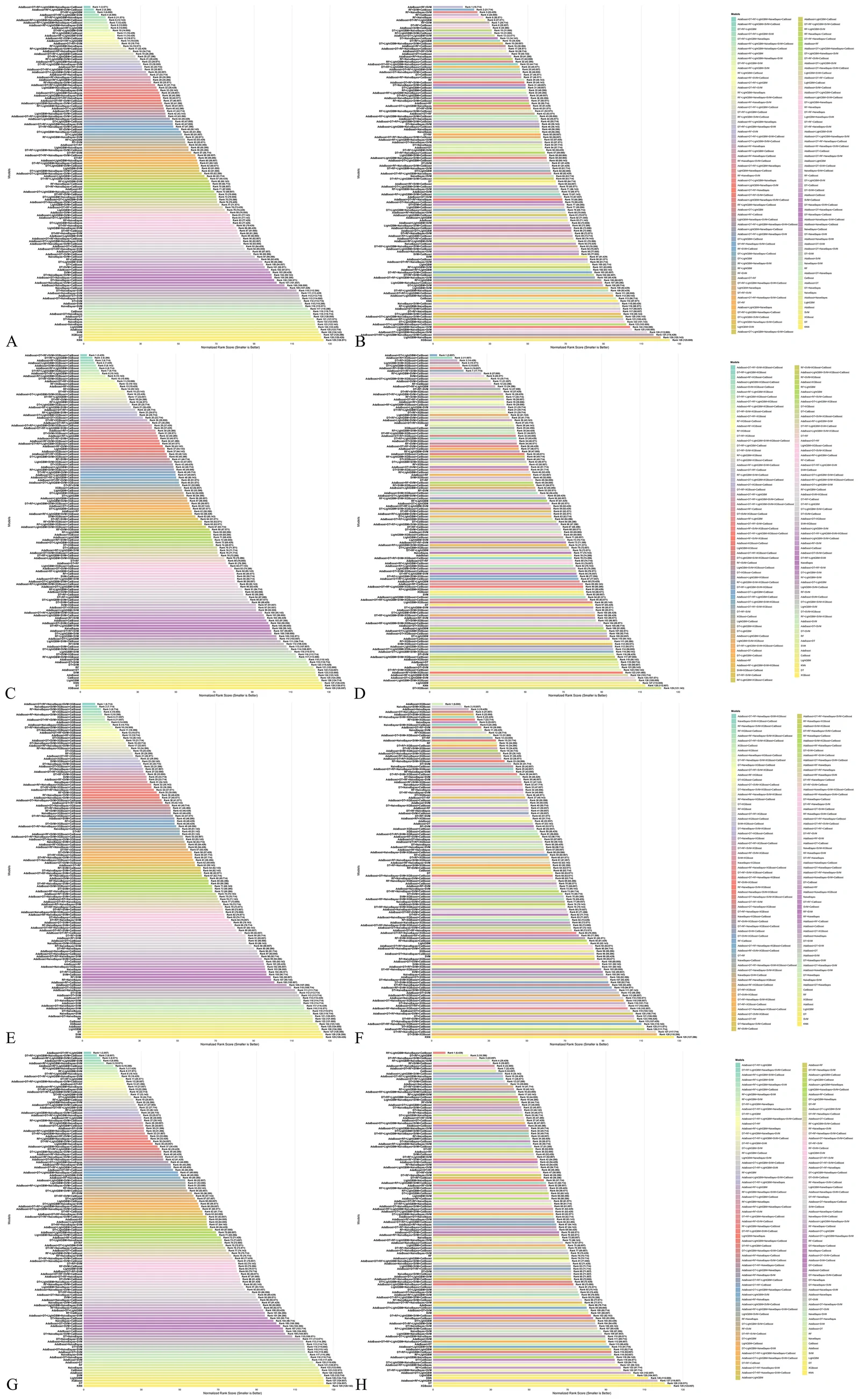
Bar plots showing the rankings of 129 machine learning models across different cohorts. **(A)** HRS training set; **(B)** HRS validation set; **(C)** ELSA training set; **(D)** ELSA validation set; **(E)** SHARE training set; **(F)** SHARE validation set; **(G)** CHARLS training set; **(H)** CHARLS validation set.

### 3.5 Interpretation of optimal machine learning models using SHAP analysis

The SHAP algorithm unmasked the directional contributions of individual features within the optimized Stacking models. **Figures 4A–D** highlight the highest-ranking predictors of new-onset diabetes across the four cohorts, utilizing SHAP values to quantify individual variable impact. Joint muscle strength and walking speed trajectories contributed to model predictions across multiple cohorts. Force and waterfall plots (**Figures 4E–L**) provide sample case-level interpretations of these optimal models, with lighter colors mapping factors that drove up the final predicted probability of diabetes. In the representative HRS model, integrating patient-specific features shifted the baseline predicted probability from 0.357 up to a final prediction of 0.544 (**Figures 4E, I**). The baseline risk in the ELSA model rose from 0.336 to 0.776 after accounting for full feature values (**Figures 4F, J**). Similarly, the SHARE baseline probability expanded from 0.405 to 0.803 (**Figures 4G, K**), and the CHARLS model shifted its prediction from a baseline of 0.407 to 0.730 upon feature integration (**Figures 4H, L**).

**Figure 4:**
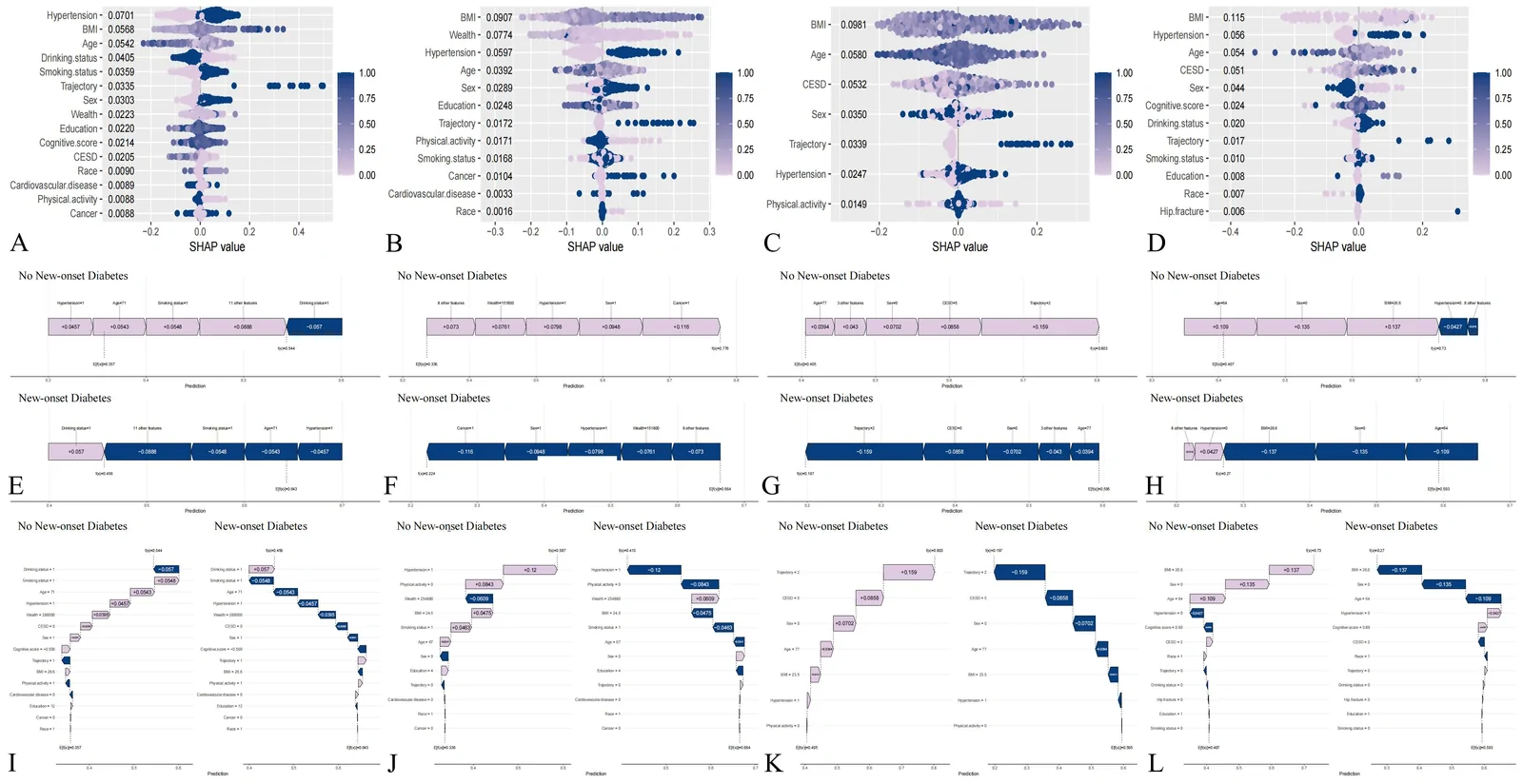
SHAP-based interpretation of the best-performing models across cohorts. **(A)** HRS SHAP beeswarm plot; **(B)** ELSA SHAP beeswarm plot; **(C)** SHARE SHAP beeswarm plot; **(D)** CHARLS SHAP beeswarm plot; **(E)** HRS force plot; **(F)** ELSA force plot; **(G)** SHARE force plot; **(H)** CHARLS force plot; **(I)** HRS waterfall plot; **(J)** ELSA waterfall plot; **(K)** SHARE waterfall plot; **(L)** CHARLS waterfall plot.

### 3.6 Calibration performance and clinical utility analysis

**Supplementary Figure 8** visually demonstrates the calibration curves and DCA profiles for the optimized Stacking models. The uncalibrated, original Stacking models maintained high baseline agreement with observed outcomes. However, post-recalibration using Platt Scaling and Isotonic Regression further smoothed prediction probabilities, with Isotonic Regression yielding the closest alignment between predicted and observed risks across all four cohorts. DCA metrics confirmed that the recalibrated Stacking models delivered superior net clinical benefits across a broad spectrum of threshold probabilities, consistently outperforming standalone models, as well as the default “Treat All” and “Treat None” clinical strategies. These findings indicate favorable discrimination and calibration of the Stacking models within the study datasets, while their clinical utility requires prospective validation.

### 3.7 Sensitivity analyses

Because mortality acts as a competing risk that can distort the true incidence of diabetes, Fine–Gray subdistribution hazard regression models were executed with death treated as a competing event (**Table 2**). The subdistribution risk estimates closely matched the primary IPTW-Cox findings. Within the fully adjusted HRS model, the “Low-level decline and low-level rise” group maintained a significantly elevated risk of new-onset diabetes compared to the “High-level decline and low-level stability” reference (adjusted sHR = 3.88, 95% CI: 2.94–5.14, p < 0.001). This pattern persisted across the remaining cohorts: the “Low-level decline and low-level rise” trajectory demonstrated significantly higher risks in the ELSA cohort (adjusted sHR = 3.37, 95% CI: 2.27–5.01, p < 0.001) and an approximate 2.18-fold higher hazard in the SHARE cohort (adjusted sHR = 3.18, 95% CI: 2.17–4.65, p < 0.001). In the CHARLS cohort, the “Low-level decline and high-level decline” group maintained a significantly elevated risk profile compared to its reference (adjusted sHR = 2.47, 95% CI: 1.81–3.37, p < 0.001). This consistency in both direction and magnitude confirms that the underlying trajectory associations are independent of competing mortality biases.

**Table 2:** Fine–Gray regression analyses examining the association between joint trajectories of muscle strength and walking speed and new-onset diabetes risk among participants without ADL impairment considering the competing risk of death.

Exposure Variable	Model 1		Model 2		Model 3		Model 4	
	sHR (95% CI)	P-value	sHR (95% CI)	P-value	sHR (95% CI)	P-value	sHR (95% CI)	P-value
HRS								
Muscle strength and Walking speed trajectory								
High level decline and Low level stability	1.00 (Reference)	-	1.00 (Reference)	-	1.00 (Reference)	-	1.00 (Reference)	-
Low level decline and low level rise	4.47 (3.43 ~ 5.84)	<.001***	3.84 (2.91 ~ 5.05)	<.001***	3.82 (2.89 ~ 5.06)	<.001***	3.88 (2.94 ~ 5.14)	<.001***
ELSA								
Muscle strength and Walking speed trajectory								
High level decline and Low level stability	1.00 (Reference)	-	1.00 (Reference)	-	1.00 (Reference)	-	1.00 (Reference)	-
Low level decline and low level rise	3.61 (2.61 ~ 5.01)	<.001***	3.62 (2.47 ~ 5.30)	<.001***	3.63 (2.49 ~ 5.31)	<.001***	3.37 (2.27 ~ 5.01)	<.001***
SHARE								
Muscle strength and Walking speed trajectory								
High level decline and Low level stability	1.00 (Reference)	-	1.00 (Reference)	-	1.00 (Reference)	-	1.00 (Reference)	-
Low level decline and Low level rise	3.38 (2.36 ~ 4.85)	<.001***	3.40 (2.35 ~ 4.92)	<.001***	3.40 (2.35 ~ 4.92)	<.001***	3.18 (2.17 ~ 4.65)	<.001***
CHARLS								
Muscle strength and Walking speed trajectory								
High level decline and Low level decline	1.00 (Reference)	-	1.00 (Reference)	-	1.00 (Reference)	-	1.00 (Reference)	-
Low level decline and High level decline	2.60 (1.93 ~ 3.50)	<.001***	2.64 (1.95 ~ 3.59)	<.001***	2.49 (1.83 ~ 3.39)	<.001***	2.47 (1.81 ~ 3.37)	<.001***

Model 1: Crude.

Model 2: Adjust: Age, Sex, Race/Native, Education, Wealth (Without SHARE), BMI.

Model 3: Adjust: Age, Sex, Race/Native, Education, Wealth (Without SHARE), BMI, Hypertension, Drinking status (Without SHARE), Smoking status.

Model 4: Adjust: Age, Sex, Race/Native, Education, Wealth (Without SHARE), BMI, Hypertension, Drinking status (Without SHARE), Smoking status, Cancer, Cardiovascular disease, Hip fracture, Physical activity, CESD, Cognitive score (Without ELSA).

Significance: *p < 0.05, **p < 0.01, ***p < 0.001.

Discrete-time logistic regression models, utilizing wave-based person-period data reformats, checked the stability of these findings under an alternative temporal modeling framework. In the HRS cohort, the “Low-level decline and low-level rise” group exhibited a 3.30-fold increased risk of diabetes compared to the reference (adjusted OR = 4.30, 95% CI: 3.09–5.91, p < 0.001). This elevated risk was sustained in the ELSA cohort (adjusted OR = 4.20, 95% CI: 2.80–6.20, p < 0.001) and the SHARE cohort (adjusted OR = 4.93, 95% CI: 3.58–6.76, p < 0.001). Similarly, the CHARLS cohort “Low-level decline and high-level decline” group showed a robust, significant risk increase (adjusted OR = 4.88, 95% CI: 2.97–7.77, p < 0.001). These congruent outcomes across distinct time-modeling strategies confirm the underlying stability of our primary calculations (**Table 3**).

**Table 3:** Discrete-time logistic regression analyses examining the association between joint trajectories of muscle strength and walking speed and new-onset diabetes risk among participants without ADL impairment under different time frameworks.

Exposure Variable	Model 1		Model 2		Model 3		Model 4	
	OR (95% CI)	P-value	OR (95% CI)	P-value	OR (95% CI)	P-value	OR (95% CI)	P-value
HRS								
Muscle strength and Walking speed trajectory								
High level decline and Low level stability	1.00 (Reference)	-	1.00 (Reference)	-	1.00 (Reference)	-	1.00 (Reference)	-
Low level decline and low level rise	4.84 (3.53 ~ 6.54)	<.001***	4.22 (3.04 ~ 5.77)	<.001***	4.19 (3.02 ~ 5.73)	<.001***	4.30 (3.09 ~ 5.91)	<.001***
ELSA								
Muscle strength and Walking speed trajectory								
High level decline and Low level stability	1.00 (Reference)	-	1.00 (Reference)	-	1.00 (Reference)	-	1.00 (Reference)	-
Low level decline and low level rise	4.10 (2.86 ~ 5.75)	<.001***	4.26 (2.85 ~ 6.25)	<.001***	4.32 (2.89 ~ 6.34)	<.001***	4.20 (2.80 ~ 6.20)	<.001***
SHARE								
Muscle strength and Walking speed trajectory								
High level decline and Low level stability	1.00 (Reference)	-	1.00 (Reference)	-	1.00 (Reference)	-	1.00 (Reference)	-
Low level decline and Low level rise	4.98 (3.68 ~ 6.70)	<.001***	5.11 (3.72 ~ 6.97)	<.001***	5.15 (3.75 ~ 7.02)	<.001***	4.93 (3.58 ~ 6.76)	<.001***
CHARLS								
Muscle strength and Walking speed trajectory								
High level decline and Low level decline	1.00 (Reference)	-	1.00 (Reference)	-	1.00 (Reference)	-	1.00 (Reference)	-
Low level decline and High level decline	5.07 (3.17 ~ 7.82)	<.001***	4.75 (2.92 ~ 7.46)	<.001***	4.86 (2.97 ~ 7.69)	<.001***	4.88 (2.97 ~ 7.77)	<.001***

Model 1: Crude.

Model 2: Adjust: Age, Sex, Race/Native, Education, Wealth (Without SHARE), BMI.

Model 3: Adjust: Age, Sex, Race/Native, Education, Wealth (Without SHARE), BMI, Hypertension, Drinking status (Without SHARE), Smoking status.

Model 4: Adjust: Age, Sex, Race/Native, Education, Wealth (Without SHARE), BMI, Hypertension, Drinking status (Without SHARE), Smoking status, Cancer, Cardiovascular disease, Hip fracture, Physical activity, CESD, Cognitive score (Without ELSA).

Significance: *p < 0.05, **p < 0.01, ***p < 0.001.

Stratified analyses were performed to evaluate the consistency of associations between joint trajectories and incident diabetes across age, sex, BMI, hypertension status, and physical activity strata (**Supplementary Figure 9**). In the HRS and ELSA cohorts, the elevated risk associated with the unfavorable “Low-level decline and low-level rise” trajectory remained stable across all age and sex strata (**Supplementary Figures 9A, B**). Within the SHARE cohort, despite slight variations in subgroup effect sizes, the directional trends were consistent; the risk was highly significant among individuals older than 70 years (HR = 4.07, 95% CI: 2.62–6.31), whereas the same positive trend in those 70 years or less did not reach statistical significance (HR = 2.22, 95% CI: 0.82–6.00). Sex-stratified analyses in SHARE confirmed a higher risk for both women (HR = 2.58, 95% CI: 1.46–4.57) and men (HR = 3.58, 95% CI: 2.04–6.29). Subgroup assessments within the CHARLS cohort further validated the primary models, showing a consistently elevated risk for the “Low-level decline and high-level decline” group across all age and sex strata (**Supplementary Figure 9D**). Additional stratified analyses according to BMI, hypertension status, and physical activity level showed generally consistent associations between unfavorable joint trajectories and incident diabetes across clinically relevant strata (**Supplementary Figure 9**). The slightly different effect estimates observed across some strata may reflect variations in baseline characteristics, event numbers, and statistical precision rather than definitive evidence of biological heterogeneity.

Finally, a vertical cross-validation framework evaluated the external transportability and generalizability of our machine learning models. The HRS and ELSA cohorts were combined as the training dataset, the SHARE cohort served as the validation set, and the CHARLS cohort functioned as an external testing database. **Supplementary Figure 10** details this feature selection and preprocessing pipeline, executed strictly inside the training split.

**Supplementary Tables 26**-**28** outline the nine algorithms’ performance across these sequential datasets. Random Forest proved most effective in both the training split (**Supplementary Table 26**, **Supplementary Figure 11A**) and the validation dataset (**Supplementary Table 27**; **Supplementary Figure 11B**). However, AdaBoost surpassed Random Forest upon exposure to the external CHARLS testing dataset, implying a superior capacity for external generalizability (**Supplementary Table 28**; **Supplementary Figure 11C**).

**Supplementary Figures 11D–I** chart the training, validation, and testing ROC and PR curves for these independent models. The seven best-performing algorithms were compiled into a vertical Stacking ensemble. On the external testing data, comprehensive benchmarking revealed that the AdaBoost+NaiveBayes+SVM ensemble configuration generated the highest predictive performance (**Supplementary Table 31**; **Supplementary Figure 12I**). Model rankings across the training and validation phases are provided in **Supplementary Tables 29** and **30** (**Supplementary Figures 12G, H**), and their corresponding ROC/PR evaluations are mapped in **Supplementary Figures 12A–F**.

SHAP analyses indicated that joint muscle strength and walking speed trajectories contributed to model predictions within the cross-cohort validation framework (**Supplementary Figures 13A, B**). DCA and calibration analyses further characterized the performance of the calibrated Stacking model.

## 4 Discussion

This study, utilizing data from four representative longitudinal aging cohorts (HRS, ELSA, SHARE, and CHARLS), systematically evaluated how joint trajectories of muscle strength and walking speed relate to new-onset diabetes risk in older adults without baseline ADL impairment. Using group-based trajectory modeling, we identified heterogeneous patterns of muscle strength and walking speed changes across these cohorts. Joint trajectories characterized by persistently low functional levels or progressive decline were consistently associated with elevated diabetes risk across different populations, although the specific trajectory patterns varied between cohorts. These associations remained robust across all four independent cohorts after adjusting for confounding via IPTW. The stability of these findings was further corroborated by Fine–Gray competing risk models, discrete-time logistic regression, and stratified analyses across multiple clinically relevant characteristics. Notably, by integrating machine learning, this study further explored how joint trajectories of muscle strength and walking speed contribute to risk-stratification models. These findings suggest that dynamic changes in muscle function represent more than passive manifestations of functional decline during aging; they serve as important early indicators for identifying individuals at high risk for metabolic disease.

The biological mechanisms linking concurrent declines in muscle strength and walking speed to diabetes development are multifaceted. Skeletal muscle is the primary site for glucose disposal, playing an essential role in maintaining systemic glucose homeostasis and insulin sensitivity (17). Age-related reductions in muscle strength often coincide with loss of muscle mass, type II fiber atrophy, mitochondrial dysfunction, and impaired intracellular insulin signaling pathways. Collectively, these alterations compromise peripheral glucose utilization and drive insulin resistance (18). Furthermore, deteriorating muscle function correlates with chronic low-grade inflammation (“inflammaging”), elevated oxidative stress, and ectopic lipid accumulation (myosteatosis)—all established drivers of metabolic dysfunction (19, 20). Concurrently, walking speed serves as an integrated indicator of lower-extremity performance, neuromuscular coordination, cardiopulmonary reserve, and overall physiological resilience (21). A progressive reduction in walking speed thus signals a subclinical depletion of multi-system functional reserves. The convergence of these muscular and systemic pathways may partly explain the observed association between concurrent functional decline and incident diabetes.

A key contribution of this study is the conceptualization of “integrated functional trajectories” as a distinct aging phenotype. Biological aging is non-linear and heterogeneous; older adults display divergent trajectories of functional deterioration shaped by genetics, lifestyle, cumulative disease burden, and environmental exposures (22, 23). While prior research has relied heavily on baseline, static measurements of grip strength or walking speed, such approaches fail to capture intra-individual dynamics over time, potentially obscuring differences in the rate, timing, and direction of functional loss (24). By applying group-based joint trajectory modeling to repeated-measures data, we identified distinct latent subgroups with specific patterns of concurrent functional change. Although trajectory classifications varied across cohorts, reflecting population-specific functional aging patterns, a consistent pattern emerged: individuals displaying persistently poor function or accelerated decline experienced higher risks of developing diabetes. This robust finding indicates that integrated trajectories effectively capture cumulative physiological deterioration, serving as a critical intermediate phenotype that connects chronological aging and physical frailty to metabolic disease (25).

Important cross-cohort differences should be considered when interpreting these findings. Although unfavorable joint trajectories were consistently associated with higher diabetes risk, trajectory structures and effect estimates varied across populations. Differences in age distribution, ethnic background, socioeconomic conditions, and metabolic profiles may partly contribute to these variations. For example, CHARLS included a relatively younger population than SHARE, whereas differences in BMI distribution and lifestyle patterns between Western and Chinese populations may influence both functional aging processes and diabetes susceptibility. In addition, methodological differences, including walking speed assessment protocols (2.5-meter walking test in CHARLS versus 8-foot walking tests in other cohorts), grip strength measurement procedures, and follow-up duration, may introduce heterogeneity in trajectory classification.

Differences in healthcare systems and diabetes detection practices may also affect outcome ascertainment across countries. Variations in healthcare accessibility, screening intensity, and disease management strategies could influence the identification of physician-diagnosed diabetes, although identical outcome definitions were applied whenever possible. Furthermore, lifestyle behaviors, including physical activity patterns, dietary habits, and broader environmental exposures, may modify the relationship between functional decline and metabolic risk. Therefore, the observed differences in hazard ratios, such as the higher estimate in HRS compared with CHARLS, should not be interpreted as indicating a preferable or optimal effect size. Instead, they likely reflect differences in population risk profiles, event rates, follow-up duration, and statistical precision. The key finding is the consistent direction of association across diverse populations, suggesting that adverse functional trajectories represent a potentially generalizable marker of diabetes vulnerability in aging populations.

These findings extend current knowledge by demonstrating the potential value of joint trajectory analysis in capturing multidimensional functional aging patterns beyond isolated functional assessments. Previous investigations have focused primarily on baseline grip strength, isolated walking speed measurements, categorical definitions of sarcopenia, or cross-sectional physical function scores (26). While these conventional indicators establish a link between muscle impairment and metabolic abnormalities, they leave critical process-oriented questions unanswered. Specifically, they cannot identify which longitudinal patterns confer the highest risk, whether an observed decline represents a permanent progression or a transient fluctuation, or whether concurrent deterioration across multiple domains exerts synergistic effects (27). By simultaneously evaluating muscle strength and walking speed over time, our framework overcomes these limitations. This dynamic approach captures the phenotypic heterogeneity of functional aging, providing a robust empirical foundation for future risk stratification based on longitudinal functional characteristics. From a clinical perspective, repeated assessment of grip strength and usual walking speed may provide a simple adjunct to conventional diabetes risk assessment in older adults. Individuals showing sustained deterioration in both domains may warrant closer evaluation of established metabolic risk factors and glycemic status, although these functional measures should complement rather than replace standard diabetes screening.

Beyond defining epidemiological associations, this study explored the potential predictive value of integrating multidimensional trajectory data with machine learning and explainable artificial intelligence. Traditional statistical models typically focus on population-level associations. Conversely, machine learning integrates high-dimensional data and maps complex, non-linear biological relationships, thereby optimizing individualized risk prediction (28, 29). We deployed multiple machine learning frameworks and optimized their predictive accuracy via a Stacking ensemble strategy. The final ensemble model exhibited excellent discrimination and robust calibration across all validation cohorts (30). Furthermore, SHAP analysis enhanced model interpretability by quantifying the contribution of joint functional trajectories and other candidate features to diabetes prediction within the machine learning framework (31). These findings suggest that dynamic functional indicators may provide complementary information within multivariable diabetes prediction models and warrant further prospective evaluation.

This study possesses several key strengths. First, the utilization of large-scale, nationally representative longitudinal cohorts from four distinct regions enhances the external validity and generalizability of the insights. Second, constructing trajectories from extensive repeated measurements improves exposure assessment accuracy and mitigates measurement errors from single-point fluctuations. Third, the application of IPTW minimizes confounding from baseline demographic and clinical variations (32). Finally, the robustness of our conclusions is validated through competing risk models, discrete-time survival analyses, and rigorous cross-cohort machine learning cross-validation (33, 34). The variation in effect estimates across strata, such as the non-significant association among younger SHARE participants, may partly reflect limited statistical power because SHARE included a relatively older population with fewer individuals in younger strata.

Several limitations warrant acknowledgment. First, given the observational design, causal relationships between physical decline and diabetes onset cannot be definitively established, leaving open the possibility of residual confounding. Second, diabetes cases were identified primarily through physician diagnosis or self-reports, which may introduce underdiagnosis or documentation lag. Moreover, the absence of laboratory biomarkers, such as fasting glucose or HbA1c, may have resulted in missed undiagnosed diabetes cases. Such nondifferential outcome misclassification could have attenuated the observed associations toward the null, suggesting that the true relationship between unfavorable functional trajectories and diabetes risk may be underestimated. Third, subtle differences in walking protocols, grip strength devices, and survey methodologies across cohorts could introduce non-differential measurement heterogeneity. Moreover, because trajectory groups were derived independently within each cohort using data-driven modeling, direct pooling of hazard ratios across cohorts was not conducted due to the cohort-specific nature of trajectory classifications. Although major demographic, socioeconomic, lifestyle, and health-related confounders were adjusted in each cohort, some covariates differed according to data availability, which may introduce residual heterogeneity when comparing effect estimates across populations. Future individual participant data meta-analyses based on predefined trajectory frameworks may further quantify overall effect estimates while accounting for between-population heterogeneity. In addition, differences in baseline survey periods and healthcare contexts across cohorts may introduce residual temporal heterogeneity, including variations in diabetes diagnosis and management practices. Selection bias is another potential limitation because participants with complete longitudinal functional assessments may differ systematically from those excluded due to missing measurements or unavailable follow-up data. Although baseline comparisons between included and excluded participants were performed, residual selection bias cannot be completely excluded, which may limit the generalizability of our findings. Fourth, although the machine learning models demonstrated stable performance during internal validation and cross-cohort evaluation, the current random training-validation partition did not fully account for temporal dependence within longitudinal cohorts. Future studies using prospective temporal validation and real-world implementation are required to further evaluate clinical utility and model transportability.

## 5 Conclusion

In conclusion, adverse joint trajectories of muscle strength and walking speed are strongly associated with an increased risk of new-onset diabetes among middle-aged and older adults without baseline ADL impairment. This association was consistently observed across four independent longitudinal cohorts spanning diverse geographic and demographic contexts, while the magnitude and trajectory structures varied across populations. By pairing machine learning with explainable artificial intelligence, this study highlights the predictive value of tracking dynamic changes in muscle function over time. Longitudinal monitoring of muscle strength and walking speed may complement conventional metabolic risk assessment and help identify older adults who may benefit from closer diabetes surveillance.

## Data Availability

The raw data supporting the conclusions of this article will be made available by the authors, without undue reservation.

## References

[1] Sun H Saeedi P Karuranga S Pinkepank M Ogurtsova K Duncan BB . IDF Diabetes Atlas: global, regional and country-level diabetes prevalence estimates for 2021 and projections for 2045. Diabetes Res Clin Pract. (2022) 183:109119. doi: 10.1016/j.diabres.2021.109119 34879977 PMC11057359

[2] Sinclair AJ Abdelhafiz AH Rodríguez-Mañas L . Frailty and sarcopenia - newly emerging and high impact complications of diabetes. J Diabetes Its Complications. (2017) 31:1465–73. doi: 10.1016/j.jdiacomp.2017.05.003 28669464

[3] Assar ME Laosa O Rodríguez Mañas L . Diabetes and frailty. Curr Opin Clin Nutr Metab Care. (2019) 22:52–7. doi: 10.1097/mco.0000000000000535 30394893

[4] Mesinovic J Zengin A De Courten B Ebeling PR Scott D . Sarcopenia and type 2 diabetes mellitus: a bidirectional relationship. Diabetes Metab Syndrome Obesity: Targets Ther. (2019) 12:1057–72. doi: 10.2147/DMSO.S186600 PMC6630094 31372016

[5] Bellary S Kyrou I Brown JE Bailey CJ . Type 2 diabetes mellitus in older adults: clinical considerations and management. Nat Rev Endocrinol. (2021) 17:534–48. doi: 10.1038/s41574-021-00512-2 34172940

[6] Cruz-Jentoft AJ Bahat G Bauer J Boirie Y Bruyère O Cederholm T . Sarcopenia: revised European consensus on definition and diagnosis. Age Ageing. (2019) 48:16–31. doi: 10.1093/ageing/afz046 30312372 PMC6322506

[7] Baskin KK Winders BR Olson EN . Muscle as a "mediator" of systemic metabolism. Cell Metab. (2015) 21:237–48. doi: 10.1016/j.cmet.2014.12.021 25651178 PMC4398026

[8] Merz KE Thurmond DC . Role of skeletal muscle in insulin resistance and glucose uptake. Compr Physiol. (2020) 10:785–809. doi: 10.1002/cphy.c190029 32940941 PMC8074531

[9] Celis-Morales CA Welsh P Lyall DM Steell L Petermann F Anderson J . Associations of grip strength with cardiovascular, respiratory, and cancer outcomes and all cause mortality: prospective cohort study of half a million UK Biobank participants. BMJ (Clinical Res Ed). (2018) 361:k1651. doi: 10.1136/bmj.k1651 29739772 PMC5939721

[10] Wu H Gu Y Wang X Meng G Rayamajhi S Thapa A . Association between handgrip strength and type 2 diabetes: a prospective cohort study and systematic review with meta-analysis. Journals Gerontology Ser A Biol Sci Med Sci. (2023) 78:1383–91. doi: 10.1093/gerona/glac241 36504134

[11] Bae KH Jo YH Lee DR Lee J . Trajectories of handgrip strength and their associations with mortality among older adults in Korea: analysis of the Korean longitudinal study of aging. Korean J Family Med. (2021) 42:38–46. doi: 10.4082/kjfm.19.0140 32456407 PMC7884901

[12] Liu J Liu W Wang L Wang N Wu L Liu X . Association of visceral adiposity index and handgrip strength with cardiometabolic multimorbidity among middle-aged and older adults: findings from Charls 2011-2020. Nutrients. (2024) 16(14):2277. doi: 10.3390/nu16142277 39064720 PMC11280108

[13] Kunutsor SK Isiozor NM Khan H Laukkanen JA . Handgrip strength-a risk indicator for type 2 diabetes: systematic review and meta-analysis of observational cohort studies. Diabetes/Metabolism Res Rev. (2021) 37:e3365. doi: 10.1002/dmrr.3365 32543028

[14] Deo RC . Machine learning in medicine. Circulation. (2015) 132:1920–30. doi: 10.1161/circulationaha.115.001593 26572668 PMC5831252

[15] Tanabe H Sato M Miyake A Shimajiri Y Ojima T Narita A . Machine learning-based reproducible prediction of type 2 diabetes subtypes. Diabetologia. (2024) 67:2446–58. doi: 10.1007/s00125-024-06248-8 39168869 PMC11519166

[16] Dinh A Miertschin S Young A Mohanty SD . A data-driven approach to predicting diabetes and cardiovascular disease with machine learning. BMC Med Inf Decis Making. (2019) 19:211. doi: 10.1186/s12911-019-0918-5 31694707 PMC6836338

[17] Petersen MC Shulman GI . Mechanisms of insulin action and insulin resistance. Physiol Rev. (2018) 98:2133–223. doi: 10.1152/physrev.00063.2017 30067154 PMC6170977

[18] Frontera WR Ochala J . Skeletal muscle: a brief review of structure and function. Calcif Tissue Int. (2015) 96:183–95. doi: 10.1007/s00223-014-9915-y 25294644

[19] Ferrucci L Fabbri E . Inflammageing: chronic inflammation in ageing, cardiovascular disease, and frailty. Nat Rev Cardiol. (2018) 15:505–22. doi: 10.1038/s41569-018-0064-2 30065258 PMC6146930

[20] Tuttle CSL Thang LAN Maier AB . Markers of inflammation and their association with muscle strength and mass: a systematic review and meta-analysis. Ageing Res Rev. (2020) 64:101185. doi: 10.1016/j.arr.2020.101185 32992047

[21] Middleton A Fritz SL Lusardi M . Walking speed: the functional vital sign. J Aging Phys Activity. (2015) 23:314–22. doi: 10.1123/japa.23.2.314 PMC4254896 24812254

[22] Ferrucci L Gonzalez-Freire M Fabbri E Simonsick E Tanaka T Moore Z . Measuring biological aging in humans: a quest. Aging Cell. (2020) 19:e13080. doi: 10.1111/acel.13080 PMC6996955 31833194

[23] Dent E Martin FC Bergman H Woo J Romero-Ortuno R Walston JD . Management of frailty: opportunities, challenges, and future directions. Lancet (London England). (2019) 394:1376–86. doi: 10.1016/s0140-6736(19)31785-4 31609229

[24] Vetrano DL Palmer K Marengoni A Marzetti E Lattanzio F Roller-Wirnsberger R . Frailty and multimorbidity: a systematic review and meta-analysis. Journals Gerontology Ser A Biol Sci Med Sci. (2019) 74:659–66. doi: 10.1093/gerona/gly110 29726918

[25] Bohannon RW . Grip strength: an indispensable biomarker for older adults. Clin Interventions Aging. (2019) 14:1681–91. doi: 10.2147/CIA.S194543 PMC6778477 31631989

[26] Cho MR Lee S Song SK . A review of sarcopenia pathophysiology, diagnosis, treatment and future direction. J Korean Med Sci. (2022) 37:e146. doi: 10.3346/jkms.2022.37.e146 35535373 PMC9091430

[27] Schrack JA Cooper R Koster A Shiroma EJ Murabito JM Rejeski WJ . Assessing daily physical activity in older adults: unraveling the complexity of monitors, measures, and methods. Journals Gerontology Ser A Biol Sci Med Sci. (2016) 71:1039–48. doi: 10.1093/gerona/glw026 26957472 PMC4945889

[28] Topol EJ . High-performance medicine: the convergence of human and artificial intelligence. Nat Med. (2019) 25:44–56. doi: 10.1038/s41591-018-0300-7 30617339

[29] Obermeyer Z Emanuel EJ . Predicting the future - big data, machine learning, and clinical medicine. N Engl J Med. (2016) 375:1216–9. doi: 10.1056/nejmp1606181 27682033 PMC5070532

[30] Pfob A Lu SC Sidey-Gibbons C . Machine learning in medicine: a practical introduction to techniques for data pre-processing, hyperparameter tuning, and model comparison. BMC Med Res Methodol. (2022) 22:282. doi: 10.1186/s12874-022-01758-8 36319956 PMC9624048

[31] Kelly CJ Karthikesalingam A Suleyman M Corrado G King D . Key challenges for delivering clinical impact with artificial intelligence. BMC Med. (2019) 17:195. doi: 10.1186/s12916-019-1426-2 31665002 PMC6821018

[32] Austin PC Stuart EA . Moving towards best practice when using inverse probability of treatment weighting (IPTW) using the propensity score to estimate causal treatment effects in observational studies. Stat Med. (2015) 34:3661–79. doi: 10.1002/sim.6607 26238958 PMC4626409

[33] Austin PC Lee DS Fine JP . Introduction to the analysis of survival data in the presence of competing risks. Circulation. (2016) 133:601–9. doi: 10.1161/circulationaha.115.017719 26858290 PMC4741409

[34] Chesnaye NC Stel VS Tripepi G Dekker FW Fu EL Zoccali C . An introduction to inverse probability of treatment weighting in observational research. Clin Kidney J. (2022) 15:14–20. doi: 10.1093/ckj/sfab158 PMC8757413 35035932

