# Supplementary material for: Beyond functional disability: longitudinal joint trajectories of muscle strength and walking speed and their association with new-onset diabetes risk among older adults without ADL impairment
Source: Front Endocrinol (Lausanne). 2026 Oct 28;17:1935223. doi: 10.3389/fendo.2026.1935223 (PMC13752118; doi:10.3389/fendo.2026.1935223)
Supplement: Supplementary file 1 [file SupplementaryFile1.docx]

Supplementary Material

## Supplementary Figures:

**Supplementary Figure 1.** Normality assessment using histograms and probability density curves.(A) HRS; (B) ELSA; (C) SHARE; (D) CHARLS.

**Supplementary Figure 2.** Love plots showing baseline covariate balance before and after IPTW adjustment, and histograms showing the distribution of extreme weights before and after truncation.(A) HRS Love plot; (B) ELSA Love plot; (C) SHARE Love plot; (D) CHARLS Love plot; (E) HRS histogram; (F) ELSA histogram; (G) SHARE histogram; (H) CHARLS histogram.

**Supplementary Figure 3.** IPTW-adjusted Kaplan–Meier curves showing survival probabilities among different trajectory groups across cohorts.(A) HRS; (B) ELSA; (C) SHARE; (D) CHARLS.

**Supplementary Figure 4.** Feature selection using variance inflation factor (VIF), correlation analysis, LASSO regression, and the BORUTA algorithm.(A) VIF plot in the HRS cohort; (B) correlation heatmap in the HRS cohort; (C) LASSO coefficient plot in the HRS cohort; (D) LASSO regularization path plot in the HRS cohort; (E) BORUTA importance plot in the HRS cohort; (F) VIF plot in the ELSA cohort; (G) correlation heatmap in the ELSA cohort; (H) LASSO coefficient plot in the ELSA cohort; (I) LASSO regularization path plot in the ELSA cohort; (J) BORUTA importance plot in the ELSA cohort; (K) VIF plot in the SHARE cohort; (L) correlation heatmap in the SHARE cohort; (M) LASSO coefficient plot in the SHARE cohort; (N) LASSO regularization path plot in the SHARE cohort; (O) BORUTA importance plot in the SHARE cohort; (P) VIF plot in the CHARLS cohort; (Q) correlation heatmap in the CHARLS cohort; (R) LASSO coefficient plot in the CHARLS cohort; (S) LASSO regularization path plot in the CHARLS cohort; (T) BORUTA importance plot in the CHARLS cohort. set.

**Supplementary Figure 5.** Heatmaps showing the performance of nine machine learning models across four different cohorts.(A) HRS training set; (B) ELSA training set; (C) SHARE training set; (D) CHARLS training set; (E) HRS validation set; (F) ELSA validation set; (G) SHARE validation set; (H) CHARLS validation set.

**Supplementary Figure 6.** ROC curves and precision–recall (PR) curves of nine machine learning models across four different cohorts.(A) HRS training set ROC curves; (B) ELSA training set ROC curves; (C) SHARE training set ROC curves; (D) CHARLS training set ROC curves; (E) HRS validation set ROC curves; (F) ELSA validation set ROC curves; (G) SHARE validation set ROC curves; (H) CHARLS validation set ROC curves; (I) HRS training set PR curves; (J) ELSA training set PR curves; (K) SHARE training set PR curves; (L) CHARLS training set PR curves; (M) HRS validation set PR curves; (N) ELSA validation set PR curves; (O) SHARE validation set PR curves; (P) CHARLS validation set PR curves.

**Supplementary Figure 7.** ROC curves and precision–recall (PR) curves of 129 machine learning models across four different cohorts.(A) HRS training set ROC curves; (B) ELSA training set ROC curves; (C) SHARE training set ROC curves; (D) CHARLS training set ROC curves; (E) HRS validation set ROC curves; (F) ELSA validation set ROC curves; (G) SHARE validation set ROC curves; (H) CHARLS validation set ROC curves; (I) HRS training set PR curves; (J) ELSA training set PR curves; (K) SHARE training set PR curves; (L) CHARLS training set PR curves; (M) HRS validation set PR curves; (N) ELSA validation set PR curves; (O) SHARE validation set PR curves; (P) CHARLS validation set PR curves.

**Supplementary Figure 8.** Calibration curves and decision curve analysis (DCA) curves evaluating the calibration performance and clinical utility of the best-performing Stacking model and nine individual models across four cohorts using Platt scaling and Isotonic regression.(A)HRS DCA curve; (B) HRS calibration curve; (C) ELSA DCA curve; (D) ELSA calibration curve; (E) SHARE DCA curve; (F) SHARE calibration curve; (G) CHARLS DCA curve; (H) CHARLS calibration curve.

**Supplementary Figure 9.** Associations between joint trajectories of muscle strength and walking speed and new-onset diabetes risk among participants without ADL impairment across different age and sex groups in four cohorts.(A) HRS; (B) ELSA; (C) SHARE; (D) CHARLS.

**Supplementary Figure 10.** Feature selection when the HRS and ELSA cohorts were used as training datasets, the SHARE cohort as the validation dataset, and the CHARLS cohort as the testing dataset.(A) VIF; (B) correlation heatmap; (C) LASSO regression; (D) BORUTA.

**Supplementary Figure 11.** Heatmaps, ROC curves, and precision–recall curves showing the performance of nine machine learning models when the HRS and ELSA cohorts were used as training datasets, the SHARE cohort as the validation dataset, and the CHARLS cohort as the testing dataset.(A) Training set heatmap; (B) validation set heatmap; (C) testing set heatmap; (D) training set ROC curves; (E) validation set ROC curves; (F) testing set ROC curves; (G) training set PR curves; (H) validation set PR curves; (I) testing set PR curves.

**Supplementary Figure 12.** ROC curves, precision–recall curves, and rankings of 129 machine learning models when the HRS and ELSA cohorts were used as training datasets, the SHARE cohort as the validation dataset, and the CHARLS cohort as the testing dataset.(A) Training set ROC curves; (B) validation set ROC curves; (C) testing set ROC curves; (D) training set PR curves; (E) validation set PR curves; (F) testing set PR curves; (G) training set rankings; (H) validation set rankings; (I) testing set rankings.

**Supplementary Figure 13.** SHAP interpretation of the best-performing model, decision curve analysis (DCA), and calibration curves assessing model stability when the HRS and ELSA cohorts were used as training datasets, the SHARE cohort as the validation dataset, and the CHARLS cohort as the testing dataset.(A) SHAP beeswarm plot; (B) waterfall plot; (C) DCA curve; (D) calibration curve.

**Supplementary Figure 14.** Cohort-specific single-domain trajectories of muscle strength and walking speed identified by group-based trajectory modeling.(A) Muscle strength trajectories in the HRS cohort;(B) Walking speed trajectories in the HRS cohort;(C) Muscle strength trajectories in the ELSA cohort;(D) Walking speed trajectories in the ELSA cohort;(E) Muscle strength trajectories in the SHARE cohort;(F) Walking speed trajectories in the SHARE cohort;(G) Muscle strength trajectories in the CHARLS cohort;(H) Walking speed trajectories in the CHARLS cohort.

**Supplementary Figure 15.** Flowchart of the sequential feature selection process for machine learning model development.
