# Supplementary material for: Beyond functional disability: longitudinal joint trajectories of muscle strength and walking speed and their association with new-onset diabetes risk among older adults without ADL impairment
Source: Front Endocrinol (Lausanne). 2026 Oct 28;17:1935223. doi: 10.3389/fendo.2026.1935223 (PMC13752118; doi:10.3389/fendo.2026.1935223)
Supplement: Supplementary file 2 [file SupplementaryFile2.docx]

Supplementary Material

## Supplementary tables:

**Supplementary Table 1.** Baseline characteristics of participants in the HRS cohort.

| **Characteristic** | **N** | **Overall**  N = 2,600 | **Normal** N = 2,259 | **New-onset Diabetes** N = 341 | **p-value** |
| --- | --- | --- | --- | --- | --- |
| Sex^b^, n(%) | 2,600 |  |  |  | 0.036 |
| FeMale |  | 1,456 (56.00%) | 1,283 (56.80%) | 173 (50.73%) |  |
| Male |  | 1,144 (44.00%) | 976 (43.20%) | 168 (49.27%) |  |
| Age(year) ^a^, M (Q1, Q3) | 2,600 | 72.00 (68.00 - 78.00) | 73.00 (68.00 - 79.00) | 71.00 (68.00 - 75.00) | <0.001 |
| Race^b^, n(%) | 2,600 |  |  |  | 0.033 |
| Non-Hispanic white |  | 2,206 (84.85%) | 1,933 (85.57%) | 273 (80.06%) |  |
| Non-Hispanic Blacks |  | 216 (8.31%) | 177 (7.84%) | 39 (11.44%) |  |
| Hispanic |  | 142 (5.46%) | 121 (5.36%) | 21 (6.16%) |  |
| Other Race |  | 36 (1.38%) | 28 (1.24%) | 8 (2.35%) |  |
| Education^b^, n(%) | 2,600 |  |  |  | 0.445 |
| High school or less |  | 529 (20.35%) | 467 (20.67%) | 62 (18.18%) |  |
| Some college |  | 1,501 (57.73%) | 1,294 (57.28%) | 207 (60.70%) |  |
| College graduate or above |  | 570 (21.92%) | 498 (22.05%) | 72 (21.11%) |  |
| Wealth ^a^, M (Q1, Q3) | 2,600 | 326,750.00 (117,100.00 - 711,450.00) | 329,500.00 (120,000.00 - 723,000.00) | 290,000.00 (102,500.00 - 603,000.00) | 0.045 |
| BMI ^a^, M (Q1, Q3) | 2,600 | 26.10 (23.50 - 29.20) | 25.80 (23.20 - 28.80) | 28.10 (25.50 - 32.10) | <0.001 |
| Hypertension ^b^, n(%) | 2,600 |  |  |  | <0.001 |
| No |  | 1,197 (46.04%) | 1,081 (47.85%) | 116 (34.02%) |  |
| Yes |  | 1,403 (53.96%) | 1,178 (52.15%) | 225 (65.98%) |  |
| Smoking status^b^, n(%) | 2,600 |  |  |  | 0.255 |
| No |  | 1,157 (44.50%) | 1,015 (44.93%) | 142 (41.64%) |  |
| Yes |  | 1,443 (55.50%) | 1,244 (55.07%) | 199 (58.36%) |  |
| Drinking status^b^, n(%) | 2,600 |  |  |  | 0.136 |
| No |  | 1,191 (45.81%) | 1,022 (45.24%) | 169 (49.56%) |  |
| Yes |  | 1,409 (54.19%) | 1,237 (54.76%) | 172 (50.44%) |  |
| Cancer ^b^, n(%) | 2,600 |  |  |  | 0.643 |
| No |  | 2,150 (82.69%) | 1,865 (82.56%) | 285 (83.58%) |  |
| Yes |  | 450 (17.31%) | 394 (17.44%) | 56 (16.42%) |  |
| Cardiovascular disease ^b^, n(%) | 2,600 |  |  |  | 0.716 |
| No |  | 1,912 (73.54%) | 1,664 (73.66%) | 248 (72.73%) |  |
| Yes |  | 688 (26.46%) | 595 (26.34%) | 93 (27.27%) |  |
| Hip fracture ^b^, n(%) | 2,600 |  |  |  | 0.082 |
| No |  | 2,544 (97.85%) | 2,206 (97.65%) | 338 (99.12%) |  |
| Yes |  | 56 (2.15%) | 53 (2.35%) | 3 (0.88%) |  |
| Physical activity | 2,600 |  |  |  | 0.820 |
| No |  | 484 (18.62%) | 419 (18.55%) | 65 (19.06%) |  |
| Yes |  | 2,116 (81.38%) | 1,840 (81.45%) | 276 (80.94%) |  |
| CESD ^a^, M (Q1, Q3) | 2,600 | 0.00 (0.00 - 1.00) | 0.00 (0.00 - 1.00) | 0.00 (0.00 - 1.00) | 0.918 |
| Cognitive score ^a^, M (Q1, Q3) | 2,600 | 0.08 (-0.41 - 0.54) | 0.08 (-0.51 - 0.54) | 0.18 (-0.40 - 0.54) | 0.347 |
| Muscle strength and Walking speed trajectory ^b^, n(%) | 2,600 |  |  |  | <0.001 |
| High level decline and Low level stability |  | 2,459 (94.58%) | 2,174 (96.24%) | 285 (83.58%) |  |
| Low level decline and low level rise |  | 141 (5.42%) | 85 (3.76%) | 56 (16.42%) |  |
| a: Kruskal-Wallis rank sum test, b: Chi-square test | | | | | |

**Supplementary Table 2.** Baseline characteristics of participants in the ELSA cohort.

| **Characteristic** | **N** | **Overall**  N = 3,043 | **Normal** N = 2,778 | **New-onset Diabetes** N = 265 | **p-value** |
| --- | --- | --- | --- | --- | --- |
| Sex^b^, n(%) | 3,043 |  |  |  | 0.326 |
| FeMale |  | 1,672 (54.95%) | 1,534 (55.22%) | 138 (52.08%) |  |
| Male |  | 1,371 (45.05%) | 1,244 (44.78%) | 127 (47.92%) |  |
| Age(year) ^a^, M (Q1, Q3) | 3,043 | 69.00 (64.00 - 75.00) | 69.00 (64.00 - 75.00) | 68.00 (64.00 - 73.00) | 0.212 |
| Race^b^, n(%) | 3,043 |  |  |  | 0.131 |
| No white |  | 37 (1.22%) | 31 (1.12%) | 6 (2.26%) |  |
| White |  | 3,006 (98.78%) | 2,747 (98.88%) | 259 (97.74%) |  |
| Education^b^, n(%) | 3,043 |  |  |  | 0.175 |
| Before high school |  | 1,492 (49.03%) | 1,349 (48.56%) | 143 (53.96%) |  |
| High school |  | 548 (18.01%) | 507 (18.25%) | 41 (15.47%) |  |
| Junior college |  | 618 (20.31%) | 562 (20.23%) | 56 (21.13%) |  |
| College graduate or above |  | 385 (12.65%) | 360 (12.96%) | 25 (9.43%) |  |
| Wealth ^a^, M (Q1, Q3) | 3,043 | 212,000.00 (120,000.00 - 359,700.00) | 217,875.00 (123,425.00 - 364,900.00) | 161,000.00 (85,000.00 - 297,100.00) | <0.001 |
| BMI ^a^, M (Q1, Q3) | 3,043 | 26.65 (24.35 - 29.61) | 26.50 (24.14 - 29.23) | 29.40 (26.43 - 32.14) | <0.001 |
| Hypertension ^b^, n(%) | 3,043 |  |  |  | <0.001 |
| No |  | 1,839 (60.43%) | 1,723 (62.02%) | 116 (43.77%) |  |
| Yes |  | 1,204 (39.57%) | 1,055 (37.98%) | 149 (56.23%) |  |
| Smoking status^b^, n(%) | 3,043 |  |  |  | 0.141 |
| No |  | 1,173 (38.55%) | 1,082 (38.95%) | 91 (34.34%) |  |
| Yes |  | 1,870 (61.45%) | 1,696 (61.05%) | 174 (65.66%) |  |
| Drinking status^b^, n(%) | 3,043 |  |  |  | 0.737 |
| No |  | 270 (8.87%) | 245 (8.82%) | 25 (9.43%) |  |
| Yes |  | 2,773 (91.13%) | 2,533 (91.18%) | 240 (90.57%) |  |
| Cancer ^b^, n(%) | 3,043 |  |  |  | 0.446 |
| No |  | 2,787 (91.59%) | 2,541 (91.47%) | 246 (92.83%) |  |
| Yes |  | 256 (8.41%) | 237 (8.53%) | 19 (7.17%) |  |
| Cardiovascular disease ^b^, n(%) | 3,043 |  |  |  | 0.141 |
| No |  | 2,941 (96.65%) | 2,689 (96.80%) | 252 (95.09%) |  |
| Yes |  | 102 (3.35%) | 89 (3.20%) | 13 (4.91%) |  |
| Hip fracture ^b^, n(%) | 3,043 |  |  |  | 0.252 |
| No |  | 3,021 (99.28%) | 2,756 (99.21%) | 265 (100.00%) |  |
| Yes |  | 22 (0.72%) | 22 (0.79%) | 0 (0.00%) |  |
| Physical activity ^b^, n(%) | 3,043 |  |  |  | <0.001 |
| No |  | 490 (16.10%) | 426 (15.33%) | 64 (24.15%) |  |
| Yes |  | 2,553 (83.90%) | 2,352 (84.67%) | 201 (75.85%) |  |
| CESD ^a^, M (Q1, Q3) | 3,043 | 1.00 (0.00 - 2.00) | 1.00 (0.00 - 2.00) | 1.00 (0.00 - 2.00) | 0.166 |
| Muscle strength and Walking speed trajectory ^b^, n(%) | 3,043 |  |  |  | <0.001 |
| High level decline and Low level stability |  | 2,842 (93.39%) | 2,618 (94.24%) | 224 (84.53%) |  |
| Low level decline and low level rise |  | 201 (6.61%) | 160 (5.76%) | 41 (15.47%) |  |
| a: Kruskal-Wallis rank sum test, b: Chi-square test | | | | | |

**Supplementary Table 3.** Baseline characteristics of participants in the SHARE cohort.

| **Characteristic** | **N** | **Overall**  N = 1,533 | **Normal** N = 1,363 | **New-onset Diabetes** N = 170 | **p-value** |
| --- | --- | --- | --- | --- | --- |
| Sex^b^, n(%) | 1,533 |  |  |  | 0.256 |
| FeMale |  | 865 (56.43%) | 776 (56.93%) | 89 (52.35%) |  |
| Male |  | 668 (43.57%) | 587 (43.07%) | 81 (47.65%) |  |
| Age(year) ^a^, M (Q1, Q3) | 1,533 | 78.00 (76.00 - 82.00) | 79.00 (76.00 - 82.00) | 77.00 (76.00 - 81.00) | <0.001 |
| Native ^b^, n(%) | 1,533 |  |  |  | 0.722 |
| No |  | 21 (1.37%) | 20 (1.47%) | 1 (0.59%) |  |
| Yes |  | 1,512 (98.63%) | 1,343 (98.53%) | 169 (99.41%) |  |
| Education^b^, n(%) | 1,533 |  |  |  | 0.376 |
| Before high school |  | 1,046 (68.23%) | 922 (67.64%) | 124 (72.94%) |  |
| High school and vocational training |  | 308 (20.09%) | 279 (20.47%) | 29 (17.06%) |  |
| Higher Education |  | 179 (11.68%) | 162 (11.89%) | 17 (10.00%) |  |
| BMI ^a^, M (Q1, Q3) | 1,533 | 25.39 (23.15 - 27.77) | 25.25 (23.01 - 27.64) | 26.40 (24.80 - 29.59) | <0.001 |
| Hypertension ^b^, n(%) | 1,533 |  |  |  | 0.025 |
| No |  | 898 (58.58%) | 812 (59.57%) | 86 (50.59%) |  |
| Yes |  | 635 (41.42%) | 551 (40.43%) | 84 (49.41%) |  |
| Smoking status^b^, n(%) | 1,533 |  |  |  | 0.437 |
| No |  | 908 (59.23%) | 812 (59.57%) | 96 (56.47%) |  |
| Yes |  | 625 (40.77%) | 551 (40.43%) | 74 (43.53%) |  |
| Cancer ^b^, n(%) | 1,533 |  |  |  | 0.406 |
| No |  | 1,438 (93.80%) | 1,281 (93.98%) | 157 (92.35%) |  |
| Yes |  | 95 (6.20%) | 82 (6.02%) | 13 (7.65%) |  |
| Hip fracture r ^b^, n(%) | 1,533 |  |  |  | 0.615 |
| No |  | 1,492 (97.33%) | 1,325 (97.21%) | 167 (98.24%) |  |
| Yes |  | 41 (2.67%) | 38 (2.79%) | 3 (1.76%) |  |
| Physical activity ^b^, n(%) | 1,533 |  |  |  | 0.017 |
| No |  | 285 (18.59%) | 242 (17.75%) | 43 (25.29%) |  |
| Yes |  | 1,248 (81.41%) | 1,121 (82.25%) | 127 (74.71%) |  |
| CES-D ^a^, M (Q1, Q3) | 1,533 | 2.00 (1.00 - 4.00) | 2.00 (1.00 - 4.00) | 2.00 (1.00 - 4.00) | 0.089 |
| Muscle strength and Walking speed trajectory ^b^, n(%) | 1,533 |  |  |  | <0.001 |
| High level decline and Low level stability |  | 1,426 (93.02%) | 1,290 (94.64%) | 136 (80.00%) |  |
| Low level decline and Low level rise |  | 107 (6.98%) | 73 (5.36%) | 34 (20.00%) |  |
| a: Student t-test, b: Chi-square test, SD: standard deviation | | | | | |

**Supplementary Table 4.** Baseline characteristics of participants in the CHARLS cohort.

| **Characteristic** | **N** | **Overall**  N =3,550 | **Normal** N = 3,102 | **New-onset Diabetes** N =448 | **p-value** |
| --- | --- | --- | --- | --- | --- |
| Sex^b^, n(%) | 3,550 |  |  |  | <0.001 |
| FeMale |  | 1,664 (46.87%) | 1,408 (45.39%) | 256 (57.14%) |  |
| Male |  | 1,886 (53.13%) | 1,694 (54.61%) | 192 (42.86%) |  |
| Age(year) ^a^, M (Q1, Q3) | 3,550 | 66.00 (62.00 - 71.00) | 66.00 (62.00 - 71.00) | 65.00 (62.00 - 70.00) | 0.002 |
| Race ^b^, n(%) | 3,550 |  |  |  | 0.026 |
| Other |  | 209 (5.89%) | 193 (6.22%) | 16 (3.57%) |  |
| Han Chinese |  | 3,341 (94.11%) | 2,909 (93.78%) | 432 (96.43%) |  |
| Education^b^, n(%) | 3,550 |  |  |  | 0.192 |
| Before high school |  | 3,354 (94.48%) | 2,935 (94.62%) | 419 (93.53%) |  |
| High school and vocational training |  | 148 (4.17%) | 123 (3.97%) | 25 (5.58%) |  |
| Higher Education |  | 48 (1.35%) | 44 (1.42%) | 4 (0.89%) |  |
| Wealth ^a^, M (Q1, Q3) | 3,550 | 9,090.91 (1,893.94 - 28,977.27) | 8,958.33 (1,856.06 - 28,409.09) | 10,482.95 (2,149.62 - 31,859.85) | 0.196 |
| BMI ^a^, M (Q1, Q3) | 3,550 | 22.32 (20.08 - 24.85) | 22.12 (19.94 - 24.55) | 24.03 (21.60 - 26.68) | <0.001 |
| Hypertension ^b^, n(%) | 3,550 |  |  |  | <0.001 |
| No |  | 2,544 (71.66%) | 2,287 (73.73%) | 257 (57.37%) |  |
| Yes |  | 1,006 (28.34%) | 815 (26.27%) | 191 (42.63%) |  |
| Smoking status^b^, n(%) | 3,550 |  |  |  | <0.001 |
| No |  | 1,979 (55.75%) | 1,690 (54.48%) | 289 (64.51%) |  |
| Yes |  | 1,571 (44.25%) | 1,412 (45.52%) | 159 (35.49%) |  |
| Drinking status ^b^, n(%) | 3,550 |  |  |  | 0.243 |
| No |  | 2,081 (58.62%) | 1,807 (58.25%) | 274 (61.16%) |  |
| Yes |  | 1,469 (41.38%) | 1,295 (41.75%) | 174 (38.84%) |  |
| Cancer ^b^, n(%) | 3,550 |  |  |  | 0.522 |
| No |  | 3,527 (99.35%) | 3,083 (99.39%) | 444 (99.11%) |  |
| Yes |  | 23 (0.65%) | 19 (0.61%) | 4 (0.89%) |  |
| Cardiovascular disease ^b^, n(%) | 3,550 |  |  |  | 0.104 |
| No |  | 3,030 (85.35%) | 2,659 (85.72%) | 371 (82.81%) |  |
| Yes |  | 520 (14.65%) | 443 (14.28%) | 77 (17.19%) |  |
| Hip fracture ^b^, n(%) | 3,550 |  |  |  | 0.098 |
| No |  | 3,501 (98.62%) | 3,063 (98.74%) | 438 (97.77%) |  |
| Yes |  | 49 (1.38%) | 39 (1.26%) | 10 (2.23%) |  |
| Physical activity ^b^, n(%) | 3,550 |  |  |  | 0.444 |
| No |  | 2,537 (71.46%) | 2,210 (71.24%) | 327 (72.99%) |  |
| Yes |  | 1,013 (28.54%) | 892 (28.76%) | 121 (27.01%) |  |
| CESD ^a^, M (Q1, Q3) | 3,550 | 7.00 (3.00 - 11.00) | 7.00 (3.00 - 11.00) | 8.00 (3.50 - 13.00) | 0.008 |
| Cognitive score ^a^, M (Q1, Q3) | 3,550 | 0.01 (-0.76 - 0.60) | 0.01 (-0.85 - 0.60) | 0.01 (-0.73 - 0.51) | 0.965 |
| Muscle strength and Walking speed trajectory ^b^, n(%) | 3,550 |  |  |  | <0.001 |
| High level decline and Low level decline |  | 3,361 (94.68%) | 2,955 (95.26%) | 406 (90.63%) |  |
| Low level decline and High level decline |  | 189 (5.32%) | 147 (4.74%) | 42 (9.38%) |  |
| a: Kruskal-Wallis rank sum test, b: Chi-square test | | | | | |

**Supplementary Table 5.** Model selection results of GBTM across different cohorts.

| **Model** | **AIC** | **BIC** | **Log-Likelihood** | **Group** | **Sample Size (n)** | **Proportion (%)** | **Average Posterior Probability** |
| --- | --- | --- | --- | --- | --- | --- | --- |
| HRS |  |  |  |  |  |  |  |
| 1-class | 118,579.1 | 118,649.5 | -59,277.57 |  |  |  |  |
| 2-class | 117,418.1 | 117,517.8 | -58,692.07 | 1 | 2,459 | 94.58% | 0.998 |
|  |  |  |  | 2 | 141 | 5.42% | 0.952 |
| 3-class | 114,828.1 | 114,957.1 | -57,392.06 | 1 | 2,491 | 95.81% | 0.998 |
|  |  |  |  | 2 | 11 | 0.42% | 1.000 |
|  |  |  |  | 3 | 98 | 3.77% | 0.952 |
| 4-class | 114,253.7 | 114,412.0 | -57,099.84 | 1 | 1,740 | 66.92% | 0.945 |
|  |  |  |  | 2 | 94 | 3.62% | 0.939 |
|  |  |  |  | 3 | 11 | 0.42% | 1.000 |
|  |  |  |  | 4 | 755 | 29.04% | 0.897 |
| ELSA |  |  |  |  |  |  |  |
| 1-class | 143,496.1 | 143,568.4 | -71,736.07 |  |  |  |  |
| 2-class | 142,197.9 | 142,300.2 | -71,081.94 | 1 | 201 | 6.61% | 0.929 |
|  |  |  |  | 2 | 2,842 | 93.39% | 0.993 |
| 3-class | 141,543.2 | 141,675.6 | -70,749.59 | 1 | 82 | 2.69% | 0.964 |
|  |  |  |  | 2 | 2,529 | 83.11% | 0.974 |
|  |  |  |  | 3 | 432 | 14.2% | 0.888 |
| 4-class | 141,129.1 | 141,291.6 | -70,537.54 | 1 | 83 | 2.73% | 0.981 |
|  |  |  |  | 2 | 2,473 | 81.27% | 0.976 |
|  |  |  |  | 3 | 5 | 0.16% | 1.000 |
|  |  |  |  | 4 | 482 | 15.84% | 0.897 |
| SHARE |  |  |  |  |  |  |  |
| 1-class | 40,662.27 | 40,726.29 | -20,319.13 |  |  |  |  |
| 2-class | 39,646.86 | 39,737.55 | -19,806.43 | 1 | 1,290 | 94.64% | 0.996 |
|  |  |  |  | 2 | 73 | 5.36% | 0.955 |
| 3-class | 39,144.36 | 39,261.73 | -19,550.18 | 1 | 57 | 3.72% | 0.994 |
|  |  |  |  | 2 | 1,336 | 87.15% | 0.991 |
|  |  |  |  | 3 | 140 | 9.13% | 0.935 |
| 4-class | 39,484.25 | 39,628.30 | -19,715.13 | 1 | 77 | 5.02% | 0.966 |
|  |  |  |  | 2 | 44 | 2.87% | 0.583 |
|  |  |  |  | 3 | 949 | 61.9% | 0.856 |
|  |  |  |  | 4 | 463 | 30.2% | 0.652 |
| CHARLS |  |  |  |  |  |  |  |
| 1-class | 131,051.4 | 131,125.5 | -65,513.69 |  |  |  |  |
| 2-class | 129,806.0 | 129,911.0 | -64,886.00 | 1 | 189 | 5.32% | 0.914 |
|  |  |  |  | 2 | 3,361 | 94.68% | 0.995 |
| 3-class | 129,363.8 | 129,499.6 | -64,659.90 | 1 | 1,498 | 42.2% | 0.858 |
|  |  |  |  | 2 | 1,939 | 54.62% | 0.864 |
|  |  |  |  | 3 | 113 | 3.18% | 0.936 |
| 4-class | 128,578.6 | 128,745.4 | -64,262.32 | 1 | 2,783 | 78.39% | 0.877 |
|  |  |  |  | 2 | 93 | 2.62% | 0.840 |
|  |  |  |  | 3 | 602 | 16.96% | 0.805 |
|  |  |  |  | 4 | 72 | 2.03% | 0.884 |

**Supplementary Table 6.** Benchmarking analysis of machine learning models in the HRS training set.

| **Model** | **Accuracy** | **F Beta** | **Area under the ROC curve** | **Sensitivity** | **Specificity** | **Area under the PR curve** | **Brier** |
| --- | --- | --- | --- | --- | --- | --- | --- |
| AdaBoost | 0.618 (0.576-0.660) | 0.462 (0.411-0.512) | 0.674 (0.640-0.708) | 0.650 (0.567-0.734) | 0.612 (0.556-0.668) | 0.294 (0.237-0.350) | 0.224 (0.211-0.237) |
| DT | 0.668 (0.642-0.694) | 0.395 (0.357-0.433) | 0.622 (0.602-0.641) | 0.505 (0.450-0.560) | 0.696 (0.662-0.730) | 0.263 (0.224-0.303) | 0.230 (0.217-0.244) |
| Random Forest | 0.753 (0.733-0.774) | 0.395 (0.362-0.428) | 0.681 (0.646-0.716) | 0.447 (0.399-0.496) | 0.805 (0.786-0.824) | 0.307 (0.274-0.341) | 0.178 (0.168-0.187) |
| Light GBM | 0.726 (0.693-0.758) | 0.372 (0.314-0.430) | 0.654 (0.613-0.694) | 0.433 (0.368-0.498) | 0.774 (0.736-0.813) | 0.261 (0.229-0.294) | 0.194 (0.178-0.209) |
| KNN | 0.623 (0.596-0.650) | 0.405 (0.367-0.444) | 0.612 (0.591-0.633) | 0.549 (0.501-0.598) | 0.635 (0.604-0.666) | 0.221 (0.193-0.249) | 0.273 (0.259-0.287) |
| Naive Bayes | 0.730 (0.703-0.757) | 0.379 (0.311-0.448) | 0.640 (0.601-0.679) | 0.443 (0.355-0.530) | 0.778 (0.753-0.804) | 0.267 (0.228-0.306) | 0.208 (0.192-0.223) |
| SVM | 0.673 (0.652-0.693) | 0.409 (0.375-0.443) | 0.639 (0.602-0.676) | 0.522 (0.474-0.570) | 0.698 (0.677-0.719) | 0.256 (0.215-0.296) | 0.212 (0.201-0.223) |
| XGBoost | 0.703 (0.675-0.732) | 0.346 (0.312-0.379) | 0.630 (0.593-0.667) | 0.410 (0.366-0.454) | 0.753 (0.727-0.779) | 0.248 (0.220-0.275) | 0.233 (0.213-0.252) |
| CATBoost | 0.735 (0.707-0.763) | 0.373 (0.336-0.411) | 0.666 (0.633-0.698) | 0.428 (0.381-0.476) | 0.786 (0.759-0.814) | 0.307 (0.255-0.360) | 0.177 (0.164-0.189) |
| P | <.001^a^ | <.001^a^ | <.001^b^ | <.001^a^ | <.001^a^ | <.001^a^ | <.001^a^ |
| ^a^ANOVA test; ^b^Kruskal-Wallis | | |  |  |  |  |  |

**Supplementary Table 7.** Benchmarking analysis of machine learning models in the HRS validation set.

| **Model** | **Accuracy** | **F Beta** | **Area under the ROC curve** | **Sensitivity** | **Specificity** | **Area under the PR curve** | **Brier** |
| --- | --- | --- | --- | --- | --- | --- | --- |
| AdaBoost | 0.584 (0.559-0.610) | 0.454 (0.401-0.500) | 0.703 (0.660-0.743) | 0.779 (0.712-0.851) | 0.562 (0.534-0.588) | 0.221 (0.162-0.282) | 0.241 (0.232-0.250) |
| DT | 0.702 (0.678-0.725) | 0.382 (0.320-0.440) | 0.692 (0.641-0.740) | 0.531 (0.452-0.612) | 0.721 (0.697-0.747) | 0.286 (0.213-0.358) | 0.222 (0.211-0.233) |
| Random Forest | 0.791 (0.771-0.813) | 0.339 (0.271-0.406) | 0.705 (0.662-0.755) | 0.395 (0.310-0.479) | 0.837 (0.816-0.857) | 0.228 (0.166-0.287) | 0.171 (0.163-0.178) |
| Light GBM | 0.745 (0.722-0.768) | 0.334 (0.269-0.396) | 0.670 (0.623-0.720) | 0.421 (0.340-0.500) | 0.783 (0.760-0.805) | 0.245 (0.174-0.309) | 0.183 (0.169-0.196) |
| KNN | 0.619 (0.594-0.646) | 0.355 (0.297-0.408) | 0.648 (0.596-0.704) | 0.553 (0.473-0.640) | 0.627 (0.600-0.652) | 0.197 (0.149-0.245) | 0.273 (0.255-0.289) |
| Naive Bayes | 0.761 (0.737-0.785) | 0.341 (0.272-0.406) | 0.694 (0.647-0.742) | 0.419 (0.333-0.508) | 0.801 (0.778-0.825) | 0.308 (0.222-0.387) | 0.184 (0.169-0.201) |
| SVM | 0.677 (0.653-0.703) | 0.372 (0.309-0.434) | 0.680 (0.631-0.727) | 0.534 (0.449-0.625) | 0.694 (0.667-0.721) | 0.222 (0.166-0.288) | 0.201 (0.191-0.211) |
| XGBoost | 0.705 (0.683-0.730) | 0.229 (0.162-0.288) | 0.598 (0.550-0.646) | 0.295 (0.210-0.370) | 0.753 (0.729-0.778) | 0.137 (0.107-0.170) | 0.232 (0.213-0.250) |
| CATBoost | 0.745 (0.723-0.768) | 0.307 (0.240-0.371) | 0.669 (0.624-0.715) | 0.384 (0.302-0.463) | 0.787 (0.766-0.808) | 0.205 (0.151-0.266) | 0.175 (0.165-0.185) |
| P | <.001^a^ | <.001^a^ | <.001^b^ | <.001^a^ | <.001^a^ | <.001^a^ | <.001^a^ |
| ^a^ANOVA test; ^b^Kruskal-Wallis | | |  |  |  |  |  |

**Supplementary Table 8.** Benchmarking analysis of machine learning models in the ELSA training set.

| **Model** | **Accuracy** | **F Beta** | **Area under the ROC curve** | **Sensitivity** | **Specificity** | **Area under the PR curve** | **Brier** |
| --- | --- | --- | --- | --- | --- | --- | --- |
| AdaBoost | 0.662 (0.621-0.703) | 0.371 (0.330-0.411) | 0.677 (0.637-0.716) | 0.586 (0.521-0.652) | 0.669 (0.622-0.717) | 0.209 (0.158-0.261) | 0.221 (0.202-0.239) |
| DT | 0.603 (0.565-0.642) | 0.354 (0.316-0.391) | 0.633 (0.603-0.664) | 0.607 (0.554-0.660) | 0.603 (0.560-0.646) | 0.144 (0.125-0.162) | 0.238 (0.226-0.250) |
| Random Forest | 0.706 (0.682-0.730) | 0.337 (0.300-0.374) | 0.669 (0.625-0.712) | 0.491 (0.419-0.564) | 0.729 (0.706-0.752) | 0.195 (0.144-0.245) | 0.188 (0.180-0.196) |
| Light GBM | 0.680 (0.654-0.705) | 0.334 (0.277-0.390) | 0.641 (0.589-0.693) | 0.508 (0.411-0.605) | 0.698 (0.673-0.723) | 0.187 (0.137-0.237) | 0.221 (0.206-0.237) |
| KNN | 0.637 (0.603-0.670) | 0.345 (0.317-0.372) | 0.634 (0.613-0.656) | 0.562 (0.513-0.611) | 0.644 (0.605-0.682) | 0.150 (0.109-0.191) | 0.257 (0.239-0.275) |
| Naive Bayes | 0.785 (0.745-0.826) | 0.294 (0.208-0.380) | 0.669 (0.641-0.696) | 0.385 (0.254-0.515) | 0.827 (0.769-0.885) | 0.175 (0.138-0.213) | 0.182 (0.156-0.209) |
| SVM | 0.678 (0.649-0.707) | 0.355 (0.305-0.405) | 0.681 (0.645-0.716) | 0.546 (0.456-0.635) | 0.691 (0.662-0.720) | 0.193 (0.158-0.229) | 0.203 (0.192-0.214) |
| XGBoost | 0.669 (0.648-0.690) | 0.325 (0.275-0.376) | 0.624 (0.582-0.666) | 0.500 (0.429-0.570) | 0.686 (0.663-0.709) | 0.172 (0.126-0.218) | 0.270 (0.253-0.287) |
| CATBoost | 0.692 (0.671-0.712) | 0.305 (0.236-0.375) | 0.650 (0.596-0.705) | 0.452 (0.334-0.571) | 0.716 (0.699-0.734) | 0.179 (0.134-0.223) | 0.194 (0.182-0.205) |
| P | <.001^a^ | <.001^a^ | <.001^b^ | <.001^a^ | <.001^a^ | <.001^a^ | <.001^a^ |
| ^a^ANOVA test; ^b^Kruskal-Wallis | | |  |  |  |  |  |

**Supplementary Table 9.** Benchmarking analysis of machine learning models in the ELSA validation set.

| **Model** | **Accuracy** | **F Beta** | **Area under the ROC curve** | **Sensitivity** | **Specificity** | **Area under the PR curve** | **Brier** |
| --- | --- | --- | --- | --- | --- | --- | --- |
| AdaBoost | 0.674 (0.651-0.701) | 0.385 (0.326-0.439) | 0.708 (0.665-0.754) | 0.668 (0.578-0.762) | 0.675 (0.651-0.702) | 0.133 (0.103-0.163) | 0.206 (0.198-0.214) |
| DT | 0.679 (0.656-0.701) | 0.343 (0.278-0.399) | 0.689 (0.641-0.736) | 0.579 (0.486-0.667) | 0.687 (0.663-0.711) | 0.152 (0.107-0.198) | 0.214 (0.203-0.226) |
| Random Forest | 0.753 (0.732-0.774) | 0.322 (0.255-0.385) | 0.699 (0.650-0.749) | 0.464 (0.370-0.564) | 0.777 (0.756-0.797) | 0.195 (0.126-0.271) | 0.175 (0.168-0.183) |
| Light GBM | 0.710 (0.688-0.733) | 0.291 (0.224-0.347) | 0.660 (0.608-0.705) | 0.452 (0.351-0.538) | 0.731 (0.708-0.754) | 0.137 (0.096-0.179) | 0.206 (0.192-0.219) |
| KNN | 0.660 (0.637-0.683) | 0.317 (0.254-0.375) | 0.629 (0.580-0.683) | 0.547 (0.457-0.636) | 0.669 (0.644-0.693) | 0.115 (0.086-0.146) | 0.255 (0.239-0.270) |
| Naive Bayes | 0.833 (0.815-0.850) | 0.215 (0.146-0.280) | 0.577 (0.515-0.638) | 0.246 (0.167-0.326) | 0.881 (0.864-0.898) | 0.105 (0.078-0.130) | 0.158 (0.142-0.173) |
| SVM | 0.661 (0.637-0.684) | 0.365 (0.311-0.418) | 0.681 (0.628-0.732) | 0.640 (0.553-0.721) | 0.662 (0.637-0.688) | 0.158 (0.115-0.205) | 0.209 (0.200-0.219) |
| XGBoost | 0.688 (0.666-0.710) | 0.335 (0.275-0.393) | 0.695 (0.647-0.742) | 0.553 (0.465-0.650) | 0.699 (0.678-0.723) | 0.176 (0.122-0.227) | 0.239 (0.222-0.256) |
| CATBoost | 0.706 (0.684-0.730) | 0.320 (0.255-0.379) | 0.665 (0.615-0.719) | 0.509 (0.417-0.605) | 0.722 (0.700-0.747) | 0.126 (0.093-0.160) | 0.184 (0.174-0.194) |
| P | <.001^a^ | <.001^a^ | <.001^b^ | <.001^a^ | <.001^a^ | <.001^a^ | <.001^a^ |
| ^a^ANOVA test; ^b^Kruskal-Wallis | | |  |  |  |  |  |

**Supplementary Table 10.** Benchmarking analysis of machine learning models in the SHARE training set.

| **Model** | **Accuracy** | **F Beta** | **Area under the ROC curve** | **Sensitivity** | **Specificity** | **Area under the PR curve** | **Brier** |
| --- | --- | --- | --- | --- | --- | --- | --- |
| AdaBoost | 0.578 (0.529-0.626) | 0.363 (0.298-0.428) | 0.632 (0.585-0.679) | 0.595 (0.459-0.732) | 0.574 (0.507-0.642) | 0.212 (0.149-0.276) | 0.233 (0.221-0.245) |
| DT | 0.612 (0.575-0.650) | 0.319 (0.244-0.394) | 0.596 (0.507-0.685) | 0.492 (0.361-0.623) | 0.629 (0.585-0.673) | 0.188 (0.138-0.237) | 0.242 (0.227-0.258) |
| Random Forest | 0.664 (0.636-0.693) | 0.293 (0.230-0.356) | 0.626 (0.561-0.691) | 0.410 (0.306-0.513) | 0.696 (0.665-0.727) | 0.212 (0.143-0.281) | 0.202 (0.193-0.210) |
| Light GBM | 0.647 (0.608-0.685) | 0.298 (0.241-0.355) | 0.601 (0.549-0.653) | 0.424 (0.336-0.513) | 0.675 (0.639-0.710) | 0.167 (0.130-0.205) | 0.230 (0.213-0.247) |
| KNN | 0.578 (0.537-0.618) | 0.362 (0.286-0.438) | 0.594 (0.530-0.659) | 0.580 (0.465-0.694) | 0.577 (0.534-0.619) | 0.158 (0.121-0.194) | 0.291 (0.266-0.315) |
| Naive Bayes | 0.786 (0.731-0.841) | 0.294 (0.199-0.388) | 0.668 (0.627-0.708) | 0.339 (0.220-0.457) | 0.841 (0.773-0.909) | 0.267 (0.187-0.347) | 0.176 (0.143-0.208) |
| SVM | 0.609 (0.570-0.647) | 0.298 (0.205-0.392) | 0.601 (0.542-0.660) | 0.454 (0.311-0.597) | 0.628 (0.587-0.670) | 0.159 (0.121-0.197) | 0.234 (0.225-0.244) |
| XGBoost | 0.663 (0.629-0.696) | 0.320 (0.219-0.422) | 0.602 (0.523-0.682) | 0.457 (0.291-0.623) | 0.689 (0.658-0.720) | 0.183 (0.125-0.242) | 0.270 (0.245-0.296) |
| CATBoost | 0.643 (0.608-0.678) | 0.355 (0.309-0.402) | 0.661 (0.613-0.709) | 0.526 (0.448-0.603) | 0.659 (0.617-0.701) | 0.252 (0.155-0.350) | 0.212 (0.197-0.227) |
| P | <.001^a^ | <.001^a^ | <.001^b^ | <.001^a^ | <.001^a^ | <.001^a^ | <.001^a^ |
| ^a^ANOVA test; ^b^Kruskal-Wallis | | |  |  |  |  |  |

**Supplementary Table 11.** Benchmarking analysis of machine learning models in the SHARE validation set.

| **Model** | **Accuracy** | **F Beta** | **Area under the ROC curve** | **Sensitivity** | **Specificity** | **Area under the PR curve** | **Brier** |
| --- | --- | --- | --- | --- | --- | --- | --- |
| AdaBoost | 0.555 (0.521-0.588) | 0.381 (0.309-0.450) | 0.654 (0.600-0.710) | 0.640 (0.538-0.750) | 0.544 (0.509-0.581) | 0.188 (0.130-0.252) | 0.215 (0.206-0.224) |
| DT | 0.501 (0.467-0.536) | 0.381 (0.314-0.447) | 0.611 (0.555-0.671) | 0.682 (0.583-0.793) | 0.479 (0.443-0.513) | 0.170 (0.116-0.224) | 0.236 (0.220-0.250) |
| Random Forest | 0.678 (0.648-0.712) | 0.333 (0.254-0.408) | 0.617 (0.558-0.674) | 0.460 (0.353-0.563) | 0.705 (0.674-0.740) | 0.161 (0.111-0.215) | 0.197 (0.188-0.207) |
| Light GBM | 0.661 (0.629-0.696) | 0.263 (0.183-0.335) | 0.566 (0.498-0.633) | 0.361 (0.250-0.467) | 0.698 (0.665-0.731) | 0.169 (0.106-0.239) | 0.222 (0.202-0.240) |
| KNN | 0.574 (0.541-0.610) | 0.198 (0.132-0.262) | 0.499 (0.433-0.562) | 0.300 (0.200-0.395) | 0.608 (0.571-0.643) | 0.113 (0.080-0.146) | 0.292 (0.271-0.313) |
| Naive Bayes | 0.791 (0.763-0.818) | 0.303 (0.212-0.381) | 0.674 (0.617-0.732) | 0.340 (0.241-0.438) | 0.846 (0.818-0.873) | 0.223 (0.151-0.302) | 0.162 (0.145-0.181) |
| SVM | 0.630 (0.596-0.662) | 0.324 (0.253-0.396) | 0.568 (0.510-0.634) | 0.479 (0.379-0.588) | 0.648 (0.612-0.682) | 0.130 (0.098-0.166) | 0.227 (0.218-0.238) |
| XGBoost | 0.674 (0.639-0.707) | 0.268 (0.185-0.340) | 0.580 (0.519-0.640) | 0.361 (0.258-0.467) | 0.712 (0.677-0.747) | 0.128 (0.095-0.162) | 0.255 (0.228-0.282) |
| CATBoost | 0.663 (0.629-0.698) | 0.326 (0.250-0.398) | 0.590 (0.527-0.655) | 0.459 (0.353-0.571) | 0.688 (0.653-0.723) | 0.136 (0.102-0.173) | 0.212 (0.196-0.228) |
| P | <.001^a^ | <.001^a^ | <.001^b^ | <.001^a^ | <.001^a^ | <.001^a^ | <.001^a^ |
| ^a^ANOVA test; ^b^Kruskal-Wallis | | |  |  |  |  |  |

**Supplementary Table 12.** Benchmarking analysis of machine learning models in the CHARLS training set.

| **Model** | **Accuracy** | **F Beta** | **Area under the ROC curve** | **Sensitivity** | **Specificity** | **Area under the PR curve** | **Brier** |
| --- | --- | --- | --- | --- | --- | --- | --- |
| AdaBoost | 0.594 (0.554-0.633) | 0.385 (0.324-0.446) | 0.626 (0.589-0.663) | 0.555 (0.489-0.622) | 0.598 (0.550-0.646) | 0.214 (0.176-0.252) | 0.231 (0.222-0.239) |
| DT | 0.585 (0.543-0.627) | 0.379 (0.335-0.422) | 0.609 (0.574-0.643) | 0.555 (0.490-0.621) | 0.589 (0.532-0.646) | 0.183 (0.151-0.215) | 0.239 (0.233-0.246) |
| Random Forest | 0.665 (0.647-0.684) | 0.346 (0.289-0.403) | 0.622 (0.582-0.661) | 0.448 (0.380-0.515) | 0.698 (0.676-0.720) | 0.213 (0.159-0.267) | 0.206 (0.199-0.214) |
| Light GBM | 0.659 (0.644-0.673) | 0.321 (0.266-0.375) | 0.578 (0.536-0.620) | 0.415 (0.347-0.484) | 0.696 (0.687-0.705) | 0.188 (0.146-0.229) | 0.228 (0.218-0.237) |
| KNN | 0.584 (0.557-0.610) | 0.305 (0.263-0.347) | 0.542 (0.494-0.591) | 0.428 (0.392-0.465) | 0.607 (0.579-0.634) | 0.162 (0.119-0.206) | 0.287 (0.268-0.306) |
| Naive Bayes | 0.690 (0.546-0.834) | 0.296 (0.234-0.359) | 0.650 (0.624-0.677) | 0.396 (0.212-0.580) | 0.739 (0.554-0.925) | 0.221 (0.191-0.250) | 0.251 (0.131-0.372) |
| SVM | 0.635 (0.600-0.671) | 0.376 (0.333-0.418) | 0.619 (0.587-0.651) | 0.522 (0.441-0.603) | 0.655 (0.609-0.701) | 0.203 (0.165-0.240) | 0.232 (0.225-0.238) |
| XGBoost | 0.659 (0.629-0.689) | 0.330 (0.281-0.378) | 0.586 (0.543-0.630) | 0.433 (0.349-0.517) | 0.696 (0.672-0.720) | 0.171 (0.151-0.191) | 0.276 (0.253-0.299) |
| CATBoost | 0.653 (0.632-0.675) | 0.342 (0.290-0.393) | 0.601 (0.569-0.633) | 0.448 (0.396-0.501) | 0.684 (0.660-0.708) | 0.198 (0.148-0.248) | 0.208 (0.199-0.218) |
| P | <.001^a^ | <.001^a^ | <.001^b^ | <.001^a^ | <.001^a^ | <.001^a^ | <.001^a^ |
| ^a^ANOVA test; ^b^Kruskal-Wallis | | |  |  |  |  |  |

**Supplementary Table 13.** Benchmarking analysis of machine learning models in the CHARLS validation set.

| **Model** | **Accuracy** | **F Beta** | **Area under the ROC curve** | **Sensitivity** | **Specificity** | **Area under the PR curve** | **Brier** |
| --- | --- | --- | --- | --- | --- | --- | --- |
| AdaBoost | 0.626 (0.606-0.647) | 0.390 (0.341-0.434) | 0.643 (0.606-0.683) | 0.587 (0.522-0.653) | 0.631 (0.609-0.653) | 0.186 (0.145-0.225) | 0.218 (0.211-0.224) |
| DT | 0.658 (0.637-0.679) | 0.318 (0.268-0.367) | 0.598 (0.562-0.637) | 0.443 (0.373-0.508) | 0.685 (0.664-0.709) | 0.147 (0.121-0.173) | 0.237 (0.229-0.244) |
| Random Forest | 0.690 (0.670-0.711) | 0.350 (0.295-0.399) | 0.639 (0.597-0.680) | 0.471 (0.400-0.538) | 0.718 (0.697-0.740) | 0.207 (0.156-0.258) | 0.198 (0.192-0.205) |
| Light GBM | 0.680 (0.657-0.702) | 0.308 (0.253-0.356) | 0.628 (0.584-0.665) | 0.413 (0.342-0.479) | 0.714 (0.689-0.736) | 0.198 (0.151-0.245) | 0.210 (0.199-0.222) |
| KNN | 0.600 (0.577-0.622) | 0.327 (0.279-0.373) | 0.583 (0.541-0.624) | 0.496 (0.429-0.562) | 0.613 (0.589-0.635) | 0.149 (0.122-0.181) | 0.276 (0.262-0.290) |
| Naive Bayes | 0.785 (0.766-0.804) | 0.293 (0.235-0.347) | 0.637 (0.595-0.681) | 0.327 (0.260-0.394) | 0.842 (0.825-0.860) | 0.186 (0.145-0.228) | 0.173 (0.158-0.189) |
| SVM | 0.636 (0.613-0.657) | 0.372 (0.319-0.418) | 0.639 (0.597-0.677) | 0.548 (0.480-0.615) | 0.647 (0.624-0.670) | 0.198 (0.152-0.241) | 0.228 (0.224-0.233) |
| XGBoost | 0.665 (0.644-0.686) | 0.286 (0.238-0.330) | 0.566 (0.525-0.610) | 0.388 (0.324-0.456) | 0.700 (0.678-0.722) | 0.153 (0.121-0.187) | 0.272 (0.256-0.289) |
| CATBoost | 0.705 (0.683-0.726) | 0.395 (0.341-0.444) | 0.651 (0.610-0.693) | 0.529 (0.457-0.600) | 0.727 (0.704-0.750) | 0.224 (0.172-0.279) | 0.196 (0.188-0.204) |
| P | <.001^a^ | <.001^a^ | <.001^b^ | <.001^a^ | <.001^a^ | <.001^a^ | <.001^a^ |
| ^a^ANOVA test; ^b^Kruskal-Wallis | | |  |  |  |  |  |

**Supplementary Table 14.** Ranking of 129 machine learning models in the HRS training set.

| **Model** | **Normalized Rank Score** | **Comprehensive Rank** | **Accuracy (95% CI)** | **F_Beta (95% CI)** | **ROC_AUC (95% CI)** | **Sensitivity (95% CI)** | **Specificity (95% CI)** | **PR_AUC (95% CI)** | **Brier (95% CI)** |
| --- | --- | --- | --- | --- | --- | --- | --- | --- | --- |
| AdaBoost+DT+RF+LightGBM+NaiveBayes+CatBoost | 3.571 | Rank 1 | 0.905 (0.895-0.915) | 0.883 (0.868-0.897) | 0.982 (0.978-0.986) | 1.000 (1.000-1.000) | 0.890 (0.878-0.901) | 0.875 (0.842-0.908) | 0.084 (0.079-0.089) |
| AdaBoost+RF+LightGBM+SVM+CatBoost | 5.286 | Rank 2 | 0.910 (0.901-0.920) | 0.882 (0.865-0.898) | 0.986 (0.983-0.989) | 0.988 (0.981-1.000) | 0.897 (0.887-0.908) | 0.902 (0.875-0.928) | 0.076 (0.071-0.081) |
| DT+RF+LightGBM | 6.000 | Rank 3 | 0.903 (0.892-0.913) | 0.878 (0.861-0.893) | 0.982 (0.978-0.986) | 0.996 (0.993-1.000) | 0.887 (0.875-0.899) | 0.885 (0.857-0.913) | 0.086 (0.082-0.091) |
| AdaBoost+DT+RF+LightGBM+SVM | 8.000 | Rank 4 | 0.897 (0.886-0.908) | 0.872 (0.856-0.888) | 0.983 (0.979-0.987) | 0.996 (0.993-1.000) | 0.881 (0.869-0.892) | 0.885 (0.855-0.914) | 0.083 (0.078-0.088) |
| DT+RF+LightGBM+NaiveBayes | 11.571 | Rank 5 | 0.892 (0.882-0.904) | 0.866 (0.850-0.883) | 0.985 (0.981-0.989) | 0.996 (0.993-1.000) | 0.875 (0.863-0.888) | 0.888 (0.856-0.923) | 0.085 (0.080-0.090) |
| AdaBoost+RF+LightGBM+NaiveBayes+SVM+CatBoost | 12.143 | Rank 6 | 0.896 (0.886-0.907) | 0.868 (0.850-0.885) | 0.979 (0.975-0.984) | 0.992 (0.987-1.000) | 0.880 (0.868-0.893) | 0.875 (0.847-0.901) | 0.085 (0.079-0.090) |
| AdaBoost+RF+LightGBM+NaiveBayes+CatBoost | 12.429 | Rank 7 | 0.898 (0.887-0.908) | 0.868 (0.851-0.886) | 0.979 (0.975-0.983) | 0.989 (0.981-1.000) | 0.882 (0.871-0.895) | 0.870 (0.841-0.897) | 0.085 (0.080-0.090) |
| AdaBoost+RF+LightGBM | 13.000 | Rank 8 | 0.889 (0.878-0.901) | 0.865 (0.848-0.881) | 0.984 (0.981-0.988) | 1.000 (1.000-1.000) | 0.870 (0.858-0.884) | 0.887 (0.857-0.915) | 0.088 (0.083-0.094) |
| AdaBoost+RF+LightGBM+NaiveBayes+SVM | 13.000 | Rank 8 | 0.894 (0.883-0.905) | 0.869 (0.852-0.885) | 0.981 (0.977-0.985) | 0.996 (0.993-1.000) | 0.877 (0.865-0.889) | 0.871 (0.843-0.898) | 0.088 (0.083-0.094) |
| DT+RF+LightGBM+SVM | 14.000 | Rank 10 | 0.896 (0.886-0.907) | 0.867 (0.849-0.883) | 0.981 (0.977-0.985) | 0.989 (0.981-1.000) | 0.881 (0.868-0.893) | 0.870 (0.838-0.905) | 0.088 (0.082-0.092) |
| AdaBoost+RF+LightGBM+SVM | 16.429 | Rank 11 | 0.895 (0.884-0.906) | 0.867 (0.849-0.883) | 0.978 (0.973-0.982) | 0.993 (0.987-1.000) | 0.878 (0.866-0.891) | 0.861 (0.831-0.890) | 0.089 (0.084-0.095) |
| RF+LightGBM+CatBoost | 16.429 | Rank 11 | 0.898 (0.887-0.908) | 0.866 (0.848-0.884) | 0.979 (0.975-0.983) | 0.985 (0.975-0.994) | 0.884 (0.871-0.896) | 0.864 (0.833-0.895) | 0.089 (0.084-0.094) |
| AdaBoost+RF+SVM+CatBoost | 16.571 | Rank 13 | 0.901 (0.890-0.911) | 0.867 (0.848-0.884) | 0.978 (0.973-0.982) | 0.981 (0.969-0.994) | 0.888 (0.876-0.900) | 0.851 (0.816-0.884) | 0.080 (0.075-0.086) |
| AdaBoost+DT+RF+LightGBM | 18.429 | Rank 14 | 0.890 (0.879-0.900) | 0.862 (0.845-0.879) | 0.980 (0.976-0.984) | 0.992 (0.987-1.000) | 0.873 (0.861-0.885) | 0.877 (0.851-0.904) | 0.090 (0.085-0.095) |
| AdaBoost+DT+RF+SVM | 18.714 | Rank 15 | 0.898 (0.887-0.908) | 0.859 (0.839-0.877) | 0.979 (0.974-0.984) | 0.973 (0.958-0.988) | 0.885 (0.873-0.897) | 0.881 (0.853-0.910) | 0.085 (0.080-0.090) |
| RF+LightGBM+NaiveBayes | 19.571 | Rank 16 | 0.888 (0.877-0.899) | 0.865 (0.848-0.879) | 0.977 (0.973-0.982) | 1.000 (1.000-1.000) | 0.870 (0.857-0.882) | 0.859 (0.826-0.889) | 0.091 (0.085-0.096) |
| RF+LightGBM+NaiveBayes+SVM+CatBoost | 22.429 | Rank 17 | 0.887 (0.876-0.898) | 0.861 (0.844-0.878) | 0.979 (0.975-0.983) | 0.996 (0.993-1.000) | 0.869 (0.856-0.882) | 0.853 (0.822-0.886) | 0.090 (0.085-0.095) |
| AdaBoost+RF+NaiveBayes+SVM | 24.714 | Rank 18 | 0.902 (0.892-0.912) | 0.862 (0.843-0.881) | 0.974 (0.969-0.979) | 0.969 (0.955-0.988) | 0.891 (0.880-0.902) | 0.847 (0.813-0.878) | 0.089 (0.085-0.094) |
| AdaBoost+DT+RF+LightGBM+CatBoost | 26.286 | Rank 19 | 0.885 (0.874-0.897) | 0.855 (0.837-0.872) | 0.979 (0.974-0.983) | 0.988 (0.981-1.000) | 0.868 (0.855-0.882) | 0.871 (0.843-0.899) | 0.093 (0.088-0.099) |
| DT+RF+LightGBM+CatBoost | 27.286 | Rank 20 | 0.895 (0.884-0.906) | 0.854 (0.834-0.872) | 0.976 (0.972-0.981) | 0.969 (0.954-0.987) | 0.883 (0.870-0.896) | 0.850 (0.817-0.885) | 0.088 (0.083-0.092) |
| RF+LightGBM+SVM+CatBoost | 28.429 | Rank 21 | 0.901 (0.890-0.911) | 0.861 (0.841-0.879) | 0.973 (0.968-0.978) | 0.969 (0.955-0.988) | 0.890 (0.877-0.901) | 0.821 (0.785-0.858) | 0.087 (0.082-0.092) |
| AdaBoost+RF+LightGBM+NaiveBayes | 29.571 | Rank 22 | 0.880 (0.868-0.892) | 0.852 (0.833-0.869) | 0.980 (0.976-0.984) | 0.993 (0.987-1.000) | 0.861 (0.847-0.875) | 0.864 (0.829-0.899) | 0.094 (0.088-0.100) |
| DT+RF+LightGBM+NaiveBayes+SVM | 30.000 | Rank 23 | 0.880 (0.869-0.891) | 0.856 (0.838-0.871) | 0.978 (0.974-0.982) | 1.000 (1.000-1.000) | 0.860 (0.847-0.872) | 0.853 (0.823-0.885) | 0.095 (0.090-0.101) |
| AdaBoost+RF+SVM | 30.714 | Rank 24 | 0.894 (0.883-0.906) | 0.851 (0.831-0.871) | 0.975 (0.970-0.980) | 0.965 (0.949-0.982) | 0.882 (0.870-0.895) | 0.860 (0.828-0.890) | 0.091 (0.086-0.096) |
| AdaBoost+DT+RF+LightGBM+SVM+CatBoost | 30.857 | Rank 25 | 0.884 (0.874-0.895) | 0.854 (0.835-0.872) | 0.977 (0.973-0.982) | 0.989 (0.981-1.000) | 0.867 (0.854-0.879) | 0.843 (0.807-0.880) | 0.093 (0.087-0.099) |
| AdaBoost+DT+LightGBM+SVM+CatBoost | 32.857 | Rank 26 | 0.898 (0.888-0.909) | 0.852 (0.832-0.872) | 0.972 (0.967-0.977) | 0.961 (0.944-0.981) | 0.888 (0.876-0.899) | 0.832 (0.798-0.866) | 0.090 (0.085-0.094) |
| AdaBoost+RF+NaiveBayes | 33.714 | Rank 27 | 0.891 (0.880-0.902) | 0.854 (0.835-0.872) | 0.974 (0.969-0.979) | 0.977 (0.964-0.993) | 0.877 (0.864-0.889) | 0.838 (0.802-0.872) | 0.094 (0.089-0.099) |
| AdaBoost+RF+LightGBM+CatBoost | 35.286 | Rank 28 | 0.881 (0.869-0.893) | 0.850 (0.831-0.867) | 0.976 (0.971-0.980) | 0.988 (0.981-1.000) | 0.863 (0.850-0.876) | 0.851 (0.820-0.883) | 0.094 (0.088-0.099) |
| AdaBoost+RF+NaiveBayes+CatBoost | 35.286 | Rank 28 | 0.882 (0.871-0.893) | 0.847 (0.828-0.864) | 0.976 (0.971-0.981) | 0.980 (0.969-0.994) | 0.866 (0.853-0.878) | 0.856 (0.826-0.888) | 0.091 (0.086-0.097) |
| RF+NaiveBayes+SVM+CatBoost | 35.571 | Rank 30 | 0.888 (0.876-0.900) | 0.844 (0.823-0.864) | 0.974 (0.970-0.979) | 0.965 (0.949-0.982) | 0.875 (0.862-0.888) | 0.855 (0.825-0.882) | 0.090 (0.085-0.095) |
| AdaBoost+DT+RF+LightGBM+NaiveBayes | 37.714 | Rank 31 | 0.875 (0.863-0.886) | 0.846 (0.827-0.862) | 0.980 (0.975-0.984) | 0.992 (0.987-1.000) | 0.855 (0.842-0.868) | 0.854 (0.819-0.889) | 0.093 (0.087-0.099) |
| LightGBM+NaiveBayes+CatBoost | 38.429 | Rank 32 | 0.886 (0.875-0.898) | 0.844 (0.825-0.864) | 0.975 (0.970-0.979) | 0.969 (0.954-0.983) | 0.872 (0.860-0.885) | 0.843 (0.811-0.873) | 0.091 (0.086-0.097) |
| RF+NaiveBayes+SVM | 39.143 | Rank 33 | 0.884 (0.873-0.895) | 0.845 (0.826-0.864) | 0.973 (0.968-0.979) | 0.973 (0.959-0.988) | 0.869 (0.856-0.882) | 0.856 (0.827-0.888) | 0.094 (0.089-0.099) |
| AdaBoost+DT+LightGBM+NaiveBayes | 39.857 | Rank 34 | 0.888 (0.878-0.900) | 0.864 (0.848-0.881) | 0.967 (0.961-0.972) | 1.000 (1.000-1.000) | 0.870 (0.858-0.883) | 0.754 (0.706-0.805) | 0.094 (0.089-0.100) |
| AdaBoost+LightGBM+NaiveBayes+SVM | 40.286 | Rank 35 | 0.882 (0.871-0.893) | 0.855 (0.837-0.873) | 0.973 (0.968-0.977) | 0.996 (0.993-1.000) | 0.863 (0.850-0.876) | 0.806 (0.766-0.844) | 0.098 (0.092-0.103) |
| AdaBoost+DT+RF+NaiveBayes | 40.571 | Rank 36 | 0.884 (0.873-0.896) | 0.849 (0.830-0.867) | 0.971 (0.966-0.977) | 0.981 (0.969-0.994) | 0.868 (0.855-0.882) | 0.836 (0.804-0.867) | 0.095 (0.090-0.100) |
| AdaBoost+DT+RF+SVM+CatBoost | 40.857 | Rank 37 | 0.881 (0.870-0.892) | 0.842 (0.823-0.861) | 0.976 (0.972-0.981) | 0.973 (0.959-0.988) | 0.866 (0.853-0.878) | 0.856 (0.826-0.885) | 0.095 (0.089-0.101) |
| AdaBoost+LightGBM+NaiveBayes+SVM+CatBoost | 41.286 | Rank 38 | 0.893 (0.883-0.903) | 0.861 (0.842-0.878) | 0.968 (0.963-0.973) | 0.985 (0.975-0.994) | 0.878 (0.866-0.890) | 0.779 (0.736-0.822) | 0.096 (0.091-0.101) |
| RF+LightGBM+NaiveBayes+CatBoost | 41.857 | Rank 39 | 0.885 (0.874-0.896) | 0.844 (0.824-0.863) | 0.972 (0.967-0.977) | 0.969 (0.955-0.988) | 0.871 (0.859-0.882) | 0.821 (0.782-0.857) | 0.090 (0.085-0.096) |
| AdaBoost+DT+LightGBM | 42.286 | Rank 40 | 0.891 (0.880-0.901) | 0.865 (0.849-0.881) | 0.968 (0.963-0.974) | 0.996 (0.993-1.000) | 0.874 (0.861-0.885) | 0.762 (0.720-0.804) | 0.101 (0.096-0.106) |
| AdaBoost+RF+CatBoost | 42.714 | Rank 41 | 0.883 (0.872-0.895) | 0.846 (0.827-0.865) | 0.972 (0.967-0.977) | 0.977 (0.963-0.988) | 0.868 (0.855-0.881) | 0.833 (0.798-0.870) | 0.096 (0.090-0.101) |
| LightGBM+NaiveBayes+SVM+CatBoost | 43.143 | Rank 42 | 0.885 (0.873-0.896) | 0.852 (0.833-0.870) | 0.971 (0.966-0.976) | 0.985 (0.975-0.994) | 0.868 (0.855-0.881) | 0.814 (0.775-0.853) | 0.099 (0.093-0.104) |
| AdaBoost+DT+RF+LightGBM+NaiveBayes+SVM+CatBoost | 43.286 | Rank 43 | 0.877 (0.866-0.888) | 0.844 (0.825-0.862) | 0.976 (0.971-0.981) | 0.984 (0.975-0.994) | 0.859 (0.846-0.872) | 0.852 (0.823-0.885) | 0.096 (0.090-0.101) |
| AdaBoost+LightGBM+NaiveBayes+CatBoost | 45.429 | Rank 44 | 0.895 (0.884-0.906) | 0.851 (0.832-0.871) | 0.964 (0.959-0.970) | 0.965 (0.949-0.982) | 0.883 (0.871-0.895) | 0.780 (0.743-0.816) | 0.090 (0.084-0.096) |
| AdaBoost+DT+RF+LightGBM+NaiveBayes+SVM | 47.000 | Rank 45 | 0.877 (0.866-0.889) | 0.842 (0.823-0.861) | 0.974 (0.969-0.979) | 0.981 (0.969-0.994) | 0.860 (0.847-0.873) | 0.818 (0.777-0.856) | 0.094 (0.088-0.100) |
| DT+LightGBM+CatBoost | 47.000 | Rank 45 | 0.874 (0.863-0.886) | 0.848 (0.831-0.866) | 0.974 (0.970-0.979) | 0.996 (0.993-1.000) | 0.854 (0.840-0.868) | 0.819 (0.779-0.856) | 0.097 (0.091-0.103) |
| DT+RF+NaiveBayes+SVM+CatBoost | 47.286 | Rank 47 | 0.880 (0.868-0.892) | 0.836 (0.814-0.856) | 0.973 (0.968-0.978) | 0.965 (0.949-0.982) | 0.866 (0.852-0.879) | 0.852 (0.821-0.882) | 0.094 (0.088-0.099) |
| RF+SVM+CatBoost | 50.143 | Rank 48 | 0.884 (0.872-0.896) | 0.838 (0.817-0.860) | 0.969 (0.964-0.975) | 0.962 (0.945-0.981) | 0.871 (0.858-0.883) | 0.822 (0.787-0.856) | 0.094 (0.089-0.099) |
| DT+LightGBM+NaiveBayes+CatBoost | 51.286 | Rank 49 | 0.875 (0.862-0.887) | 0.842 (0.821-0.860) | 0.972 (0.967-0.977) | 0.985 (0.975-0.994) | 0.856 (0.842-0.870) | 0.826 (0.793-0.861) | 0.096 (0.090-0.102) |
| DT+LightGBM | 51.857 | Rank 50 | 0.879 (0.868-0.891) | 0.856 (0.838-0.871) | 0.967 (0.962-0.973) | 1.000 (1.000-1.000) | 0.859 (0.846-0.873) | 0.729 (0.681-0.782) | 0.100 (0.095-0.106) |
| RF+LightGBM+NaiveBayes+SVM | 52.571 | Rank 51 | 0.875 (0.863-0.886) | 0.840 (0.819-0.858) | 0.973 (0.968-0.978) | 0.981 (0.969-0.994) | 0.857 (0.844-0.870) | 0.838 (0.801-0.877) | 0.098 (0.093-0.104) |
| RF+LightGBM | 53.429 | Rank 52 | 0.877 (0.866-0.889) | 0.840 (0.822-0.858) | 0.972 (0.968-0.978) | 0.977 (0.964-0.989) | 0.861 (0.848-0.873) | 0.838 (0.806-0.872) | 0.102 (0.097-0.107) |
| RF+SVM | 53.571 | Rank 53 | 0.891 (0.880-0.903) | 0.839 (0.818-0.859) | 0.965 (0.959-0.972) | 0.951 (0.931-0.970) | 0.881 (0.869-0.894) | 0.800 (0.763-0.837) | 0.096 (0.090-0.102) |
| AdaBoost+DT+RF | 54.286 | Rank 54 | 0.891 (0.880-0.901) | 0.838 (0.816-0.859) | 0.968 (0.962-0.973) | 0.949 (0.929-0.969) | 0.881 (0.869-0.893) | 0.820 (0.785-0.854) | 0.101 (0.096-0.105) |
| DT+RF+LightGBM+NaiveBayes+SVM+CatBoost | 55.286 | Rank 55 | 0.869 (0.857-0.880) | 0.845 (0.827-0.862) | 0.972 (0.967-0.977) | 1.000 (1.000-1.000) | 0.847 (0.834-0.861) | 0.810 (0.771-0.847) | 0.101 (0.093-0.107) |
| LightGBM+NaiveBayes | 55.286 | Rank 55 | 0.876 (0.864-0.888) | 0.852 (0.834-0.868) | 0.971 (0.966-0.976) | 1.000 (1.000-1.000) | 0.855 (0.842-0.869) | 0.777 (0.733-0.820) | 0.108 (0.103-0.113) |
| DT+RF+SVM | 56.714 | Rank 57 | 0.874 (0.863-0.887) | 0.841 (0.821-0.859) | 0.971 (0.965-0.976) | 0.984 (0.975-0.994) | 0.856 (0.844-0.870) | 0.836 (0.802-0.869) | 0.101 (0.096-0.107) |
| AdaBoost+DT+RF+NaiveBayes+SVM+CatBoost | 58.857 | Rank 58 | 0.872 (0.860-0.885) | 0.837 (0.816-0.856) | 0.973 (0.968-0.978) | 0.981 (0.969-0.994) | 0.854 (0.840-0.868) | 0.815 (0.772-0.856) | 0.100 (0.094-0.106) |
| DT+RF | 59.286 | Rank 59 | 0.877 (0.866-0.889) | 0.829 (0.807-0.851) | 0.970 (0.963-0.975) | 0.958 (0.940-0.976) | 0.864 (0.852-0.877) | 0.845 (0.815-0.875) | 0.101 (0.096-0.106) |
| AdaBoost+LightGBM+NaiveBayes | 59.857 | Rank 60 | 0.872 (0.860-0.883) | 0.846 (0.827-0.863) | 0.969 (0.964-0.975) | 0.996 (0.993-1.000) | 0.851 (0.838-0.864) | 0.758 (0.711-0.806) | 0.099 (0.093-0.104) |
| DT+RF+LightGBM+NaiveBayes+CatBoost | 60.143 | Rank 61 | 0.877 (0.865-0.888) | 0.827 (0.806-0.849) | 0.969 (0.964-0.974) | 0.954 (0.935-0.976) | 0.864 (0.851-0.877) | 0.818 (0.782-0.853) | 0.096 (0.090-0.101) |
| DT+LightGBM+SVM+CatBoost | 60.571 | Rank 62 | 0.879 (0.867-0.891) | 0.840 (0.819-0.859) | 0.966 (0.960-0.972) | 0.973 (0.959-0.988) | 0.864 (0.850-0.878) | 0.772 (0.727-0.814) | 0.098 (0.091-0.104) |
| DT+LightGBM+NaiveBayes+SVM+CatBoost | 61.000 | Rank 63 | 0.875 (0.864-0.887) | 0.838 (0.817-0.858) | 0.968 (0.962-0.973) | 0.977 (0.963-0.989) | 0.858 (0.845-0.871) | 0.795 (0.752-0.836) | 0.099 (0.092-0.105) |
| LightGBM+SVM | 61.000 | Rank 63 | 0.875 (0.864-0.887) | 0.844 (0.827-0.861) | 0.968 (0.963-0.973) | 0.988 (0.981-1.000) | 0.857 (0.844-0.869) | 0.801 (0.761-0.842) | 0.110 (0.105-0.115) |
| AdaBoost+DT+LightGBM+NaiveBayes+SVM+CatBoost | 62.286 | Rank 65 | 0.871 (0.860-0.883) | 0.840 (0.822-0.858) | 0.969 (0.963-0.974) | 0.988 (0.981-1.000) | 0.851 (0.839-0.865) | 0.797 (0.756-0.838) | 0.100 (0.094-0.106) |
| AdaBoost+LightGBM+CatBoost | 63.714 | Rank 66 | 0.871 (0.859-0.882) | 0.840 (0.820-0.858) | 0.968 (0.963-0.973) | 0.988 (0.981-1.000) | 0.851 (0.838-0.864) | 0.785 (0.742-0.827) | 0.098 (0.092-0.104) |
| DT+RF+LightGBM+SVM+CatBoost | 65.429 | Rank 67 | 0.873 (0.862-0.884) | 0.836 (0.816-0.855) | 0.967 (0.962-0.972) | 0.977 (0.964-0.994) | 0.856 (0.843-0.869) | 0.800 (0.761-0.835) | 0.099 (0.093-0.104) |
| RF+LightGBM+SVM | 66.143 | Rank 68 | 0.872 (0.860-0.884) | 0.842 (0.823-0.861) | 0.967 (0.962-0.973) | 0.989 (0.981-1.000) | 0.853 (0.840-0.866) | 0.785 (0.745-0.825) | 0.106 (0.100-0.112) |
| RF+NaiveBayes+CatBoost | 66.714 | Rank 69 | 0.879 (0.868-0.891) | 0.824 (0.801-0.846) | 0.964 (0.957-0.970) | 0.946 (0.926-0.969) | 0.868 (0.855-0.881) | 0.797 (0.759-0.836) | 0.097 (0.091-0.103) |
| DT+RF+NaiveBayes+CatBoost | 66.857 | Rank 70 | 0.876 (0.865-0.887) | 0.828 (0.807-0.849) | 0.967 (0.962-0.974) | 0.958 (0.940-0.980) | 0.863 (0.849-0.875) | 0.790 (0.749-0.834) | 0.097 (0.091-0.102) |
| AdaBoost+RF | 67.000 | Rank 71 | 0.870 (0.858-0.881) | 0.832 (0.813-0.851) | 0.971 (0.966-0.976) | 0.977 (0.963-0.993) | 0.852 (0.838-0.865) | 0.824 (0.790-0.857) | 0.105 (0.099-0.110) |
| AdaBoost+DT+LightGBM+NaiveBayes+CatBoost | 68.857 | Rank 72 | 0.865 (0.853-0.877) | 0.837 (0.817-0.856) | 0.968 (0.963-0.973) | 0.992 (0.987-1.000) | 0.844 (0.830-0.858) | 0.813 (0.777-0.848) | 0.103 (0.097-0.109) |
| DT+LightGBM+NaiveBayes+SVM | 70.000 | Rank 73 | 0.871 (0.859-0.882) | 0.845 (0.825-0.862) | 0.965 (0.959-0.970) | 0.996 (0.993-1.000) | 0.850 (0.837-0.863) | 0.750 (0.701-0.801) | 0.108 (0.103-0.114) |
| DT+RF+SVM+CatBoost | 70.000 | Rank 73 | 0.872 (0.860-0.884) | 0.833 (0.813-0.852) | 0.966 (0.960-0.972) | 0.973 (0.958-0.988) | 0.855 (0.842-0.868) | 0.791 (0.751-0.828) | 0.102 (0.096-0.108) |
| AdaBoost+DT+LightGBM+SVM | 70.286 | Rank 75 | 0.875 (0.864-0.886) | 0.842 (0.824-0.860) | 0.962 (0.955-0.968) | 0.985 (0.975-0.994) | 0.857 (0.844-0.869) | 0.744 (0.700-0.787) | 0.107 (0.101-0.112) |
| AdaBoost+DT+NaiveBayes+SVM+CatBoost | 70.429 | Rank 76 | 0.877 (0.865-0.888) | 0.824 (0.802-0.844) | 0.963 (0.956-0.969) | 0.951 (0.932-0.970) | 0.864 (0.851-0.877) | 0.801 (0.764-0.838) | 0.101 (0.095-0.106) |
| LightGBM+SVM+CatBoost | 71.571 | Rank 77 | 0.876 (0.865-0.888) | 0.828 (0.806-0.847) | 0.964 (0.959-0.970) | 0.958 (0.940-0.976) | 0.863 (0.849-0.876) | 0.797 (0.759-0.831) | 0.104 (0.099-0.109) |
| AdaBoost+DT+RF+CatBoost | 73.429 | Rank 78 | 0.865 (0.854-0.877) | 0.820 (0.799-0.839) | 0.971 (0.965-0.976) | 0.962 (0.945-0.981) | 0.849 (0.837-0.862) | 0.847 (0.816-0.877) | 0.102 (0.097-0.107) |
| LightGBM+CatBoost | 74.429 | Rank 79 | 0.868 (0.857-0.880) | 0.829 (0.807-0.847) | 0.966 (0.960-0.971) | 0.973 (0.959-0.988) | 0.851 (0.838-0.864) | 0.802 (0.767-0.838) | 0.102 (0.096-0.107) |
| AdaBoost+SVM+CatBoost | 76.000 | Rank 80 | 0.875 (0.864-0.886) | 0.818 (0.795-0.840) | 0.960 (0.953-0.967) | 0.942 (0.922-0.963) | 0.864 (0.851-0.876) | 0.790 (0.750-0.829) | 0.101 (0.095-0.106) |
| AdaBoost+DT+LightGBM+CatBoost | 77.143 | Rank 81 | 0.867 (0.856-0.879) | 0.836 (0.817-0.854) | 0.964 (0.958-0.970) | 0.988 (0.981-1.000) | 0.847 (0.833-0.860) | 0.763 (0.719-0.811) | 0.105 (0.099-0.112) |
| AdaBoost+LightGBM+SVM+CatBoost | 77.143 | Rank 81 | 0.867 (0.855-0.880) | 0.830 (0.809-0.850) | 0.966 (0.960-0.972) | 0.977 (0.963-0.994) | 0.849 (0.835-0.863) | 0.790 (0.748-0.832) | 0.103 (0.096-0.109) |
| DT+LightGBM+NaiveBayes | 77.429 | Rank 83 | 0.868 (0.856-0.879) | 0.844 (0.826-0.861) | 0.960 (0.954-0.967) | 1.000 (1.000-1.000) | 0.846 (0.833-0.859) | 0.723 (0.676-0.771) | 0.118 (0.114-0.123) |
| RF+NaiveBayes | 77.429 | Rank 83 | 0.874 (0.862-0.885) | 0.828 (0.807-0.848) | 0.962 (0.956-0.969) | 0.962 (0.946-0.981) | 0.859 (0.847-0.872) | 0.779 (0.742-0.818) | 0.104 (0.098-0.110) |
| DT+RF+NaiveBayes | 79.429 | Rank 85 | 0.870 (0.858-0.881) | 0.829 (0.808-0.848) | 0.962 (0.956-0.968) | 0.969 (0.955-0.987) | 0.854 (0.840-0.866) | 0.762 (0.719-0.805) | 0.102 (0.096-0.109) |
| LightGBM+NaiveBayes+SVM | 80.429 | Rank 86 | 0.869 (0.858-0.882) | 0.838 (0.819-0.857) | 0.960 (0.954-0.966) | 0.988 (0.981-1.000) | 0.849 (0.836-0.863) | 0.746 (0.701-0.791) | 0.110 (0.104-0.116) |
| DT+RF+CatBoost | 81.000 | Rank 87 | 0.868 (0.856-0.880) | 0.817 (0.797-0.838) | 0.962 (0.956-0.969) | 0.954 (0.935-0.975) | 0.853 (0.840-0.867) | 0.794 (0.752-0.834) | 0.100 (0.094-0.105) |
| DT+RF+NaiveBayes+SVM | 81.286 | Rank 88 | 0.870 (0.858-0.881) | 0.822 (0.800-0.842) | 0.963 (0.957-0.969) | 0.958 (0.940-0.976) | 0.855 (0.842-0.868) | 0.795 (0.756-0.834) | 0.107 (0.102-0.113) |
| AdaBoost+LightGBM+SVM | 82.000 | Rank 89 | 0.868 (0.856-0.880) | 0.833 (0.815-0.852) | 0.963 (0.957-0.969) | 0.981 (0.969-0.994) | 0.849 (0.835-0.864) | 0.762 (0.720-0.810) | 0.109 (0.103-0.115) |
| AdaBoost+DT+LightGBM+NaiveBayes+SVM | 82.857 | Rank 90 | 0.869 (0.857-0.881) | 0.829 (0.810-0.848) | 0.962 (0.956-0.968) | 0.973 (0.959-0.988) | 0.851 (0.838-0.865) | 0.755 (0.709-0.807) | 0.107 (0.101-0.112) |
| AdaBoost+DT+RF+NaiveBayes+CatBoost | 82.857 | Rank 90 | 0.867 (0.855-0.878) | 0.821 (0.801-0.841) | 0.963 (0.957-0.969) | 0.962 (0.945-0.978) | 0.851 (0.838-0.864) | 0.791 (0.753-0.828) | 0.103 (0.097-0.109) |
| AdaBoost+RF+NaiveBayes+SVM+CatBoost | 83.000 | Rank 92 | 0.868 (0.856-0.880) | 0.821 (0.798-0.841) | 0.959 (0.953-0.966) | 0.958 (0.940-0.976) | 0.853 (0.841-0.866) | 0.790 (0.753-0.825) | 0.102 (0.096-0.108) |
| AdaBoost+DT+CatBoost | 84.429 | Rank 93 | 0.873 (0.862-0.884) | 0.811 (0.789-0.834) | 0.957 (0.950-0.964) | 0.934 (0.912-0.957) | 0.862 (0.850-0.875) | 0.769 (0.729-0.809) | 0.104 (0.099-0.109) |
| AdaBoost+DT+RF+NaiveBayes+SVM | 85.000 | Rank 94 | 0.856 (0.844-0.868) | 0.819 (0.798-0.840) | 0.963 (0.957-0.970) | 0.977 (0.964-0.994) | 0.836 (0.823-0.850) | 0.801 (0.766-0.837) | 0.110 (0.104-0.116) |
| AdaBoost+LightGBM | 87.857 | Rank 95 | 0.865 (0.853-0.876) | 0.830 (0.808-0.849) | 0.961 (0.955-0.967) | 0.981 (0.969-0.994) | 0.845 (0.832-0.858) | 0.743 (0.699-0.791) | 0.114 (0.108-0.119) |
| AdaBoost+DT+SVM+CatBoost | 88.286 | Rank 96 | 0.868 (0.857-0.880) | 0.805 (0.783-0.828) | 0.956 (0.949-0.963) | 0.934 (0.913-0.958) | 0.856 (0.844-0.869) | 0.780 (0.743-0.815) | 0.103 (0.097-0.108) |
| NaiveBayes+SVM+CatBoost | 90.286 | Rank 97 | 0.848 (0.837-0.861) | 0.806 (0.784-0.826) | 0.963 (0.957-0.969) | 0.965 (0.950-0.982) | 0.829 (0.815-0.842) | 0.806 (0.770-0.844) | 0.110 (0.105-0.115) |
| RF+CatBoost | 92.429 | Rank 98 | 0.860 (0.848-0.873) | 0.806 (0.782-0.828) | 0.961 (0.954-0.968) | 0.946 (0.925-0.967) | 0.846 (0.833-0.860) | 0.788 (0.748-0.824) | 0.106 (0.101-0.112) |
| DT+LightGBM+SVM | 94.286 | Rank 99 | 0.857 (0.845-0.869) | 0.822 (0.802-0.841) | 0.953 (0.946-0.960) | 0.981 (0.969-0.994) | 0.836 (0.822-0.850) | 0.696 (0.647-0.746) | 0.114 (0.108-0.120) |
| DT+CatBoost | 94.857 | Rank 100 | 0.866 (0.854-0.878) | 0.791 (0.765-0.816) | 0.948 (0.940-0.957) | 0.911 (0.886-0.937) | 0.858 (0.845-0.872) | 0.759 (0.722-0.795) | 0.110 (0.105-0.116) |
| DT+SVM+CatBoost | 95.571 | Rank 101 | 0.861 (0.849-0.874) | 0.811 (0.787-0.835) | 0.957 (0.950-0.964) | 0.954 (0.935-0.974) | 0.846 (0.832-0.861) | 0.779 (0.740-0.814) | 0.111 (0.106-0.117) |
| AdaBoost+CatBoost | 97.571 | Rank 102 | 0.853 (0.841-0.865) | 0.805 (0.783-0.826) | 0.959 (0.952-0.966) | 0.958 (0.939-0.976) | 0.836 (0.821-0.849) | 0.770 (0.731-0.809) | 0.111 (0.105-0.116) |
| SVM+CatBoost | 98.429 | Rank 103 | 0.852 (0.840-0.864) | 0.811 (0.790-0.832) | 0.953 (0.945-0.960) | 0.969 (0.955-0.987) | 0.832 (0.819-0.846) | 0.736 (0.692-0.776) | 0.113 (0.107-0.119) |
| DT+NaiveBayes+SVM+CatBoost | 99.143 | Rank 104 | 0.862 (0.850-0.874) | 0.785 (0.758-0.809) | 0.948 (0.939-0.956) | 0.907 (0.881-0.933) | 0.854 (0.841-0.867) | 0.752 (0.711-0.792) | 0.113 (0.109-0.118) |
| AdaBoost+DT+NaiveBayes+CatBoost | 99.286 | Rank 105 | 0.855 (0.843-0.867) | 0.800 (0.776-0.823) | 0.954 (0.947-0.961) | 0.946 (0.926-0.968) | 0.840 (0.826-0.853) | 0.754 (0.713-0.794) | 0.108 (0.102-0.113) |
| DT+NaiveBayes+CatBoost | 100.571 | Rank 106 | 0.858 (0.845-0.872) | 0.781 (0.754-0.805) | 0.949 (0.940-0.957) | 0.907 (0.880-0.933) | 0.850 (0.836-0.864) | 0.763 (0.724-0.800) | 0.114 (0.109-0.119) |
| AdaBoost+NaiveBayes+SVM+CatBoost | 101.857 | Rank 107 | 0.855 (0.843-0.867) | 0.796 (0.770-0.818) | 0.952 (0.944-0.959) | 0.938 (0.917-0.960) | 0.841 (0.828-0.854) | 0.732 (0.684-0.780) | 0.108 (0.102-0.114) |
| AdaBoost+NaiveBayes+CatBoost | 106.000 | Rank 108 | 0.851 (0.838-0.863) | 0.794 (0.770-0.816) | 0.945 (0.936-0.952) | 0.942 (0.921-0.963) | 0.836 (0.823-0.850) | 0.720 (0.679-0.760) | 0.120 (0.114-0.126) |
| NaiveBayes+CatBoost | 107.429 | Rank 109 | 0.842 (0.829-0.855) | 0.784 (0.758-0.807) | 0.947 (0.939-0.956) | 0.938 (0.916-0.961) | 0.826 (0.812-0.840) | 0.714 (0.667-0.762) | 0.115 (0.109-0.121) |
| DT+NaiveBayes+SVM | 112.286 | Rank 110 | 0.762 (0.746-0.775) | 0.591 (0.558-0.624) | 0.824 (0.804-0.844) | 0.723 (0.683-0.765) | 0.768 (0.751-0.782) | 0.465 (0.414-0.514) | 0.170 (0.164-0.176) |
| AdaBoost+NaiveBayes+SVM | 113.429 | Rank 111 | 0.737 (0.721-0.752) | 0.584 (0.550-0.617) | 0.815 (0.794-0.837) | 0.738 (0.699-0.777) | 0.736 (0.720-0.753) | 0.472 (0.423-0.521) | 0.175 (0.168-0.181) |
| AdaBoost+DT+SVM | 113.714 | Rank 112 | 0.742 (0.727-0.757) | 0.597 (0.566-0.627) | 0.812 (0.790-0.834) | 0.754 (0.716-0.794) | 0.740 (0.724-0.756) | 0.442 (0.390-0.488) | 0.178 (0.172-0.184) |
| AdaBoost+DT+NaiveBayes+SVM | 114.000 | Rank 113 | 0.739 (0.725-0.755) | 0.582 (0.547-0.615) | 0.807 (0.785-0.829) | 0.732 (0.693-0.776) | 0.741 (0.724-0.758) | 0.508 (0.462-0.550) | 0.176 (0.169-0.181) |
| DT+SVM | 114.714 | Rank 114 | 0.729 (0.713-0.744) | 0.600 (0.569-0.633) | 0.817 (0.796-0.841) | 0.774 (0.737-0.812) | 0.722 (0.705-0.738) | 0.464 (0.415-0.517) | 0.185 (0.179-0.191) |
| AdaBoost+SVM | 115.286 | Rank 115 | 0.734 (0.719-0.749) | 0.585 (0.552-0.617) | 0.809 (0.787-0.830) | 0.743 (0.704-0.781) | 0.733 (0.716-0.749) | 0.454 (0.400-0.503) | 0.178 (0.171-0.185) |
| NaiveBayes+SVM | 115.571 | Rank 116 | 0.734 (0.718-0.748) | 0.581 (0.548-0.611) | 0.814 (0.793-0.835) | 0.736 (0.694-0.773) | 0.733 (0.717-0.750) | 0.466 (0.416-0.513) | 0.182 (0.175-0.187) |
| RF | 118.286 | Rank 117 | 0.753 (0.733-0.774) | 0.395 (0.362-0.428) | 0.681 (0.646-0.716) | 0.447 (0.399-0.496) | 0.805 (0.786-0.824) | 0.307 (0.274-0.341) | 0.178 (0.168-0.187) |
| AdaBoost+DT+NaiveBayes | 119.714 | Rank 118 | 0.678 (0.662-0.694) | 0.498 (0.463-0.530) | 0.739 (0.716-0.762) | 0.654 (0.611-0.700) | 0.682 (0.665-0.699) | 0.362 (0.318-0.406) | 0.202 (0.196-0.208) |
| CatBoost | 119.714 | Rank 118 | 0.735 (0.707-0.763) | 0.373 (0.336-0.411) | 0.666 (0.633-0.698) | 0.428 (0.381-0.476) | 0.786 (0.759-0.814) | 0.307 (0.255-0.360) | 0.177 (0.164-0.189) |
| AdaBoost+DT | 120.000 | Rank 120 | 0.698 (0.683-0.715) | 0.480 (0.443-0.516) | 0.722 (0.696-0.748) | 0.608 (0.562-0.655) | 0.714 (0.697-0.730) | 0.352 (0.306-0.394) | 0.203 (0.197-0.209) |
| DT+NaiveBayes | 121.571 | Rank 121 | 0.581 (0.564-0.598) | 0.500 (0.467-0.531) | 0.715 (0.689-0.744) | 0.743 (0.701-0.784) | 0.554 (0.536-0.572) | 0.321 (0.276-0.369) | 0.215 (0.210-0.220) |
| NaiveBayes | 122.000 | Rank 122 | 0.730 (0.703-0.757) | 0.379 (0.311-0.448) | 0.640 (0.601-0.679) | 0.443 (0.355-0.530) | 0.778 (0.753-0.804) | 0.267 (0.228-0.306) | 0.208 (0.192-0.223) |
| AdaBoost+NaiveBayes | 122.143 | Rank 123 | 0.668 (0.651-0.684) | 0.454 (0.417-0.488) | 0.707 (0.682-0.733) | 0.591 (0.544-0.635) | 0.681 (0.663-0.699) | 0.318 (0.276-0.361) | 0.205 (0.199-0.211) |
| LightGBM | 122.429 | Rank 124 | 0.726 (0.693-0.758) | 0.372 (0.314-0.430) | 0.654 (0.613-0.694) | 0.433 (0.368-0.498) | 0.774 (0.736-0.813) | 0.261 (0.229-0.294) | 0.194 (0.178-0.209) |
| AdaBoost | 123.714 | Rank 125 | 0.618 (0.576-0.660) | 0.462 (0.411-0.512) | 0.674 (0.640-0.708) | 0.650 (0.567-0.734) | 0.612 (0.556-0.668) | 0.294 (0.237-0.350) | 0.224 (0.211-0.237) |
| SVM | 124.143 | Rank 126 | 0.673 (0.652-0.693) | 0.409 (0.375-0.443) | 0.639 (0.602-0.676) | 0.522 (0.474-0.570) | 0.698 (0.677-0.719) | 0.256 (0.215-0.296) | 0.212 (0.201-0.223) |
| XGBoost | 125.286 | Rank 127 | 0.703 (0.675-0.732) | 0.346 (0.312-0.379) | 0.630 (0.593-0.667) | 0.410 (0.366-0.454) | 0.753 (0.727-0.779) | 0.248 (0.220-0.275) | 0.233 (0.213-0.252) |
| DT | 125.429 | Rank 128 | 0.668 (0.642-0.694) | 0.395 (0.357-0.433) | 0.622 (0.602-0.641) | 0.505 (0.450-0.560) | 0.696 (0.662-0.730) | 0.263 (0.224-0.303) | 0.230 (0.217-0.244) |
| KNN | 126.571 | Rank 129 | 0.623 (0.596-0.650) | 0.405 (0.367-0.444) | 0.612 (0.591-0.633) | 0.549 (0.501-0.598) | 0.635 (0.604-0.666) | 0.221 (0.193-0.249) | 0.273 (0.259-0.287) |

**Supplementary Table 15.** Ranking of 129 machine learning models in the HRS validation set.

| **Model** | **Normalized Rank Score** | **Comprehensive Rank** | **Accuracy (95% CI)** | **F_Beta (95% CI)** | **ROC_AUC (95% CI)** | **Sensitivity (95% CI)** | **Specificity (95% CI)** | **PR_AUC (95% CI)** | **Brier (95% CI)** |
| --- | --- | --- | --- | --- | --- | --- | --- | --- | --- |
| AdaBoost+RF+SVM | 15.714 | Rank 1 | 0.793 (0.771-0.815) | 0.376 (0.305-0.442) | 0.707 (0.659-0.755) | 0.442 (0.356-0.529) | 0.833 (0.813-0.853) | 0.282 (0.209-0.350) | 0.157 (0.145-0.167) |
| RF+SVM+CatBoost | 21.714 | Rank 2 | 0.782 (0.761-0.805) | 0.380 (0.311-0.441) | 0.704 (0.657-0.748) | 0.458 (0.373-0.542) | 0.820 (0.798-0.841) | 0.244 (0.177-0.306) | 0.161 (0.150-0.171) |
| RF+NaiveBayes+SVM | 23.429 | Rank 3 | 0.781 (0.760-0.802) | 0.396 (0.329-0.462) | 0.703 (0.659-0.747) | 0.481 (0.400-0.562) | 0.816 (0.795-0.838) | 0.231 (0.168-0.295) | 0.160 (0.150-0.170) |
| RF+CatBoost | 24.000 | Rank 4 | 0.764 (0.743-0.786) | 0.434 (0.367-0.491) | 0.739 (0.698-0.785) | 0.555 (0.472-0.640) | 0.788 (0.765-0.810) | 0.288 (0.214-0.361) | 0.164 (0.153-0.175) |
| RF+NaiveBayes | 26.571 | Rank 5 | 0.769 (0.746-0.789) | 0.398 (0.327-0.459) | 0.716 (0.672-0.759) | 0.495 (0.408-0.575) | 0.801 (0.779-0.822) | 0.253 (0.183-0.323) | 0.167 (0.156-0.178) |
| AdaBoost+DT+RF+LightGBM | 27.571 | Rank 6 | 0.776 (0.752-0.798) | 0.375 (0.307-0.440) | 0.694 (0.650-0.740) | 0.456 (0.375-0.542) | 0.813 (0.792-0.835) | 0.254 (0.187-0.318) | 0.163 (0.152-0.174) |
| RF+SVM | 29.714 | Rank 7 | 0.783 (0.762-0.803) | 0.354 (0.284-0.418) | 0.693 (0.644-0.742) | 0.422 (0.340-0.500) | 0.824 (0.804-0.845) | 0.262 (0.189-0.331) | 0.162 (0.152-0.173) |
| DT+RF+CatBoost | 29.857 | Rank 8 | 0.771 (0.747-0.794) | 0.373 (0.307-0.435) | 0.701 (0.658-0.747) | 0.458 (0.375-0.538) | 0.807 (0.784-0.831) | 0.241 (0.175-0.314) | 0.161 (0.150-0.172) |
| AdaBoost+SVM+CatBoost | 30.286 | Rank 9 | 0.777 (0.754-0.798) | 0.358 (0.290-0.425) | 0.697 (0.647-0.748) | 0.431 (0.340-0.519) | 0.817 (0.795-0.839) | 0.251 (0.178-0.325) | 0.160 (0.149-0.170) |
| DT+LightGBM+NaiveBayes+SVM | 31.000 | Rank 10 | 0.767 (0.744-0.788) | 0.388 (0.320-0.449) | 0.708 (0.659-0.754) | 0.482 (0.397-0.569) | 0.800 (0.777-0.822) | 0.268 (0.193-0.344) | 0.170 (0.159-0.181) |
| RF+LightGBM+SVM+CatBoost | 31.000 | Rank 10 | 0.785 (0.765-0.808) | 0.357 (0.290-0.424) | 0.697 (0.653-0.745) | 0.423 (0.340-0.509) | 0.827 (0.807-0.848) | 0.234 (0.171-0.296) | 0.156 (0.144-0.167) |
| AdaBoost+DT+LightGBM+SVM | 32.571 | Rank 12 | 0.767 (0.745-0.791) | 0.363 (0.297-0.427) | 0.694 (0.648-0.743) | 0.446 (0.364-0.528) | 0.804 (0.782-0.828) | 0.302 (0.224-0.375) | 0.166 (0.154-0.177) |
| DT+NaiveBayes+CatBoost | 34.143 | Rank 13 | 0.772 (0.750-0.795) | 0.348 (0.281-0.412) | 0.695 (0.649-0.742) | 0.421 (0.339-0.509) | 0.813 (0.791-0.836) | 0.284 (0.209-0.354) | 0.162 (0.153-0.172) |
| DT+RF+LightGBM | 35.429 | Rank 14 | 0.781 (0.759-0.804) | 0.343 (0.269-0.408) | 0.713 (0.670-0.758) | 0.408 (0.321-0.489) | 0.824 (0.804-0.847) | 0.235 (0.171-0.303) | 0.156 (0.145-0.166) |
| DT+LightGBM+NaiveBayes+SVM+CatBoost | 36.857 | Rank 15 | 0.769 (0.745-0.792) | 0.397 (0.331-0.459) | 0.693 (0.642-0.746) | 0.494 (0.414-0.577) | 0.801 (0.779-0.824) | 0.235 (0.178-0.295) | 0.167 (0.154-0.179) |
| AdaBoost+RF+CatBoost | 38.286 | Rank 16 | 0.763 (0.740-0.786) | 0.377 (0.305-0.441) | 0.694 (0.649-0.742) | 0.469 (0.380-0.553) | 0.797 (0.775-0.820) | 0.254 (0.184-0.323) | 0.168 (0.155-0.179) |
| AdaBoost+DT+RF+NaiveBayes+CatBoost | 38.571 | Rank 17 | 0.768 (0.745-0.790) | 0.378 (0.306-0.440) | 0.700 (0.652-0.747) | 0.467 (0.380-0.550) | 0.803 (0.779-0.825) | 0.238 (0.175-0.304) | 0.169 (0.158-0.181) |
| AdaBoost+DT+RF+SVM | 38.714 | Rank 18 | 0.778 (0.755-0.800) | 0.349 (0.280-0.414) | 0.698 (0.652-0.744) | 0.418 (0.333-0.500) | 0.820 (0.797-0.842) | 0.231 (0.172-0.294) | 0.159 (0.148-0.170) |
| AdaBoost+DT+CatBoost | 40.143 | Rank 19 | 0.767 (0.744-0.789) | 0.352 (0.281-0.417) | 0.683 (0.634-0.729) | 0.431 (0.341-0.509) | 0.805 (0.783-0.828) | 0.287 (0.207-0.360) | 0.162 (0.152-0.173) |
| AdaBoost+RF+NaiveBayes | 41.286 | Rank 20 | 0.771 (0.749-0.792) | 0.362 (0.292-0.425) | 0.692 (0.646-0.742) | 0.443 (0.360-0.531) | 0.808 (0.786-0.830) | 0.230 (0.165-0.291) | 0.163 (0.152-0.175) |
| RF+NaiveBayes+CatBoost | 42.000 | Rank 21 | 0.767 (0.744-0.789) | 0.361 (0.289-0.425) | 0.693 (0.645-0.738) | 0.444 (0.357-0.531) | 0.804 (0.782-0.827) | 0.238 (0.170-0.299) | 0.165 (0.154-0.178) |
| RF+LightGBM+NaiveBayes+CatBoost | 42.286 | Rank 22 | 0.773 (0.751-0.797) | 0.360 (0.287-0.423) | 0.702 (0.657-0.750) | 0.436 (0.353-0.511) | 0.813 (0.790-0.835) | 0.206 (0.153-0.258) | 0.165 (0.152-0.177) |
| AdaBoost+DT+RF+LightGBM+NaiveBayes+CatBoost | 44.714 | Rank 23 | 0.780 (0.757-0.803) | 0.344 (0.273-0.407) | 0.686 (0.639-0.734) | 0.410 (0.327-0.491) | 0.822 (0.801-0.845) | 0.229 (0.162-0.294) | 0.159 (0.149-0.171) |
| DT+RF+LightGBM+NaiveBayes+CatBoost | 45.571 | Rank 24 | 0.771 (0.749-0.794) | 0.338 (0.265-0.401) | 0.686 (0.633-0.733) | 0.408 (0.319-0.490) | 0.813 (0.792-0.837) | 0.278 (0.192-0.353) | 0.165 (0.153-0.177) |
| AdaBoost+DT+RF+LightGBM+SVM | 45.857 | Rank 25 | 0.780 (0.759-0.801) | 0.325 (0.253-0.387) | 0.694 (0.650-0.742) | 0.383 (0.298-0.467) | 0.826 (0.805-0.847) | 0.237 (0.176-0.307) | 0.158 (0.147-0.168) |
| AdaBoost+DT+NaiveBayes+SVM+CatBoost | 46.000 | Rank 26 | 0.765 (0.744-0.788) | 0.384 (0.317-0.443) | 0.674 (0.623-0.720) | 0.478 (0.396-0.558) | 0.799 (0.778-0.821) | 0.239 (0.169-0.308) | 0.166 (0.154-0.176) |
| AdaBoost+RF+NaiveBayes+CatBoost | 46.571 | Rank 27 | 0.763 (0.740-0.785) | 0.332 (0.268-0.395) | 0.707 (0.665-0.748) | 0.407 (0.327-0.490) | 0.804 (0.781-0.828) | 0.255 (0.177-0.327) | 0.161 (0.150-0.173) |
| DT+CatBoost | 46.571 | Rank 27 | 0.765 (0.743-0.787) | 0.387 (0.322-0.449) | 0.678 (0.631-0.730) | 0.482 (0.400-0.563) | 0.797 (0.775-0.821) | 0.234 (0.173-0.299) | 0.166 (0.156-0.176) |
| AdaBoost+DT+RF+SVM+CatBoost | 48.143 | Rank 29 | 0.772 (0.750-0.794) | 0.346 (0.279-0.412) | 0.696 (0.650-0.738) | 0.418 (0.333-0.500) | 0.813 (0.790-0.836) | 0.226 (0.164-0.288) | 0.165 (0.154-0.177) |
| RF | 48.143 | Rank 29 | 0.791 (0.771-0.813) | 0.339 (0.271-0.406) | 0.705 (0.662-0.755) | 0.395 (0.310-0.479) | 0.837 (0.816-0.857) | 0.228 (0.166-0.287) | 0.171 (0.163-0.178) |
| DT+LightGBM+CatBoost | 48.857 | Rank 31 | 0.760 (0.737-0.783) | 0.367 (0.301-0.427) | 0.686 (0.640-0.736) | 0.457 (0.375-0.532) | 0.796 (0.772-0.819) | 0.247 (0.180-0.325) | 0.170 (0.158-0.182) |
| DT+NaiveBayes+SVM+CatBoost | 48.857 | Rank 31 | 0.755 (0.734-0.778) | 0.363 (0.299-0.425) | 0.692 (0.646-0.738) | 0.455 (0.377-0.538) | 0.790 (0.767-0.812) | 0.241 (0.177-0.307) | 0.165 (0.156-0.174) |
| AdaBoost+RF+LightGBM+NaiveBayes+CatBoost | 49.286 | Rank 33 | 0.775 (0.751-0.798) | 0.320 (0.252-0.383) | 0.681 (0.637-0.725) | 0.380 (0.302-0.460) | 0.821 (0.799-0.844) | 0.275 (0.197-0.344) | 0.162 (0.151-0.174) |
| RF+LightGBM+CatBoost | 49.286 | Rank 33 | 0.768 (0.746-0.791) | 0.346 (0.278-0.410) | 0.679 (0.633-0.728) | 0.421 (0.340-0.510) | 0.808 (0.787-0.831) | 0.245 (0.173-0.320) | 0.164 (0.153-0.175) |
| DT+RF+LightGBM+CatBoost | 49.857 | Rank 35 | 0.768 (0.747-0.790) | 0.346 (0.273-0.408) | 0.688 (0.641-0.735) | 0.421 (0.333-0.500) | 0.809 (0.787-0.830) | 0.225 (0.164-0.293) | 0.161 (0.150-0.171) |
| AdaBoost+NaiveBayes+SVM+CatBoost | 50.286 | Rank 36 | 0.759 (0.737-0.781) | 0.358 (0.292-0.421) | 0.692 (0.645-0.736) | 0.445 (0.360-0.527) | 0.795 (0.774-0.817) | 0.229 (0.170-0.295) | 0.165 (0.153-0.177) |
| RF+NaiveBayes+SVM+CatBoost | 50.571 | Rank 37 | 0.772 (0.751-0.795) | 0.347 (0.284-0.411) | 0.682 (0.639-0.728) | 0.420 (0.340-0.500) | 0.813 (0.790-0.834) | 0.229 (0.160-0.290) | 0.165 (0.153-0.176) |
| AdaBoost+RF | 50.714 | Rank 38 | 0.762 (0.740-0.785) | 0.393 (0.322-0.456) | 0.690 (0.640-0.733) | 0.494 (0.408-0.571) | 0.793 (0.770-0.817) | 0.228 (0.169-0.288) | 0.174 (0.163-0.185) |
| AdaBoost+DT+LightGBM+NaiveBayes | 51.429 | Rank 39 | 0.759 (0.736-0.784) | 0.349 (0.280-0.412) | 0.682 (0.636-0.729) | 0.432 (0.348-0.519) | 0.797 (0.775-0.821) | 0.279 (0.203-0.360) | 0.168 (0.155-0.180) |
| DT+RF+SVM | 51.571 | Rank 40 | 0.765 (0.743-0.789) | 0.371 (0.303-0.436) | 0.682 (0.635-0.727) | 0.459 (0.375-0.538) | 0.801 (0.778-0.823) | 0.221 (0.163-0.281) | 0.169 (0.158-0.179) |
| RF+LightGBM+NaiveBayes+SVM+CatBoost | 52.571 | Rank 41 | 0.757 (0.734-0.778) | 0.373 (0.307-0.437) | 0.680 (0.635-0.728) | 0.469 (0.385-0.556) | 0.790 (0.766-0.813) | 0.247 (0.180-0.310) | 0.172 (0.161-0.183) |
| AdaBoost+DT+RF | 53.571 | Rank 42 | 0.752 (0.729-0.776) | 0.337 (0.274-0.399) | 0.701 (0.657-0.746) | 0.419 (0.340-0.500) | 0.791 (0.768-0.813) | 0.298 (0.218-0.375) | 0.168 (0.158-0.178) |
| AdaBoost+RF+LightGBM+NaiveBayes+SVM+CatBoost | 55.000 | Rank 43 | 0.769 (0.747-0.792) | 0.354 (0.287-0.418) | 0.674 (0.623-0.723) | 0.432 (0.347-0.520) | 0.808 (0.787-0.832) | 0.211 (0.154-0.268) | 0.163 (0.151-0.175) |
| AdaBoost+DT+NaiveBayes | 55.571 | Rank 44 | 0.681 (0.657-0.708) | 0.476 (0.419-0.532) | 0.736 (0.694-0.781) | 0.716 (0.643-0.796) | 0.677 (0.650-0.703) | 0.286 (0.210-0.358) | 0.204 (0.194-0.212) |
| AdaBoost+DT+RF+LightGBM+SVM+CatBoost | 55.714 | Rank 45 | 0.771 (0.747-0.792) | 0.338 (0.272-0.402) | 0.682 (0.635-0.728) | 0.409 (0.327-0.490) | 0.813 (0.790-0.835) | 0.235 (0.168-0.299) | 0.167 (0.155-0.179) |
| AdaBoost+LightGBM+NaiveBayes+CatBoost | 56.143 | Rank 46 | 0.779 (0.757-0.801) | 0.306 (0.234-0.370) | 0.670 (0.620-0.719) | 0.358 (0.269-0.438) | 0.828 (0.808-0.851) | 0.246 (0.172-0.329) | 0.159 (0.148-0.171) |
| DT+NaiveBayes+SVM | 56.143 | Rank 46 | 0.745 (0.721-0.767) | 0.424 (0.360-0.484) | 0.719 (0.675-0.763) | 0.557 (0.478-0.638) | 0.767 (0.742-0.791) | 0.237 (0.181-0.296) | 0.187 (0.177-0.195) |
| AdaBoost+NaiveBayes | 56.286 | Rank 48 | 0.679 (0.655-0.702) | 0.456 (0.397-0.507) | 0.729 (0.690-0.770) | 0.679 (0.604-0.756) | 0.678 (0.652-0.704) | 0.280 (0.201-0.347) | 0.202 (0.193-0.211) |
| NaiveBayes | 56.286 | Rank 48 | 0.761 (0.737-0.785) | 0.341 (0.272-0.406) | 0.694 (0.647-0.742) | 0.419 (0.333-0.508) | 0.801 (0.778-0.825) | 0.308 (0.222-0.387) | 0.184 (0.169-0.201) |
| AdaBoost+DT+RF+NaiveBayes+SVM+CatBoost | 56.857 | Rank 50 | 0.742 (0.719-0.765) | 0.347 (0.282-0.410) | 0.700 (0.654-0.746) | 0.443 (0.364-0.528) | 0.776 (0.755-0.799) | 0.289 (0.209-0.364) | 0.171 (0.160-0.182) |
| DT+RF | 56.857 | Rank 50 | 0.752 (0.729-0.776) | 0.353 (0.282-0.418) | 0.682 (0.633-0.729) | 0.444 (0.359-0.531) | 0.788 (0.765-0.811) | 0.268 (0.186-0.341) | 0.172 (0.161-0.183) |
| AdaBoost+RF+LightGBM+NaiveBayes+SVM | 57.429 | Rank 52 | 0.763 (0.740-0.786) | 0.334 (0.261-0.398) | 0.684 (0.637-0.732) | 0.407 (0.318-0.491) | 0.804 (0.780-0.828) | 0.245 (0.178-0.313) | 0.166 (0.154-0.177) |
| AdaBoost+DT+NaiveBayes+SVM | 57.571 | Rank 53 | 0.724 (0.701-0.747) | 0.395 (0.329-0.457) | 0.705 (0.659-0.750) | 0.531 (0.446-0.615) | 0.746 (0.723-0.770) | 0.272 (0.202-0.341) | 0.187 (0.177-0.196) |
| AdaBoost+DT | 57.714 | Rank 54 | 0.692 (0.668-0.718) | 0.413 (0.352-0.471) | 0.702 (0.656-0.750) | 0.591 (0.510-0.673) | 0.704 (0.679-0.732) | 0.301 (0.218-0.369) | 0.210 (0.199-0.220) |
| DT+NaiveBayes | 57.714 | Rank 54 | 0.559 (0.533-0.585) | 0.424 (0.366-0.473) | 0.725 (0.687-0.768) | 0.742 (0.667-0.821) | 0.538 (0.510-0.565) | 0.287 (0.211-0.358) | 0.215 (0.207-0.222) |
| DT+RF+LightGBM+SVM | 58.429 | Rank 56 | 0.765 (0.743-0.787) | 0.352 (0.284-0.413) | 0.680 (0.636-0.727) | 0.432 (0.347-0.511) | 0.803 (0.782-0.824) | 0.211 (0.159-0.271) | 0.166 (0.155-0.178) |
| DT+RF+NaiveBayes+CatBoost | 59.000 | Rank 57 | 0.770 (0.749-0.792) | 0.336 (0.275-0.398) | 0.676 (0.626-0.724) | 0.406 (0.327-0.480) | 0.812 (0.791-0.834) | 0.238 (0.170-0.306) | 0.166 (0.154-0.177) |
| AdaBoost+LightGBM+NaiveBayes+SVM+CatBoost | 60.000 | Rank 58 | 0.759 (0.736-0.784) | 0.340 (0.270-0.404) | 0.674 (0.624-0.725) | 0.420 (0.333-0.500) | 0.798 (0.775-0.822) | 0.266 (0.187-0.343) | 0.169 (0.156-0.180) |
| LightGBM+SVM+CatBoost | 60.000 | Rank 58 | 0.758 (0.736-0.782) | 0.348 (0.277-0.410) | 0.671 (0.622-0.719) | 0.432 (0.348-0.510) | 0.796 (0.773-0.818) | 0.243 (0.174-0.307) | 0.168 (0.157-0.179) |
| AdaBoost+DT+LightGBM+CatBoost | 60.143 | Rank 60 | 0.761 (0.739-0.783) | 0.390 (0.323-0.457) | 0.678 (0.631-0.723) | 0.492 (0.409-0.580) | 0.792 (0.770-0.814) | 0.225 (0.162-0.280) | 0.179 (0.167-0.190) |
| AdaBoost+RF+NaiveBayes+SVM+CatBoost | 61.429 | Rank 61 | 0.778 (0.756-0.800) | 0.342 (0.275-0.407) | 0.670 (0.622-0.718) | 0.409 (0.327-0.491) | 0.821 (0.800-0.843) | 0.212 (0.152-0.269) | 0.168 (0.156-0.180) |
| DT+SVM | 61.429 | Rank 61 | 0.683 (0.657-0.709) | 0.422 (0.360-0.478) | 0.722 (0.680-0.765) | 0.615 (0.529-0.698) | 0.691 (0.664-0.716) | 0.246 (0.180-0.316) | 0.204 (0.194-0.213) |
| DT+RF+LightGBM+NaiveBayes+SVM | 61.714 | Rank 63 | 0.756 (0.733-0.780) | 0.338 (0.268-0.399) | 0.683 (0.633-0.735) | 0.418 (0.333-0.500) | 0.796 (0.773-0.819) | 0.253 (0.178-0.327) | 0.170 (0.157-0.182) |
| DT+LightGBM | 62.000 | Rank 64 | 0.755 (0.730-0.778) | 0.339 (0.272-0.401) | 0.676 (0.626-0.728) | 0.422 (0.345-0.500) | 0.793 (0.769-0.817) | 0.290 (0.207-0.362) | 0.174 (0.162-0.185) |
| AdaBoost+DT+RF+LightGBM+NaiveBayes | 62.429 | Rank 65 | 0.754 (0.730-0.777) | 0.388 (0.321-0.452) | 0.680 (0.633-0.725) | 0.495 (0.412-0.577) | 0.784 (0.761-0.807) | 0.211 (0.156-0.266) | 0.174 (0.162-0.186) |
| DT+RF+LightGBM+SVM+CatBoost | 63.714 | Rank 66 | 0.753 (0.731-0.775) | 0.369 (0.300-0.431) | 0.680 (0.632-0.727) | 0.468 (0.380-0.550) | 0.785 (0.764-0.808) | 0.221 (0.159-0.290) | 0.172 (0.161-0.183) |
| NaiveBayes+SVM | 63.714 | Rank 66 | 0.694 (0.669-0.718) | 0.371 (0.304-0.425) | 0.700 (0.653-0.745) | 0.518 (0.440-0.600) | 0.714 (0.688-0.740) | 0.274 (0.198-0.344) | 0.200 (0.190-0.210) |
| DT | 64.714 | Rank 68 | 0.702 (0.678-0.725) | 0.382 (0.320-0.440) | 0.692 (0.641-0.740) | 0.531 (0.452-0.612) | 0.721 (0.697-0.747) | 0.286 (0.213-0.358) | 0.222 (0.211-0.233) |
| AdaBoost+RF+SVM+CatBoost | 64.857 | Rank 69 | 0.777 (0.756-0.798) | 0.313 (0.243-0.377) | 0.675 (0.631-0.723) | 0.369 (0.286-0.451) | 0.824 (0.801-0.847) | 0.204 (0.147-0.257) | 0.161 (0.148-0.173) |
| AdaBoost+DT+LightGBM+SVM+CatBoost | 65.571 | Rank 70 | 0.768 (0.746-0.791) | 0.301 (0.236-0.363) | 0.658 (0.611-0.705) | 0.359 (0.283-0.436) | 0.816 (0.793-0.838) | 0.244 (0.172-0.315) | 0.161 (0.150-0.171) |
| RF+LightGBM+NaiveBayes+SVM | 66.143 | Rank 71 | 0.762 (0.739-0.784) | 0.358 (0.292-0.421) | 0.679 (0.635-0.722) | 0.444 (0.362-0.528) | 0.798 (0.775-0.820) | 0.187 (0.141-0.240) | 0.173 (0.162-0.185) |
| AdaBoost+SVM | 66.571 | Rank 72 | 0.689 (0.664-0.715) | 0.412 (0.343-0.471) | 0.697 (0.648-0.746) | 0.593 (0.509-0.684) | 0.700 (0.674-0.726) | 0.237 (0.171-0.305) | 0.200 (0.188-0.210) |
| AdaBoost+RF+LightGBM+SVM+CatBoost | 67.143 | Rank 73 | 0.769 (0.748-0.791) | 0.283 (0.217-0.344) | 0.687 (0.643-0.731) | 0.335 (0.255-0.411) | 0.819 (0.797-0.841) | 0.201 (0.152-0.253) | 0.156 (0.145-0.167) |
| AdaBoost+DT+SVM | 67.857 | Rank 74 | 0.708 (0.685-0.733) | 0.430 (0.371-0.488) | 0.707 (0.663-0.750) | 0.605 (0.528-0.681) | 0.720 (0.695-0.745) | 0.211 (0.162-0.266) | 0.199 (0.189-0.208) |
| AdaBoost+DT+RF+NaiveBayes | 68.286 | Rank 75 | 0.767 (0.743-0.791) | 0.318 (0.244-0.381) | 0.678 (0.635-0.726) | 0.383 (0.294-0.468) | 0.811 (0.789-0.834) | 0.226 (0.157-0.285) | 0.166 (0.155-0.177) |
| AdaBoost+DT+NaiveBayes+CatBoost | 68.429 | Rank 76 | 0.740 (0.716-0.765) | 0.347 (0.283-0.404) | 0.683 (0.637-0.731) | 0.444 (0.360-0.526) | 0.774 (0.749-0.799) | 0.253 (0.184-0.316) | 0.174 (0.163-0.185) |
| DT+SVM+CatBoost | 69.286 | Rank 77 | 0.746 (0.722-0.769) | 0.341 (0.270-0.404) | 0.678 (0.630-0.727) | 0.432 (0.345-0.511) | 0.782 (0.758-0.806) | 0.252 (0.177-0.330) | 0.173 (0.162-0.183) |
| LightGBM+CatBoost | 69.286 | Rank 77 | 0.747 (0.724-0.771) | 0.353 (0.283-0.413) | 0.675 (0.628-0.724) | 0.447 (0.362-0.533) | 0.781 (0.756-0.806) | 0.240 (0.171-0.308) | 0.174 (0.162-0.187) |
| DT+LightGBM+NaiveBayes+CatBoost | 69.714 | Rank 79 | 0.756 (0.734-0.779) | 0.364 (0.299-0.423) | 0.654 (0.605-0.705) | 0.457 (0.377-0.531) | 0.791 (0.768-0.814) | 0.208 (0.151-0.266) | 0.170 (0.159-0.182) |
| AdaBoost+RF+NaiveBayes+SVM | 70.000 | Rank 80 | 0.774 (0.752-0.796) | 0.321 (0.252-0.390) | 0.674 (0.631-0.724) | 0.381 (0.296-0.464) | 0.820 (0.799-0.842) | 0.186 (0.142-0.237) | 0.166 (0.154-0.177) |
| AdaBoost+LightGBM+CatBoost | 70.571 | Rank 81 | 0.756 (0.733-0.781) | 0.338 (0.270-0.398) | 0.660 (0.613-0.709) | 0.419 (0.340-0.500) | 0.796 (0.774-0.819) | 0.243 (0.169-0.312) | 0.170 (0.157-0.182) |
| AdaBoost+LightGBM | 71.000 | Rank 82 | 0.735 (0.711-0.758) | 0.344 (0.281-0.406) | 0.695 (0.651-0.740) | 0.443 (0.364-0.531) | 0.768 (0.744-0.793) | 0.262 (0.187-0.336) | 0.183 (0.172-0.193) |
| AdaBoost | 71.143 | Rank 83 | 0.584 (0.559-0.610) | 0.454 (0.401-0.500) | 0.703 (0.660-0.743) | 0.779 (0.712-0.851) | 0.562 (0.534-0.588) | 0.221 (0.162-0.282) | 0.241 (0.232-0.250) |
| AdaBoost+LightGBM+SVM | 72.000 | Rank 84 | 0.744 (0.720-0.769) | 0.348 (0.279-0.409) | 0.673 (0.622-0.718) | 0.443 (0.356-0.526) | 0.779 (0.754-0.803) | 0.268 (0.190-0.335) | 0.180 (0.168-0.191) |
| LightGBM+NaiveBayes+SVM+CatBoost | 72.286 | Rank 85 | 0.747 (0.725-0.770) | 0.341 (0.276-0.401) | 0.668 (0.620-0.716) | 0.431 (0.354-0.512) | 0.784 (0.760-0.807) | 0.252 (0.176-0.321) | 0.173 (0.161-0.184) |
| AdaBoost+DT+LightGBM | 73.000 | Rank 86 | 0.753 (0.730-0.775) | 0.327 (0.256-0.388) | 0.689 (0.646-0.738) | 0.407 (0.321-0.500) | 0.793 (0.770-0.815) | 0.222 (0.161-0.286) | 0.168 (0.157-0.178) |
| AdaBoost+NaiveBayes+CatBoost | 73.000 | Rank 86 | 0.749 (0.726-0.772) | 0.334 (0.269-0.395) | 0.691 (0.645-0.736) | 0.418 (0.339-0.500) | 0.787 (0.764-0.810) | 0.238 (0.173-0.309) | 0.174 (0.163-0.185) |
| DT+RF+NaiveBayes+SVM | 73.571 | Rank 88 | 0.752 (0.730-0.775) | 0.352 (0.281-0.417) | 0.688 (0.643-0.736) | 0.442 (0.353-0.528) | 0.788 (0.765-0.811) | 0.198 (0.146-0.247) | 0.177 (0.166-0.189) |
| AdaBoost+RF+LightGBM+CatBoost | 73.714 | Rank 89 | 0.753 (0.729-0.776) | 0.311 (0.246-0.373) | 0.674 (0.628-0.722) | 0.383 (0.302-0.462) | 0.796 (0.774-0.817) | 0.249 (0.177-0.316) | 0.168 (0.156-0.179) |
| AdaBoost+DT+RF+CatBoost | 74.143 | Rank 90 | 0.748 (0.726-0.771) | 0.384 (0.316-0.446) | 0.671 (0.625-0.716) | 0.494 (0.408-0.576) | 0.778 (0.755-0.802) | 0.197 (0.148-0.253) | 0.177 (0.166-0.188) |
| AdaBoost+RF+LightGBM+SVM | 75.286 | Rank 91 | 0.775 (0.752-0.797) | 0.304 (0.237-0.369) | 0.666 (0.620-0.713) | 0.359 (0.283-0.442) | 0.823 (0.802-0.844) | 0.200 (0.144-0.255) | 0.168 (0.156-0.180) |
| NaiveBayes+CatBoost | 75.429 | Rank 92 | 0.744 (0.720-0.767) | 0.333 (0.270-0.398) | 0.677 (0.630-0.726) | 0.421 (0.340-0.500) | 0.781 (0.757-0.806) | 0.261 (0.185-0.329) | 0.174 (0.163-0.185) |
| DT+RF+LightGBM+NaiveBayes+SVM+CatBoost | 76.286 | Rank 93 | 0.736 (0.713-0.760) | 0.385 (0.317-0.446) | 0.688 (0.643-0.735) | 0.505 (0.420-0.588) | 0.763 (0.740-0.788) | 0.204 (0.156-0.253) | 0.188 (0.175-0.202) |
| AdaBoost+LightGBM+SVM+CatBoost | 76.857 | Rank 94 | 0.752 (0.728-0.774) | 0.335 (0.266-0.398) | 0.660 (0.608-0.715) | 0.419 (0.333-0.500) | 0.790 (0.769-0.813) | 0.253 (0.179-0.328) | 0.175 (0.163-0.187) |
| AdaBoost+NaiveBayes+SVM | 77.000 | Rank 95 | 0.691 (0.666-0.715) | 0.370 (0.305-0.429) | 0.692 (0.645-0.740) | 0.518 (0.431-0.604) | 0.712 (0.685-0.738) | 0.221 (0.164-0.284) | 0.194 (0.185-0.204) |
| SVM+CatBoost | 77.000 | Rank 95 | 0.739 (0.716-0.764) | 0.329 (0.260-0.394) | 0.695 (0.649-0.741) | 0.419 (0.333-0.509) | 0.776 (0.753-0.799) | 0.238 (0.174-0.305) | 0.174 (0.163-0.186) |
| SVM | 81.429 | Rank 97 | 0.677 (0.653-0.703) | 0.372 (0.309-0.434) | 0.680 (0.631-0.727) | 0.534 (0.449-0.625) | 0.694 (0.667-0.721) | 0.222 (0.166-0.288) | 0.201 (0.191-0.211) |
| AdaBoost+CatBoost | 81.571 | Rank 98 | 0.728 (0.705-0.752) | 0.349 (0.283-0.409) | 0.655 (0.605-0.706) | 0.457 (0.370-0.542) | 0.759 (0.735-0.784) | 0.251 (0.180-0.322) | 0.181 (0.170-0.192) |
| AdaBoost+DT+RF+NaiveBayes+SVM | 81.714 | Rank 99 | 0.727 (0.702-0.750) | 0.341 (0.276-0.405) | 0.692 (0.650-0.741) | 0.445 (0.365-0.536) | 0.759 (0.736-0.783) | 0.223 (0.165-0.285) | 0.180 (0.169-0.192) |
| LightGBM | 82.714 | Rank 100 | 0.745 (0.722-0.768) | 0.334 (0.269-0.396) | 0.670 (0.623-0.720) | 0.421 (0.340-0.500) | 0.783 (0.760-0.805) | 0.245 (0.174-0.309) | 0.183 (0.169-0.196) |
| RF+LightGBM+SVM | 83.000 | Rank 101 | 0.748 (0.724-0.770) | 0.327 (0.260-0.387) | 0.690 (0.647-0.736) | 0.408 (0.326-0.489) | 0.787 (0.764-0.810) | 0.214 (0.164-0.273) | 0.177 (0.166-0.188) |
| AdaBoost+RF+LightGBM | 83.143 | Rank 102 | 0.760 (0.738-0.783) | 0.287 (0.221-0.350) | 0.658 (0.610-0.706) | 0.345 (0.265-0.422) | 0.809 (0.786-0.831) | 0.226 (0.163-0.286) | 0.169 (0.158-0.181) |
| DT+RF+NaiveBayes+SVM+CatBoost | 83.857 | Rank 103 | 0.755 (0.732-0.776) | 0.286 (0.221-0.344) | 0.659 (0.612-0.704) | 0.346 (0.269-0.420) | 0.802 (0.781-0.823) | 0.215 (0.146-0.281) | 0.164 (0.154-0.174) |
| DT+RF+SVM+CatBoost | 84.857 | Rank 104 | 0.742 (0.718-0.766) | 0.332 (0.268-0.394) | 0.664 (0.614-0.715) | 0.420 (0.339-0.500) | 0.780 (0.757-0.803) | 0.240 (0.168-0.304) | 0.175 (0.163-0.187) |
| AdaBoost+DT+RF+LightGBM+NaiveBayes+SVM | 85.429 | Rank 105 | 0.741 (0.717-0.764) | 0.340 (0.273-0.401) | 0.689 (0.646-0.729) | 0.433 (0.346-0.513) | 0.777 (0.755-0.801) | 0.177 (0.138-0.217) | 0.178 (0.166-0.190) |
| RF+LightGBM | 88.857 | Rank 106 | 0.744 (0.722-0.767) | 0.280 (0.216-0.344) | 0.679 (0.635-0.724) | 0.345 (0.264-0.424) | 0.791 (0.768-0.812) | 0.240 (0.174-0.307) | 0.174 (0.163-0.185) |
| LightGBM+NaiveBayes+CatBoost | 89.286 | Rank 107 | 0.766 (0.742-0.789) | 0.283 (0.213-0.341) | 0.636 (0.589-0.683) | 0.335 (0.254-0.408) | 0.816 (0.794-0.838) | 0.191 (0.135-0.240) | 0.171 (0.158-0.183) |
| DT+LightGBM+SVM | 89.714 | Rank 108 | 0.734 (0.711-0.756) | 0.336 (0.271-0.397) | 0.672 (0.625-0.717) | 0.433 (0.352-0.512) | 0.768 (0.746-0.793) | 0.233 (0.163-0.297) | 0.182 (0.171-0.193) |
| DT+RF+LightGBM+NaiveBayes | 92.429 | Rank 109 | 0.751 (0.729-0.774) | 0.328 (0.260-0.389) | 0.644 (0.597-0.693) | 0.407 (0.327-0.489) | 0.791 (0.770-0.816) | 0.183 (0.137-0.235) | 0.172 (0.160-0.183) |
| LightGBM+SVM | 92.429 | Rank 109 | 0.753 (0.729-0.775) | 0.317 (0.253-0.380) | 0.658 (0.615-0.707) | 0.392 (0.312-0.477) | 0.794 (0.770-0.816) | 0.208 (0.155-0.271) | 0.178 (0.167-0.189) |
| AdaBoost+DT+RF+LightGBM+CatBoost | 95.000 | Rank 111 | 0.738 (0.715-0.761) | 0.327 (0.262-0.387) | 0.652 (0.604-0.702) | 0.417 (0.333-0.500) | 0.775 (0.752-0.798) | 0.222 (0.158-0.289) | 0.174 (0.162-0.186) |
| AdaBoost+LightGBM+NaiveBayes | 95.143 | Rank 112 | 0.744 (0.723-0.766) | 0.307 (0.240-0.370) | 0.661 (0.611-0.707) | 0.383 (0.300-0.462) | 0.785 (0.764-0.808) | 0.221 (0.153-0.281) | 0.174 (0.163-0.185) |
| CatBoost | 96.714 | Rank 113 | 0.745 (0.723-0.768) | 0.307 (0.240-0.371) | 0.669 (0.624-0.715) | 0.384 (0.302-0.463) | 0.787 (0.766-0.808) | 0.205 (0.151-0.266) | 0.175 (0.165-0.185) |
| KNN | 97.571 | Rank 114 | 0.619 (0.594-0.646) | 0.355 (0.297-0.408) | 0.648 (0.596-0.704) | 0.553 (0.473-0.640) | 0.627 (0.600-0.652) | 0.197 (0.149-0.245) | 0.273 (0.255-0.289) |
| NaiveBayes+SVM+CatBoost | 98.429 | Rank 115 | 0.740 (0.719-0.766) | 0.321 (0.257-0.381) | 0.654 (0.607-0.705) | 0.405 (0.327-0.490) | 0.779 (0.756-0.804) | 0.231 (0.166-0.296) | 0.182 (0.171-0.193) |
| RF+LightGBM+NaiveBayes | 98.571 | Rank 116 | 0.748 (0.725-0.772) | 0.299 (0.230-0.361) | 0.658 (0.609-0.709) | 0.369 (0.291-0.449) | 0.791 (0.769-0.814) | 0.211 (0.147-0.270) | 0.178 (0.166-0.190) |
| AdaBoost+DT+SVM+CatBoost | 98.857 | Rank 117 | 0.737 (0.714-0.762) | 0.294 (0.226-0.357) | 0.651 (0.599-0.699) | 0.369 (0.283-0.453) | 0.780 (0.756-0.806) | 0.236 (0.167-0.305) | 0.174 (0.163-0.186) |
| DT+RF+NaiveBayes | 98.857 | Rank 117 | 0.742 (0.719-0.765) | 0.305 (0.243-0.367) | 0.686 (0.645-0.726) | 0.382 (0.304-0.462) | 0.784 (0.761-0.808) | 0.183 (0.139-0.230) | 0.182 (0.170-0.194) |
| DT+LightGBM+SVM+CatBoost | 99.143 | Rank 119 | 0.746 (0.722-0.770) | 0.309 (0.243-0.375) | 0.656 (0.613-0.705) | 0.385 (0.304-0.471) | 0.788 (0.763-0.812) | 0.204 (0.147-0.255) | 0.178 (0.165-0.189) |
| AdaBoost+DT+LightGBM+NaiveBayes+SVM+CatBoost | 100.143 | Rank 120 | 0.740 (0.717-0.765) | 0.305 (0.236-0.365) | 0.665 (0.618-0.716) | 0.383 (0.300-0.460) | 0.782 (0.758-0.805) | 0.212 (0.152-0.281) | 0.176 (0.163-0.189) |
| AdaBoost+RF+LightGBM+NaiveBayes | 100.429 | Rank 121 | 0.743 (0.720-0.767) | 0.296 (0.227-0.359) | 0.670 (0.620-0.718) | 0.368 (0.286-0.449) | 0.787 (0.764-0.809) | 0.205 (0.149-0.274) | 0.175 (0.162-0.187) |
| DT+LightGBM+NaiveBayes | 101.143 | Rank 122 | 0.740 (0.718-0.763) | 0.314 (0.247-0.376) | 0.657 (0.609-0.703) | 0.395 (0.314-0.476) | 0.780 (0.757-0.804) | 0.220 (0.160-0.290) | 0.181 (0.170-0.191) |
| AdaBoost+DT+LightGBM+NaiveBayes+CatBoost | 102.000 | Rank 123 | 0.732 (0.709-0.755) | 0.311 (0.242-0.374) | 0.649 (0.601-0.701) | 0.396 (0.306-0.479) | 0.770 (0.747-0.795) | 0.233 (0.167-0.300) | 0.178 (0.167-0.190) |
| AdaBoost+LightGBM+NaiveBayes+SVM | 104.000 | Rank 124 | 0.736 (0.713-0.760) | 0.311 (0.244-0.373) | 0.650 (0.601-0.698) | 0.395 (0.311-0.473) | 0.775 (0.752-0.800) | 0.218 (0.153-0.281) | 0.178 (0.166-0.189) |
| LightGBM+NaiveBayes+SVM | 105.000 | Rank 125 | 0.737 (0.714-0.760) | 0.319 (0.257-0.378) | 0.659 (0.613-0.706) | 0.406 (0.327-0.482) | 0.775 (0.754-0.798) | 0.195 (0.146-0.249) | 0.182 (0.170-0.193) |
| AdaBoost+DT+LightGBM+NaiveBayes+SVM | 113.000 | Rank 126 | 0.733 (0.710-0.756) | 0.294 (0.230-0.350) | 0.650 (0.600-0.697) | 0.371 (0.294-0.452) | 0.775 (0.752-0.799) | 0.193 (0.144-0.249) | 0.180 (0.168-0.191) |
| AdaBoost+DT+RF+LightGBM+NaiveBayes+SVM+CatBoost | 116.429 | Rank 127 | 0.726 (0.702-0.751) | 0.289 (0.223-0.349) | 0.633 (0.590-0.682) | 0.370 (0.289-0.445) | 0.767 (0.744-0.792) | 0.175 (0.129-0.226) | 0.180 (0.167-0.192) |
| LightGBM+NaiveBayes | 120.143 | Rank 128 | 0.719 (0.694-0.743) | 0.278 (0.215-0.341) | 0.638 (0.590-0.683) | 0.357 (0.278-0.436) | 0.762 (0.737-0.785) | 0.187 (0.132-0.250) | 0.190 (0.178-0.201) |
| XGBoost | 125.000 | Rank 129 | 0.705 (0.683-0.730) | 0.229 (0.162-0.288) | 0.598 (0.550-0.646) | 0.295 (0.210-0.370) | 0.753 (0.729-0.778) | 0.137 (0.107-0.170) | 0.232 (0.213-0.250) |

**Supplementary Table 16.** Ranking of 129 machine learning models in the ELSA training set.

| **Model** | **Normalized Rank Score** | **Comprehensive Rank** | **Accuracy (95% CI)** | **F_Beta (95% CI)** | **ROC_AUC (95% CI)** | **Sensitivity (95% CI)** | **Specificity (95% CI)** | **PR_AUC (95% CI)** | **Brier (95% CI)** |
| --- | --- | --- | --- | --- | --- | --- | --- | --- | --- |
| AdaBoost+DT+RF+SVM+XGBoost+CatBoost | 3.429 | Rank 1 | 0.835 (0.822-0.847) | 0.730 (0.703-0.755) | 0.958 (0.952-0.965) | 0.990 (0.982-1.000) | 0.819 (0.805-0.832) | 0.657 (0.603-0.708) | 0.125 (0.120-0.131) |
| DT+RF+LightGBM+XGBoost | 6.286 | Rank 2 | 0.844 (0.833-0.856) | 0.728 (0.698-0.752) | 0.953 (0.946-0.961) | 0.964 (0.945-0.984) | 0.832 (0.820-0.845) | 0.640 (0.585-0.697) | 0.125 (0.120-0.130) |
| AdaBoost+RF+XGBoost+CatBoost | 6.714 | Rank 3 | 0.833 (0.820-0.845) | 0.725 (0.699-0.749) | 0.956 (0.949-0.962) | 0.985 (0.974-1.000) | 0.817 (0.804-0.832) | 0.614 (0.557-0.672) | 0.127 (0.122-0.132) |
| AdaBoost+LightGBM+XGBoost+CatBoost | 7.429 | Rank 4 | 0.818 (0.806-0.831) | 0.709 (0.681-0.735) | 0.953 (0.946-0.960) | 0.985 (0.974-1.000) | 0.802 (0.788-0.815) | 0.629 (0.576-0.679) | 0.130 (0.124-0.136) |
| AdaBoost+SVM+XGBoost+CatBoost | 8.143 | Rank 5 | 0.829 (0.816-0.841) | 0.717 (0.690-0.742) | 0.951 (0.943-0.958) | 0.980 (0.966-0.992) | 0.813 (0.800-0.828) | 0.617 (0.560-0.670) | 0.131 (0.125-0.137) |
| AdaBoost+RF+LightGBM+XGBoost | 8.714 | Rank 6 | 0.812 (0.800-0.825) | 0.707 (0.678-0.733) | 0.954 (0.947-0.961) | 0.995 (0.991-1.000) | 0.794 (0.780-0.809) | 0.646 (0.593-0.697) | 0.137 (0.131-0.143) |
| DT+RF+LightGBM+XGBoost+CatBoost | 9.714 | Rank 7 | 0.808 (0.796-0.821) | 0.703 (0.674-0.730) | 0.952 (0.944-0.959) | 0.995 (0.991-1.000) | 0.789 (0.776-0.803) | 0.650 (0.592-0.705) | 0.133 (0.126-0.138) |
| AdaBoost+DT+RF+LightGBM+XGBoost | 10.143 | Rank 8 | 0.820 (0.807-0.833) | 0.703 (0.673-0.731) | 0.949 (0.942-0.957) | 0.969 (0.952-0.992) | 0.805 (0.791-0.819) | 0.619 (0.564-0.676) | 0.131 (0.125-0.137) |
| AdaBoost+RF+LightGBM+XGBoost+CatBoost | 13.143 | Rank 9 | 0.810 (0.797-0.823) | 0.689 (0.659-0.720) | 0.950 (0.942-0.958) | 0.965 (0.946-0.984) | 0.795 (0.780-0.808) | 0.625 (0.566-0.683) | 0.136 (0.130-0.141) |
| DT+RF+SVM+XGBoost+CatBoost | 15.000 | Rank 10 | 0.805 (0.793-0.819) | 0.697 (0.669-0.723) | 0.948 (0.940-0.956) | 0.990 (0.982-1.000) | 0.787 (0.772-0.802) | 0.623 (0.564-0.676) | 0.138 (0.132-0.144) |
| AdaBoost+DT+RF+XGBoost | 18.000 | Rank 11 | 0.806 (0.793-0.819) | 0.685 (0.656-0.712) | 0.947 (0.939-0.956) | 0.964 (0.945-0.984) | 0.790 (0.776-0.804) | 0.631 (0.579-0.682) | 0.138 (0.132-0.144) |
| RF+XGBoost+CatBoost | 19.143 | Rank 12 | 0.805 (0.791-0.819) | 0.697 (0.667-0.727) | 0.948 (0.940-0.955) | 0.990 (0.983-1.000) | 0.786 (0.772-0.802) | 0.586 (0.527-0.640) | 0.138 (0.131-0.144) |
| AdaBoost+RF+XGBoost | 19.429 | Rank 13 | 0.811 (0.799-0.825) | 0.683 (0.651-0.710) | 0.947 (0.939-0.956) | 0.949 (0.927-0.974) | 0.797 (0.784-0.812) | 0.615 (0.559-0.669) | 0.133 (0.126-0.138) |
| RF+XGBoost | 20.143 | Rank 14 | 0.813 (0.800-0.826) | 0.684 (0.654-0.714) | 0.939 (0.929-0.949) | 0.949 (0.927-0.976) | 0.799 (0.786-0.814) | 0.598 (0.541-0.655) | 0.135 (0.130-0.140) |
| DT+RF+XGBoost | 23.143 | Rank 15 | 0.798 (0.785-0.811) | 0.684 (0.655-0.712) | 0.949 (0.942-0.957) | 0.979 (0.966-0.992) | 0.779 (0.766-0.794) | 0.630 (0.574-0.682) | 0.141 (0.135-0.147) |
| AdaBoost+DT+LightGBM+SVM+XGBoost+CatBoost | 23.429 | Rank 16 | 0.806 (0.794-0.818) | 0.685 (0.657-0.710) | 0.940 (0.931-0.948) | 0.964 (0.945-0.984) | 0.790 (0.777-0.803) | 0.581 (0.525-0.634) | 0.138 (0.132-0.145) |
| DT+RF+LightGBM+CatBoost | 23.857 | Rank 17 | 0.812 (0.800-0.826) | 0.681 (0.650-0.710) | 0.939 (0.929-0.949) | 0.944 (0.921-0.968) | 0.799 (0.785-0.813) | 0.594 (0.532-0.649) | 0.138 (0.133-0.143) |
| DT+RF+SVM+XGBoost | 24.286 | Rank 18 | 0.803 (0.790-0.817) | 0.684 (0.654-0.714) | 0.940 (0.931-0.950) | 0.969 (0.951-0.991) | 0.786 (0.772-0.801) | 0.587 (0.527-0.647) | 0.139 (0.133-0.146) |
| RF+LightGBM+XGBoost | 24.571 | Rank 19 | 0.801 (0.788-0.813) | 0.685 (0.655-0.712) | 0.938 (0.929-0.947) | 0.974 (0.959-0.992) | 0.783 (0.769-0.797) | 0.607 (0.552-0.663) | 0.141 (0.136-0.147) |
| DT+LightGBM+XGBoost+CatBoost | 25.714 | Rank 20 | 0.806 (0.793-0.819) | 0.683 (0.653-0.710) | 0.937 (0.928-0.946) | 0.959 (0.939-0.983) | 0.791 (0.777-0.804) | 0.552 (0.490-0.609) | 0.137 (0.131-0.143) |
| AdaBoost+RF+LightGBM+SVM+CatBoost | 26.429 | Rank 21 | 0.798 (0.785-0.811) | 0.676 (0.646-0.704) | 0.946 (0.937-0.954) | 0.964 (0.945-0.984) | 0.781 (0.767-0.796) | 0.620 (0.566-0.675) | 0.139 (0.133-0.145) |
| AdaBoost+DT+RF+CatBoost | 28.714 | Rank 22 | 0.813 (0.801-0.825) | 0.674 (0.643-0.703) | 0.936 (0.926-0.946) | 0.928 (0.901-0.957) | 0.801 (0.789-0.814) | 0.599 (0.541-0.657) | 0.136 (0.131-0.141) |
| RF+LightGBM+SVM+CatBoost | 29.571 | Rank 23 | 0.818 (0.806-0.830) | 0.681 (0.648-0.710) | 0.931 (0.921-0.941) | 0.934 (0.908-0.962) | 0.806 (0.793-0.819) | 0.554 (0.498-0.612) | 0.137 (0.132-0.143) |
| AdaBoost+DT+LightGBM+XGBoost+CatBoost | 29.714 | Rank 24 | 0.801 (0.788-0.814) | 0.687 (0.659-0.714) | 0.936 (0.928-0.945) | 0.979 (0.966-0.992) | 0.783 (0.769-0.798) | 0.544 (0.484-0.600) | 0.141 (0.135-0.148) |
| AdaBoost+DT+XGBoost+CatBoost | 32.714 | Rank 25 | 0.790 (0.776-0.803) | 0.686 (0.658-0.713) | 0.945 (0.937-0.953) | 1.000 (1.000-1.000) | 0.769 (0.754-0.783) | 0.562 (0.508-0.621) | 0.145 (0.139-0.150) |
| DT+RF+XGBoost+CatBoost | 35.000 | Rank 26 | 0.805 (0.793-0.819) | 0.678 (0.647-0.707) | 0.930 (0.921-0.940) | 0.954 (0.933-0.976) | 0.790 (0.776-0.805) | 0.526 (0.469-0.581) | 0.142 (0.136-0.148) |
| AdaBoost+DT+RF+LightGBM | 36.286 | Rank 27 | 0.798 (0.786-0.812) | 0.669 (0.638-0.699) | 0.937 (0.928-0.946) | 0.949 (0.926-0.976) | 0.783 (0.769-0.797) | 0.586 (0.530-0.643) | 0.141 (0.135-0.146) |
| AdaBoost+DT+RF+LightGBM+SVM+CatBoost | 37.429 | Rank 28 | 0.792 (0.779-0.806) | 0.671 (0.640-0.700) | 0.938 (0.928-0.946) | 0.964 (0.945-0.984) | 0.775 (0.761-0.790) | 0.587 (0.531-0.638) | 0.146 (0.140-0.152) |
| AdaBoost+DT+RF+LightGBM+SVM+XGBoost+CatBoost | 37.429 | Rank 28 | 0.800 (0.787-0.814) | 0.672 (0.640-0.702) | 0.934 (0.925-0.943) | 0.954 (0.933-0.976) | 0.784 (0.770-0.799) | 0.558 (0.497-0.614) | 0.144 (0.137-0.150) |
| AdaBoost+RF+CatBoost | 39.286 | Rank 30 | 0.802 (0.788-0.814) | 0.675 (0.644-0.704) | 0.929 (0.919-0.939) | 0.954 (0.933-0.976) | 0.786 (0.771-0.799) | 0.540 (0.482-0.598) | 0.143 (0.138-0.149) |
| DT+SVM+XGBoost+CatBoost | 39.571 | Rank 31 | 0.804 (0.790-0.816) | 0.656 (0.624-0.687) | 0.932 (0.921-0.942) | 0.913 (0.885-0.944) | 0.793 (0.778-0.806) | 0.590 (0.530-0.644) | 0.138 (0.133-0.144) |
| AdaBoost+RF+LightGBM | 40.286 | Rank 32 | 0.789 (0.776-0.803) | 0.670 (0.638-0.697) | 0.942 (0.934-0.951) | 0.969 (0.952-0.992) | 0.771 (0.757-0.786) | 0.593 (0.529-0.653) | 0.149 (0.142-0.156) |
| AdaBoost+DT+RF+SVM+CatBoost | 40.571 | Rank 33 | 0.790 (0.777-0.803) | 0.666 (0.637-0.693) | 0.937 (0.929-0.947) | 0.959 (0.938-0.983) | 0.773 (0.758-0.787) | 0.563 (0.506-0.620) | 0.142 (0.135-0.148) |
| AdaBoost+RF+SVM+XGBoost+CatBoost | 41.286 | Rank 34 | 0.790 (0.777-0.803) | 0.665 (0.633-0.692) | 0.936 (0.927-0.946) | 0.959 (0.939-0.983) | 0.773 (0.760-0.786) | 0.604 (0.550-0.656) | 0.147 (0.140-0.153) |
| AdaBoost+DT+RF+LightGBM+XGBoost+CatBoost | 41.571 | Rank 35 | 0.789 (0.776-0.802) | 0.670 (0.640-0.699) | 0.937 (0.928-0.946) | 0.970 (0.952-0.991) | 0.770 (0.757-0.785) | 0.584 (0.528-0.640) | 0.147 (0.140-0.153) |
| AdaBoost+RF+SVM+XGBoost | 43.143 | Rank 36 | 0.786 (0.773-0.800) | 0.662 (0.631-0.690) | 0.938 (0.929-0.947) | 0.960 (0.939-0.984) | 0.769 (0.755-0.783) | 0.603 (0.549-0.656) | 0.146 (0.140-0.153) |
| AdaBoost+XGBoost+CatBoost | 44.143 | Rank 37 | 0.785 (0.771-0.797) | 0.676 (0.647-0.701) | 0.942 (0.934-0.950) | 0.990 (0.982-1.000) | 0.764 (0.749-0.779) | 0.579 (0.522-0.629) | 0.151 (0.145-0.156) |
| LightGBM+XGBoost | 44.143 | Rank 37 | 0.786 (0.772-0.799) | 0.683 (0.654-0.709) | 0.946 (0.938-0.954) | 1.000 (1.000-1.000) | 0.764 (0.750-0.779) | 0.531 (0.475-0.591) | 0.153 (0.147-0.159) |
| AdaBoost+DT+RF+XGBoost+CatBoost | 45.143 | Rank 39 | 0.788 (0.775-0.803) | 0.661 (0.629-0.691) | 0.932 (0.922-0.941) | 0.954 (0.932-0.976) | 0.772 (0.758-0.786) | 0.594 (0.536-0.647) | 0.145 (0.139-0.151) |
| DT+LightGBM+SVM+XGBoost+CatBoost | 45.286 | Rank 40 | 0.787 (0.774-0.801) | 0.663 (0.632-0.693) | 0.939 (0.930-0.948) | 0.959 (0.937-0.982) | 0.770 (0.755-0.784) | 0.564 (0.500-0.626) | 0.146 (0.140-0.151) |
| RF+SVM+CatBoost | 46.714 | Rank 41 | 0.804 (0.791-0.818) | 0.659 (0.628-0.690) | 0.923 (0.912-0.934) | 0.918 (0.891-0.949) | 0.792 (0.778-0.807) | 0.548 (0.493-0.607) | 0.140 (0.134-0.146) |
| LightGBM+SVM+XGBoost+CatBoost | 47.857 | Rank 42 | 0.800 (0.788-0.814) | 0.660 (0.627-0.689) | 0.926 (0.916-0.936) | 0.928 (0.902-0.957) | 0.787 (0.773-0.801) | 0.524 (0.463-0.584) | 0.142 (0.135-0.148) |
| DT+XGBoost+CatBoost | 48.429 | Rank 43 | 0.790 (0.777-0.802) | 0.657 (0.624-0.685) | 0.933 (0.923-0.942) | 0.943 (0.921-0.968) | 0.775 (0.760-0.787) | 0.560 (0.502-0.619) | 0.145 (0.140-0.151) |
| AdaBoost+LightGBM+XGBoost | 48.714 | Rank 44 | 0.791 (0.778-0.804) | 0.687 (0.659-0.714) | 0.930 (0.922-0.939) | 1.000 (1.000-1.000) | 0.770 (0.754-0.784) | 0.438 (0.389-0.493) | 0.151 (0.145-0.157) |
| RF+LightGBM+SVM+XGBoost+CatBoost | 49.000 | Rank 45 | 0.800 (0.787-0.812) | 0.662 (0.632-0.692) | 0.926 (0.915-0.936) | 0.934 (0.908-0.962) | 0.786 (0.772-0.799) | 0.515 (0.456-0.574) | 0.143 (0.137-0.149) |
| DT+RF+LightGBM+SVM+XGBoost | 49.714 | Rank 46 | 0.786 (0.773-0.799) | 0.662 (0.632-0.690) | 0.937 (0.927-0.947) | 0.959 (0.940-0.983) | 0.769 (0.754-0.783) | 0.575 (0.513-0.636) | 0.148 (0.142-0.155) |
| AdaBoost+DT+LightGBM+CatBoost | 49.857 | Rank 47 | 0.797 (0.784-0.810) | 0.654 (0.622-0.683) | 0.929 (0.919-0.939) | 0.923 (0.896-0.952) | 0.784 (0.770-0.797) | 0.535 (0.479-0.591) | 0.140 (0.135-0.146) |
| AdaBoost+DT+RF+LightGBM+CatBoost | 50.143 | Rank 48 | 0.788 (0.774-0.801) | 0.655 (0.623-0.686) | 0.930 (0.921-0.940) | 0.943 (0.921-0.969) | 0.772 (0.758-0.786) | 0.575 (0.517-0.632) | 0.144 (0.138-0.150) |
| AdaBoost+DT+LightGBM+SVM+XGBoost | 51.571 | Rank 49 | 0.791 (0.778-0.805) | 0.670 (0.639-0.697) | 0.930 (0.921-0.939) | 0.964 (0.945-0.984) | 0.774 (0.760-0.788) | 0.484 (0.428-0.539) | 0.148 (0.142-0.154) |
| AdaBoost+DT+RF+SVM+XGBoost | 51.571 | Rank 49 | 0.794 (0.781-0.807) | 0.659 (0.626-0.690) | 0.928 (0.918-0.938) | 0.939 (0.914-0.966) | 0.779 (0.766-0.793) | 0.518 (0.457-0.574) | 0.143 (0.138-0.149) |
| DT+RF+SVM | 52.000 | Rank 51 | 0.805 (0.792-0.817) | 0.651 (0.620-0.681) | 0.918 (0.907-0.929) | 0.903 (0.871-0.933) | 0.795 (0.781-0.808) | 0.508 (0.454-0.563) | 0.136 (0.131-0.142) |
| XGBoost+CatBoost | 52.857 | Rank 52 | 0.795 (0.782-0.809) | 0.668 (0.639-0.696) | 0.928 (0.918-0.937) | 0.954 (0.933-0.976) | 0.779 (0.765-0.793) | 0.488 (0.434-0.546) | 0.147 (0.141-0.153) |
| LightGBM+CatBoost | 53.286 | Rank 53 | 0.799 (0.787-0.812) | 0.662 (0.633-0.691) | 0.923 (0.914-0.934) | 0.933 (0.908-0.960) | 0.785 (0.771-0.798) | 0.500 (0.447-0.552) | 0.144 (0.138-0.150) |
| DT+LightGBM+XGBoost | 54.000 | Rank 54 | 0.787 (0.775-0.800) | 0.683 (0.655-0.710) | 0.933 (0.926-0.942) | 1.000 (1.000-1.000) | 0.765 (0.753-0.779) | 0.478 (0.425-0.529) | 0.158 (0.152-0.164) |
| DT+LightGBM | 54.286 | Rank 55 | 0.788 (0.775-0.801) | 0.679 (0.650-0.706) | 0.937 (0.928-0.945) | 0.990 (0.982-1.000) | 0.768 (0.754-0.781) | 0.500 (0.445-0.557) | 0.160 (0.156-0.165) |
| AdaBoost+LightGBM+CatBoost | 55.857 | Rank 56 | 0.805 (0.792-0.817) | 0.654 (0.623-0.686) | 0.920 (0.910-0.930) | 0.909 (0.879-0.941) | 0.794 (0.780-0.807) | 0.498 (0.439-0.552) | 0.143 (0.138-0.149) |
| LightGBM+SVM+XGBoost | 56.000 | Rank 57 | 0.796 (0.783-0.808) | 0.666 (0.636-0.694) | 0.928 (0.918-0.937) | 0.949 (0.927-0.975) | 0.780 (0.766-0.794) | 0.479 (0.430-0.533) | 0.150 (0.144-0.156) |
| DT+RF+LightGBM+SVM+XGBoost+CatBoost | 56.857 | Rank 58 | 0.770 (0.756-0.784) | 0.661 (0.630-0.688) | 0.942 (0.934-0.950) | 0.990 (0.983-1.000) | 0.748 (0.733-0.763) | 0.573 (0.516-0.628) | 0.154 (0.148-0.160) |
| AdaBoost+DT+CatBoost | 57.143 | Rank 59 | 0.799 (0.786-0.813) | 0.646 (0.611-0.678) | 0.917 (0.906-0.930) | 0.903 (0.874-0.938) | 0.789 (0.775-0.803) | 0.542 (0.485-0.599) | 0.144 (0.139-0.149) |
| DT+LightGBM+CatBoost | 57.571 | Rank 60 | 0.790 (0.777-0.803) | 0.657 (0.627-0.686) | 0.926 (0.917-0.937) | 0.943 (0.921-0.969) | 0.774 (0.760-0.788) | 0.518 (0.460-0.572) | 0.149 (0.143-0.155) |
| AdaBoost+RF | 58.286 | Rank 61 | 0.784 (0.771-0.798) | 0.647 (0.616-0.676) | 0.930 (0.919-0.941) | 0.933 (0.908-0.961) | 0.769 (0.754-0.783) | 0.588 (0.530-0.643) | 0.148 (0.142-0.154) |
| AdaBoost+RF+LightGBM+SVM+XGBoost | 58.429 | Rank 62 | 0.794 (0.782-0.807) | 0.652 (0.620-0.683) | 0.923 (0.911-0.935) | 0.923 (0.895-0.952) | 0.781 (0.768-0.796) | 0.537 (0.478-0.591) | 0.149 (0.144-0.154) |
| SVM+XGBoost+CatBoost | 60.143 | Rank 63 | 0.778 (0.765-0.790) | 0.649 (0.615-0.678) | 0.930 (0.920-0.940) | 0.950 (0.927-0.975) | 0.760 (0.746-0.774) | 0.571 (0.513-0.634) | 0.148 (0.141-0.153) |
| DT+RF+SVM+CatBoost | 61.571 | Rank 64 | 0.800 (0.787-0.813) | 0.644 (0.611-0.674) | 0.915 (0.904-0.928) | 0.898 (0.870-0.931) | 0.790 (0.775-0.804) | 0.514 (0.450-0.569) | 0.146 (0.140-0.151) |
| RF+LightGBM+XGBoost+CatBoost | 63.571 | Rank 65 | 0.793 (0.780-0.806) | 0.647 (0.613-0.677) | 0.920 (0.910-0.930) | 0.919 (0.892-0.950) | 0.780 (0.766-0.794) | 0.509 (0.449-0.571) | 0.146 (0.140-0.152) |
| RF+SVM+XGBoost+CatBoost | 63.571 | Rank 65 | 0.782 (0.767-0.795) | 0.642 (0.610-0.673) | 0.926 (0.915-0.936) | 0.929 (0.903-0.959) | 0.767 (0.752-0.781) | 0.557 (0.497-0.611) | 0.146 (0.140-0.152) |
| AdaBoost+LightGBM+SVM+XGBoost+CatBoost | 65.714 | Rank 67 | 0.780 (0.767-0.793) | 0.650 (0.620-0.678) | 0.927 (0.918-0.937) | 0.949 (0.927-0.974) | 0.762 (0.749-0.777) | 0.522 (0.464-0.577) | 0.150 (0.143-0.156) |
| RF+SVM+XGBoost | 67.571 | Rank 68 | 0.778 (0.764-0.791) | 0.641 (0.607-0.671) | 0.927 (0.917-0.938) | 0.933 (0.908-0.960) | 0.762 (0.747-0.777) | 0.578 (0.519-0.633) | 0.150 (0.144-0.156) |
| AdaBoost+XGBoost | 68.286 | Rank 69 | 0.787 (0.773-0.801) | 0.681 (0.651-0.709) | 0.916 (0.906-0.925) | 0.995 (0.991-1.000) | 0.766 (0.751-0.781) | 0.417 (0.366-0.473) | 0.169 (0.163-0.174) |
| RF+LightGBM | 68.286 | Rank 69 | 0.776 (0.762-0.789) | 0.651 (0.624-0.681) | 0.928 (0.918-0.937) | 0.959 (0.940-0.983) | 0.757 (0.742-0.772) | 0.515 (0.455-0.577) | 0.154 (0.148-0.160) |
| AdaBoost+LightGBM | 68.429 | Rank 71 | 0.784 (0.771-0.798) | 0.678 (0.646-0.705) | 0.919 (0.909-0.928) | 0.995 (0.991-1.000) | 0.763 (0.749-0.778) | 0.425 (0.375-0.477) | 0.163 (0.157-0.169) |
| AdaBoost+RF+SVM+CatBoost | 69.000 | Rank 72 | 0.782 (0.769-0.796) | 0.642 (0.609-0.673) | 0.924 (0.913-0.934) | 0.928 (0.902-0.954) | 0.767 (0.753-0.783) | 0.540 (0.480-0.595) | 0.150 (0.144-0.156) |
| AdaBoost+DT+LightGBM+XGBoost | 69.429 | Rank 73 | 0.778 (0.766-0.792) | 0.675 (0.646-0.703) | 0.922 (0.913-0.931) | 1.000 (1.000-1.000) | 0.756 (0.742-0.771) | 0.438 (0.386-0.493) | 0.162 (0.155-0.170) |
| DT+XGBoost | 69.571 | Rank 74 | 0.782 (0.769-0.796) | 0.679 (0.649-0.705) | 0.920 (0.911-0.929) | 1.000 (1.000-1.000) | 0.760 (0.746-0.775) | 0.419 (0.372-0.472) | 0.171 (0.165-0.176) |
| DT+CatBoost | 71.571 | Rank 75 | 0.789 (0.776-0.802) | 0.626 (0.594-0.659) | 0.917 (0.905-0.928) | 0.884 (0.850-0.921) | 0.779 (0.766-0.794) | 0.512 (0.454-0.569) | 0.147 (0.141-0.152) |
| AdaBoost+DT+SVM+XGBoost+CatBoost | 71.714 | Rank 76 | 0.778 (0.765-0.791) | 0.648 (0.617-0.678) | 0.922 (0.911-0.934) | 0.950 (0.926-0.975) | 0.761 (0.747-0.774) | 0.509 (0.448-0.574) | 0.154 (0.148-0.160) |
| AdaBoost+RF+LightGBM+SVM | 71.714 | Rank 76 | 0.772 (0.758-0.785) | 0.619 (0.585-0.648) | 0.929 (0.919-0.941) | 0.902 (0.872-0.933) | 0.758 (0.744-0.773) | 0.600 (0.542-0.653) | 0.148 (0.142-0.154) |
| DT+RF+LightGBM+SVM+CatBoost | 72.286 | Rank 78 | 0.788 (0.775-0.800) | 0.646 (0.615-0.676) | 0.917 (0.905-0.928) | 0.925 (0.896-0.952) | 0.774 (0.760-0.787) | 0.466 (0.410-0.524) | 0.149 (0.143-0.156) |
| AdaBoost+LightGBM+SVM+XGBoost | 75.286 | Rank 79 | 0.770 (0.756-0.785) | 0.657 (0.626-0.683) | 0.928 (0.919-0.937) | 0.979 (0.966-0.992) | 0.749 (0.734-0.765) | 0.483 (0.428-0.539) | 0.163 (0.156-0.169) |
| DT+RF | 76.000 | Rank 80 | 0.781 (0.769-0.795) | 0.634 (0.600-0.664) | 0.914 (0.902-0.925) | 0.913 (0.887-0.943) | 0.768 (0.754-0.783) | 0.522 (0.459-0.582) | 0.149 (0.143-0.154) |
| AdaBoost+DT+RF | 76.286 | Rank 81 | 0.779 (0.766-0.793) | 0.639 (0.608-0.669) | 0.920 (0.909-0.932) | 0.928 (0.901-0.960) | 0.764 (0.750-0.778) | 0.520 (0.468-0.577) | 0.156 (0.150-0.162) |
| LightGBM+XGBoost+CatBoost | 77.143 | Rank 82 | 0.772 (0.759-0.786) | 0.648 (0.616-0.676) | 0.922 (0.912-0.932) | 0.959 (0.940-0.983) | 0.753 (0.738-0.768) | 0.492 (0.436-0.548) | 0.155 (0.149-0.161) |
| AdaBoost+DT+SVM+XGBoost | 79.286 | Rank 83 | 0.784 (0.771-0.797) | 0.645 (0.611-0.673) | 0.911 (0.901-0.922) | 0.928 (0.901-0.957) | 0.770 (0.755-0.784) | 0.438 (0.386-0.489) | 0.153 (0.147-0.160) |
| AdaBoost+RF+LightGBM+CatBoost | 80.143 | Rank 84 | 0.774 (0.760-0.788) | 0.640 (0.609-0.671) | 0.917 (0.905-0.927) | 0.939 (0.915-0.967) | 0.757 (0.742-0.772) | 0.518 (0.459-0.570) | 0.157 (0.149-0.163) |
| RF+CatBoost | 80.571 | Rank 85 | 0.770 (0.756-0.783) | 0.625 (0.592-0.657) | 0.919 (0.907-0.932) | 0.919 (0.892-0.950) | 0.755 (0.740-0.770) | 0.548 (0.486-0.604) | 0.149 (0.143-0.156) |
| AdaBoost+DT+RF+LightGBM+SVM | 80.714 | Rank 86 | 0.762 (0.749-0.776) | 0.636 (0.603-0.665) | 0.922 (0.912-0.931) | 0.954 (0.931-0.976) | 0.743 (0.729-0.757) | 0.529 (0.475-0.579) | 0.156 (0.150-0.163) |
| SVM+CatBoost | 80.714 | Rank 86 | 0.788 (0.775-0.802) | 0.613 (0.578-0.647) | 0.901 (0.888-0.913) | 0.861 (0.825-0.901) | 0.781 (0.767-0.795) | 0.455 (0.398-0.508) | 0.146 (0.140-0.152) |
| AdaBoost+DT+LightGBM+SVM+CatBoost | 80.857 | Rank 88 | 0.776 (0.763-0.790) | 0.636 (0.604-0.664) | 0.922 (0.911-0.933) | 0.928 (0.901-0.958) | 0.761 (0.747-0.776) | 0.499 (0.438-0.564) | 0.154 (0.148-0.160) |
| AdaBoost+RF+LightGBM+SVM+XGBoost+CatBoost | 82.143 | Rank 89 | 0.762 (0.748-0.776) | 0.629 (0.597-0.659) | 0.924 (0.913-0.936) | 0.938 (0.913-0.966) | 0.745 (0.729-0.759) | 0.551 (0.495-0.602) | 0.161 (0.154-0.168) |
| AdaBoost+DT+LightGBM+SVM | 82.429 | Rank 90 | 0.772 (0.758-0.786) | 0.641 (0.609-0.670) | 0.923 (0.913-0.934) | 0.945 (0.921-0.969) | 0.755 (0.739-0.769) | 0.477 (0.420-0.539) | 0.158 (0.152-0.164) |
| RF+LightGBM+CatBoost | 84.714 | Rank 91 | 0.774 (0.760-0.787) | 0.629 (0.598-0.659) | 0.912 (0.899-0.924) | 0.918 (0.892-0.951) | 0.759 (0.744-0.774) | 0.511 (0.452-0.564) | 0.153 (0.147-0.159) |
| AdaBoost+SVM+XGBoost | 85.000 | Rank 92 | 0.780 (0.767-0.794) | 0.640 (0.609-0.671) | 0.915 (0.904-0.926) | 0.929 (0.902-0.958) | 0.765 (0.751-0.779) | 0.416 (0.367-0.465) | 0.157 (0.151-0.164) |
| DT+RF+CatBoost | 85.286 | Rank 93 | 0.774 (0.761-0.787) | 0.632 (0.599-0.662) | 0.915 (0.903-0.927) | 0.923 (0.895-0.951) | 0.759 (0.745-0.774) | 0.501 (0.444-0.555) | 0.155 (0.149-0.162) |
| DT+RF+LightGBM | 87.000 | Rank 94 | 0.767 (0.753-0.781) | 0.625 (0.592-0.656) | 0.919 (0.907-0.930) | 0.924 (0.895-0.953) | 0.751 (0.737-0.766) | 0.511 (0.455-0.567) | 0.154 (0.148-0.161) |
| DT+LightGBM+SVM+CatBoost | 87.571 | Rank 95 | 0.773 (0.758-0.786) | 0.623 (0.588-0.652) | 0.916 (0.904-0.927) | 0.908 (0.878-0.939) | 0.759 (0.744-0.773) | 0.509 (0.453-0.570) | 0.155 (0.148-0.161) |
| DT+SVM+CatBoost | 88.429 | Rank 96 | 0.774 (0.762-0.788) | 0.614 (0.581-0.647) | 0.907 (0.894-0.920) | 0.889 (0.854-0.923) | 0.762 (0.750-0.777) | 0.504 (0.448-0.560) | 0.153 (0.147-0.158) |
| AdaBoost+DT+XGBoost | 91.857 | Rank 97 | 0.786 (0.772-0.800) | 0.620 (0.584-0.652) | 0.901 (0.890-0.913) | 0.877 (0.844-0.913) | 0.777 (0.762-0.792) | 0.406 (0.352-0.461) | 0.164 (0.159-0.170) |
| SVM+XGBoost | 91.857 | Rank 97 | 0.777 (0.764-0.790) | 0.609 (0.575-0.643) | 0.907 (0.894-0.920) | 0.873 (0.839-0.908) | 0.768 (0.754-0.782) | 0.448 (0.391-0.504) | 0.155 (0.149-0.160) |
| AdaBoost+LightGBM+SVM | 92.000 | Rank 99 | 0.773 (0.760-0.787) | 0.638 (0.607-0.669) | 0.905 (0.893-0.916) | 0.938 (0.913-0.966) | 0.756 (0.742-0.771) | 0.411 (0.364-0.463) | 0.160 (0.153-0.166) |
| AdaBoost+DT+RF+LightGBM+SVM+XGBoost | 95.143 | Rank 100 | 0.767 (0.754-0.781) | 0.592 (0.558-0.623) | 0.908 (0.895-0.921) | 0.857 (0.821-0.894) | 0.758 (0.744-0.773) | 0.511 (0.454-0.571) | 0.156 (0.149-0.162) |
| AdaBoost+LightGBM+SVM+CatBoost | 96.000 | Rank 101 | 0.765 (0.752-0.779) | 0.613 (0.579-0.642) | 0.909 (0.896-0.920) | 0.902 (0.870-0.934) | 0.751 (0.737-0.765) | 0.496 (0.434-0.549) | 0.157 (0.151-0.163) |
| AdaBoost+RF+SVM | 96.429 | Rank 102 | 0.765 (0.751-0.779) | 0.597 (0.564-0.629) | 0.898 (0.885-0.913) | 0.872 (0.836-0.906) | 0.754 (0.739-0.769) | 0.530 (0.474-0.583) | 0.159 (0.153-0.165) |
| AdaBoost+CatBoost | 97.286 | Rank 103 | 0.764 (0.751-0.778) | 0.628 (0.595-0.657) | 0.902 (0.890-0.915) | 0.933 (0.907-0.961) | 0.747 (0.732-0.762) | 0.448 (0.400-0.503) | 0.167 (0.160-0.174) |
| AdaBoost+DT+SVM+CatBoost | 98.286 | Rank 104 | 0.770 (0.756-0.783) | 0.615 (0.583-0.645) | 0.903 (0.891-0.917) | 0.898 (0.866-0.934) | 0.757 (0.742-0.772) | 0.478 (0.423-0.534) | 0.162 (0.156-0.168) |
| DT+RF+LightGBM+SVM | 99.000 | Rank 105 | 0.744 (0.729-0.758) | 0.604 (0.572-0.634) | 0.912 (0.900-0.924) | 0.924 (0.897-0.952) | 0.725 (0.710-0.741) | 0.516 (0.462-0.569) | 0.166 (0.159-0.173) |
| NaiveBayes | 99.143 | Rank 106 | 0.785 (0.745-0.826) | 0.294 (0.208-0.380) | 0.669 (0.641-0.696) | 0.385 (0.254-0.515) | 0.827 (0.769-0.885) | 0.175 (0.138-0.213) | 0.182 (0.156-0.209) |
| AdaBoost+DT+RF+SVM | 99.857 | Rank 107 | 0.760 (0.746-0.775) | 0.607 (0.573-0.636) | 0.902 (0.887-0.918) | 0.898 (0.866-0.933) | 0.746 (0.730-0.762) | 0.500 (0.445-0.557) | 0.160 (0.154-0.166) |
| DT+LightGBM+SVM | 100.000 | Rank 108 | 0.760 (0.747-0.774) | 0.623 (0.589-0.652) | 0.906 (0.894-0.917) | 0.929 (0.904-0.957) | 0.743 (0.729-0.757) | 0.415 (0.359-0.471) | 0.162 (0.155-0.169) |
| RF+LightGBM+SVM | 102.571 | Rank 109 | 0.754 (0.739-0.768) | 0.582 (0.547-0.617) | 0.894 (0.880-0.910) | 0.862 (0.826-0.900) | 0.743 (0.729-0.758) | 0.518 (0.458-0.575) | 0.160 (0.154-0.167) |
| AdaBoost+DT+LightGBM | 102.857 | Rank 110 | 0.753 (0.739-0.768) | 0.623 (0.591-0.654) | 0.900 (0.889-0.912) | 0.944 (0.920-0.968) | 0.734 (0.718-0.749) | 0.395 (0.344-0.446) | 0.171 (0.165-0.177) |
| LightGBM+SVM+CatBoost | 104.714 | Rank 111 | 0.764 (0.750-0.777) | 0.589 (0.554-0.619) | 0.892 (0.878-0.906) | 0.857 (0.821-0.895) | 0.754 (0.740-0.769) | 0.460 (0.404-0.512) | 0.160 (0.154-0.166) |
| RF+SVM | 106.429 | Rank 112 | 0.757 (0.742-0.771) | 0.598 (0.565-0.632) | 0.892 (0.877-0.908) | 0.888 (0.855-0.924) | 0.743 (0.729-0.758) | 0.472 (0.414-0.528) | 0.165 (0.158-0.171) |
| AdaBoost+SVM+CatBoost | 107.857 | Rank 113 | 0.756 (0.742-0.769) | 0.586 (0.553-0.617) | 0.893 (0.879-0.905) | 0.867 (0.831-0.904) | 0.744 (0.730-0.760) | 0.418 (0.363-0.471) | 0.160 (0.154-0.165) |
| DT+LightGBM+SVM+XGBoost | 108.857 | Rank 114 | 0.751 (0.738-0.765) | 0.591 (0.557-0.621) | 0.902 (0.890-0.915) | 0.882 (0.851-0.918) | 0.738 (0.723-0.753) | 0.432 (0.380-0.487) | 0.165 (0.158-0.171) |
| LightGBM+SVM | 110.571 | Rank 115 | 0.753 (0.740-0.767) | 0.592 (0.556-0.623) | 0.896 (0.881-0.910) | 0.881 (0.847-0.917) | 0.740 (0.726-0.755) | 0.413 (0.355-0.465) | 0.167 (0.161-0.173) |
| DT+SVM+XGBoost | 112.714 | Rank 116 | 0.743 (0.728-0.756) | 0.566 (0.533-0.598) | 0.891 (0.876-0.905) | 0.847 (0.811-0.887) | 0.732 (0.717-0.746) | 0.435 (0.385-0.488) | 0.163 (0.158-0.169) |
| RF+LightGBM+SVM+XGBoost | 113.000 | Rank 117 | 0.751 (0.737-0.766) | 0.572 (0.537-0.606) | 0.882 (0.867-0.898) | 0.847 (0.807-0.887) | 0.742 (0.726-0.757) | 0.432 (0.380-0.484) | 0.167 (0.161-0.174) |
| AdaBoost+SVM | 118.143 | Rank 118 | 0.720 (0.706-0.735) | 0.491 (0.454-0.526) | 0.796 (0.774-0.818) | 0.739 (0.691-0.787) | 0.718 (0.703-0.734) | 0.265 (0.225-0.305) | 0.187 (0.180-0.193) |
| AdaBoost+DT+SVM | 118.714 | Rank 119 | 0.714 (0.699-0.728) | 0.516 (0.481-0.552) | 0.795 (0.771-0.820) | 0.796 (0.756-0.841) | 0.706 (0.691-0.720) | 0.275 (0.232-0.318) | 0.191 (0.185-0.198) |
| DT+SVM | 119.429 | Rank 120 | 0.712 (0.697-0.727) | 0.490 (0.455-0.523) | 0.793 (0.769-0.816) | 0.747 (0.702-0.793) | 0.709 (0.693-0.724) | 0.262 (0.221-0.300) | 0.191 (0.185-0.197) |
| RF | 122.000 | Rank 121 | 0.706 (0.682-0.730) | 0.337 (0.300-0.374) | 0.669 (0.625-0.712) | 0.491 (0.419-0.564) | 0.729 (0.706-0.752) | 0.195 (0.144-0.245) | 0.188 (0.180-0.196) |
| AdaBoost+DT | 122.857 | Rank 122 | 0.640 (0.624-0.655) | 0.435 (0.402-0.468) | 0.718 (0.691-0.746) | 0.730 (0.683-0.779) | 0.631 (0.613-0.647) | 0.205 (0.169-0.243) | 0.220 (0.215-0.225) |
| SVM | 123.000 | Rank 123 | 0.678 (0.649-0.707) | 0.355 (0.305-0.405) | 0.681 (0.645-0.716) | 0.546 (0.456-0.635) | 0.691 (0.662-0.720) | 0.193 (0.158-0.229) | 0.203 (0.192-0.214) |
| AdaBoost | 123.143 | Rank 124 | 0.662 (0.621-0.703) | 0.371 (0.330-0.411) | 0.677 (0.637-0.716) | 0.586 (0.521-0.652) | 0.669 (0.622-0.717) | 0.209 (0.158-0.261) | 0.221 (0.202-0.239) |
| CatBoost | 124.286 | Rank 125 | 0.692 (0.671-0.712) | 0.305 (0.236-0.375) | 0.650 (0.596-0.705) | 0.452 (0.334-0.571) | 0.716 (0.699-0.734) | 0.179 (0.134-0.223) | 0.194 (0.182-0.205) |
| LightGBM | 124.714 | Rank 126 | 0.680 (0.654-0.705) | 0.334 (0.277-0.390) | 0.641 (0.589-0.693) | 0.508 (0.411-0.605) | 0.698 (0.673-0.723) | 0.187 (0.137-0.237) | 0.221 (0.206-0.237) |
| KNN | 126.429 | Rank 127 | 0.637 (0.603-0.670) | 0.345 (0.317-0.372) | 0.634 (0.613-0.656) | 0.562 (0.513-0.611) | 0.644 (0.605-0.682) | 0.150 (0.109-0.191) | 0.257 (0.239-0.275) |
| DT | 126.571 | Rank 128 | 0.603 (0.565-0.642) | 0.354 (0.316-0.391) | 0.633 (0.603-0.664) | 0.607 (0.554-0.660) | 0.603 (0.560-0.646) | 0.144 (0.125-0.162) | 0.238 (0.226-0.250) |
| XGBoost | 126.857 | Rank 129 | 0.669 (0.648-0.690) | 0.325 (0.275-0.376) | 0.624 (0.582-0.666) | 0.500 (0.429-0.570) | 0.686 (0.663-0.709) | 0.172 (0.126-0.218) | 0.270 (0.253-0.287) |

**Supplementary Table 17.** Ranking of 129 machine learning models in the ELSA validation set.

| **Model** | **Normalized Rank Score** | **Comprehensive Rank** | **Accuracy (95% CI)** | **F_Beta (95% CI)** | **ROC_AUC (95% CI)** | **Sensitivity (95% CI)** | **Specificity (95% CI)** | **PR_AUC (95% CI)** | **Brier (95% CI)** |
| --- | --- | --- | --- | --- | --- | --- | --- | --- | --- |
| AdaBoost+DT+LightGBM+CatBoost | 3.857 | Rank 1 | 0.763 (0.743-0.784) | 0.429 (0.367-0.490) | 0.719 (0.667-0.772) | 0.637 (0.548-0.725) | 0.774 (0.753-0.795) | 0.218 (0.152-0.285) | 0.163 (0.153-0.172) |
| AdaBoost+RF+XGBoost+CatBoost | 11.857 | Rank 2 | 0.763 (0.741-0.784) | 0.379 (0.311-0.440) | 0.716 (0.666-0.764) | 0.549 (0.458-0.636) | 0.780 (0.759-0.801) | 0.206 (0.142-0.282) | 0.161 (0.152-0.171) |
| DT+RF+XGBoost+CatBoost | 14.429 | Rank 3 | 0.755 (0.733-0.775) | 0.381 (0.312-0.444) | 0.716 (0.671-0.768) | 0.563 (0.475-0.658) | 0.770 (0.749-0.791) | 0.208 (0.144-0.264) | 0.173 (0.163-0.183) |
| LightGBM+SVM+XGBoost+CatBoost | 15.571 | Rank 4 | 0.752 (0.733-0.774) | 0.388 (0.318-0.447) | 0.711 (0.661-0.761) | 0.579 (0.488-0.674) | 0.767 (0.746-0.787) | 0.192 (0.127-0.252) | 0.172 (0.162-0.182) |
| DT+LightGBM+XGBoost+CatBoost | 15.857 | Rank 5 | 0.751 (0.730-0.774) | 0.389 (0.320-0.448) | 0.712 (0.661-0.761) | 0.582 (0.489-0.673) | 0.765 (0.744-0.788) | 0.182 (0.126-0.235) | 0.170 (0.159-0.180) |
| AdaBoost+RF+SVM+XGBoost+CatBoost | 16.857 | Rank 6 | 0.740 (0.719-0.762) | 0.403 (0.336-0.461) | 0.721 (0.670-0.774) | 0.620 (0.535-0.711) | 0.750 (0.728-0.773) | 0.208 (0.139-0.272) | 0.176 (0.166-0.186) |
| AdaBoost+DT+RF+SVM+XGBoost | 17.714 | Rank 7 | 0.748 (0.726-0.770) | 0.378 (0.309-0.439) | 0.720 (0.670-0.774) | 0.566 (0.474-0.667) | 0.763 (0.741-0.785) | 0.194 (0.136-0.261) | 0.172 (0.162-0.183) |
| RF+LightGBM+SVM+CatBoost | 27.000 | Rank 8 | 0.740 (0.718-0.762) | 0.366 (0.298-0.423) | 0.706 (0.657-0.757) | 0.552 (0.459-0.644) | 0.756 (0.734-0.779) | 0.182 (0.127-0.244) | 0.171 (0.161-0.180) |
| SVM+CatBoost | 28.571 | Rank 9 | 0.740 (0.719-0.761) | 0.364 (0.298-0.424) | 0.701 (0.648-0.756) | 0.551 (0.457-0.644) | 0.755 (0.733-0.777) | 0.188 (0.127-0.242) | 0.170 (0.160-0.180) |
| AdaBoost+DT+LightGBM+SVM+XGBoost+CatBoost | 30.714 | Rank 10 | 0.741 (0.719-0.762) | 0.373 (0.307-0.432) | 0.702 (0.653-0.755) | 0.565 (0.476-0.659) | 0.756 (0.735-0.778) | 0.165 (0.121-0.214) | 0.172 (0.162-0.182) |
| AdaBoost+SVM+CatBoost | 31.857 | Rank 11 | 0.726 (0.705-0.746) | 0.380 (0.319-0.436) | 0.731 (0.684-0.776) | 0.594 (0.511-0.675) | 0.737 (0.715-0.758) | 0.185 (0.133-0.238) | 0.178 (0.169-0.188) |
| RF+CatBoost | 32.286 | Rank 12 | 0.720 (0.697-0.741) | 0.397 (0.331-0.455) | 0.750 (0.710-0.793) | 0.637 (0.553-0.727) | 0.726 (0.703-0.750) | 0.195 (0.137-0.253) | 0.176 (0.166-0.186) |
| AdaBoost+DT+RF+LightGBM | 34.286 | Rank 13 | 0.729 (0.709-0.751) | 0.349 (0.285-0.408) | 0.720 (0.675-0.765) | 0.536 (0.447-0.634) | 0.745 (0.723-0.768) | 0.195 (0.133-0.252) | 0.175 (0.165-0.183) |
| DT+RF+SVM | 37.000 | Rank 14 | 0.758 (0.737-0.780) | 0.341 (0.271-0.410) | 0.687 (0.638-0.744) | 0.491 (0.400-0.590) | 0.780 (0.757-0.802) | 0.198 (0.129-0.262) | 0.164 (0.155-0.174) |
| DT+RF+SVM+XGBoost | 37.429 | Rank 15 | 0.727 (0.706-0.750) | 0.365 (0.297-0.425) | 0.702 (0.654-0.748) | 0.566 (0.465-0.653) | 0.740 (0.718-0.764) | 0.171 (0.118-0.229) | 0.174 (0.164-0.184) |
| AdaBoost+DT+RF+LightGBM+SVM+CatBoost | 38.429 | Rank 16 | 0.729 (0.707-0.752) | 0.380 (0.318-0.441) | 0.709 (0.661-0.757) | 0.593 (0.510-0.684) | 0.740 (0.718-0.762) | 0.165 (0.119-0.220) | 0.182 (0.171-0.192) |
| RF+SVM+XGBoost | 38.714 | Rank 17 | 0.726 (0.703-0.750) | 0.372 (0.305-0.431) | 0.696 (0.644-0.746) | 0.579 (0.488-0.667) | 0.738 (0.715-0.764) | 0.190 (0.130-0.244) | 0.180 (0.170-0.191) |
| AdaBoost+DT+RF+CatBoost | 38.857 | Rank 18 | 0.751 (0.730-0.773) | 0.337 (0.271-0.396) | 0.702 (0.653-0.750) | 0.492 (0.400-0.583) | 0.772 (0.751-0.795) | 0.179 (0.125-0.230) | 0.166 (0.157-0.175) |
| AdaBoost+RF+LightGBM+XGBoost+CatBoost | 39.000 | Rank 19 | 0.748 (0.729-0.769) | 0.344 (0.276-0.405) | 0.714 (0.668-0.759) | 0.507 (0.421-0.595) | 0.768 (0.748-0.789) | 0.159 (0.115-0.206) | 0.168 (0.157-0.177) |
| AdaBoost+RF+XGBoost | 39.000 | Rank 19 | 0.755 (0.735-0.776) | 0.367 (0.295-0.429) | 0.685 (0.637-0.731) | 0.537 (0.444-0.628) | 0.773 (0.752-0.795) | 0.147 (0.106-0.196) | 0.170 (0.161-0.180) |
| AdaBoost+LightGBM+SVM | 39.714 | Rank 21 | 0.722 (0.700-0.744) | 0.385 (0.321-0.440) | 0.707 (0.656-0.759) | 0.609 (0.522-0.698) | 0.731 (0.708-0.754) | 0.196 (0.133-0.254) | 0.186 (0.175-0.197) |
| DT+RF+LightGBM+CatBoost | 39.714 | Rank 21 | 0.729 (0.708-0.750) | 0.343 (0.279-0.402) | 0.712 (0.668-0.758) | 0.524 (0.439-0.619) | 0.746 (0.724-0.768) | 0.184 (0.124-0.241) | 0.171 (0.162-0.180) |
| DT+RF+XGBoost | 39.714 | Rank 21 | 0.718 (0.697-0.739) | 0.366 (0.303-0.427) | 0.715 (0.666-0.761) | 0.579 (0.489-0.667) | 0.729 (0.709-0.751) | 0.197 (0.129-0.257) | 0.180 (0.170-0.190) |
| LightGBM+SVM+XGBoost | 41.143 | Rank 24 | 0.731 (0.708-0.752) | 0.352 (0.291-0.411) | 0.706 (0.661-0.753) | 0.538 (0.452-0.630) | 0.746 (0.723-0.768) | 0.173 (0.118-0.225) | 0.179 (0.170-0.189) |
| DT+RF+LightGBM+SVM+CatBoost | 41.714 | Rank 25 | 0.736 (0.714-0.759) | 0.353 (0.284-0.414) | 0.689 (0.640-0.746) | 0.536 (0.437-0.628) | 0.752 (0.730-0.774) | 0.176 (0.116-0.233) | 0.175 (0.163-0.186) |
| AdaBoost+LightGBM+SVM+CatBoost | 43.143 | Rank 26 | 0.730 (0.708-0.753) | 0.358 (0.288-0.420) | 0.698 (0.646-0.749) | 0.550 (0.462-0.640) | 0.745 (0.723-0.768) | 0.174 (0.121-0.237) | 0.181 (0.170-0.191) |
| AdaBoost+DT+RF+XGBoost | 43.714 | Rank 27 | 0.748 (0.727-0.770) | 0.335 (0.272-0.398) | 0.703 (0.658-0.751) | 0.492 (0.408-0.585) | 0.769 (0.747-0.790) | 0.169 (0.119-0.231) | 0.170 (0.161-0.179) |
| RF | 44.143 | Rank 28 | 0.753 (0.732-0.774) | 0.322 (0.255-0.385) | 0.699 (0.650-0.749) | 0.464 (0.370-0.564) | 0.777 (0.756-0.797) | 0.195 (0.126-0.271) | 0.175 (0.168-0.183) |
| XGBoost+CatBoost | 44.286 | Rank 29 | 0.738 (0.717-0.759) | 0.346 (0.278-0.402) | 0.701 (0.654-0.749) | 0.520 (0.428-0.605) | 0.756 (0.735-0.777) | 0.162 (0.114-0.215) | 0.174 (0.164-0.183) |
| RF+LightGBM+SVM+XGBoost | 44.429 | Rank 30 | 0.724 (0.702-0.747) | 0.378 (0.311-0.441) | 0.705 (0.655-0.756) | 0.595 (0.511-0.690) | 0.735 (0.712-0.758) | 0.172 (0.120-0.226) | 0.185 (0.174-0.196) |
| DT+RF+LightGBM+XGBoost+CatBoost | 44.857 | Rank 31 | 0.741 (0.720-0.762) | 0.341 (0.272-0.402) | 0.684 (0.634-0.738) | 0.508 (0.415-0.600) | 0.760 (0.739-0.782) | 0.183 (0.123-0.244) | 0.170 (0.160-0.180) |
| AdaBoost+DT+RF+XGBoost+CatBoost | 45.000 | Rank 32 | 0.727 (0.706-0.749) | 0.354 (0.291-0.412) | 0.715 (0.672-0.761) | 0.548 (0.463-0.639) | 0.742 (0.719-0.766) | 0.154 (0.115-0.195) | 0.177 (0.168-0.188) |
| DT+RF+LightGBM+XGBoost | 45.429 | Rank 33 | 0.774 (0.753-0.794) | 0.343 (0.280-0.405) | 0.686 (0.638-0.740) | 0.478 (0.390-0.565) | 0.798 (0.778-0.818) | 0.156 (0.111-0.205) | 0.161 (0.152-0.169) |
| AdaBoost+DT+SVM+XGBoost | 45.571 | Rank 34 | 0.722 (0.699-0.744) | 0.345 (0.277-0.404) | 0.707 (0.656-0.760) | 0.535 (0.447-0.625) | 0.738 (0.714-0.760) | 0.213 (0.141-0.280) | 0.180 (0.170-0.190) |
| RF+SVM+CatBoost | 45.571 | Rank 34 | 0.746 (0.725-0.768) | 0.353 (0.284-0.419) | 0.688 (0.636-0.742) | 0.524 (0.432-0.620) | 0.765 (0.744-0.786) | 0.145 (0.107-0.186) | 0.169 (0.158-0.180) |
| AdaBoost+DT+RF+SVM+CatBoost | 46.429 | Rank 36 | 0.750 (0.728-0.772) | 0.338 (0.277-0.401) | 0.694 (0.648-0.744) | 0.494 (0.400-0.591) | 0.771 (0.750-0.791) | 0.158 (0.108-0.204) | 0.168 (0.157-0.178) |
| AdaBoost+DT+CatBoost | 46.571 | Rank 37 | 0.751 (0.731-0.772) | 0.348 (0.282-0.408) | 0.680 (0.627-0.734) | 0.508 (0.419-0.600) | 0.771 (0.750-0.792) | 0.161 (0.110-0.208) | 0.171 (0.162-0.180) |
| RF+LightGBM+SVM | 47.000 | Rank 38 | 0.725 (0.705-0.747) | 0.356 (0.294-0.414) | 0.688 (0.635-0.742) | 0.552 (0.463-0.643) | 0.740 (0.719-0.762) | 0.191 (0.127-0.258) | 0.182 (0.172-0.191) |
| AdaBoost+XGBoost+CatBoost | 49.571 | Rank 39 | 0.721 (0.701-0.743) | 0.351 (0.286-0.410) | 0.706 (0.654-0.758) | 0.550 (0.458-0.649) | 0.735 (0.714-0.758) | 0.182 (0.129-0.241) | 0.183 (0.173-0.192) |
| DT+XGBoost+CatBoost | 49.714 | Rank 40 | 0.727 (0.705-0.751) | 0.356 (0.294-0.417) | 0.695 (0.642-0.749) | 0.550 (0.465-0.639) | 0.741 (0.718-0.766) | 0.152 (0.113-0.194) | 0.176 (0.165-0.186) |
| RF+LightGBM+XGBoost+CatBoost | 49.714 | Rank 40 | 0.727 (0.705-0.749) | 0.323 (0.261-0.381) | 0.717 (0.672-0.760) | 0.492 (0.404-0.578) | 0.746 (0.724-0.768) | 0.192 (0.133-0.249) | 0.175 (0.165-0.184) |
| DT+LightGBM+SVM+XGBoost | 50.571 | Rank 42 | 0.717 (0.697-0.740) | 0.359 (0.297-0.419) | 0.701 (0.652-0.752) | 0.567 (0.478-0.659) | 0.730 (0.709-0.753) | 0.185 (0.129-0.246) | 0.185 (0.174-0.195) |
| RF+XGBoost | 50.857 | Rank 43 | 0.745 (0.723-0.768) | 0.316 (0.251-0.383) | 0.692 (0.648-0.738) | 0.463 (0.375-0.560) | 0.768 (0.745-0.790) | 0.179 (0.120-0.240) | 0.166 (0.156-0.175) |
| AdaBoost+DT+SVM+CatBoost | 51.714 | Rank 44 | 0.729 (0.708-0.751) | 0.382 (0.322-0.438) | 0.690 (0.643-0.741) | 0.594 (0.500-0.684) | 0.741 (0.719-0.763) | 0.152 (0.109-0.205) | 0.188 (0.178-0.198) |
| DT+SVM+XGBoost+CatBoost | 51.714 | Rank 44 | 0.750 (0.728-0.773) | 0.344 (0.272-0.408) | 0.683 (0.631-0.736) | 0.506 (0.408-0.592) | 0.769 (0.749-0.791) | 0.148 (0.105-0.194) | 0.169 (0.159-0.179) |
| RF+LightGBM+CatBoost | 52.429 | Rank 46 | 0.723 (0.701-0.744) | 0.355 (0.289-0.413) | 0.692 (0.643-0.745) | 0.551 (0.459-0.645) | 0.737 (0.715-0.760) | 0.169 (0.113-0.217) | 0.181 (0.171-0.192) |
| AdaBoost+RF | 52.857 | Rank 47 | 0.716 (0.695-0.739) | 0.351 (0.286-0.406) | 0.693 (0.641-0.742) | 0.553 (0.463-0.636) | 0.730 (0.707-0.752) | 0.191 (0.129-0.250) | 0.181 (0.171-0.191) |
| SVM+XGBoost | 53.000 | Rank 48 | 0.739 (0.717-0.759) | 0.348 (0.286-0.407) | 0.697 (0.651-0.745) | 0.522 (0.429-0.614) | 0.757 (0.734-0.778) | 0.144 (0.108-0.178) | 0.178 (0.169-0.188) |
| AdaBoost+RF+SVM+CatBoost | 54.000 | Rank 49 | 0.724 (0.701-0.745) | 0.345 (0.279-0.405) | 0.687 (0.638-0.733) | 0.536 (0.450-0.622) | 0.740 (0.716-0.762) | 0.186 (0.120-0.245) | 0.182 (0.172-0.193) |
| DT+RF | 54.000 | Rank 49 | 0.745 (0.724-0.765) | 0.334 (0.265-0.396) | 0.698 (0.648-0.748) | 0.494 (0.395-0.581) | 0.765 (0.744-0.787) | 0.150 (0.106-0.195) | 0.175 (0.165-0.185) |
| RF+SVM+XGBoost+CatBoost | 54.571 | Rank 51 | 0.714 (0.691-0.735) | 0.341 (0.272-0.402) | 0.695 (0.647-0.745) | 0.537 (0.444-0.630) | 0.728 (0.706-0.750) | 0.208 (0.141-0.267) | 0.177 (0.167-0.187) |
| AdaBoost+LightGBM+CatBoost | 55.286 | Rank 52 | 0.742 (0.721-0.763) | 0.349 (0.284-0.406) | 0.685 (0.640-0.733) | 0.521 (0.436-0.611) | 0.760 (0.738-0.782) | 0.132 (0.101-0.165) | 0.174 (0.165-0.183) |
| RF+SVM | 55.571 | Rank 53 | 0.709 (0.686-0.732) | 0.370 (0.299-0.431) | 0.705 (0.657-0.755) | 0.596 (0.510-0.688) | 0.719 (0.696-0.741) | 0.176 (0.123-0.235) | 0.188 (0.177-0.198) |
| AdaBoost+SVM+XGBoost+CatBoost | 56.429 | Rank 54 | 0.752 (0.732-0.772) | 0.338 (0.268-0.393) | 0.683 (0.638-0.730) | 0.490 (0.395-0.581) | 0.774 (0.753-0.796) | 0.142 (0.104-0.183) | 0.170 (0.160-0.180) |
| AdaBoost+DT+LightGBM+SVM+CatBoost | 60.429 | Rank 55 | 0.725 (0.703-0.746) | 0.363 (0.295-0.425) | 0.675 (0.621-0.731) | 0.564 (0.471-0.656) | 0.738 (0.716-0.760) | 0.159 (0.112-0.213) | 0.185 (0.173-0.196) |
| RF+LightGBM+SVM+XGBoost+CatBoost | 60.857 | Rank 56 | 0.730 (0.708-0.750) | 0.334 (0.264-0.392) | 0.675 (0.622-0.728) | 0.508 (0.413-0.600) | 0.748 (0.726-0.769) | 0.165 (0.115-0.221) | 0.174 (0.164-0.183) |
| RF+LightGBM | 61.429 | Rank 57 | 0.718 (0.695-0.740) | 0.382 (0.323-0.439) | 0.683 (0.638-0.729) | 0.608 (0.525-0.696) | 0.727 (0.704-0.750) | 0.158 (0.109-0.202) | 0.186 (0.175-0.197) |
| AdaBoost+DT+LightGBM | 62.571 | Rank 58 | 0.700 (0.677-0.723) | 0.377 (0.312-0.438) | 0.719 (0.667-0.768) | 0.622 (0.524-0.717) | 0.706 (0.684-0.729) | 0.162 (0.117-0.208) | 0.201 (0.191-0.211) |
| AdaBoost+DT+RF+LightGBM+SVM | 63.429 | Rank 59 | 0.701 (0.677-0.725) | 0.350 (0.288-0.410) | 0.712 (0.666-0.760) | 0.567 (0.477-0.651) | 0.712 (0.689-0.736) | 0.169 (0.120-0.221) | 0.186 (0.176-0.197) |
| DT+LightGBM+SVM+XGBoost+CatBoost | 63.429 | Rank 59 | 0.717 (0.694-0.740) | 0.366 (0.301-0.425) | 0.679 (0.629-0.734) | 0.579 (0.488-0.674) | 0.728 (0.704-0.751) | 0.149 (0.112-0.189) | 0.182 (0.171-0.192) |
| AdaBoost+RF+LightGBM+SVM+CatBoost | 63.571 | Rank 61 | 0.731 (0.709-0.752) | 0.349 (0.284-0.410) | 0.672 (0.623-0.728) | 0.535 (0.450-0.628) | 0.747 (0.725-0.768) | 0.137 (0.102-0.174) | 0.178 (0.169-0.188) |
| DT+RF+SVM+CatBoost | 63.571 | Rank 61 | 0.726 (0.703-0.748) | 0.333 (0.268-0.390) | 0.687 (0.636-0.736) | 0.509 (0.419-0.600) | 0.744 (0.722-0.765) | 0.165 (0.116-0.220) | 0.180 (0.170-0.190) |
| DT+LightGBM+CatBoost | 63.714 | Rank 63 | 0.729 (0.708-0.752) | 0.341 (0.280-0.402) | 0.680 (0.631-0.730) | 0.522 (0.432-0.615) | 0.746 (0.726-0.770) | 0.159 (0.110-0.211) | 0.184 (0.173-0.195) |
| AdaBoost+DT+RF+LightGBM+XGBoost | 64.286 | Rank 64 | 0.739 (0.716-0.762) | 0.296 (0.228-0.355) | 0.698 (0.653-0.742) | 0.434 (0.333-0.525) | 0.764 (0.742-0.785) | 0.144 (0.105-0.187) | 0.169 (0.157-0.179) |
| DT+CatBoost | 66.286 | Rank 65 | 0.734 (0.711-0.756) | 0.337 (0.270-0.399) | 0.683 (0.634-0.733) | 0.507 (0.415-0.600) | 0.752 (0.729-0.774) | 0.144 (0.103-0.192) | 0.177 (0.168-0.187) |
| AdaBoost+DT+RF+LightGBM+SVM+XGBoost | 67.286 | Rank 66 | 0.721 (0.700-0.743) | 0.337 (0.274-0.397) | 0.659 (0.609-0.716) | 0.521 (0.432-0.615) | 0.738 (0.715-0.760) | 0.192 (0.122-0.252) | 0.182 (0.172-0.192) |
| DT+RF+CatBoost | 68.286 | Rank 67 | 0.717 (0.695-0.739) | 0.324 (0.262-0.386) | 0.698 (0.650-0.751) | 0.505 (0.419-0.600) | 0.734 (0.712-0.756) | 0.187 (0.126-0.251) | 0.184 (0.172-0.195) |
| DT+SVM+CatBoost | 68.571 | Rank 68 | 0.722 (0.700-0.746) | 0.330 (0.263-0.390) | 0.693 (0.647-0.743) | 0.507 (0.408-0.600) | 0.740 (0.718-0.763) | 0.153 (0.110-0.200) | 0.177 (0.166-0.187) |
| AdaBoost+SVM | 69.000 | Rank 69 | 0.719 (0.695-0.742) | 0.353 (0.291-0.409) | 0.691 (0.643-0.740) | 0.554 (0.465-0.644) | 0.732 (0.709-0.756) | 0.146 (0.108-0.187) | 0.192 (0.181-0.202) |
| LightGBM+XGBoost+CatBoost | 69.000 | Rank 69 | 0.719 (0.697-0.741) | 0.345 (0.279-0.405) | 0.661 (0.607-0.716) | 0.539 (0.447-0.634) | 0.734 (0.712-0.755) | 0.183 (0.122-0.242) | 0.186 (0.175-0.196) |
| LightGBM+CatBoost | 69.857 | Rank 71 | 0.719 (0.698-0.741) | 0.343 (0.281-0.402) | 0.653 (0.597-0.711) | 0.536 (0.447-0.636) | 0.734 (0.712-0.756) | 0.172 (0.108-0.227) | 0.182 (0.171-0.193) |
| LightGBM+SVM | 70.714 | Rank 72 | 0.704 (0.682-0.727) | 0.352 (0.287-0.409) | 0.683 (0.630-0.736) | 0.568 (0.476-0.659) | 0.715 (0.692-0.738) | 0.173 (0.115-0.237) | 0.192 (0.182-0.201) |
| AdaBoost+DT+RF+SVM | 71.286 | Rank 73 | 0.700 (0.679-0.722) | 0.333 (0.272-0.391) | 0.702 (0.658-0.750) | 0.538 (0.447-0.628) | 0.714 (0.690-0.737) | 0.182 (0.127-0.252) | 0.185 (0.176-0.195) |
| AdaBoost+RF+LightGBM+SVM+XGBoost | 71.571 | Rank 74 | 0.722 (0.701-0.743) | 0.304 (0.238-0.362) | 0.678 (0.625-0.729) | 0.463 (0.375-0.556) | 0.743 (0.721-0.765) | 0.187 (0.129-0.250) | 0.176 (0.168-0.184) |
| DT+LightGBM+XGBoost | 72.857 | Rank 75 | 0.699 (0.676-0.720) | 0.363 (0.297-0.420) | 0.696 (0.648-0.745) | 0.594 (0.500-0.684) | 0.708 (0.685-0.730) | 0.153 (0.109-0.201) | 0.199 (0.190-0.209) |
| DT+RF+LightGBM | 73.571 | Rank 76 | 0.712 (0.688-0.734) | 0.323 (0.257-0.382) | 0.696 (0.649-0.740) | 0.508 (0.413-0.600) | 0.729 (0.705-0.752) | 0.176 (0.119-0.235) | 0.184 (0.174-0.195) |
| NaiveBayes | 74.143 | Rank 77 | 0.833 (0.815-0.850) | 0.215 (0.146-0.280) | 0.577 (0.515-0.638) | 0.246 (0.167-0.326) | 0.881 (0.864-0.898) | 0.105 (0.078-0.130) | 0.158 (0.142-0.173) |
| AdaBoost | 74.286 | Rank 78 | 0.674 (0.651-0.701) | 0.385 (0.326-0.439) | 0.708 (0.665-0.754) | 0.668 (0.578-0.762) | 0.675 (0.651-0.702) | 0.133 (0.103-0.163) | 0.206 (0.198-0.214) |
| AdaBoost+DT+RF+SVM+XGBoost+CatBoost | 74.286 | Rank 78 | 0.747 (0.725-0.767) | 0.300 (0.235-0.364) | 0.641 (0.586-0.696) | 0.434 (0.341-0.528) | 0.772 (0.751-0.794) | 0.139 (0.100-0.183) | 0.169 (0.159-0.179) |
| AdaBoost+DT+RF | 74.714 | Rank 80 | 0.717 (0.697-0.741) | 0.327 (0.265-0.388) | 0.691 (0.643-0.741) | 0.508 (0.413-0.600) | 0.734 (0.713-0.758) | 0.160 (0.112-0.215) | 0.184 (0.173-0.193) |
| AdaBoost+RF+LightGBM+CatBoost | 74.857 | Rank 81 | 0.698 (0.675-0.720) | 0.348 (0.284-0.407) | 0.704 (0.661-0.751) | 0.566 (0.474-0.659) | 0.709 (0.685-0.731) | 0.147 (0.106-0.190) | 0.190 (0.179-0.201) |
| AdaBoost+RF+LightGBM+XGBoost | 75.143 | Rank 82 | 0.734 (0.713-0.755) | 0.311 (0.242-0.370) | 0.667 (0.617-0.719) | 0.465 (0.365-0.560) | 0.756 (0.734-0.779) | 0.153 (0.106-0.202) | 0.179 (0.169-0.188) |
| LightGBM+SVM+CatBoost | 76.286 | Rank 83 | 0.723 (0.701-0.744) | 0.331 (0.270-0.390) | 0.673 (0.626-0.725) | 0.510 (0.422-0.596) | 0.740 (0.719-0.761) | 0.151 (0.108-0.199) | 0.183 (0.172-0.193) |
| AdaBoost+DT+SVM+XGBoost+CatBoost | 76.571 | Rank 84 | 0.699 (0.676-0.721) | 0.347 (0.280-0.409) | 0.689 (0.638-0.738) | 0.564 (0.474-0.659) | 0.710 (0.687-0.733) | 0.159 (0.114-0.208) | 0.186 (0.176-0.197) |
| DT+RF+LightGBM+SVM | 76.857 | Rank 85 | 0.701 (0.678-0.723) | 0.341 (0.274-0.399) | 0.693 (0.646-0.742) | 0.551 (0.455-0.647) | 0.713 (0.690-0.737) | 0.167 (0.115-0.227) | 0.193 (0.182-0.204) |
| DT+RF+LightGBM+SVM+XGBoost | 77.286 | Rank 86 | 0.707 (0.686-0.730) | 0.336 (0.278-0.391) | 0.676 (0.629-0.725) | 0.535 (0.444-0.622) | 0.722 (0.699-0.744) | 0.180 (0.121-0.236) | 0.185 (0.174-0.195) |
| AdaBoost+DT+RF+LightGBM+CatBoost | 77.857 | Rank 87 | 0.726 (0.704-0.750) | 0.322 (0.258-0.381) | 0.677 (0.630-0.726) | 0.491 (0.404-0.585) | 0.745 (0.723-0.770) | 0.140 (0.103-0.180) | 0.177 (0.167-0.187) |
| AdaBoost+RF+LightGBM | 78.429 | Rank 88 | 0.713 (0.692-0.736) | 0.338 (0.278-0.396) | 0.684 (0.632-0.738) | 0.534 (0.442-0.625) | 0.728 (0.706-0.751) | 0.162 (0.113-0.216) | 0.191 (0.180-0.202) |
| AdaBoost+RF+CatBoost | 80.143 | Rank 89 | 0.729 (0.707-0.752) | 0.307 (0.239-0.363) | 0.657 (0.606-0.711) | 0.463 (0.368-0.553) | 0.750 (0.729-0.773) | 0.147 (0.098-0.202) | 0.175 (0.165-0.186) |
| AdaBoost+DT+RF+LightGBM+XGBoost+CatBoost | 80.286 | Rank 90 | 0.727 (0.705-0.749) | 0.331 (0.267-0.392) | 0.669 (0.620-0.721) | 0.507 (0.419-0.595) | 0.745 (0.724-0.767) | 0.132 (0.097-0.169) | 0.183 (0.172-0.193) |
| RF+LightGBM+XGBoost | 80.429 | Rank 91 | 0.734 (0.712-0.756) | 0.302 (0.236-0.360) | 0.668 (0.619-0.715) | 0.448 (0.357-0.538) | 0.758 (0.736-0.780) | 0.144 (0.100-0.191) | 0.179 (0.169-0.188) |
| XGBoost | 80.571 | Rank 92 | 0.688 (0.666-0.710) | 0.335 (0.275-0.393) | 0.695 (0.647-0.742) | 0.553 (0.465-0.650) | 0.699 (0.678-0.723) | 0.176 (0.122-0.227) | 0.239 (0.222-0.256) |
| SVM | 80.857 | Rank 93 | 0.661 (0.637-0.684) | 0.365 (0.311-0.418) | 0.681 (0.628-0.732) | 0.640 (0.553-0.721) | 0.662 (0.637-0.688) | 0.158 (0.115-0.205) | 0.209 (0.200-0.219) |
| AdaBoost+LightGBM+XGBoost+CatBoost | 81.571 | Rank 94 | 0.736 (0.714-0.759) | 0.303 (0.239-0.365) | 0.649 (0.598-0.702) | 0.449 (0.364-0.538) | 0.759 (0.738-0.782) | 0.133 (0.095-0.179) | 0.175 (0.163-0.186) |
| AdaBoost+DT+RF+LightGBM+SVM+XGBoost+CatBoost | 82.571 | Rank 95 | 0.726 (0.704-0.747) | 0.298 (0.234-0.355) | 0.673 (0.629-0.721) | 0.450 (0.359-0.545) | 0.748 (0.726-0.770) | 0.161 (0.110-0.214) | 0.184 (0.173-0.196) |
| LightGBM+XGBoost | 85.143 | Rank 96 | 0.689 (0.665-0.713) | 0.328 (0.267-0.388) | 0.687 (0.639-0.738) | 0.540 (0.450-0.634) | 0.701 (0.676-0.726) | 0.174 (0.115-0.229) | 0.195 (0.185-0.206) |
| DT | 85.429 | Rank 97 | 0.679 (0.656-0.701) | 0.343 (0.278-0.399) | 0.689 (0.641-0.736) | 0.579 (0.486-0.667) | 0.687 (0.663-0.711) | 0.152 (0.107-0.198) | 0.214 (0.203-0.226) |
| AdaBoost+SVM+XGBoost | 85.571 | Rank 98 | 0.721 (0.698-0.741) | 0.327 (0.266-0.386) | 0.677 (0.630-0.723) | 0.504 (0.415-0.591) | 0.738 (0.716-0.760) | 0.145 (0.105-0.191) | 0.186 (0.175-0.196) |
| DT+LightGBM+SVM | 85.571 | Rank 98 | 0.715 (0.692-0.737) | 0.350 (0.282-0.409) | 0.666 (0.613-0.714) | 0.552 (0.458-0.644) | 0.728 (0.705-0.750) | 0.122 (0.092-0.151) | 0.193 (0.182-0.204) |
| DT+LightGBM+SVM+CatBoost | 86.429 | Rank 100 | 0.710 (0.687-0.732) | 0.337 (0.274-0.392) | 0.666 (0.611-0.722) | 0.534 (0.442-0.622) | 0.724 (0.703-0.746) | 0.160 (0.114-0.210) | 0.188 (0.177-0.199) |
| DT+RF+LightGBM+SVM+XGBoost+CatBoost | 86.429 | Rank 100 | 0.685 (0.662-0.708) | 0.311 (0.251-0.368) | 0.692 (0.639-0.739) | 0.510 (0.425-0.600) | 0.699 (0.675-0.723) | 0.202 (0.137-0.273) | 0.193 (0.182-0.203) |
| AdaBoost+RF+SVM | 86.571 | Rank 102 | 0.705 (0.683-0.728) | 0.334 (0.265-0.391) | 0.685 (0.635-0.732) | 0.535 (0.439-0.622) | 0.718 (0.695-0.741) | 0.144 (0.107-0.185) | 0.185 (0.176-0.195) |
| DT+SVM+XGBoost | 86.857 | Rank 103 | 0.708 (0.684-0.730) | 0.321 (0.259-0.380) | 0.671 (0.618-0.723) | 0.506 (0.415-0.605) | 0.724 (0.702-0.747) | 0.170 (0.115-0.227) | 0.183 (0.173-0.193) |
| AdaBoost+RF+LightGBM+SVM+XGBoost+CatBoost | 89.571 | Rank 104 | 0.704 (0.682-0.727) | 0.328 (0.266-0.387) | 0.682 (0.630-0.732) | 0.523 (0.429-0.615) | 0.719 (0.696-0.742) | 0.153 (0.109-0.207) | 0.192 (0.180-0.203) |
| AdaBoost+DT+XGBoost | 90.714 | Rank 105 | 0.703 (0.680-0.727) | 0.318 (0.253-0.377) | 0.695 (0.645-0.743) | 0.508 (0.419-0.595) | 0.719 (0.694-0.742) | 0.149 (0.106-0.198) | 0.191 (0.182-0.200) |
| AdaBoost+LightGBM | 91.000 | Rank 106 | 0.713 (0.692-0.737) | 0.340 (0.275-0.400) | 0.670 (0.622-0.721) | 0.535 (0.447-0.630) | 0.728 (0.706-0.752) | 0.136 (0.098-0.177) | 0.197 (0.187-0.206) |
| AdaBoost+DT+LightGBM+XGBoost | 92.571 | Rank 107 | 0.691 (0.668-0.714) | 0.321 (0.255-0.380) | 0.695 (0.645-0.748) | 0.522 (0.428-0.615) | 0.705 (0.683-0.728) | 0.159 (0.115-0.203) | 0.204 (0.193-0.216) |
| AdaBoost+DT+XGBoost+CatBoost | 92.714 | Rank 108 | 0.713 (0.689-0.735) | 0.315 (0.251-0.374) | 0.672 (0.620-0.724) | 0.492 (0.400-0.583) | 0.730 (0.706-0.753) | 0.143 (0.101-0.192) | 0.182 (0.172-0.193) |
| DT+LightGBM | 92.857 | Rank 109 | 0.699 (0.677-0.722) | 0.323 (0.256-0.380) | 0.673 (0.620-0.726) | 0.520 (0.429-0.609) | 0.714 (0.692-0.738) | 0.166 (0.115-0.222) | 0.195 (0.187-0.203) |
| AdaBoost+CatBoost | 94.143 | Rank 110 | 0.712 (0.689-0.733) | 0.330 (0.266-0.386) | 0.660 (0.612-0.709) | 0.521 (0.429-0.614) | 0.727 (0.705-0.750) | 0.149 (0.102-0.196) | 0.195 (0.184-0.206) |
| RF+XGBoost+CatBoost | 95.286 | Rank 111 | 0.702 (0.680-0.722) | 0.287 (0.223-0.344) | 0.674 (0.627-0.719) | 0.452 (0.356-0.545) | 0.723 (0.701-0.744) | 0.171 (0.109-0.231) | 0.184 (0.173-0.195) |
| AdaBoost+LightGBM+XGBoost | 95.571 | Rank 112 | 0.714 (0.692-0.736) | 0.301 (0.236-0.359) | 0.679 (0.634-0.725) | 0.464 (0.368-0.556) | 0.734 (0.712-0.757) | 0.146 (0.107-0.191) | 0.186 (0.176-0.196) |
| SVM+XGBoost+CatBoost | 95.571 | Rank 112 | 0.712 (0.689-0.736) | 0.300 (0.231-0.361) | 0.671 (0.621-0.720) | 0.464 (0.370-0.556) | 0.732 (0.709-0.755) | 0.146 (0.103-0.194) | 0.183 (0.173-0.193) |
| AdaBoost+DT+LightGBM+XGBoost+CatBoost | 96.000 | Rank 114 | 0.725 (0.704-0.747) | 0.288 (0.226-0.346) | 0.640 (0.591-0.689) | 0.433 (0.342-0.522) | 0.749 (0.727-0.771) | 0.122 (0.092-0.156) | 0.183 (0.172-0.193) |
| AdaBoost+DT+LightGBM+SVM+XGBoost | 96.143 | Rank 115 | 0.724 (0.702-0.746) | 0.294 (0.230-0.353) | 0.655 (0.604-0.707) | 0.444 (0.351-0.535) | 0.747 (0.725-0.770) | 0.140 (0.099-0.185) | 0.186 (0.175-0.196) |
| DT+SVM | 96.429 | Rank 116 | 0.694 (0.671-0.716) | 0.322 (0.259-0.377) | 0.666 (0.616-0.717) | 0.523 (0.435-0.609) | 0.708 (0.684-0.731) | 0.167 (0.109-0.222) | 0.196 (0.187-0.205) |
| AdaBoost+LightGBM+SVM+XGBoost+CatBoost | 97.000 | Rank 117 | 0.717 (0.694-0.740) | 0.318 (0.258-0.378) | 0.664 (0.613-0.714) | 0.492 (0.405-0.583) | 0.736 (0.713-0.758) | 0.129 (0.094-0.167) | 0.186 (0.175-0.198) |
| AdaBoost+XGBoost | 98.143 | Rank 118 | 0.682 (0.660-0.704) | 0.315 (0.255-0.372) | 0.683 (0.633-0.731) | 0.521 (0.429-0.610) | 0.695 (0.673-0.718) | 0.166 (0.112-0.213) | 0.202 (0.193-0.211) |
| AdaBoost+DT | 98.714 | Rank 119 | 0.649 (0.626-0.673) | 0.343 (0.279-0.398) | 0.661 (0.611-0.713) | 0.609 (0.524-0.696) | 0.652 (0.628-0.677) | 0.125 (0.093-0.158) | 0.223 (0.216-0.231) |
| CatBoost | 98.857 | Rank 120 | 0.706 (0.684-0.730) | 0.320 (0.255-0.379) | 0.665 (0.615-0.719) | 0.509 (0.417-0.605) | 0.722 (0.700-0.747) | 0.126 (0.093-0.160) | 0.184 (0.174-0.194) |
| AdaBoost+DT+SVM | 99.143 | Rank 121 | 0.698 (0.676-0.720) | 0.324 (0.262-0.384) | 0.669 (0.619-0.721) | 0.523 (0.436-0.622) | 0.713 (0.689-0.736) | 0.149 (0.105-0.201) | 0.198 (0.188-0.208) |
| DT+RF+SVM+XGBoost+CatBoost | 100.143 | Rank 122 | 0.707 (0.686-0.730) | 0.279 (0.216-0.341) | 0.684 (0.644-0.727) | 0.433 (0.341-0.523) | 0.730 (0.708-0.752) | 0.138 (0.096-0.176) | 0.184 (0.174-0.195) |
| AdaBoost+RF+SVM+XGBoost | 101.000 | Rank 123 | 0.708 (0.685-0.731) | 0.305 (0.239-0.361) | 0.670 (0.621-0.717) | 0.478 (0.386-0.567) | 0.727 (0.704-0.750) | 0.147 (0.106-0.193) | 0.187 (0.176-0.198) |
| AdaBoost+RF+LightGBM+SVM | 103.714 | Rank 124 | 0.690 (0.667-0.714) | 0.313 (0.249-0.366) | 0.672 (0.625-0.718) | 0.511 (0.419-0.595) | 0.705 (0.682-0.730) | 0.134 (0.100-0.172) | 0.185 (0.175-0.196) |
| AdaBoost+DT+LightGBM+SVM | 107.571 | Rank 125 | 0.703 (0.681-0.724) | 0.311 (0.246-0.371) | 0.649 (0.598-0.701) | 0.495 (0.400-0.581) | 0.720 (0.697-0.742) | 0.135 (0.091-0.173) | 0.189 (0.181-0.198) |
| AdaBoost+LightGBM+SVM+XGBoost | 108.000 | Rank 126 | 0.695 (0.672-0.718) | 0.299 (0.233-0.357) | 0.650 (0.594-0.705) | 0.479 (0.386-0.580) | 0.713 (0.690-0.737) | 0.164 (0.104-0.217) | 0.199 (0.188-0.209) |
| LightGBM | 110.000 | Rank 127 | 0.710 (0.688-0.733) | 0.291 (0.224-0.347) | 0.660 (0.608-0.705) | 0.452 (0.351-0.538) | 0.731 (0.708-0.754) | 0.137 (0.096-0.179) | 0.206 (0.192-0.219) |
| KNN | 112.857 | Rank 128 | 0.660 (0.637-0.683) | 0.317 (0.254-0.375) | 0.629 (0.580-0.683) | 0.547 (0.457-0.636) | 0.669 (0.644-0.693) | 0.115 (0.086-0.146) | 0.255 (0.239-0.270) |
| DT+XGBoost | 121.143 | Rank 129 | 0.693 (0.670-0.716) | 0.274 (0.216-0.327) | 0.649 (0.605-0.693) | 0.434 (0.349-0.523) | 0.714 (0.691-0.736) | 0.125 (0.089-0.169) | 0.203 (0.195-0.212) |

**Supplementary Table 18.** Ranking of 129 machine learning models in the SHARE training set.

| **Model** | **Normalized Rank Score** | **Comprehensive Rank** | **Accuracy (95% CI)** | **F_Beta (95% CI)** | **ROC_AUC (95% CI)** | **Sensitivity (95% CI)** | **Specificity (95% CI)** | **PR_AUC (95% CI)** | **Brier (95% CI)** |
| --- | --- | --- | --- | --- | --- | --- | --- | --- | --- |
| AdaBoost+DT+RF+NaiveBayes+SVM+XGBoost | 6.714 | Rank 1 | 0.831 (0.813-0.848) | 0.767 (0.734-0.797) | 0.949 (0.939-0.958) | 1.000 (1.000-1.000) | 0.809 (0.790-0.828) | 0.638 (0.565-0.705) | 0.128 (0.120-0.137) |
| NaiveBayes+SVM+XGBoost+CatBoost | 7.714 | Rank 2 | 0.843 (0.826-0.859) | 0.775 (0.740-0.806) | 0.951 (0.941-0.961) | 0.991 (0.985-1.000) | 0.824 (0.807-0.843) | 0.660 (0.586-0.734) | 0.129 (0.121-0.135) |
| RF+NaiveBayes+SVM+XGBoost+CatBoost | 9.714 | Rank 3 | 0.829 (0.812-0.847) | 0.761 (0.731-0.792) | 0.953 (0.944-0.962) | 0.992 (0.985-1.000) | 0.808 (0.790-0.828) | 0.640 (0.566-0.715) | 0.130 (0.123-0.138) |
| RF+XGBoost+CatBoost | 10.000 | Rank 4 | 0.816 (0.798-0.834) | 0.753 (0.719-0.784) | 0.952 (0.942-0.961) | 1.000 (1.000-1.000) | 0.793 (0.773-0.812) | 0.683 (0.614-0.750) | 0.137 (0.130-0.145) |
| AdaBoost+RF+NaiveBayes+SVM+XGBoost+CatBoost | 10.286 | Rank 5 | 0.825 (0.808-0.842) | 0.757 (0.722-0.787) | 0.956 (0.946-0.966) | 0.992 (0.985-1.000) | 0.804 (0.785-0.822) | 0.644 (0.563-0.725) | 0.130 (0.123-0.138) |
| AdaBoost+DT+RF+SVM+XGBoost+CatBoost | 11.857 | Rank 6 | 0.845 (0.828-0.861) | 0.778 (0.744-0.807) | 0.943 (0.932-0.953) | 0.991 (0.985-1.000) | 0.826 (0.807-0.844) | 0.614 (0.538-0.690) | 0.121 (0.114-0.129) |
| XGBoost+CatBoost | 11.857 | Rank 6 | 0.813 (0.795-0.830) | 0.748 (0.716-0.776) | 0.958 (0.949-0.967) | 1.000 (1.000-1.000) | 0.789 (0.769-0.809) | 0.702 (0.632-0.762) | 0.136 (0.128-0.144) |
| AdaBoost+XGBoost | 14.000 | Rank 8 | 0.826 (0.808-0.844) | 0.762 (0.731-0.793) | 0.949 (0.940-0.958) | 1.000 (1.000-1.000) | 0.804 (0.784-0.824) | 0.569 (0.500-0.645) | 0.137 (0.129-0.145) |
| AdaBoost+NaiveBayes+XGBoost+CatBoost | 14.714 | Rank 9 | 0.817 (0.801-0.835) | 0.749 (0.716-0.781) | 0.946 (0.936-0.956) | 0.992 (0.985-1.000) | 0.795 (0.776-0.814) | 0.646 (0.580-0.709) | 0.132 (0.125-0.140) |
| DT+RF+NaiveBayes+SVM+XGBoost+CatBoost | 16.000 | Rank 10 | 0.815 (0.797-0.831) | 0.738 (0.701-0.770) | 0.952 (0.941-0.961) | 0.975 (0.958-1.000) | 0.795 (0.775-0.812) | 0.671 (0.601-0.744) | 0.129 (0.122-0.137) |
| DT+NaiveBayes+XGBoost+CatBoost | 18.286 | Rank 11 | 0.854 (0.838-0.870) | 0.737 (0.698-0.773) | 0.945 (0.935-0.955) | 0.900 (0.862-0.944) | 0.848 (0.831-0.866) | 0.657 (0.589-0.722) | 0.124 (0.118-0.130) |
| AdaBoost+DT+RF+SVM+XGBoost | 19.571 | Rank 12 | 0.807 (0.788-0.824) | 0.742 (0.708-0.773) | 0.948 (0.937-0.958) | 1.000 (1.000-1.000) | 0.783 (0.763-0.802) | 0.639 (0.570-0.699) | 0.138 (0.131-0.146) |
| AdaBoost+RF+XGBoost | 19.714 | Rank 13 | 0.822 (0.805-0.839) | 0.740 (0.702-0.776) | 0.948 (0.937-0.958) | 0.966 (0.944-0.988) | 0.804 (0.786-0.823) | 0.654 (0.580-0.719) | 0.139 (0.133-0.145) |
| DT+XGBoost+CatBoost | 20.143 | Rank 14 | 0.815 (0.797-0.833) | 0.750 (0.717-0.782) | 0.943 (0.932-0.953) | 1.000 (1.000-1.000) | 0.791 (0.771-0.811) | 0.575 (0.508-0.646) | 0.138 (0.129-0.147) |
| AdaBoost+DT+SVM+XGBoost+CatBoost | 21.714 | Rank 15 | 0.811 (0.793-0.829) | 0.733 (0.693-0.764) | 0.947 (0.937-0.958) | 0.974 (0.958-1.000) | 0.791 (0.771-0.810) | 0.688 (0.626-0.750) | 0.135 (0.127-0.143) |
| DT+NaiveBayes+SVM+XGBoost+CatBoost | 22.714 | Rank 16 | 0.816 (0.799-0.833) | 0.738 (0.705-0.771) | 0.942 (0.931-0.952) | 0.975 (0.958-1.000) | 0.796 (0.777-0.815) | 0.619 (0.548-0.691) | 0.138 (0.130-0.145) |
| AdaBoost+RF+NaiveBayes+SVM+XGBoost | 22.857 | Rank 17 | 0.822 (0.805-0.841) | 0.744 (0.708-0.778) | 0.938 (0.927-0.949) | 0.975 (0.958-1.000) | 0.803 (0.785-0.822) | 0.614 (0.540-0.684) | 0.139 (0.131-0.146) |
| RF+NaiveBayes+XGBoost+CatBoost | 24.286 | Rank 18 | 0.802 (0.784-0.821) | 0.737 (0.703-0.769) | 0.944 (0.934-0.955) | 1.000 (1.000-1.000) | 0.777 (0.757-0.799) | 0.638 (0.575-0.701) | 0.139 (0.129-0.148) |
| DT+XGBoost | 25.429 | Rank 19 | 0.814 (0.796-0.832) | 0.751 (0.719-0.780) | 0.943 (0.933-0.953) | 1.000 (1.000-1.000) | 0.791 (0.771-0.811) | 0.523 (0.459-0.597) | 0.137 (0.129-0.145) |
| RF+XGBoost | 26.286 | Rank 20 | 0.806 (0.788-0.822) | 0.742 (0.707-0.771) | 0.945 (0.934-0.955) | 1.000 (1.000-1.000) | 0.781 (0.762-0.800) | 0.618 (0.544-0.689) | 0.146 (0.139-0.153) |
| AdaBoost+DT+RF+XGBoost | 26.571 | Rank 21 | 0.814 (0.796-0.832) | 0.737 (0.702-0.770) | 0.939 (0.928-0.949) | 0.975 (0.957-1.000) | 0.794 (0.773-0.813) | 0.604 (0.536-0.669) | 0.138 (0.130-0.147) |
| AdaBoost+XGBoost+CatBoost | 29.286 | Rank 22 | 0.808 (0.789-0.828) | 0.726 (0.688-0.760) | 0.942 (0.930-0.953) | 0.966 (0.944-0.988) | 0.788 (0.767-0.810) | 0.644 (0.574-0.711) | 0.137 (0.130-0.145) |
| SVM+XGBoost+CatBoost | 30.143 | Rank 23 | 0.803 (0.785-0.822) | 0.726 (0.691-0.757) | 0.945 (0.933-0.956) | 0.975 (0.957-1.000) | 0.781 (0.762-0.802) | 0.626 (0.553-0.695) | 0.139 (0.131-0.146) |
| DT+NaiveBayes+SVM+XGBoost | 30.714 | Rank 24 | 0.796 (0.778-0.814) | 0.731 (0.697-0.762) | 0.958 (0.949-0.967) | 1.000 (1.000-1.000) | 0.770 (0.750-0.789) | 0.679 (0.604-0.748) | 0.151 (0.144-0.158) |
| AdaBoost+DT+XGBoost+CatBoost | 31.286 | Rank 25 | 0.803 (0.785-0.821) | 0.731 (0.694-0.764) | 0.936 (0.924-0.948) | 0.983 (0.971-1.000) | 0.780 (0.761-0.800) | 0.630 (0.558-0.694) | 0.140 (0.131-0.149) |
| DT+NaiveBayes+XGBoost | 31.857 | Rank 26 | 0.808 (0.790-0.827) | 0.744 (0.708-0.775) | 0.935 (0.924-0.947) | 1.000 (1.000-1.000) | 0.784 (0.764-0.804) | 0.561 (0.483-0.643) | 0.145 (0.136-0.153) |
| AdaBoost+DT+RF+XGBoost+CatBoost | 32.000 | Rank 27 | 0.809 (0.793-0.827) | 0.732 (0.698-0.765) | 0.936 (0.924-0.948) | 0.975 (0.957-1.000) | 0.788 (0.770-0.808) | 0.605 (0.533-0.674) | 0.141 (0.133-0.150) |
| DT+RF+SVM+XGBoost | 33.000 | Rank 28 | 0.813 (0.796-0.830) | 0.735 (0.698-0.767) | 0.935 (0.924-0.947) | 0.974 (0.957-1.000) | 0.792 (0.774-0.811) | 0.592 (0.516-0.664) | 0.143 (0.136-0.151) |
| AdaBoost+RF+SVM+XGBoost | 33.714 | Rank 29 | 0.820 (0.803-0.837) | 0.715 (0.679-0.750) | 0.928 (0.915-0.942) | 0.925 (0.891-0.962) | 0.807 (0.789-0.825) | 0.587 (0.520-0.657) | 0.138 (0.130-0.144) |
| SVM+XGBoost | 33.714 | Rank 29 | 0.815 (0.796-0.833) | 0.751 (0.714-0.781) | 0.938 (0.926-0.948) | 1.000 (1.000-1.000) | 0.791 (0.771-0.812) | 0.521 (0.453-0.595) | 0.150 (0.143-0.157) |
| NaiveBayes+XGBoost | 34.143 | Rank 31 | 0.799 (0.780-0.817) | 0.735 (0.701-0.768) | 0.941 (0.931-0.952) | 1.000 (1.000-1.000) | 0.774 (0.754-0.794) | 0.567 (0.494-0.646) | 0.144 (0.136-0.151) |
| AdaBoost+RF+NaiveBayes+XGBoost+CatBoost | 36.143 | Rank 32 | 0.798 (0.779-0.816) | 0.716 (0.679-0.750) | 0.940 (0.928-0.952) | 0.966 (0.944-0.988) | 0.776 (0.756-0.796) | 0.674 (0.610-0.735) | 0.142 (0.134-0.150) |
| DT+RF+SVM+XGBoost+CatBoost | 36.286 | Rank 33 | 0.795 (0.779-0.814) | 0.718 (0.683-0.750) | 0.941 (0.930-0.953) | 0.974 (0.958-1.000) | 0.772 (0.753-0.794) | 0.658 (0.592-0.724) | 0.141 (0.132-0.150) |
| AdaBoost+DT+RF+NaiveBayes+XGBoost | 37.571 | Rank 34 | 0.792 (0.774-0.810) | 0.729 (0.692-0.758) | 0.943 (0.932-0.953) | 1.000 (1.000-1.000) | 0.766 (0.746-0.786) | 0.615 (0.541-0.683) | 0.150 (0.141-0.159) |
| RF+SVM+XGBoost | 39.000 | Rank 35 | 0.810 (0.793-0.829) | 0.728 (0.693-0.763) | 0.934 (0.922-0.946) | 0.967 (0.944-0.988) | 0.790 (0.771-0.810) | 0.583 (0.512-0.650) | 0.147 (0.139-0.154) |
| RF+NaiveBayes+SVM+XGBoost | 40.429 | Rank 36 | 0.830 (0.813-0.847) | 0.731 (0.693-0.766) | 0.922 (0.910-0.934) | 0.935 (0.902-0.972) | 0.817 (0.798-0.836) | 0.486 (0.417-0.559) | 0.140 (0.132-0.149) |
| AdaBoost+NaiveBayes+SVM+XGBoost | 40.571 | Rank 37 | 0.821 (0.804-0.838) | 0.738 (0.701-0.771) | 0.925 (0.913-0.938) | 0.967 (0.945-0.988) | 0.802 (0.784-0.822) | 0.510 (0.438-0.580) | 0.145 (0.138-0.152) |
| AdaBoost+DT+NaiveBayes+SVM+XGBoost+CatBoost | 41.571 | Rank 38 | 0.793 (0.773-0.810) | 0.716 (0.679-0.750) | 0.940 (0.928-0.951) | 0.975 (0.957-1.000) | 0.770 (0.748-0.789) | 0.621 (0.550-0.685) | 0.144 (0.136-0.152) |
| AdaBoost+DT+RF+SVM | 42.143 | Rank 39 | 0.842 (0.825-0.858) | 0.709 (0.663-0.751) | 0.921 (0.906-0.935) | 0.873 (0.831-0.918) | 0.838 (0.821-0.855) | 0.530 (0.454-0.610) | 0.136 (0.130-0.142) |
| AdaBoost+DT+NaiveBayes+XGBoost | 45.714 | Rank 40 | 0.813 (0.794-0.831) | 0.748 (0.716-0.780) | 0.911 (0.897-0.923) | 1.000 (1.000-1.000) | 0.789 (0.769-0.810) | 0.431 (0.371-0.499) | 0.151 (0.144-0.159) |
| DT+RF+NaiveBayes+XGBoost | 46.286 | Rank 41 | 0.788 (0.769-0.808) | 0.724 (0.686-0.758) | 0.939 (0.928-0.949) | 1.000 (1.000-1.000) | 0.761 (0.740-0.784) | 0.557 (0.487-0.634) | 0.149 (0.139-0.157) |
| NaiveBayes+XGBoost+CatBoost | 46.429 | Rank 42 | 0.798 (0.779-0.816) | 0.694 (0.655-0.731) | 0.927 (0.913-0.940) | 0.924 (0.890-0.960) | 0.782 (0.762-0.802) | 0.601 (0.535-0.664) | 0.143 (0.135-0.151) |
| RF+SVM+XGBoost+CatBoost | 46.429 | Rank 42 | 0.803 (0.784-0.820) | 0.680 (0.638-0.720) | 0.920 (0.905-0.934) | 0.892 (0.848-0.935) | 0.791 (0.772-0.810) | 0.618 (0.550-0.679) | 0.140 (0.133-0.148) |
| DT+RF+NaiveBayes+XGBoost+CatBoost | 47.571 | Rank 44 | 0.794 (0.776-0.812) | 0.703 (0.661-0.739) | 0.927 (0.912-0.940) | 0.950 (0.922-0.976) | 0.774 (0.754-0.794) | 0.595 (0.519-0.668) | 0.143 (0.135-0.152) |
| AdaBoost+SVM+CatBoost | 48.286 | Rank 45 | 0.809 (0.791-0.827) | 0.681 (0.637-0.722) | 0.919 (0.904-0.934) | 0.883 (0.838-0.932) | 0.800 (0.780-0.819) | 0.562 (0.486-0.636) | 0.139 (0.132-0.146) |
| DT+SVM+XGBoost+CatBoost | 48.429 | Rank 46 | 0.785 (0.767-0.803) | 0.716 (0.681-0.748) | 0.931 (0.920-0.943) | 0.992 (0.985-1.000) | 0.759 (0.739-0.780) | 0.597 (0.527-0.660) | 0.145 (0.137-0.153) |
| RF+CatBoost | 48.429 | Rank 46 | 0.806 (0.788-0.825) | 0.683 (0.639-0.724) | 0.920 (0.906-0.935) | 0.892 (0.851-0.935) | 0.795 (0.776-0.815) | 0.583 (0.506-0.659) | 0.144 (0.137-0.150) |
| AdaBoost+DT+RF+NaiveBayes+XGBoost+CatBoost | 49.286 | Rank 48 | 0.787 (0.770-0.806) | 0.724 (0.688-0.755) | 0.938 (0.927-0.949) | 1.000 (1.000-1.000) | 0.760 (0.741-0.782) | 0.555 (0.478-0.633) | 0.152 (0.142-0.162) |
| AdaBoost+RF+SVM+XGBoost+CatBoost | 51.143 | Rank 49 | 0.790 (0.772-0.809) | 0.709 (0.673-0.743) | 0.922 (0.909-0.935) | 0.967 (0.945-1.000) | 0.768 (0.748-0.788) | 0.565 (0.494-0.631) | 0.143 (0.134-0.152) |
| DT+RF | 51.143 | Rank 49 | 0.813 (0.795-0.831) | 0.670 (0.625-0.713) | 0.914 (0.896-0.930) | 0.858 (0.812-0.908) | 0.807 (0.788-0.826) | 0.573 (0.497-0.639) | 0.141 (0.135-0.148) |
| NaiveBayes+CatBoost | 51.143 | Rank 49 | 0.798 (0.780-0.817) | 0.685 (0.644-0.723) | 0.917 (0.902-0.933) | 0.908 (0.871-0.948) | 0.784 (0.764-0.805) | 0.594 (0.520-0.660) | 0.144 (0.135-0.152) |
| AdaBoost+DT+RF+NaiveBayes+SVM+XGBoost+CatBoost | 52.857 | Rank 52 | 0.785 (0.768-0.804) | 0.705 (0.665-0.738) | 0.932 (0.919-0.944) | 0.967 (0.944-0.988) | 0.763 (0.743-0.782) | 0.589 (0.515-0.656) | 0.147 (0.138-0.156) |
| AdaBoost+DT+NaiveBayes+SVM+XGBoost | 53.143 | Rank 53 | 0.797 (0.779-0.815) | 0.733 (0.697-0.763) | 0.919 (0.905-0.933) | 1.000 (1.000-1.000) | 0.772 (0.752-0.791) | 0.525 (0.450-0.602) | 0.160 (0.153-0.167) |
| NaiveBayes+SVM+CatBoost | 53.714 | Rank 54 | 0.811 (0.795-0.829) | 0.669 (0.622-0.713) | 0.914 (0.899-0.929) | 0.858 (0.809-0.906) | 0.805 (0.788-0.825) | 0.580 (0.510-0.646) | 0.145 (0.139-0.151) |
| AdaBoost+RF+NaiveBayes+XGBoost | 54.429 | Rank 55 | 0.782 (0.764-0.800) | 0.696 (0.657-0.733) | 0.934 (0.921-0.948) | 0.958 (0.932-0.987) | 0.759 (0.739-0.779) | 0.659 (0.594-0.719) | 0.153 (0.146-0.159) |
| AdaBoost+RF+XGBoost+CatBoost | 54.429 | Rank 55 | 0.776 (0.758-0.794) | 0.704 (0.669-0.738) | 0.941 (0.930-0.952) | 0.983 (0.971-1.000) | 0.750 (0.730-0.771) | 0.590 (0.512-0.666) | 0.149 (0.140-0.158) |
| DT+RF+XGBoost | 55.429 | Rank 57 | 0.783 (0.763-0.802) | 0.710 (0.669-0.744) | 0.934 (0.920-0.946) | 0.983 (0.971-1.000) | 0.757 (0.736-0.779) | 0.583 (0.503-0.654) | 0.152 (0.142-0.161) |
| DT+SVM+XGBoost | 57.429 | Rank 58 | 0.791 (0.772-0.810) | 0.727 (0.690-0.760) | 0.925 (0.913-0.937) | 1.000 (1.000-1.000) | 0.765 (0.744-0.785) | 0.504 (0.435-0.581) | 0.160 (0.152-0.168) |
| DT+RF+NaiveBayes+SVM+XGBoost | 57.714 | Rank 59 | 0.778 (0.761-0.797) | 0.716 (0.680-0.747) | 0.931 (0.920-0.942) | 1.000 (1.000-1.000) | 0.750 (0.732-0.771) | 0.551 (0.480-0.623) | 0.158 (0.148-0.166) |
| DT+RF+XGBoost+CatBoost | 57.714 | Rank 59 | 0.784 (0.765-0.803) | 0.708 (0.671-0.741) | 0.929 (0.917-0.940) | 0.975 (0.957-1.000) | 0.759 (0.738-0.781) | 0.557 (0.487-0.624) | 0.149 (0.140-0.158) |
| AdaBoost+DT+NaiveBayes+XGBoost+CatBoost | 58.286 | Rank 61 | 0.787 (0.768-0.806) | 0.706 (0.669-0.741) | 0.929 (0.917-0.941) | 0.966 (0.944-0.988) | 0.765 (0.743-0.785) | 0.563 (0.486-0.637) | 0.154 (0.145-0.162) |
| AdaBoost+DT+SVM+XGBoost | 59.000 | Rank 62 | 0.801 (0.783-0.821) | 0.724 (0.687-0.759) | 0.918 (0.904-0.932) | 0.975 (0.958-1.000) | 0.779 (0.760-0.800) | 0.509 (0.434-0.586) | 0.159 (0.151-0.166) |
| AdaBoost+DT+RF | 59.143 | Rank 63 | 0.809 (0.792-0.828) | 0.634 (0.587-0.680) | 0.905 (0.887-0.923) | 0.800 (0.744-0.855) | 0.811 (0.792-0.829) | 0.559 (0.484-0.631) | 0.140 (0.133-0.147) |
| DT+NaiveBayes+SVM+CatBoost | 62.143 | Rank 64 | 0.793 (0.776-0.811) | 0.685 (0.649-0.722) | 0.914 (0.900-0.929) | 0.917 (0.883-0.958) | 0.778 (0.759-0.798) | 0.518 (0.450-0.597) | 0.147 (0.139-0.155) |
| RF+SVM+CatBoost | 62.143 | Rank 64 | 0.789 (0.771-0.808) | 0.681 (0.642-0.720) | 0.914 (0.899-0.929) | 0.917 (0.880-0.959) | 0.773 (0.753-0.794) | 0.560 (0.489-0.629) | 0.150 (0.142-0.158) |
| AdaBoost+DT+RF+NaiveBayes+SVM+CatBoost | 62.571 | Rank 66 | 0.786 (0.767-0.805) | 0.660 (0.616-0.701) | 0.915 (0.900-0.931) | 0.882 (0.842-0.927) | 0.774 (0.754-0.795) | 0.595 (0.519-0.663) | 0.146 (0.139-0.154) |
| RF+NaiveBayes+XGBoost | 62.714 | Rank 67 | 0.764 (0.744-0.783) | 0.694 (0.654-0.725) | 0.929 (0.916-0.941) | 0.983 (0.971-1.000) | 0.736 (0.715-0.758) | 0.605 (0.536-0.668) | 0.157 (0.148-0.166) |
| AdaBoost+SVM+XGBoost | 65.714 | Rank 68 | 0.797 (0.778-0.814) | 0.720 (0.679-0.756) | 0.910 (0.896-0.924) | 0.975 (0.958-1.000) | 0.774 (0.754-0.794) | 0.461 (0.394-0.527) | 0.169 (0.161-0.177) |
| DT+RF+NaiveBayes+SVM+CatBoost | 66.286 | Rank 69 | 0.787 (0.768-0.806) | 0.662 (0.617-0.702) | 0.911 (0.895-0.926) | 0.884 (0.842-0.932) | 0.774 (0.753-0.795) | 0.549 (0.476-0.621) | 0.145 (0.136-0.154) |
| RF+NaiveBayes+CatBoost | 66.286 | Rank 69 | 0.787 (0.768-0.806) | 0.675 (0.631-0.714) | 0.909 (0.893-0.924) | 0.909 (0.870-0.948) | 0.772 (0.751-0.791) | 0.536 (0.469-0.602) | 0.146 (0.136-0.155) |
| AdaBoost+SVM+XGBoost+CatBoost | 68.143 | Rank 71 | 0.774 (0.756-0.793) | 0.672 (0.633-0.710) | 0.917 (0.902-0.931) | 0.924 (0.889-0.962) | 0.756 (0.736-0.776) | 0.568 (0.495-0.637) | 0.148 (0.140-0.156) |
| AdaBoost+RF+NaiveBayes+CatBoost | 68.286 | Rank 72 | 0.782 (0.763-0.802) | 0.692 (0.653-0.725) | 0.917 (0.904-0.930) | 0.950 (0.922-0.984) | 0.761 (0.740-0.782) | 0.515 (0.446-0.582) | 0.150 (0.140-0.160) |
| DT+SVM+CatBoost | 68.286 | Rank 72 | 0.801 (0.783-0.818) | 0.646 (0.597-0.687) | 0.900 (0.882-0.917) | 0.833 (0.786-0.884) | 0.797 (0.777-0.815) | 0.538 (0.465-0.613) | 0.148 (0.140-0.155) |
| AdaBoost+RF+SVM+CatBoost | 70.143 | Rank 74 | 0.764 (0.745-0.784) | 0.669 (0.631-0.705) | 0.919 (0.904-0.934) | 0.935 (0.901-0.972) | 0.743 (0.722-0.765) | 0.608 (0.540-0.677) | 0.158 (0.150-0.166) |
| AdaBoost+DT+NaiveBayes+SVM+CatBoost | 70.714 | Rank 75 | 0.778 (0.760-0.797) | 0.681 (0.643-0.716) | 0.912 (0.896-0.927) | 0.933 (0.901-0.971) | 0.759 (0.739-0.779) | 0.546 (0.468-0.619) | 0.151 (0.142-0.160) |
| AdaBoost+RF+NaiveBayes | 71.143 | Rank 76 | 0.788 (0.770-0.807) | 0.681 (0.639-0.718) | 0.904 (0.888-0.922) | 0.917 (0.880-0.957) | 0.772 (0.752-0.793) | 0.516 (0.439-0.588) | 0.156 (0.148-0.164) |
| AdaBoost+DT+RF+NaiveBayes | 72.000 | Rank 77 | 0.762 (0.744-0.781) | 0.675 (0.635-0.713) | 0.924 (0.910-0.939) | 0.951 (0.922-0.986) | 0.738 (0.718-0.759) | 0.567 (0.497-0.637) | 0.157 (0.149-0.165) |
| AdaBoost+RF+NaiveBayes+SVM | 72.714 | Rank 78 | 0.798 (0.778-0.818) | 0.671 (0.629-0.713) | 0.900 (0.882-0.916) | 0.884 (0.840-0.926) | 0.787 (0.766-0.807) | 0.519 (0.444-0.586) | 0.159 (0.152-0.166) |
| DT+RF+NaiveBayes+CatBoost | 73.571 | Rank 79 | 0.757 (0.738-0.777) | 0.653 (0.612-0.691) | 0.915 (0.899-0.931) | 0.917 (0.881-0.957) | 0.737 (0.715-0.758) | 0.606 (0.538-0.669) | 0.150 (0.141-0.158) |
| DT+RF+SVM+CatBoost | 73.857 | Rank 80 | 0.784 (0.765-0.802) | 0.659 (0.616-0.697) | 0.901 (0.882-0.917) | 0.884 (0.840-0.931) | 0.771 (0.751-0.791) | 0.530 (0.450-0.607) | 0.148 (0.139-0.158) |
| AdaBoost+NaiveBayes+SVM+XGBoost+CatBoost | 74.000 | Rank 81 | 0.768 (0.750-0.786) | 0.702 (0.666-0.733) | 0.917 (0.904-0.929) | 0.992 (0.985-1.000) | 0.740 (0.720-0.761) | 0.513 (0.443-0.582) | 0.164 (0.153-0.174) |
| AdaBoost+RF+NaiveBayes+SVM+CatBoost | 74.571 | Rank 82 | 0.778 (0.760-0.798) | 0.645 (0.600-0.684) | 0.902 (0.885-0.919) | 0.866 (0.821-0.912) | 0.767 (0.746-0.788) | 0.576 (0.506-0.638) | 0.147 (0.139-0.156) |
| DT+NaiveBayes+CatBoost | 74.714 | Rank 83 | 0.789 (0.771-0.807) | 0.654 (0.613-0.695) | 0.900 (0.881-0.919) | 0.865 (0.819-0.914) | 0.780 (0.761-0.799) | 0.543 (0.473-0.607) | 0.155 (0.149-0.161) |
| DT+RF+NaiveBayes+SVM | 76.857 | Rank 84 | 0.779 (0.761-0.798) | 0.659 (0.618-0.701) | 0.907 (0.889-0.924) | 0.891 (0.849-0.938) | 0.765 (0.745-0.786) | 0.541 (0.464-0.611) | 0.156 (0.149-0.163) |
| AdaBoost+DT+SVM+CatBoost | 78.143 | Rank 85 | 0.774 (0.756-0.794) | 0.655 (0.612-0.697) | 0.910 (0.894-0.925) | 0.891 (0.848-0.933) | 0.759 (0.738-0.781) | 0.542 (0.466-0.611) | 0.154 (0.146-0.161) |
| RF+NaiveBayes+SVM+CatBoost | 78.714 | Rank 86 | 0.777 (0.758-0.797) | 0.676 (0.633-0.716) | 0.902 (0.886-0.919) | 0.925 (0.890-0.960) | 0.759 (0.738-0.780) | 0.504 (0.430-0.572) | 0.156 (0.146-0.165) |
| AdaBoost+DT+RF+NaiveBayes+CatBoost | 80.143 | Rank 87 | 0.781 (0.763-0.800) | 0.648 (0.602-0.688) | 0.902 (0.883-0.919) | 0.868 (0.822-0.918) | 0.771 (0.750-0.791) | 0.513 (0.437-0.587) | 0.150 (0.142-0.157) |
| AdaBoost+DT+RF+SVM+CatBoost | 80.857 | Rank 88 | 0.772 (0.753-0.792) | 0.653 (0.612-0.694) | 0.909 (0.894-0.924) | 0.892 (0.853-0.935) | 0.757 (0.737-0.778) | 0.528 (0.444-0.605) | 0.154 (0.145-0.162) |
| AdaBoost+DT+RF+CatBoost | 83.857 | Rank 89 | 0.772 (0.754-0.792) | 0.620 (0.576-0.664) | 0.900 (0.883-0.917) | 0.832 (0.781-0.886) | 0.764 (0.744-0.785) | 0.537 (0.466-0.608) | 0.148 (0.138-0.156) |
| DT+RF+SVM | 85.714 | Rank 90 | 0.768 (0.749-0.787) | 0.640 (0.595-0.681) | 0.903 (0.886-0.921) | 0.875 (0.833-0.921) | 0.755 (0.733-0.775) | 0.560 (0.484-0.633) | 0.159 (0.152-0.166) |
| AdaBoost+RF+SVM | 85.857 | Rank 91 | 0.774 (0.754-0.793) | 0.650 (0.606-0.690) | 0.897 (0.881-0.914) | 0.884 (0.842-0.926) | 0.760 (0.738-0.781) | 0.529 (0.452-0.594) | 0.160 (0.153-0.167) |
| AdaBoost+DT+CatBoost | 86.000 | Rank 92 | 0.753 (0.734-0.774) | 0.654 (0.613-0.693) | 0.907 (0.891-0.923) | 0.925 (0.889-0.962) | 0.732 (0.710-0.755) | 0.514 (0.438-0.594) | 0.158 (0.148-0.167) |
| NaiveBayes+SVM+XGBoost | 86.143 | Rank 93 | 0.769 (0.748-0.788) | 0.640 (0.597-0.683) | 0.900 (0.883-0.918) | 0.874 (0.827-0.923) | 0.756 (0.735-0.776) | 0.562 (0.486-0.633) | 0.160 (0.153-0.167) |
| RF+NaiveBayes+SVM | 88.857 | Rank 94 | 0.758 (0.739-0.777) | 0.650 (0.607-0.687) | 0.900 (0.882-0.918) | 0.908 (0.870-0.945) | 0.740 (0.719-0.760) | 0.529 (0.455-0.602) | 0.161 (0.154-0.168) |
| DT+RF+NaiveBayes | 90.286 | Rank 95 | 0.757 (0.737-0.778) | 0.646 (0.600-0.685) | 0.899 (0.880-0.918) | 0.900 (0.861-0.944) | 0.739 (0.718-0.762) | 0.515 (0.442-0.589) | 0.158 (0.149-0.166) |
| AdaBoost+NaiveBayes+CatBoost | 91.714 | Rank 96 | 0.743 (0.723-0.762) | 0.642 (0.602-0.679) | 0.896 (0.878-0.914) | 0.916 (0.883-0.957) | 0.721 (0.700-0.741) | 0.557 (0.493-0.621) | 0.165 (0.157-0.173) |
| AdaBoost+DT+NaiveBayes+CatBoost | 92.000 | Rank 97 | 0.766 (0.747-0.787) | 0.657 (0.615-0.696) | 0.891 (0.873-0.908) | 0.909 (0.870-0.947) | 0.748 (0.727-0.770) | 0.458 (0.386-0.529) | 0.163 (0.153-0.172) |
| AdaBoost+DT+RF+NaiveBayes+SVM | 92.714 | Rank 98 | 0.768 (0.749-0.787) | 0.633 (0.589-0.674) | 0.892 (0.873-0.910) | 0.860 (0.814-0.910) | 0.757 (0.736-0.778) | 0.525 (0.455-0.593) | 0.158 (0.150-0.167) |
| AdaBoost+NaiveBayes+SVM+CatBoost | 94.286 | Rank 99 | 0.745 (0.724-0.765) | 0.640 (0.600-0.678) | 0.896 (0.879-0.915) | 0.909 (0.875-0.948) | 0.724 (0.702-0.746) | 0.537 (0.464-0.600) | 0.168 (0.159-0.175) |
| DT+CatBoost | 95.143 | Rank 100 | 0.740 (0.719-0.761) | 0.618 (0.574-0.656) | 0.897 (0.878-0.916) | 0.874 (0.831-0.921) | 0.723 (0.701-0.746) | 0.574 (0.511-0.636) | 0.166 (0.159-0.173) |
| AdaBoost+RF | 95.429 | Rank 101 | 0.758 (0.738-0.777) | 0.645 (0.604-0.684) | 0.893 (0.873-0.912) | 0.899 (0.857-0.944) | 0.740 (0.718-0.761) | 0.492 (0.421-0.566) | 0.166 (0.157-0.174) |
| AdaBoost+NaiveBayes+XGBoost | 95.857 | Rank 102 | 0.756 (0.738-0.776) | 0.649 (0.607-0.686) | 0.898 (0.882-0.914) | 0.907 (0.870-0.947) | 0.737 (0.718-0.758) | 0.459 (0.391-0.537) | 0.168 (0.161-0.175) |
| NaiveBayes | 96.000 | Rank 103 | 0.786 (0.731-0.841) | 0.294 (0.199-0.388) | 0.668 (0.627-0.708) | 0.339 (0.220-0.457) | 0.841 (0.773-0.909) | 0.267 (0.187-0.347) | 0.176 (0.143-0.208) |
| DT+RF+CatBoost | 96.571 | Rank 104 | 0.766 (0.746-0.785) | 0.620 (0.574-0.662) | 0.885 (0.866-0.904) | 0.841 (0.792-0.894) | 0.757 (0.735-0.779) | 0.510 (0.439-0.578) | 0.158 (0.150-0.167) |
| SVM+CatBoost | 96.714 | Rank 105 | 0.748 (0.728-0.768) | 0.624 (0.582-0.663) | 0.893 (0.874-0.912) | 0.875 (0.831-0.922) | 0.732 (0.710-0.754) | 0.542 (0.471-0.604) | 0.166 (0.158-0.174) |
| RF+SVM | 98.857 | Rank 106 | 0.749 (0.729-0.769) | 0.638 (0.598-0.677) | 0.887 (0.868-0.907) | 0.900 (0.859-0.944) | 0.730 (0.708-0.752) | 0.506 (0.428-0.586) | 0.167 (0.158-0.175) |
| RF+NaiveBayes | 99.143 | Rank 107 | 0.759 (0.738-0.780) | 0.629 (0.582-0.672) | 0.892 (0.872-0.911) | 0.867 (0.819-0.918) | 0.745 (0.724-0.767) | 0.467 (0.393-0.543) | 0.164 (0.156-0.173) |
| AdaBoost+RF+CatBoost | 100.857 | Rank 108 | 0.746 (0.726-0.765) | 0.622 (0.577-0.662) | 0.892 (0.874-0.911) | 0.875 (0.831-0.917) | 0.729 (0.708-0.751) | 0.489 (0.418-0.569) | 0.161 (0.153-0.170) |
| AdaBoost+CatBoost | 107.286 | Rank 109 | 0.741 (0.722-0.761) | 0.609 (0.564-0.653) | 0.867 (0.846-0.887) | 0.857 (0.809-0.905) | 0.726 (0.706-0.748) | 0.389 (0.326-0.461) | 0.170 (0.160-0.178) |
| AdaBoost+DT+XGBoost | 109.714 | Rank 110 | 0.725 (0.704-0.745) | 0.543 (0.499-0.588) | 0.820 (0.793-0.847) | 0.759 (0.703-0.819) | 0.720 (0.699-0.742) | 0.344 (0.281-0.410) | 0.188 (0.181-0.195) |
| AdaBoost+NaiveBayes | 111.714 | Rank 111 | 0.728 (0.707-0.748) | 0.360 (0.307-0.409) | 0.691 (0.654-0.726) | 0.458 (0.390-0.521) | 0.762 (0.741-0.782) | 0.248 (0.192-0.303) | 0.211 (0.204-0.217) |
| DT+SVM | 112.714 | Rank 112 | 0.687 (0.666-0.707) | 0.457 (0.411-0.504) | 0.767 (0.737-0.796) | 0.650 (0.587-0.714) | 0.691 (0.668-0.713) | 0.302 (0.247-0.360) | 0.192 (0.185-0.199) |
| AdaBoost+DT+SVM | 113.714 | Rank 113 | 0.671 (0.649-0.693) | 0.468 (0.421-0.511) | 0.749 (0.714-0.783) | 0.684 (0.625-0.740) | 0.670 (0.647-0.693) | 0.317 (0.253-0.382) | 0.209 (0.201-0.218) |
| AdaBoost+DT | 114.429 | Rank 114 | 0.690 (0.671-0.710) | 0.478 (0.429-0.524) | 0.721 (0.686-0.758) | 0.684 (0.619-0.750) | 0.691 (0.669-0.713) | 0.235 (0.191-0.277) | 0.207 (0.200-0.213) |
| AdaBoost+SVM | 114.714 | Rank 115 | 0.672 (0.649-0.692) | 0.434 (0.382-0.481) | 0.740 (0.706-0.774) | 0.625 (0.556-0.693) | 0.678 (0.654-0.699) | 0.305 (0.244-0.363) | 0.201 (0.194-0.209) |
| DT+NaiveBayes+SVM | 114.714 | Rank 115 | 0.686 (0.664-0.708) | 0.462 (0.410-0.511) | 0.739 (0.702-0.774) | 0.659 (0.592-0.722) | 0.689 (0.667-0.713) | 0.282 (0.227-0.339) | 0.211 (0.204-0.218) |
| AdaBoost+DT+NaiveBayes+SVM | 116.429 | Rank 117 | 0.638 (0.616-0.661) | 0.481 (0.434-0.525) | 0.748 (0.716-0.780) | 0.743 (0.680-0.810) | 0.625 (0.602-0.648) | 0.294 (0.236-0.349) | 0.223 (0.215-0.232) |
| AdaBoost+NaiveBayes+SVM | 116.571 | Rank 118 | 0.661 (0.639-0.681) | 0.452 (0.401-0.497) | 0.737 (0.704-0.772) | 0.666 (0.600-0.732) | 0.660 (0.637-0.682) | 0.289 (0.231-0.351) | 0.211 (0.205-0.218) |
| DT+NaiveBayes | 119.571 | Rank 119 | 0.619 (0.599-0.641) | 0.440 (0.393-0.485) | 0.721 (0.684-0.754) | 0.686 (0.625-0.747) | 0.611 (0.588-0.635) | 0.236 (0.190-0.285) | 0.218 (0.211-0.226) |
| NaiveBayes+SVM | 119.714 | Rank 120 | 0.660 (0.640-0.682) | 0.404 (0.353-0.450) | 0.708 (0.673-0.743) | 0.583 (0.519-0.646) | 0.670 (0.648-0.693) | 0.247 (0.196-0.297) | 0.225 (0.219-0.232) |
| AdaBoost+DT+NaiveBayes | 120.857 | Rank 121 | 0.626 (0.605-0.647) | 0.421 (0.375-0.468) | 0.701 (0.662-0.737) | 0.642 (0.573-0.709) | 0.624 (0.600-0.648) | 0.231 (0.186-0.276) | 0.212 (0.207-0.218) |
| CatBoost | 121.000 | Rank 122 | 0.643 (0.608-0.678) | 0.355 (0.309-0.402) | 0.661 (0.613-0.709) | 0.526 (0.448-0.603) | 0.659 (0.617-0.701) | 0.252 (0.155-0.350) | 0.212 (0.197-0.227) |
| RF | 121.000 | Rank 122 | 0.664 (0.636-0.693) | 0.293 (0.230-0.356) | 0.626 (0.561-0.691) | 0.410 (0.306-0.513) | 0.696 (0.665-0.727) | 0.212 (0.143-0.281) | 0.202 (0.193-0.210) |
| XGBoost | 123.143 | Rank 124 | 0.663 (0.629-0.696) | 0.320 (0.219-0.422) | 0.602 (0.523-0.682) | 0.457 (0.291-0.623) | 0.689 (0.658-0.720) | 0.183 (0.125-0.242) | 0.270 (0.245-0.296) |
| AdaBoost | 124.000 | Rank 125 | 0.578 (0.529-0.626) | 0.363 (0.298-0.428) | 0.632 (0.585-0.679) | 0.595 (0.459-0.732) | 0.574 (0.507-0.642) | 0.212 (0.149-0.276) | 0.233 (0.221-0.245) |
| LightGBM | 124.286 | Rank 126 | 0.647 (0.608-0.685) | 0.298 (0.241-0.355) | 0.601 (0.549-0.653) | 0.424 (0.336-0.513) | 0.675 (0.639-0.710) | 0.167 (0.130-0.205) | 0.230 (0.213-0.247) |
| DT | 125.286 | Rank 127 | 0.612 (0.575-0.650) | 0.319 (0.244-0.394) | 0.596 (0.507-0.685) | 0.492 (0.361-0.623) | 0.629 (0.585-0.673) | 0.188 (0.138-0.237) | 0.242 (0.227-0.258) |
| SVM | 126.286 | Rank 128 | 0.609 (0.570-0.647) | 0.298 (0.205-0.392) | 0.601 (0.542-0.660) | 0.454 (0.311-0.597) | 0.628 (0.587-0.670) | 0.159 (0.121-0.197) | 0.234 (0.225-0.244) |
| KNN | 126.429 | Rank 129 | 0.578 (0.537-0.618) | 0.362 (0.286-0.438) | 0.594 (0.530-0.659) | 0.580 (0.465-0.694) | 0.577 (0.534-0.619) | 0.158 (0.121-0.194) | 0.291 (0.266-0.315) |

**Supplementary Table 19.** Ranking of 129 machine learning models in the SHARE validation set.

| **Model** | **Normalized Rank Score** | **Comprehensive Rank** | **Accuracy (95% CI)** | **F_Beta (95% CI)** | **ROC_AUC (95% CI)** | **Sensitivity (95% CI)** | **Specificity (95% CI)** | **PR_AUC (95% CI)** | **Brier (95% CI)** |
| --- | --- | --- | --- | --- | --- | --- | --- | --- | --- |
| AdaBoost+XGBoost | 6.000 | Rank 1 | 0.724 (0.693-0.755) | 0.407 (0.328-0.490) | 0.652 (0.591-0.720) | 0.541 (0.440-0.655) | 0.746 (0.713-0.777) | 0.244 (0.159-0.319) | 0.191 (0.176-0.205) |
| NaiveBayes+SVM+XGBoost | 15.857 | Rank 2 | 0.709 (0.677-0.738) | 0.383 (0.297-0.459) | 0.618 (0.555-0.680) | 0.517 (0.406-0.621) | 0.732 (0.700-0.765) | 0.167 (0.109-0.217) | 0.192 (0.179-0.204) |
| AdaBoost+NaiveBayes | 19.429 | Rank 3 | 0.741 (0.710-0.770) | 0.349 (0.266-0.425) | 0.651 (0.590-0.712) | 0.438 (0.333-0.541) | 0.778 (0.746-0.807) | 0.197 (0.138-0.266) | 0.209 (0.199-0.218) |
| AdaBoost+DT+NaiveBayes+XGBoost | 21.857 | Rank 4 | 0.718 (0.687-0.749) | 0.328 (0.245-0.406) | 0.613 (0.547-0.677) | 0.423 (0.323-0.533) | 0.754 (0.723-0.786) | 0.170 (0.116-0.235) | 0.191 (0.178-0.204) |
| AdaBoost+DT+SVM+XGBoost | 22.000 | Rank 5 | 0.704 (0.671-0.736) | 0.345 (0.261-0.420) | 0.635 (0.576-0.696) | 0.460 (0.355-0.571) | 0.734 (0.700-0.767) | 0.174 (0.117-0.225) | 0.203 (0.189-0.216) |
| RF+NaiveBayes+SVM+XGBoost+CatBoost | 22.429 | Rank 6 | 0.709 (0.677-0.740) | 0.309 (0.221-0.386) | 0.627 (0.563-0.687) | 0.402 (0.296-0.500) | 0.746 (0.714-0.779) | 0.205 (0.126-0.281) | 0.189 (0.175-0.203) |
| RF+SVM+XGBoost | 22.714 | Rank 7 | 0.687 (0.655-0.716) | 0.350 (0.276-0.420) | 0.650 (0.595-0.705) | 0.480 (0.378-0.583) | 0.712 (0.681-0.746) | 0.186 (0.129-0.249) | 0.200 (0.187-0.213) |
| NaiveBayes | 23.857 | Rank 8 | 0.791 (0.763-0.818) | 0.303 (0.212-0.381) | 0.674 (0.617-0.732) | 0.340 (0.241-0.438) | 0.846 (0.818-0.873) | 0.223 (0.151-0.302) | 0.162 (0.145-0.181) |
| NaiveBayes+SVM+XGBoost+CatBoost | 24.286 | Rank 9 | 0.700 (0.667-0.732) | 0.343 (0.262-0.419) | 0.613 (0.547-0.678) | 0.460 (0.355-0.567) | 0.729 (0.694-0.762) | 0.166 (0.117-0.222) | 0.198 (0.184-0.213) |
| AdaBoost+DT+RF+SVM | 26.000 | Rank 10 | 0.752 (0.720-0.780) | 0.300 (0.214-0.376) | 0.620 (0.550-0.681) | 0.362 (0.258-0.467) | 0.800 (0.769-0.828) | 0.202 (0.116-0.268) | 0.178 (0.167-0.191) |
| NaiveBayes+SVM | 26.429 | Rank 11 | 0.707 (0.677-0.737) | 0.399 (0.316-0.472) | 0.668 (0.611-0.728) | 0.542 (0.438-0.655) | 0.727 (0.696-0.759) | 0.173 (0.127-0.221) | 0.222 (0.212-0.231) |
| RF+XGBoost | 28.714 | Rank 12 | 0.679 (0.648-0.710) | 0.348 (0.271-0.417) | 0.631 (0.568-0.697) | 0.482 (0.375-0.586) | 0.703 (0.672-0.737) | 0.191 (0.128-0.271) | 0.203 (0.189-0.217) |
| AdaBoost+DT+SVM+XGBoost+CatBoost | 31.000 | Rank 13 | 0.691 (0.658-0.724) | 0.341 (0.253-0.415) | 0.608 (0.542-0.669) | 0.463 (0.355-0.571) | 0.719 (0.685-0.754) | 0.150 (0.106-0.198) | 0.199 (0.185-0.214) |
| DT+RF | 32.000 | Rank 14 | 0.717 (0.686-0.748) | 0.297 (0.210-0.371) | 0.605 (0.541-0.673) | 0.379 (0.265-0.484) | 0.758 (0.727-0.792) | 0.183 (0.118-0.245) | 0.184 (0.171-0.197) |
| AdaBoost+NaiveBayes+SVM+XGBoost | 33.714 | Rank 15 | 0.685 (0.653-0.718) | 0.310 (0.224-0.379) | 0.644 (0.586-0.700) | 0.420 (0.310-0.519) | 0.717 (0.686-0.751) | 0.159 (0.112-0.210) | 0.196 (0.182-0.210) |
| DT+RF+XGBoost+CatBoost | 34.286 | Rank 16 | 0.676 (0.644-0.708) | 0.358 (0.264-0.433) | 0.624 (0.560-0.690) | 0.501 (0.375-0.613) | 0.697 (0.662-0.731) | 0.162 (0.111-0.216) | 0.202 (0.186-0.218) |
| RF | 34.286 | Rank 16 | 0.678 (0.648-0.712) | 0.333 (0.254-0.408) | 0.617 (0.558-0.674) | 0.460 (0.353-0.563) | 0.705 (0.674-0.740) | 0.161 (0.111-0.215) | 0.197 (0.188-0.207) |
| DT+RF+SVM+XGBoost | 34.429 | Rank 18 | 0.721 (0.690-0.753) | 0.286 (0.203-0.361) | 0.623 (0.560-0.689) | 0.360 (0.258-0.464) | 0.765 (0.733-0.797) | 0.164 (0.115-0.221) | 0.189 (0.175-0.202) |
| AdaBoost+SVM+XGBoost+CatBoost | 34.714 | Rank 19 | 0.693 (0.662-0.722) | 0.366 (0.278-0.439) | 0.607 (0.548-0.664) | 0.500 (0.387-0.606) | 0.717 (0.685-0.747) | 0.138 (0.103-0.173) | 0.205 (0.191-0.220) |
| AdaBoost+DT+NaiveBayes+SVM+XGBoost | 36.143 | Rank 20 | 0.676 (0.647-0.709) | 0.334 (0.252-0.403) | 0.632 (0.569-0.693) | 0.462 (0.355-0.567) | 0.703 (0.671-0.737) | 0.172 (0.119-0.228) | 0.207 (0.194-0.221) |
| AdaBoost+RF+NaiveBayes+XGBoost | 36.429 | Rank 21 | 0.680 (0.645-0.715) | 0.311 (0.230-0.385) | 0.618 (0.556-0.681) | 0.423 (0.320-0.531) | 0.711 (0.677-0.744) | 0.162 (0.115-0.216) | 0.198 (0.186-0.209) |
| AdaBoost+DT+RF+XGBoost+CatBoost | 36.571 | Rank 22 | 0.695 (0.661-0.727) | 0.316 (0.237-0.392) | 0.604 (0.537-0.665) | 0.420 (0.312-0.531) | 0.729 (0.694-0.762) | 0.157 (0.110-0.206) | 0.200 (0.185-0.216) |
| RF+NaiveBayes+SVM+XGBoost | 38.286 | Rank 23 | 0.708 (0.677-0.740) | 0.293 (0.201-0.367) | 0.599 (0.531-0.661) | 0.377 (0.267-0.471) | 0.749 (0.717-0.781) | 0.185 (0.116-0.244) | 0.197 (0.182-0.211) |
| DT+SVM | 41.714 | Rank 24 | 0.670 (0.638-0.704) | 0.332 (0.247-0.402) | 0.621 (0.566-0.678) | 0.464 (0.345-0.567) | 0.695 (0.661-0.729) | 0.149 (0.109-0.197) | 0.202 (0.191-0.213) |
| AdaBoost+DT+RF+NaiveBayes+SVM+XGBoost | 42.857 | Rank 25 | 0.703 (0.671-0.736) | 0.305 (0.219-0.383) | 0.589 (0.524-0.657) | 0.400 (0.292-0.500) | 0.740 (0.707-0.774) | 0.138 (0.102-0.176) | 0.194 (0.178-0.210) |
| NaiveBayes+XGBoost+CatBoost | 42.857 | Rank 25 | 0.679 (0.648-0.710) | 0.335 (0.247-0.409) | 0.592 (0.529-0.656) | 0.462 (0.353-0.571) | 0.705 (0.673-0.738) | 0.152 (0.105-0.204) | 0.206 (0.191-0.219) |
| RF+NaiveBayes+SVM | 43.000 | Rank 27 | 0.659 (0.628-0.692) | 0.374 (0.295-0.445) | 0.619 (0.551-0.688) | 0.543 (0.437-0.645) | 0.673 (0.639-0.706) | 0.180 (0.124-0.239) | 0.208 (0.195-0.220) |
| DT+RF+SVM+XGBoost+CatBoost | 44.857 | Rank 28 | 0.674 (0.642-0.707) | 0.347 (0.266-0.420) | 0.608 (0.546-0.672) | 0.484 (0.379-0.594) | 0.697 (0.664-0.733) | 0.147 (0.107-0.187) | 0.210 (0.193-0.225) |
| DT+NaiveBayes+SVM | 46.429 | Rank 29 | 0.647 (0.614-0.682) | 0.401 (0.317-0.475) | 0.675 (0.616-0.735) | 0.599 (0.484-0.704) | 0.653 (0.619-0.689) | 0.196 (0.137-0.256) | 0.215 (0.204-0.226) |
| AdaBoost+DT+RF+NaiveBayes+SVM | 46.857 | Rank 30 | 0.681 (0.648-0.714) | 0.310 (0.230-0.385) | 0.599 (0.536-0.659) | 0.422 (0.321-0.531) | 0.712 (0.679-0.746) | 0.142 (0.104-0.184) | 0.203 (0.189-0.218) |
| AdaBoost+RF+SVM+XGBoost | 47.143 | Rank 31 | 0.706 (0.676-0.737) | 0.280 (0.201-0.350) | 0.599 (0.542-0.660) | 0.361 (0.259-0.462) | 0.748 (0.719-0.780) | 0.142 (0.107-0.180) | 0.190 (0.178-0.202) |
| NaiveBayes+XGBoost | 47.714 | Rank 32 | 0.670 (0.635-0.701) | 0.305 (0.226-0.373) | 0.609 (0.545-0.664) | 0.419 (0.312-0.516) | 0.701 (0.667-0.733) | 0.178 (0.115-0.242) | 0.206 (0.193-0.221) |
| DT+NaiveBayes+CatBoost | 47.857 | Rank 33 | 0.672 (0.641-0.705) | 0.306 (0.224-0.375) | 0.604 (0.546-0.670) | 0.422 (0.310-0.517) | 0.703 (0.668-0.740) | 0.145 (0.108-0.184) | 0.198 (0.187-0.208) |
| DT+RF+NaiveBayes+SVM | 48.000 | Rank 34 | 0.682 (0.650-0.715) | 0.295 (0.212-0.366) | 0.606 (0.546-0.667) | 0.397 (0.292-0.500) | 0.717 (0.684-0.751) | 0.144 (0.103-0.186) | 0.198 (0.186-0.210) |
| RF+NaiveBayes | 48.000 | Rank 34 | 0.663 (0.628-0.697) | 0.326 (0.244-0.402) | 0.604 (0.548-0.667) | 0.458 (0.353-0.567) | 0.688 (0.653-0.723) | 0.192 (0.113-0.251) | 0.208 (0.194-0.223) |
| DT+XGBoost | 48.571 | Rank 36 | 0.688 (0.657-0.720) | 0.301 (0.215-0.372) | 0.597 (0.539-0.656) | 0.403 (0.296-0.500) | 0.722 (0.690-0.756) | 0.157 (0.112-0.207) | 0.210 (0.194-0.224) |
| AdaBoost+DT+RF | 49.143 | Rank 37 | 0.711 (0.680-0.739) | 0.267 (0.180-0.342) | 0.601 (0.539-0.658) | 0.340 (0.233-0.440) | 0.756 (0.725-0.784) | 0.139 (0.101-0.176) | 0.191 (0.179-0.203) |
| AdaBoost+DT+XGBoost+CatBoost | 50.286 | Rank 38 | 0.698 (0.667-0.730) | 0.276 (0.190-0.348) | 0.592 (0.526-0.664) | 0.360 (0.257-0.464) | 0.739 (0.706-0.774) | 0.164 (0.113-0.223) | 0.202 (0.185-0.218) |
| AdaBoost+SVM | 50.429 | Rank 39 | 0.650 (0.617-0.683) | 0.319 (0.238-0.391) | 0.644 (0.579-0.704) | 0.457 (0.348-0.567) | 0.674 (0.639-0.708) | 0.175 (0.125-0.231) | 0.206 (0.195-0.217) |
| DT+NaiveBayes+XGBoost+CatBoost | 50.714 | Rank 40 | 0.708 (0.678-0.740) | 0.222 (0.143-0.291) | 0.586 (0.521-0.644) | 0.279 (0.182-0.375) | 0.761 (0.732-0.792) | 0.181 (0.109-0.246) | 0.183 (0.170-0.194) |
| AdaBoost+RF | 50.857 | Rank 41 | 0.671 (0.638-0.702) | 0.304 (0.217-0.374) | 0.610 (0.547-0.670) | 0.418 (0.303-0.517) | 0.702 (0.668-0.737) | 0.167 (0.113-0.226) | 0.209 (0.195-0.223) |
| AdaBoost+SVM+CatBoost | 50.857 | Rank 41 | 0.699 (0.668-0.732) | 0.291 (0.207-0.360) | 0.587 (0.525-0.645) | 0.381 (0.273-0.484) | 0.737 (0.705-0.773) | 0.130 (0.096-0.162) | 0.192 (0.179-0.205) |
| DT+RF+NaiveBayes | 50.857 | Rank 41 | 0.667 (0.635-0.702) | 0.327 (0.243-0.402) | 0.606 (0.542-0.678) | 0.458 (0.345-0.571) | 0.693 (0.660-0.728) | 0.140 (0.104-0.179) | 0.202 (0.188-0.217) |
| DT+RF+SVM | 51.571 | Rank 44 | 0.679 (0.648-0.710) | 0.282 (0.199-0.352) | 0.617 (0.556-0.675) | 0.380 (0.276-0.482) | 0.715 (0.682-0.747) | 0.147 (0.104-0.188) | 0.199 (0.187-0.211) |
| AdaBoost+RF+XGBoost | 51.714 | Rank 45 | 0.689 (0.656-0.720) | 0.285 (0.199-0.359) | 0.580 (0.509-0.650) | 0.378 (0.273-0.483) | 0.727 (0.696-0.759) | 0.152 (0.107-0.203) | 0.196 (0.184-0.208) |
| AdaBoost | 52.857 | Rank 46 | 0.555 (0.521-0.588) | 0.381 (0.309-0.450) | 0.654 (0.600-0.710) | 0.640 (0.538-0.750) | 0.544 (0.509-0.581) | 0.188 (0.130-0.252) | 0.215 (0.206-0.224) |
| AdaBoost+DT | 53.143 | Rank 47 | 0.646 (0.612-0.679) | 0.401 (0.320-0.473) | 0.635 (0.573-0.699) | 0.599 (0.484-0.710) | 0.652 (0.617-0.688) | 0.161 (0.115-0.209) | 0.216 (0.205-0.227) |
| RF+SVM | 53.571 | Rank 48 | 0.664 (0.632-0.696) | 0.302 (0.225-0.373) | 0.605 (0.541-0.665) | 0.420 (0.320-0.520) | 0.694 (0.660-0.728) | 0.164 (0.114-0.216) | 0.204 (0.191-0.216) |
| AdaBoost+XGBoost+CatBoost | 53.857 | Rank 49 | 0.683 (0.651-0.715) | 0.270 (0.192-0.346) | 0.598 (0.536-0.659) | 0.360 (0.257-0.464) | 0.722 (0.690-0.754) | 0.151 (0.109-0.199) | 0.195 (0.182-0.209) |
| XGBoost+CatBoost | 55.429 | Rank 50 | 0.679 (0.646-0.714) | 0.334 (0.247-0.411) | 0.550 (0.484-0.616) | 0.461 (0.355-0.571) | 0.705 (0.672-0.741) | 0.144 (0.095-0.198) | 0.210 (0.194-0.225) |
| AdaBoost+DT+RF+SVM+XGBoost+CatBoost | 56.000 | Rank 51 | 0.723 (0.691-0.755) | 0.257 (0.176-0.333) | 0.578 (0.509-0.642) | 0.320 (0.219-0.417) | 0.772 (0.742-0.803) | 0.136 (0.098-0.175) | 0.184 (0.169-0.199) |
| AdaBoost+RF+SVM+CatBoost | 56.714 | Rank 52 | 0.656 (0.624-0.689) | 0.323 (0.246-0.399) | 0.594 (0.531-0.666) | 0.458 (0.346-0.576) | 0.681 (0.644-0.713) | 0.168 (0.110-0.234) | 0.209 (0.196-0.223) |
| AdaBoost+DT+NaiveBayes | 57.857 | Rank 53 | 0.618 (0.585-0.653) | 0.332 (0.253-0.404) | 0.620 (0.568-0.672) | 0.501 (0.393-0.607) | 0.632 (0.598-0.669) | 0.171 (0.113-0.251) | 0.212 (0.202-0.220) |
| AdaBoost+DT+RF+XGBoost | 58.143 | Rank 54 | 0.689 (0.657-0.722) | 0.287 (0.212-0.359) | 0.593 (0.540-0.647) | 0.381 (0.281-0.484) | 0.727 (0.694-0.761) | 0.131 (0.099-0.164) | 0.207 (0.191-0.223) |
| DT+NaiveBayes | 58.429 | Rank 55 | 0.598 (0.566-0.633) | 0.335 (0.261-0.400) | 0.626 (0.558-0.686) | 0.521 (0.414-0.621) | 0.608 (0.573-0.644) | 0.205 (0.131-0.284) | 0.221 (0.209-0.231) |
| AdaBoost+DT+NaiveBayes+SVM | 58.714 | Rank 56 | 0.618 (0.584-0.652) | 0.354 (0.274-0.426) | 0.633 (0.574-0.696) | 0.539 (0.429-0.645) | 0.627 (0.591-0.664) | 0.190 (0.126-0.271) | 0.229 (0.215-0.242) |
| AdaBoost+RF+NaiveBayes | 58.857 | Rank 57 | 0.663 (0.632-0.697) | 0.316 (0.237-0.388) | 0.586 (0.528-0.650) | 0.442 (0.333-0.556) | 0.690 (0.656-0.725) | 0.142 (0.101-0.184) | 0.207 (0.193-0.219) |
| AdaBoost+NaiveBayes+SVM+XGBoost+CatBoost | 59.286 | Rank 58 | 0.657 (0.625-0.692) | 0.372 (0.286-0.441) | 0.608 (0.539-0.671) | 0.541 (0.428-0.645) | 0.671 (0.636-0.708) | 0.150 (0.108-0.194) | 0.233 (0.213-0.251) |
| RF+CatBoost | 60.857 | Rank 59 | 0.696 (0.666-0.727) | 0.260 (0.169-0.333) | 0.577 (0.512-0.640) | 0.338 (0.219-0.444) | 0.740 (0.709-0.771) | 0.138 (0.097-0.180) | 0.195 (0.183-0.208) |
| DT+RF+XGBoost | 61.571 | Rank 60 | 0.668 (0.635-0.701) | 0.290 (0.214-0.360) | 0.613 (0.554-0.673) | 0.399 (0.294-0.500) | 0.701 (0.668-0.734) | 0.147 (0.107-0.187) | 0.211 (0.195-0.227) |
| AdaBoost+RF+NaiveBayes+SVM+XGBoost | 61.857 | Rank 61 | 0.684 (0.654-0.715) | 0.271 (0.190-0.341) | 0.582 (0.526-0.647) | 0.359 (0.259-0.455) | 0.724 (0.694-0.756) | 0.137 (0.099-0.177) | 0.198 (0.185-0.211) |
| AdaBoost+RF+NaiveBayes+SVM | 62.143 | Rank 62 | 0.671 (0.637-0.704) | 0.294 (0.212-0.367) | 0.583 (0.519-0.646) | 0.402 (0.296-0.500) | 0.704 (0.668-0.738) | 0.135 (0.099-0.170) | 0.202 (0.191-0.215) |
| DT+RF+NaiveBayes+SVM+XGBoost | 62.286 | Rank 63 | 0.653 (0.618-0.687) | 0.336 (0.257-0.410) | 0.603 (0.539-0.666) | 0.482 (0.371-0.588) | 0.674 (0.639-0.714) | 0.158 (0.108-0.210) | 0.227 (0.207-0.246) |
| RF+SVM+CatBoost | 62.571 | Rank 64 | 0.675 (0.644-0.710) | 0.296 (0.214-0.366) | 0.583 (0.523-0.642) | 0.403 (0.296-0.500) | 0.708 (0.673-0.743) | 0.128 (0.096-0.161) | 0.202 (0.188-0.217) |
| CatBoost | 62.714 | Rank 65 | 0.663 (0.629-0.698) | 0.326 (0.250-0.398) | 0.590 (0.527-0.655) | 0.459 (0.353-0.571) | 0.688 (0.653-0.723) | 0.136 (0.102-0.173) | 0.212 (0.196-0.228) |
| AdaBoost+DT+NaiveBayes+SVM+XGBoost+CatBoost | 63.571 | Rank 66 | 0.667 (0.633-0.702) | 0.279 (0.201-0.354) | 0.608 (0.553-0.667) | 0.382 (0.276-0.500) | 0.702 (0.665-0.736) | 0.141 (0.102-0.185) | 0.207 (0.191-0.223) |
| DT | 63.571 | Rank 66 | 0.501 (0.467-0.536) | 0.381 (0.314-0.447) | 0.611 (0.555-0.671) | 0.682 (0.583-0.793) | 0.479 (0.443-0.513) | 0.170 (0.116-0.224) | 0.236 (0.220-0.250) |
| RF+NaiveBayes+SVM+CatBoost | 63.714 | Rank 68 | 0.669 (0.639-0.702) | 0.303 (0.221-0.377) | 0.585 (0.521-0.648) | 0.418 (0.321-0.519) | 0.699 (0.667-0.733) | 0.143 (0.101-0.187) | 0.211 (0.196-0.226) |
| RF+NaiveBayes+XGBoost+CatBoost | 65.286 | Rank 69 | 0.672 (0.639-0.705) | 0.292 (0.209-0.361) | 0.586 (0.525-0.643) | 0.399 (0.290-0.500) | 0.705 (0.672-0.739) | 0.141 (0.101-0.187) | 0.211 (0.194-0.229) |
| AdaBoost+CatBoost | 65.571 | Rank 70 | 0.645 (0.611-0.679) | 0.319 (0.242-0.388) | 0.600 (0.539-0.662) | 0.459 (0.357-0.567) | 0.668 (0.633-0.704) | 0.160 (0.106-0.214) | 0.214 (0.199-0.229) |
| AdaBoost+RF+SVM | 65.857 | Rank 71 | 0.664 (0.633-0.698) | 0.289 (0.206-0.360) | 0.592 (0.529-0.651) | 0.399 (0.294-0.500) | 0.697 (0.664-0.730) | 0.134 (0.096-0.171) | 0.201 (0.190-0.213) |
| AdaBoost+NaiveBayes+SVM | 66.143 | Rank 72 | 0.635 (0.602-0.670) | 0.326 (0.254-0.400) | 0.607 (0.550-0.674) | 0.479 (0.379-0.593) | 0.654 (0.617-0.690) | 0.160 (0.115-0.217) | 0.219 (0.209-0.230) |
| DT+RF+NaiveBayes+SVM+CatBoost | 66.714 | Rank 73 | 0.687 (0.654-0.721) | 0.275 (0.196-0.345) | 0.576 (0.517-0.632) | 0.363 (0.258-0.469) | 0.727 (0.692-0.760) | 0.123 (0.094-0.152) | 0.202 (0.186-0.218) |
| DT+SVM+CatBoost | 68.714 | Rank 74 | 0.687 (0.656-0.720) | 0.285 (0.201-0.360) | 0.558 (0.492-0.621) | 0.380 (0.273-0.487) | 0.724 (0.693-0.758) | 0.123 (0.090-0.155) | 0.205 (0.191-0.218) |
| SVM+XGBoost+CatBoost | 69.286 | Rank 75 | 0.683 (0.652-0.714) | 0.284 (0.205-0.357) | 0.561 (0.499-0.622) | 0.381 (0.276-0.484) | 0.720 (0.686-0.753) | 0.126 (0.092-0.162) | 0.207 (0.192-0.221) |
| AdaBoost+RF+NaiveBayes+SVM+XGBoost+CatBoost | 69.429 | Rank 76 | 0.687 (0.654-0.719) | 0.243 (0.155-0.314) | 0.575 (0.513-0.639) | 0.318 (0.208-0.419) | 0.732 (0.700-0.765) | 0.126 (0.093-0.159) | 0.194 (0.179-0.208) |
| NaiveBayes+SVM+CatBoost | 69.857 | Rank 77 | 0.705 (0.674-0.738) | 0.195 (0.122-0.265) | 0.569 (0.512-0.627) | 0.244 (0.152-0.333) | 0.761 (0.728-0.793) | 0.126 (0.093-0.160) | 0.193 (0.182-0.204) |
| AdaBoost+SVM+XGBoost | 70.143 | Rank 78 | 0.652 (0.621-0.682) | 0.297 (0.220-0.369) | 0.595 (0.535-0.660) | 0.420 (0.321-0.531) | 0.680 (0.649-0.714) | 0.172 (0.112-0.233) | 0.224 (0.211-0.238) |
| DT+RF+NaiveBayes+SVM+XGBoost+CatBoost | 70.143 | Rank 78 | 0.677 (0.642-0.711) | 0.283 (0.199-0.359) | 0.559 (0.491-0.627) | 0.382 (0.276-0.485) | 0.713 (0.678-0.748) | 0.134 (0.093-0.178) | 0.207 (0.191-0.223) |
| AdaBoost+NaiveBayes+SVM+CatBoost | 70.857 | Rank 80 | 0.612 (0.578-0.646) | 0.329 (0.250-0.398) | 0.600 (0.536-0.665) | 0.498 (0.387-0.615) | 0.626 (0.591-0.661) | 0.146 (0.107-0.190) | 0.219 (0.205-0.232) |
| AdaBoost+DT+RF+SVM+CatBoost | 71.286 | Rank 81 | 0.659 (0.625-0.691) | 0.313 (0.237-0.382) | 0.583 (0.525-0.638) | 0.439 (0.333-0.546) | 0.686 (0.649-0.723) | 0.131 (0.098-0.165) | 0.215 (0.200-0.229) |
| DT+RF+CatBoost | 71.714 | Rank 82 | 0.661 (0.628-0.692) | 0.302 (0.228-0.374) | 0.573 (0.515-0.633) | 0.422 (0.323-0.531) | 0.690 (0.657-0.724) | 0.135 (0.100-0.176) | 0.212 (0.197-0.227) |
| AdaBoost+NaiveBayes+XGBoost+CatBoost | 71.857 | Rank 83 | 0.685 (0.651-0.716) | 0.232 (0.154-0.298) | 0.566 (0.502-0.626) | 0.301 (0.206-0.394) | 0.732 (0.699-0.765) | 0.134 (0.097-0.172) | 0.199 (0.184-0.214) |
| RF+XGBoost+CatBoost | 72.429 | Rank 84 | 0.678 (0.645-0.709) | 0.257 (0.174-0.330) | 0.582 (0.519-0.647) | 0.342 (0.233-0.455) | 0.719 (0.687-0.750) | 0.129 (0.096-0.162) | 0.205 (0.190-0.219) |
| AdaBoost+NaiveBayes+XGBoost | 72.571 | Rank 85 | 0.671 (0.641-0.703) | 0.281 (0.205-0.351) | 0.579 (0.522-0.635) | 0.383 (0.278-0.481) | 0.707 (0.673-0.738) | 0.128 (0.094-0.163) | 0.208 (0.196-0.221) |
| AdaBoost+DT+RF+NaiveBayes+SVM+XGBoost+CatBoost | 73.000 | Rank 86 | 0.654 (0.621-0.686) | 0.322 (0.243-0.392) | 0.594 (0.541-0.649) | 0.461 (0.353-0.571) | 0.677 (0.642-0.711) | 0.129 (0.099-0.160) | 0.221 (0.205-0.237) |
| AdaBoost+DT+NaiveBayes+SVM+CatBoost | 73.143 | Rank 87 | 0.681 (0.649-0.714) | 0.296 (0.213-0.366) | 0.557 (0.490-0.622) | 0.400 (0.290-0.500) | 0.715 (0.683-0.749) | 0.125 (0.094-0.159) | 0.214 (0.198-0.232) |
| AdaBoost+DT+RF+NaiveBayes | 73.571 | Rank 88 | 0.658 (0.625-0.693) | 0.299 (0.224-0.370) | 0.579 (0.519-0.637) | 0.417 (0.314-0.529) | 0.688 (0.655-0.723) | 0.134 (0.099-0.170) | 0.209 (0.196-0.223) |
| AdaBoost+DT+RF+NaiveBayes+XGBoost | 76.000 | Rank 89 | 0.652 (0.618-0.685) | 0.297 (0.219-0.370) | 0.595 (0.529-0.657) | 0.420 (0.310-0.529) | 0.680 (0.644-0.715) | 0.134 (0.101-0.170) | 0.220 (0.203-0.236) |
| AdaBoost+RF+CatBoost | 77.286 | Rank 90 | 0.649 (0.616-0.681) | 0.308 (0.228-0.379) | 0.585 (0.525-0.649) | 0.438 (0.333-0.552) | 0.675 (0.640-0.709) | 0.131 (0.097-0.165) | 0.216 (0.200-0.231) |
| RF+SVM+XGBoost+CatBoost | 80.571 | Rank 91 | 0.678 (0.647-0.712) | 0.253 (0.171-0.324) | 0.561 (0.502-0.622) | 0.337 (0.233-0.438) | 0.719 (0.687-0.754) | 0.124 (0.094-0.158) | 0.204 (0.189-0.219) |
| RF+NaiveBayes+CatBoost | 81.429 | Rank 92 | 0.678 (0.646-0.711) | 0.268 (0.190-0.338) | 0.571 (0.514-0.632) | 0.359 (0.257-0.460) | 0.717 (0.685-0.750) | 0.128 (0.094-0.167) | 0.217 (0.200-0.234) |
| LightGBM | 82.143 | Rank 93 | 0.661 (0.629-0.696) | 0.263 (0.183-0.335) | 0.566 (0.498-0.633) | 0.361 (0.250-0.467) | 0.698 (0.665-0.731) | 0.169 (0.106-0.239) | 0.222 (0.202-0.240) |
| AdaBoost+DT+RF+NaiveBayes+CatBoost | 84.571 | Rank 94 | 0.655 (0.623-0.687) | 0.272 (0.189-0.346) | 0.574 (0.515-0.637) | 0.379 (0.269-0.484) | 0.689 (0.654-0.723) | 0.127 (0.094-0.160) | 0.207 (0.194-0.221) |
| AdaBoost+RF+NaiveBayes+SVM+CatBoost | 84.714 | Rank 95 | 0.660 (0.626-0.692) | 0.300 (0.219-0.371) | 0.548 (0.487-0.608) | 0.420 (0.312-0.533) | 0.689 (0.654-0.723) | 0.117 (0.090-0.147) | 0.214 (0.199-0.232) |
| AdaBoost+DT+NaiveBayes+CatBoost | 85.286 | Rank 96 | 0.646 (0.611-0.680) | 0.283 (0.204-0.352) | 0.585 (0.521-0.648) | 0.401 (0.292-0.500) | 0.676 (0.639-0.710) | 0.142 (0.103-0.188) | 0.222 (0.205-0.239) |
| XGBoost | 85.286 | Rank 96 | 0.674 (0.639-0.707) | 0.268 (0.185-0.340) | 0.580 (0.519-0.640) | 0.361 (0.258-0.467) | 0.712 (0.677-0.747) | 0.128 (0.095-0.162) | 0.255 (0.228-0.282) |
| SVM | 85.429 | Rank 98 | 0.630 (0.596-0.662) | 0.324 (0.253-0.396) | 0.568 (0.510-0.634) | 0.479 (0.379-0.588) | 0.648 (0.612-0.682) | 0.130 (0.098-0.166) | 0.227 (0.218-0.238) |
| RF+NaiveBayes+XGBoost | 85.571 | Rank 99 | 0.653 (0.621-0.685) | 0.261 (0.179-0.330) | 0.590 (0.533-0.650) | 0.362 (0.258-0.467) | 0.688 (0.655-0.722) | 0.129 (0.094-0.162) | 0.210 (0.196-0.225) |
| AdaBoost+DT+SVM+CatBoost | 88.000 | Rank 100 | 0.672 (0.638-0.704) | 0.266 (0.187-0.330) | 0.542 (0.478-0.603) | 0.360 (0.258-0.464) | 0.709 (0.673-0.743) | 0.117 (0.085-0.146) | 0.210 (0.196-0.225) |
| AdaBoost+DT+RF+SVM+XGBoost | 88.143 | Rank 101 | 0.655 (0.623-0.687) | 0.248 (0.169-0.318) | 0.562 (0.491-0.623) | 0.342 (0.233-0.441) | 0.693 (0.660-0.727) | 0.137 (0.097-0.181) | 0.207 (0.194-0.221) |
| SVM+XGBoost | 88.143 | Rank 101 | 0.649 (0.618-0.684) | 0.285 (0.209-0.351) | 0.566 (0.502-0.630) | 0.401 (0.303-0.500) | 0.680 (0.646-0.716) | 0.130 (0.099-0.166) | 0.216 (0.203-0.230) |
| DT+RF+NaiveBayes+XGBoost+CatBoost | 89.000 | Rank 103 | 0.650 (0.614-0.685) | 0.296 (0.217-0.368) | 0.565 (0.502-0.625) | 0.419 (0.310-0.519) | 0.679 (0.642-0.714) | 0.122 (0.092-0.153) | 0.219 (0.202-0.236) |
| SVM+CatBoost | 89.857 | Rank 104 | 0.644 (0.611-0.680) | 0.306 (0.223-0.376) | 0.548 (0.484-0.616) | 0.440 (0.333-0.546) | 0.669 (0.632-0.707) | 0.121 (0.089-0.155) | 0.216 (0.201-0.231) |
| NaiveBayes+CatBoost | 90.143 | Rank 105 | 0.677 (0.643-0.710) | 0.254 (0.170-0.329) | 0.541 (0.481-0.602) | 0.340 (0.233-0.438) | 0.717 (0.684-0.753) | 0.111 (0.085-0.139) | 0.211 (0.195-0.228) |
| AdaBoost+DT+XGBoost | 92.286 | Rank 106 | 0.636 (0.603-0.669) | 0.265 (0.192-0.331) | 0.577 (0.517-0.640) | 0.378 (0.273-0.483) | 0.667 (0.632-0.703) | 0.149 (0.105-0.198) | 0.216 (0.204-0.227) |
| DT+NaiveBayes+SVM+XGBoost+CatBoost | 92.429 | Rank 107 | 0.670 (0.637-0.704) | 0.238 (0.159-0.310) | 0.543 (0.479-0.606) | 0.319 (0.214-0.419) | 0.713 (0.678-0.748) | 0.117 (0.089-0.146) | 0.209 (0.194-0.225) |
| DT+SVM+XGBoost+CatBoost | 92.714 | Rank 108 | 0.666 (0.634-0.700) | 0.263 (0.184-0.338) | 0.540 (0.479-0.605) | 0.360 (0.250-0.467) | 0.703 (0.669-0.737) | 0.119 (0.086-0.152) | 0.213 (0.196-0.230) |
| DT+RF+NaiveBayes+XGBoost | 93.429 | Rank 109 | 0.650 (0.619-0.684) | 0.296 (0.211-0.372) | 0.554 (0.488-0.622) | 0.419 (0.308-0.533) | 0.678 (0.647-0.711) | 0.120 (0.090-0.152) | 0.225 (0.207-0.240) |
| AdaBoost+RF+SVM+XGBoost+CatBoost | 94.571 | Rank 110 | 0.660 (0.627-0.693) | 0.249 (0.171-0.320) | 0.567 (0.504-0.622) | 0.340 (0.233-0.438) | 0.699 (0.664-0.735) | 0.120 (0.092-0.150) | 0.214 (0.196-0.231) |
| DT+NaiveBayes+SVM+XGBoost | 97.143 | Rank 111 | 0.650 (0.618-0.683) | 0.205 (0.138-0.275) | 0.539 (0.476-0.607) | 0.279 (0.182-0.379) | 0.695 (0.663-0.730) | 0.154 (0.101-0.224) | 0.214 (0.201-0.227) |
| AdaBoost+DT+SVM | 98.286 | Rank 112 | 0.628 (0.595-0.663) | 0.266 (0.190-0.333) | 0.570 (0.508-0.632) | 0.383 (0.276-0.486) | 0.658 (0.624-0.697) | 0.132 (0.096-0.175) | 0.221 (0.209-0.233) |
| AdaBoost+RF+XGBoost+CatBoost | 98.857 | Rank 113 | 0.639 (0.603-0.674) | 0.268 (0.190-0.338) | 0.565 (0.492-0.630) | 0.382 (0.276-0.485) | 0.671 (0.635-0.707) | 0.130 (0.095-0.168) | 0.224 (0.207-0.241) |
| AdaBoost+NaiveBayes+CatBoost | 100.571 | Rank 114 | 0.628 (0.594-0.661) | 0.261 (0.178-0.333) | 0.579 (0.519-0.642) | 0.376 (0.258-0.484) | 0.659 (0.623-0.695) | 0.122 (0.092-0.154) | 0.214 (0.199-0.228) |
| AdaBoost+DT+NaiveBayes+XGBoost+CatBoost | 100.857 | Rank 115 | 0.641 (0.611-0.675) | 0.268 (0.187-0.335) | 0.564 (0.504-0.626) | 0.379 (0.267-0.482) | 0.674 (0.640-0.708) | 0.130 (0.095-0.167) | 0.233 (0.217-0.248) |
| DT+NaiveBayes+XGBoost | 101.714 | Rank 116 | 0.652 (0.620-0.686) | 0.233 (0.158-0.300) | 0.575 (0.517-0.631) | 0.320 (0.219-0.417) | 0.693 (0.659-0.727) | 0.126 (0.094-0.159) | 0.223 (0.207-0.239) |
| AdaBoost+DT+RF+CatBoost | 102.000 | Rank 117 | 0.660 (0.627-0.691) | 0.222 (0.146-0.292) | 0.552 (0.494-0.611) | 0.301 (0.200-0.400) | 0.703 (0.672-0.738) | 0.114 (0.085-0.144) | 0.216 (0.199-0.231) |
| AdaBoost+RF+NaiveBayes+XGBoost+CatBoost | 102.857 | Rank 118 | 0.660 (0.626-0.693) | 0.237 (0.161-0.307) | 0.527 (0.466-0.590) | 0.321 (0.222-0.429) | 0.702 (0.668-0.735) | 0.116 (0.083-0.148) | 0.216 (0.201-0.230) |
| AdaBoost+DT+CatBoost | 104.143 | Rank 119 | 0.636 (0.600-0.671) | 0.280 (0.200-0.349) | 0.542 (0.477-0.602) | 0.402 (0.300-0.500) | 0.664 (0.628-0.700) | 0.118 (0.086-0.151) | 0.227 (0.209-0.244) |
| AdaBoost+DT+RF+NaiveBayes+SVM+CatBoost | 105.714 | Rank 120 | 0.642 (0.609-0.674) | 0.241 (0.167-0.304) | 0.554 (0.490-0.613) | 0.338 (0.235-0.444) | 0.678 (0.645-0.713) | 0.121 (0.090-0.151) | 0.213 (0.199-0.226) |
| DT+CatBoost | 106.286 | Rank 121 | 0.597 (0.563-0.633) | 0.254 (0.181-0.324) | 0.550 (0.489-0.612) | 0.381 (0.273-0.500) | 0.624 (0.588-0.662) | 0.133 (0.094-0.182) | 0.221 (0.209-0.234) |
| DT+RF+SVM+CatBoost | 106.429 | Rank 122 | 0.644 (0.611-0.677) | 0.230 (0.155-0.296) | 0.562 (0.500-0.625) | 0.320 (0.219-0.417) | 0.683 (0.649-0.718) | 0.125 (0.092-0.158) | 0.220 (0.202-0.236) |
| DT+NaiveBayes+SVM+CatBoost | 107.429 | Rank 123 | 0.641 (0.609-0.677) | 0.242 (0.168-0.314) | 0.528 (0.466-0.591) | 0.340 (0.241-0.441) | 0.678 (0.645-0.714) | 0.124 (0.089-0.165) | 0.214 (0.198-0.228) |
| AdaBoost+DT+RF+NaiveBayes+XGBoost+CatBoost | 110.143 | Rank 124 | 0.622 (0.589-0.655) | 0.261 (0.185-0.329) | 0.556 (0.492-0.616) | 0.380 (0.269-0.483) | 0.652 (0.617-0.687) | 0.123 (0.090-0.156) | 0.241 (0.223-0.259) |
| DT+XGBoost+CatBoost | 111.571 | Rank 125 | 0.650 (0.618-0.684) | 0.206 (0.136-0.275) | 0.533 (0.472-0.598) | 0.281 (0.185-0.382) | 0.695 (0.662-0.729) | 0.114 (0.089-0.143) | 0.223 (0.205-0.240) |
| AdaBoost+RF+NaiveBayes+CatBoost | 111.714 | Rank 126 | 0.642 (0.608-0.674) | 0.256 (0.177-0.332) | 0.534 (0.476-0.596) | 0.361 (0.258-0.471) | 0.676 (0.641-0.709) | 0.115 (0.085-0.147) | 0.228 (0.211-0.246) |
| DT+RF+NaiveBayes+CatBoost | 117.714 | Rank 127 | 0.630 (0.597-0.664) | 0.214 (0.141-0.281) | 0.522 (0.467-0.577) | 0.301 (0.200-0.400) | 0.670 (0.636-0.706) | 0.108 (0.082-0.135) | 0.216 (0.201-0.231) |
| DT+SVM+XGBoost | 118.286 | Rank 128 | 0.639 (0.607-0.670) | 0.201 (0.129-0.267) | 0.521 (0.461-0.580) | 0.278 (0.182-0.370) | 0.683 (0.647-0.718) | 0.118 (0.082-0.158) | 0.229 (0.215-0.243) |
| KNN | 127.286 | Rank 129 | 0.574 (0.541-0.610) | 0.198 (0.132-0.262) | 0.499 (0.433-0.562) | 0.300 (0.200-0.395) | 0.608 (0.571-0.643) | 0.113 (0.080-0.146) | 0.292 (0.271-0.313) |

**Supplementary Table 20.** Ranking of 129 machine learning models in the CHARLS training set.

| **Model** | **Normalized Rank Score** | **Comprehensive Rank** | **Accuracy (95% CI)** | **F_Beta (95% CI)** | **ROC_AUC (95% CI)** | **Sensitivity (95% CI)** | **Specificity (95% CI)** | **PR_AUC (95% CI)** | **Brier (95% CI)** |
| --- | --- | --- | --- | --- | --- | --- | --- | --- | --- |
| AdaBoost+DT+RF+LightGBM | 3.857 | Rank 1 | 0.830 (0.818-0.841) | 0.775 (0.754-0.795) | 0.947 (0.941-0.954) | 0.963 (0.948-0.980) | 0.809 (0.797-0.822) | 0.715 (0.677-0.751) | 0.133 (0.129-0.138) |
| DT+RF+LightGBM+NaiveBayes+SVM+CatBoost | 6.857 | Rank 2 | 0.833 (0.822-0.845) | 0.774 (0.753-0.794) | 0.940 (0.933-0.947) | 0.955 (0.938-0.972) | 0.815 (0.802-0.827) | 0.645 (0.599-0.689) | 0.132 (0.128-0.137) |
| AdaBoost+RF+LightGBM+SVM+CatBoost | 8.571 | Rank 3 | 0.826 (0.814-0.836) | 0.758 (0.738-0.778) | 0.944 (0.936-0.951) | 0.940 (0.920-0.959) | 0.808 (0.796-0.820) | 0.686 (0.641-0.732) | 0.132 (0.128-0.137) |
| AdaBoost+RF+LightGBM+SVM | 9.000 | Rank 4 | 0.826 (0.815-0.837) | 0.765 (0.744-0.785) | 0.944 (0.937-0.951) | 0.952 (0.935-0.971) | 0.807 (0.794-0.820) | 0.656 (0.615-0.698) | 0.133 (0.128-0.137) |
| AdaBoost+RF+LightGBM+NaiveBayes+SVM | 10.571 | Rank 5 | 0.815 (0.803-0.826) | 0.764 (0.742-0.784) | 0.943 (0.937-0.951) | 0.967 (0.953-0.982) | 0.792 (0.779-0.805) | 0.694 (0.656-0.732) | 0.135 (0.130-0.140) |
| AdaBoost+RF+LightGBM+CatBoost | 15.286 | Rank 6 | 0.816 (0.804-0.827) | 0.750 (0.728-0.771) | 0.938 (0.930-0.946) | 0.942 (0.923-0.962) | 0.796 (0.783-0.809) | 0.671 (0.628-0.712) | 0.134 (0.129-0.139) |
| RF+LightGBM+NaiveBayes+SVM | 17.429 | Rank 7 | 0.818 (0.806-0.830) | 0.746 (0.722-0.767) | 0.940 (0.932-0.947) | 0.931 (0.910-0.954) | 0.801 (0.789-0.814) | 0.666 (0.623-0.710) | 0.139 (0.134-0.143) |
| RF+LightGBM+SVM | 17.571 | Rank 8 | 0.819 (0.808-0.830) | 0.753 (0.733-0.774) | 0.937 (0.930-0.945) | 0.942 (0.924-0.961) | 0.800 (0.787-0.812) | 0.660 (0.620-0.703) | 0.140 (0.135-0.144) |
| DT+RF+LightGBM+NaiveBayes | 18.143 | Rank 9 | 0.819 (0.808-0.830) | 0.758 (0.737-0.779) | 0.938 (0.930-0.945) | 0.951 (0.934-0.970) | 0.798 (0.786-0.812) | 0.633 (0.589-0.680) | 0.140 (0.135-0.145) |
| AdaBoost+DT+RF+LightGBM+NaiveBayes+SVM | 18.429 | Rank 10 | 0.809 (0.796-0.821) | 0.758 (0.735-0.779) | 0.942 (0.934-0.949) | 0.966 (0.952-0.981) | 0.785 (0.771-0.798) | 0.656 (0.611-0.703) | 0.136 (0.130-0.141) |
| DT+RF+LightGBM | 20.571 | Rank 11 | 0.814 (0.803-0.825) | 0.752 (0.731-0.772) | 0.937 (0.929-0.944) | 0.948 (0.930-0.967) | 0.793 (0.780-0.806) | 0.641 (0.600-0.685) | 0.139 (0.134-0.143) |
| AdaBoost+DT+RF+LightGBM+NaiveBayes+SVM+CatBoost | 20.857 | Rank 12 | 0.814 (0.802-0.825) | 0.747 (0.724-0.769) | 0.934 (0.927-0.942) | 0.939 (0.919-0.960) | 0.795 (0.782-0.808) | 0.656 (0.615-0.696) | 0.138 (0.134-0.143) |
| AdaBoost+DT+RF | 21.286 | Rank 13 | 0.829 (0.817-0.840) | 0.763 (0.741-0.785) | 0.930 (0.921-0.939) | 0.942 (0.924-0.962) | 0.812 (0.799-0.824) | 0.632 (0.587-0.675) | 0.144 (0.140-0.149) |
| AdaBoost+RF+LightGBM+NaiveBayes | 21.857 | Rank 14 | 0.806 (0.794-0.819) | 0.759 (0.739-0.779) | 0.940 (0.933-0.948) | 0.973 (0.961-0.986) | 0.780 (0.767-0.795) | 0.676 (0.636-0.715) | 0.141 (0.136-0.145) |
| DT+RF+LightGBM+NaiveBayes+SVM | 22.286 | Rank 15 | 0.804 (0.793-0.815) | 0.752 (0.731-0.771) | 0.944 (0.937-0.951) | 0.964 (0.947-0.981) | 0.780 (0.767-0.792) | 0.679 (0.640-0.721) | 0.139 (0.134-0.143) |
| AdaBoost+DT+RF+LightGBM+SVM+CatBoost | 22.714 | Rank 16 | 0.810 (0.799-0.822) | 0.733 (0.712-0.756) | 0.934 (0.925-0.943) | 0.921 (0.899-0.944) | 0.793 (0.780-0.806) | 0.692 (0.655-0.729) | 0.135 (0.131-0.140) |
| DT+RF+LightGBM+SVM | 23.143 | Rank 17 | 0.811 (0.799-0.823) | 0.748 (0.726-0.769) | 0.935 (0.927-0.942) | 0.945 (0.927-0.965) | 0.791 (0.777-0.804) | 0.629 (0.586-0.673) | 0.137 (0.132-0.142) |
| DT+LightGBM+SVM | 24.714 | Rank 18 | 0.832 (0.822-0.843) | 0.768 (0.746-0.788) | 0.923 (0.914-0.931) | 0.945 (0.926-0.964) | 0.815 (0.804-0.827) | 0.550 (0.506-0.594) | 0.142 (0.138-0.146) |
| RF+LightGBM+CatBoost | 25.857 | Rank 19 | 0.823 (0.812-0.835) | 0.728 (0.702-0.751) | 0.932 (0.923-0.941) | 0.890 (0.865-0.916) | 0.813 (0.801-0.826) | 0.666 (0.623-0.709) | 0.136 (0.131-0.140) |
| LightGBM+NaiveBayes+SVM | 26.286 | Rank 20 | 0.832 (0.821-0.843) | 0.769 (0.746-0.789) | 0.924 (0.916-0.932) | 0.948 (0.930-0.967) | 0.814 (0.802-0.826) | 0.532 (0.489-0.576) | 0.142 (0.138-0.146) |
| AdaBoost+DT+LightGBM+SVM+CatBoost | 27.000 | Rank 21 | 0.817 (0.806-0.829) | 0.736 (0.712-0.760) | 0.926 (0.916-0.935) | 0.915 (0.892-0.939) | 0.803 (0.790-0.815) | 0.616 (0.569-0.661) | 0.136 (0.131-0.141) |
| AdaBoost+DT+RF+LightGBM+SVM | 27.714 | Rank 22 | 0.804 (0.792-0.816) | 0.740 (0.717-0.761) | 0.938 (0.930-0.946) | 0.942 (0.923-0.961) | 0.783 (0.769-0.797) | 0.682 (0.639-0.722) | 0.142 (0.137-0.146) |
| RF+LightGBM | 28.143 | Rank 23 | 0.811 (0.799-0.823) | 0.748 (0.725-0.769) | 0.933 (0.925-0.941) | 0.945 (0.928-0.963) | 0.790 (0.778-0.803) | 0.639 (0.592-0.683) | 0.145 (0.140-0.149) |
| AdaBoost+LightGBM+NaiveBayes+SVM+CatBoost | 28.714 | Rank 24 | 0.822 (0.810-0.834) | 0.733 (0.709-0.756) | 0.923 (0.914-0.932) | 0.902 (0.878-0.929) | 0.810 (0.797-0.823) | 0.613 (0.570-0.655) | 0.137 (0.132-0.141) |
| AdaBoost+DT+RF+LightGBM+NaiveBayes | 29.571 | Rank 25 | 0.805 (0.793-0.817) | 0.753 (0.730-0.774) | 0.933 (0.925-0.942) | 0.963 (0.948-0.980) | 0.781 (0.768-0.794) | 0.639 (0.596-0.682) | 0.144 (0.139-0.149) |
| AdaBoost+RF+LightGBM | 30.000 | Rank 26 | 0.799 (0.786-0.811) | 0.744 (0.721-0.765) | 0.939 (0.931-0.946) | 0.957 (0.941-0.975) | 0.775 (0.761-0.788) | 0.673 (0.629-0.718) | 0.142 (0.136-0.147) |
| RF+LightGBM+NaiveBayes+SVM+CatBoost | 30.143 | Rank 27 | 0.813 (0.801-0.825) | 0.739 (0.716-0.762) | 0.926 (0.917-0.934) | 0.927 (0.905-0.949) | 0.795 (0.782-0.809) | 0.596 (0.552-0.641) | 0.139 (0.134-0.144) |
| AdaBoost+DT+LightGBM+CatBoost | 30.857 | Rank 28 | 0.825 (0.814-0.837) | 0.737 (0.712-0.760) | 0.919 (0.910-0.927) | 0.903 (0.877-0.930) | 0.813 (0.800-0.825) | 0.575 (0.532-0.617) | 0.135 (0.131-0.140) |
| RF+LightGBM+NaiveBayes | 30.857 | Rank 28 | 0.810 (0.797-0.821) | 0.745 (0.721-0.766) | 0.932 (0.924-0.940) | 0.943 (0.925-0.961) | 0.789 (0.776-0.803) | 0.602 (0.555-0.650) | 0.141 (0.136-0.146) |
| AdaBoost+RF+LightGBM+NaiveBayes+CatBoost | 31.286 | Rank 30 | 0.809 (0.797-0.820) | 0.729 (0.706-0.752) | 0.929 (0.920-0.937) | 0.915 (0.893-0.939) | 0.793 (0.779-0.806) | 0.636 (0.593-0.681) | 0.137 (0.132-0.142) |
| AdaBoost+RF+SVM | 31.286 | Rank 30 | 0.805 (0.793-0.817) | 0.731 (0.708-0.752) | 0.935 (0.926-0.943) | 0.923 (0.902-0.947) | 0.787 (0.774-0.801) | 0.654 (0.610-0.699) | 0.141 (0.136-0.145) |
| DT+RF+LightGBM+NaiveBayes+CatBoost | 31.857 | Rank 32 | 0.800 (0.789-0.812) | 0.739 (0.715-0.760) | 0.933 (0.924-0.941) | 0.945 (0.927-0.964) | 0.778 (0.766-0.791) | 0.679 (0.638-0.716) | 0.142 (0.138-0.147) |
| AdaBoost+RF+SVM+CatBoost | 33.286 | Rank 33 | 0.810 (0.799-0.822) | 0.718 (0.694-0.741) | 0.926 (0.917-0.936) | 0.894 (0.870-0.920) | 0.797 (0.785-0.810) | 0.672 (0.632-0.707) | 0.138 (0.133-0.142) |
| RF+LightGBM+NaiveBayes+CatBoost | 33.429 | Rank 34 | 0.806 (0.795-0.819) | 0.725 (0.703-0.748) | 0.927 (0.919-0.936) | 0.913 (0.891-0.936) | 0.790 (0.777-0.804) | 0.655 (0.615-0.692) | 0.138 (0.133-0.143) |
| DT+RF+LightGBM+SVM+CatBoost | 34.857 | Rank 35 | 0.809 (0.796-0.821) | 0.736 (0.713-0.757) | 0.919 (0.910-0.928) | 0.927 (0.906-0.948) | 0.791 (0.777-0.804) | 0.608 (0.565-0.648) | 0.141 (0.136-0.146) |
| LightGBM+NaiveBayes | 35.857 | Rank 36 | 0.835 (0.825-0.845) | 0.746 (0.722-0.769) | 0.919 (0.911-0.927) | 0.903 (0.880-0.931) | 0.825 (0.813-0.837) | 0.539 (0.497-0.587) | 0.144 (0.141-0.148) |
| AdaBoost+LightGBM+NaiveBayes+CatBoost | 38.429 | Rank 37 | 0.821 (0.810-0.833) | 0.721 (0.697-0.745) | 0.918 (0.909-0.927) | 0.882 (0.855-0.912) | 0.812 (0.798-0.825) | 0.583 (0.537-0.625) | 0.137 (0.133-0.142) |
| DT+RF+LightGBM+CatBoost | 39.429 | Rank 38 | 0.805 (0.793-0.817) | 0.730 (0.708-0.754) | 0.924 (0.915-0.933) | 0.924 (0.902-0.947) | 0.787 (0.774-0.800) | 0.590 (0.544-0.633) | 0.142 (0.136-0.147) |
| DT+LightGBM+NaiveBayes+SVM+CatBoost | 40.286 | Rank 39 | 0.814 (0.803-0.825) | 0.733 (0.710-0.755) | 0.915 (0.906-0.925) | 0.915 (0.892-0.939) | 0.799 (0.787-0.811) | 0.566 (0.523-0.611) | 0.143 (0.138-0.147) |
| AdaBoost+RF+NaiveBayes+CatBoost | 40.429 | Rank 40 | 0.804 (0.792-0.816) | 0.715 (0.689-0.738) | 0.921 (0.911-0.931) | 0.897 (0.872-0.923) | 0.790 (0.777-0.803) | 0.659 (0.620-0.701) | 0.139 (0.134-0.144) |
| AdaBoost+RF+NaiveBayes+SVM+CatBoost | 41.143 | Rank 41 | 0.801 (0.789-0.813) | 0.726 (0.701-0.749) | 0.919 (0.910-0.929) | 0.921 (0.899-0.943) | 0.783 (0.770-0.796) | 0.641 (0.600-0.679) | 0.142 (0.137-0.147) |
| AdaBoost+DT+RF+NaiveBayes+CatBoost | 41.429 | Rank 42 | 0.804 (0.793-0.816) | 0.727 (0.702-0.749) | 0.923 (0.913-0.932) | 0.918 (0.896-0.940) | 0.787 (0.774-0.801) | 0.639 (0.600-0.682) | 0.147 (0.142-0.152) |
| AdaBoost+DT+LightGBM+NaiveBayes | 44.000 | Rank 43 | 0.821 (0.809-0.832) | 0.733 (0.709-0.755) | 0.916 (0.908-0.925) | 0.903 (0.880-0.928) | 0.808 (0.796-0.821) | 0.532 (0.486-0.577) | 0.146 (0.142-0.151) |
| AdaBoost+RF+NaiveBayes+SVM | 45.286 | Rank 44 | 0.789 (0.777-0.802) | 0.727 (0.704-0.750) | 0.931 (0.923-0.940) | 0.942 (0.924-0.963) | 0.765 (0.752-0.780) | 0.665 (0.623-0.705) | 0.146 (0.141-0.151) |
| AdaBoost+DT+RF+LightGBM+CatBoost | 45.429 | Rank 45 | 0.809 (0.797-0.820) | 0.713 (0.689-0.738) | 0.914 (0.904-0.924) | 0.887 (0.860-0.917) | 0.797 (0.784-0.809) | 0.632 (0.592-0.673) | 0.141 (0.136-0.145) |
| AdaBoost+DT+RF+CatBoost | 46.429 | Rank 46 | 0.800 (0.788-0.812) | 0.709 (0.685-0.733) | 0.922 (0.913-0.932) | 0.893 (0.868-0.920) | 0.786 (0.773-0.800) | 0.653 (0.614-0.693) | 0.139 (0.134-0.144) |
| AdaBoost+DT+LightGBM+NaiveBayes+CatBoost | 49.000 | Rank 47 | 0.814 (0.803-0.825) | 0.729 (0.704-0.752) | 0.908 (0.899-0.918) | 0.906 (0.883-0.932) | 0.800 (0.788-0.813) | 0.536 (0.494-0.577) | 0.148 (0.143-0.152) |
| AdaBoost+LightGBM+SVM | 49.286 | Rank 48 | 0.816 (0.804-0.828) | 0.725 (0.700-0.748) | 0.917 (0.908-0.925) | 0.896 (0.872-0.922) | 0.804 (0.791-0.816) | 0.539 (0.492-0.582) | 0.148 (0.144-0.153) |
| AdaBoost+RF+NaiveBayes | 50.286 | Rank 49 | 0.788 (0.777-0.801) | 0.713 (0.689-0.736) | 0.930 (0.922-0.938) | 0.918 (0.896-0.943) | 0.768 (0.755-0.782) | 0.683 (0.645-0.719) | 0.149 (0.144-0.153) |
| AdaBoost+LightGBM+SVM+CatBoost | 52.857 | Rank 50 | 0.804 (0.792-0.816) | 0.698 (0.673-0.723) | 0.913 (0.903-0.923) | 0.869 (0.842-0.896) | 0.794 (0.781-0.806) | 0.598 (0.556-0.642) | 0.138 (0.134-0.143) |
| AdaBoost+RF+LightGBM+NaiveBayes+SVM+CatBoost | 53.000 | Rank 51 | 0.795 (0.783-0.806) | 0.719 (0.694-0.742) | 0.919 (0.910-0.928) | 0.918 (0.896-0.940) | 0.776 (0.763-0.789) | 0.589 (0.541-0.634) | 0.145 (0.140-0.150) |
| LightGBM+SVM+CatBoost | 53.000 | Rank 51 | 0.801 (0.790-0.812) | 0.720 (0.695-0.742) | 0.915 (0.905-0.924) | 0.912 (0.890-0.935) | 0.784 (0.772-0.797) | 0.588 (0.540-0.631) | 0.148 (0.143-0.152) |
| RF+NaiveBayes | 53.143 | Rank 53 | 0.797 (0.785-0.809) | 0.720 (0.696-0.742) | 0.914 (0.903-0.924) | 0.918 (0.895-0.941) | 0.778 (0.765-0.793) | 0.608 (0.566-0.648) | 0.148 (0.143-0.153) |
| DT+LightGBM+SVM+CatBoost | 53.857 | Rank 54 | 0.803 (0.792-0.815) | 0.705 (0.680-0.731) | 0.917 (0.906-0.927) | 0.881 (0.855-0.908) | 0.791 (0.779-0.803) | 0.593 (0.546-0.639) | 0.143 (0.138-0.148) |
| RF+SVM | 56.286 | Rank 55 | 0.792 (0.780-0.804) | 0.721 (0.697-0.743) | 0.923 (0.913-0.933) | 0.926 (0.905-0.948) | 0.771 (0.757-0.786) | 0.631 (0.588-0.675) | 0.158 (0.153-0.162) |
| DT+RF+SVM+CatBoost | 57.143 | Rank 56 | 0.796 (0.784-0.809) | 0.708 (0.682-0.731) | 0.910 (0.900-0.922) | 0.896 (0.872-0.922) | 0.781 (0.767-0.795) | 0.614 (0.572-0.657) | 0.143 (0.138-0.147) |
| DT+LightGBM | 57.857 | Rank 57 | 0.794 (0.782-0.805) | 0.738 (0.716-0.758) | 0.918 (0.909-0.926) | 0.954 (0.937-0.971) | 0.769 (0.756-0.782) | 0.517 (0.475-0.562) | 0.153 (0.148-0.158) |
| LightGBM+CatBoost | 58.857 | Rank 58 | 0.797 (0.785-0.809) | 0.710 (0.686-0.734) | 0.914 (0.905-0.925) | 0.900 (0.875-0.925) | 0.781 (0.768-0.794) | 0.603 (0.561-0.648) | 0.149 (0.144-0.153) |
| DT+LightGBM+NaiveBayes+SVM | 59.571 | Rank 59 | 0.807 (0.795-0.819) | 0.722 (0.696-0.745) | 0.910 (0.901-0.919) | 0.906 (0.881-0.931) | 0.792 (0.779-0.804) | 0.520 (0.477-0.566) | 0.155 (0.151-0.160) |
| AdaBoost+DT+RF+LightGBM+NaiveBayes+CatBoost | 59.857 | Rank 60 | 0.789 (0.777-0.801) | 0.702 (0.677-0.726) | 0.918 (0.909-0.928) | 0.897 (0.873-0.922) | 0.773 (0.759-0.787) | 0.632 (0.592-0.671) | 0.146 (0.141-0.150) |
| AdaBoost+DT+LightGBM+NaiveBayes+SVM+CatBoost | 60.571 | Rank 61 | 0.797 (0.786-0.810) | 0.709 (0.682-0.732) | 0.909 (0.898-0.919) | 0.897 (0.872-0.922) | 0.782 (0.770-0.796) | 0.579 (0.532-0.621) | 0.146 (0.141-0.151) |
| DT+RF+CatBoost | 61.714 | Rank 62 | 0.795 (0.783-0.807) | 0.703 (0.678-0.727) | 0.911 (0.900-0.921) | 0.890 (0.865-0.917) | 0.780 (0.767-0.794) | 0.605 (0.563-0.649) | 0.147 (0.142-0.152) |
| AdaBoost+DT+RF+NaiveBayes+SVM | 62.000 | Rank 63 | 0.781 (0.769-0.793) | 0.713 (0.689-0.735) | 0.921 (0.912-0.930) | 0.927 (0.905-0.950) | 0.759 (0.746-0.773) | 0.619 (0.574-0.661) | 0.154 (0.149-0.159) |
| AdaBoost+DT+RF+NaiveBayes+SVM+CatBoost | 63.857 | Rank 64 | 0.784 (0.771-0.797) | 0.694 (0.670-0.717) | 0.920 (0.910-0.929) | 0.891 (0.867-0.917) | 0.767 (0.754-0.782) | 0.652 (0.612-0.691) | 0.145 (0.141-0.150) |
| AdaBoost+LightGBM | 64.000 | Rank 65 | 0.802 (0.791-0.815) | 0.723 (0.700-0.748) | 0.910 (0.901-0.919) | 0.915 (0.893-0.938) | 0.785 (0.773-0.798) | 0.513 (0.470-0.557) | 0.160 (0.156-0.164) |
| AdaBoost+RF | 64.000 | Rank 65 | 0.786 (0.773-0.798) | 0.715 (0.692-0.737) | 0.919 (0.910-0.928) | 0.927 (0.907-0.949) | 0.764 (0.751-0.778) | 0.587 (0.544-0.631) | 0.154 (0.149-0.159) |
| DT+RF+NaiveBayes+SVM | 64.143 | Rank 67 | 0.791 (0.778-0.803) | 0.707 (0.681-0.730) | 0.916 (0.906-0.926) | 0.903 (0.879-0.928) | 0.774 (0.761-0.788) | 0.612 (0.570-0.656) | 0.155 (0.151-0.160) |
| AdaBoost+LightGBM+CatBoost | 64.429 | Rank 68 | 0.797 (0.785-0.808) | 0.712 (0.687-0.736) | 0.909 (0.900-0.920) | 0.903 (0.878-0.927) | 0.781 (0.767-0.793) | 0.541 (0.496-0.584) | 0.149 (0.145-0.154) |
| DT+LightGBM+CatBoost | 67.000 | Rank 69 | 0.799 (0.787-0.810) | 0.689 (0.663-0.715) | 0.903 (0.891-0.914) | 0.860 (0.833-0.888) | 0.789 (0.777-0.802) | 0.563 (0.517-0.607) | 0.146 (0.142-0.151) |
| AdaBoost+LightGBM+NaiveBayes | 68.857 | Rank 70 | 0.794 (0.782-0.805) | 0.718 (0.695-0.740) | 0.910 (0.900-0.919) | 0.919 (0.896-0.940) | 0.775 (0.762-0.788) | 0.511 (0.469-0.554) | 0.158 (0.153-0.162) |
| LightGBM+NaiveBayes+SVM+CatBoost | 69.286 | Rank 71 | 0.792 (0.780-0.804) | 0.701 (0.675-0.725) | 0.908 (0.897-0.917) | 0.891 (0.866-0.917) | 0.777 (0.764-0.790) | 0.558 (0.515-0.600) | 0.150 (0.145-0.155) |
| AdaBoost+RF+CatBoost | 71.429 | Rank 72 | 0.776 (0.764-0.789) | 0.696 (0.672-0.720) | 0.910 (0.899-0.921) | 0.906 (0.882-0.930) | 0.756 (0.742-0.771) | 0.632 (0.591-0.668) | 0.150 (0.145-0.155) |
| DT+LightGBM+NaiveBayes | 71.857 | Rank 73 | 0.804 (0.792-0.817) | 0.697 (0.671-0.721) | 0.897 (0.887-0.907) | 0.867 (0.841-0.893) | 0.794 (0.781-0.808) | 0.494 (0.453-0.535) | 0.152 (0.148-0.156) |
| DT+RF | 72.000 | Rank 74 | 0.785 (0.773-0.797) | 0.701 (0.677-0.723) | 0.910 (0.900-0.921) | 0.900 (0.875-0.925) | 0.768 (0.755-0.781) | 0.571 (0.526-0.617) | 0.152 (0.147-0.157) |
| AdaBoost+DT+LightGBM+SVM | 72.571 | Rank 75 | 0.794 (0.781-0.806) | 0.719 (0.695-0.742) | 0.903 (0.892-0.913) | 0.921 (0.899-0.943) | 0.774 (0.761-0.788) | 0.491 (0.447-0.536) | 0.161 (0.157-0.166) |
| DT+RF+NaiveBayes+CatBoost | 73.571 | Rank 76 | 0.789 (0.777-0.801) | 0.704 (0.680-0.728) | 0.898 (0.887-0.910) | 0.900 (0.876-0.926) | 0.772 (0.758-0.786) | 0.551 (0.505-0.593) | 0.153 (0.148-0.158) |
| AdaBoost+DT+CatBoost | 74.143 | Rank 77 | 0.798 (0.786-0.810) | 0.652 (0.625-0.679) | 0.881 (0.868-0.894) | 0.799 (0.767-0.832) | 0.798 (0.785-0.810) | 0.555 (0.514-0.595) | 0.150 (0.146-0.154) |
| RF+LightGBM+SVM+CatBoost | 75.714 | Rank 78 | 0.783 (0.770-0.796) | 0.699 (0.676-0.723) | 0.905 (0.895-0.916) | 0.900 (0.876-0.924) | 0.765 (0.751-0.779) | 0.566 (0.521-0.609) | 0.150 (0.145-0.156) |
| RF+NaiveBayes+SVM | 76.714 | Rank 79 | 0.787 (0.775-0.799) | 0.712 (0.689-0.736) | 0.899 (0.889-0.910) | 0.918 (0.897-0.940) | 0.767 (0.755-0.780) | 0.541 (0.499-0.582) | 0.161 (0.157-0.166) |
| DT+RF+NaiveBayes+SVM+CatBoost | 77.429 | Rank 80 | 0.785 (0.773-0.798) | 0.681 (0.656-0.706) | 0.899 (0.888-0.911) | 0.866 (0.838-0.894) | 0.773 (0.758-0.786) | 0.568 (0.523-0.611) | 0.148 (0.143-0.153) |
| AdaBoost+DT+NaiveBayes+SVM+CatBoost | 78.857 | Rank 81 | 0.797 (0.784-0.809) | 0.659 (0.629-0.688) | 0.880 (0.868-0.893) | 0.812 (0.779-0.847) | 0.795 (0.781-0.808) | 0.525 (0.479-0.569) | 0.153 (0.149-0.158) |
| DT+RF+SVM | 79.143 | Rank 82 | 0.774 (0.761-0.787) | 0.701 (0.677-0.724) | 0.906 (0.895-0.917) | 0.918 (0.896-0.941) | 0.752 (0.737-0.767) | 0.559 (0.514-0.602) | 0.159 (0.154-0.165) |
| RF+SVM+CatBoost | 79.286 | Rank 83 | 0.780 (0.768-0.792) | 0.686 (0.661-0.710) | 0.905 (0.893-0.916) | 0.881 (0.855-0.908) | 0.765 (0.751-0.779) | 0.592 (0.546-0.637) | 0.151 (0.146-0.156) |
| LightGBM+SVM | 79.571 | Rank 84 | 0.790 (0.778-0.802) | 0.699 (0.674-0.722) | 0.903 (0.892-0.912) | 0.891 (0.865-0.918) | 0.775 (0.761-0.788) | 0.508 (0.462-0.552) | 0.156 (0.152-0.161) |
| AdaBoost+DT+RF+SVM | 79.714 | Rank 85 | 0.787 (0.775-0.799) | 0.700 (0.675-0.724) | 0.901 (0.891-0.911) | 0.897 (0.872-0.923) | 0.770 (0.757-0.784) | 0.555 (0.511-0.597) | 0.162 (0.157-0.166) |
| AdaBoost+DT+RF+SVM+CatBoost | 80.571 | Rank 86 | 0.789 (0.777-0.802) | 0.691 (0.667-0.715) | 0.893 (0.882-0.904) | 0.878 (0.852-0.906) | 0.776 (0.762-0.789) | 0.549 (0.508-0.592) | 0.158 (0.153-0.163) |
| AdaBoost+DT+RF+NaiveBayes | 81.000 | Rank 87 | 0.763 (0.750-0.776) | 0.689 (0.664-0.712) | 0.910 (0.899-0.922) | 0.911 (0.888-0.935) | 0.741 (0.726-0.755) | 0.588 (0.539-0.633) | 0.160 (0.154-0.165) |
| DT+LightGBM+NaiveBayes+CatBoost | 81.429 | Rank 88 | 0.786 (0.774-0.798) | 0.688 (0.661-0.712) | 0.899 (0.888-0.911) | 0.876 (0.848-0.904) | 0.772 (0.760-0.785) | 0.548 (0.503-0.593) | 0.154 (0.149-0.159) |
| DT+SVM+CatBoost | 81.429 | Rank 88 | 0.789 (0.777-0.801) | 0.649 (0.623-0.676) | 0.884 (0.872-0.897) | 0.806 (0.775-0.837) | 0.786 (0.773-0.799) | 0.542 (0.497-0.591) | 0.150 (0.145-0.154) |
| RF+NaiveBayes+SVM+CatBoost | 82.143 | Rank 90 | 0.767 (0.755-0.779) | 0.684 (0.660-0.708) | 0.907 (0.896-0.918) | 0.897 (0.874-0.923) | 0.747 (0.734-0.761) | 0.603 (0.561-0.644) | 0.155 (0.150-0.160) |
| LightGBM+NaiveBayes+CatBoost | 83.143 | Rank 91 | 0.782 (0.771-0.794) | 0.699 (0.673-0.723) | 0.899 (0.888-0.910) | 0.902 (0.880-0.926) | 0.764 (0.751-0.778) | 0.506 (0.463-0.552) | 0.154 (0.148-0.159) |
| AdaBoost+NaiveBayes+SVM+CatBoost | 83.857 | Rank 92 | 0.784 (0.772-0.797) | 0.667 (0.641-0.693) | 0.889 (0.875-0.901) | 0.842 (0.811-0.873) | 0.776 (0.763-0.790) | 0.565 (0.518-0.607) | 0.155 (0.151-0.159) |
| DT+RF+NaiveBayes | 86.286 | Rank 93 | 0.782 (0.770-0.794) | 0.680 (0.655-0.704) | 0.898 (0.888-0.910) | 0.869 (0.841-0.896) | 0.769 (0.756-0.782) | 0.548 (0.503-0.591) | 0.155 (0.150-0.160) |
| AdaBoost+DT+NaiveBayes+CatBoost | 89.429 | Rank 94 | 0.779 (0.767-0.791) | 0.652 (0.625-0.679) | 0.881 (0.868-0.894) | 0.824 (0.794-0.856) | 0.773 (0.758-0.785) | 0.537 (0.496-0.582) | 0.151 (0.147-0.156) |
| SVM+CatBoost | 89.571 | Rank 95 | 0.787 (0.775-0.799) | 0.639 (0.611-0.666) | 0.876 (0.864-0.888) | 0.791 (0.758-0.824) | 0.786 (0.773-0.799) | 0.501 (0.458-0.547) | 0.154 (0.149-0.159) |
| AdaBoost+NaiveBayes+CatBoost | 90.571 | Rank 96 | 0.775 (0.762-0.788) | 0.667 (0.639-0.694) | 0.888 (0.876-0.900) | 0.857 (0.828-0.887) | 0.762 (0.748-0.776) | 0.544 (0.499-0.591) | 0.154 (0.149-0.159) |
| NaiveBayes+SVM+CatBoost | 91.429 | Rank 97 | 0.765 (0.753-0.778) | 0.660 (0.634-0.686) | 0.891 (0.879-0.903) | 0.857 (0.829-0.885) | 0.751 (0.738-0.765) | 0.549 (0.505-0.594) | 0.153 (0.148-0.159) |
| AdaBoost+LightGBM+NaiveBayes+SVM | 92.000 | Rank 98 | 0.783 (0.771-0.795) | 0.685 (0.657-0.710) | 0.891 (0.880-0.901) | 0.875 (0.848-0.905) | 0.769 (0.756-0.782) | 0.471 (0.430-0.515) | 0.161 (0.157-0.166) |
| RF+NaiveBayes+CatBoost | 92.571 | Rank 99 | 0.768 (0.756-0.781) | 0.677 (0.653-0.701) | 0.887 (0.875-0.901) | 0.882 (0.859-0.909) | 0.751 (0.737-0.765) | 0.540 (0.495-0.583) | 0.157 (0.151-0.162) |
| AdaBoost+DT+LightGBM | 93.429 | Rank 100 | 0.785 (0.772-0.797) | 0.671 (0.644-0.698) | 0.882 (0.870-0.895) | 0.848 (0.819-0.880) | 0.775 (0.761-0.787) | 0.482 (0.436-0.526) | 0.166 (0.162-0.170) |
| AdaBoost+DT+LightGBM+NaiveBayes+SVM | 94.286 | Rank 101 | 0.786 (0.774-0.798) | 0.646 (0.616-0.674) | 0.873 (0.860-0.885) | 0.804 (0.771-0.837) | 0.783 (0.771-0.797) | 0.471 (0.430-0.515) | 0.161 (0.157-0.165) |
| RF+CatBoost | 94.286 | Rank 101 | 0.764 (0.752-0.777) | 0.655 (0.628-0.678) | 0.885 (0.874-0.896) | 0.849 (0.819-0.877) | 0.751 (0.738-0.765) | 0.554 (0.513-0.594) | 0.157 (0.152-0.161) |
| DT+NaiveBayes+CatBoost | 95.714 | Rank 103 | 0.778 (0.765-0.790) | 0.649 (0.619-0.678) | 0.874 (0.861-0.888) | 0.821 (0.791-0.854) | 0.771 (0.758-0.785) | 0.520 (0.476-0.564) | 0.157 (0.153-0.162) |
| NaiveBayes+CatBoost | 98.714 | Rank 104 | 0.777 (0.765-0.790) | 0.651 (0.624-0.677) | 0.871 (0.858-0.884) | 0.824 (0.793-0.856) | 0.770 (0.757-0.784) | 0.489 (0.443-0.534) | 0.160 (0.156-0.165) |
| AdaBoost+DT+SVM+CatBoost | 100.286 | Rank 105 | 0.771 (0.758-0.784) | 0.650 (0.626-0.675) | 0.877 (0.865-0.890) | 0.832 (0.803-0.862) | 0.761 (0.748-0.775) | 0.506 (0.462-0.550) | 0.161 (0.157-0.166) |
| AdaBoost+SVM+CatBoost | 102.286 | Rank 106 | 0.748 (0.735-0.761) | 0.651 (0.624-0.675) | 0.878 (0.865-0.892) | 0.864 (0.835-0.892) | 0.730 (0.716-0.744) | 0.520 (0.475-0.565) | 0.167 (0.161-0.172) |
| DT+CatBoost | 103.714 | Rank 107 | 0.769 (0.757-0.781) | 0.644 (0.616-0.669) | 0.872 (0.859-0.885) | 0.824 (0.794-0.854) | 0.760 (0.748-0.774) | 0.481 (0.437-0.526) | 0.162 (0.158-0.167) |
| AdaBoost+CatBoost | 104.000 | Rank 108 | 0.762 (0.750-0.776) | 0.646 (0.620-0.674) | 0.871 (0.858-0.885) | 0.836 (0.808-0.868) | 0.751 (0.738-0.765) | 0.507 (0.462-0.551) | 0.163 (0.158-0.168) |
| DT+NaiveBayes+SVM+CatBoost | 104.857 | Rank 109 | 0.766 (0.753-0.779) | 0.642 (0.615-0.667) | 0.865 (0.851-0.879) | 0.824 (0.794-0.856) | 0.757 (0.742-0.771) | 0.483 (0.438-0.527) | 0.163 (0.158-0.168) |
| DT+NaiveBayes | 108.571 | Rank 110 | 0.741 (0.727-0.754) | 0.365 (0.329-0.397) | 0.683 (0.660-0.707) | 0.424 (0.382-0.463) | 0.789 (0.776-0.802) | 0.232 (0.204-0.261) | 0.213 (0.210-0.216) |
| DT+NaiveBayes+SVM | 112.571 | Rank 111 | 0.711 (0.698-0.724) | 0.448 (0.415-0.479) | 0.721 (0.700-0.742) | 0.566 (0.524-0.607) | 0.733 (0.719-0.747) | 0.274 (0.242-0.306) | 0.208 (0.204-0.213) |
| AdaBoost+NaiveBayes+SVM | 114.286 | Rank 112 | 0.695 (0.681-0.709) | 0.447 (0.415-0.478) | 0.709 (0.687-0.731) | 0.578 (0.537-0.620) | 0.712 (0.697-0.727) | 0.276 (0.246-0.311) | 0.210 (0.206-0.215) |
| AdaBoost+NaiveBayes | 114.714 | Rank 113 | 0.717 (0.705-0.730) | 0.409 (0.377-0.440) | 0.687 (0.665-0.709) | 0.502 (0.460-0.544) | 0.750 (0.736-0.763) | 0.256 (0.225-0.288) | 0.196 (0.192-0.199) |
| DT+SVM | 115.000 | Rank 114 | 0.696 (0.682-0.710) | 0.435 (0.401-0.469) | 0.717 (0.696-0.741) | 0.560 (0.515-0.600) | 0.716 (0.701-0.732) | 0.264 (0.234-0.294) | 0.223 (0.220-0.226) |
| AdaBoost+DT+NaiveBayes+SVM | 115.143 | Rank 115 | 0.697 (0.683-0.711) | 0.431 (0.396-0.463) | 0.709 (0.686-0.731) | 0.550 (0.508-0.594) | 0.720 (0.705-0.735) | 0.259 (0.225-0.291) | 0.210 (0.205-0.215) |
| AdaBoost+DT+SVM | 115.286 | Rank 116 | 0.676 (0.663-0.690) | 0.464 (0.433-0.494) | 0.713 (0.691-0.735) | 0.620 (0.580-0.660) | 0.685 (0.670-0.699) | 0.255 (0.225-0.287) | 0.213 (0.209-0.217) |
| NaiveBayes+SVM | 116.286 | Rank 117 | 0.669 (0.655-0.683) | 0.453 (0.423-0.482) | 0.697 (0.676-0.721) | 0.608 (0.567-0.647) | 0.678 (0.663-0.693) | 0.284 (0.251-0.320) | 0.223 (0.219-0.227) |
| AdaBoost+DT+NaiveBayes | 116.714 | Rank 118 | 0.688 (0.674-0.701) | 0.421 (0.388-0.453) | 0.690 (0.667-0.713) | 0.543 (0.502-0.585) | 0.710 (0.695-0.724) | 0.254 (0.225-0.283) | 0.206 (0.202-0.210) |
| AdaBoost+SVM | 117.429 | Rank 119 | 0.647 (0.631-0.661) | 0.466 (0.434-0.496) | 0.704 (0.682-0.727) | 0.649 (0.611-0.689) | 0.647 (0.631-0.661) | 0.241 (0.213-0.268) | 0.218 (0.213-0.223) |
| AdaBoost+DT | 119.429 | Rank 120 | 0.643 (0.629-0.658) | 0.451 (0.421-0.483) | 0.681 (0.658-0.705) | 0.627 (0.587-0.665) | 0.646 (0.630-0.661) | 0.241 (0.212-0.270) | 0.228 (0.224-0.232) |
| RF | 120.571 | Rank 121 | 0.665 (0.647-0.684) | 0.346 (0.289-0.403) | 0.622 (0.582-0.661) | 0.448 (0.380-0.515) | 0.698 (0.676-0.720) | 0.213 (0.159-0.267) | 0.206 (0.199-0.214) |
| NaiveBayes | 122.000 | Rank 122 | 0.690 (0.546-0.834) | 0.296 (0.234-0.359) | 0.650 (0.624-0.677) | 0.396 (0.212-0.580) | 0.739 (0.554-0.925) | 0.221 (0.191-0.250) | 0.251 (0.131-0.372) |
| CatBoost | 122.571 | Rank 123 | 0.653 (0.632-0.675) | 0.342 (0.290-0.393) | 0.601 (0.569-0.633) | 0.448 (0.396-0.501) | 0.684 (0.660-0.708) | 0.198 (0.148-0.248) | 0.208 (0.199-0.218) |
| AdaBoost | 122.857 | Rank 124 | 0.594 (0.554-0.633) | 0.385 (0.324-0.446) | 0.626 (0.589-0.663) | 0.555 (0.489-0.622) | 0.598 (0.550-0.646) | 0.214 (0.176-0.252) | 0.231 (0.222-0.239) |
| SVM | 123.714 | Rank 125 | 0.635 (0.600-0.671) | 0.376 (0.333-0.418) | 0.619 (0.587-0.651) | 0.522 (0.441-0.603) | 0.655 (0.609-0.701) | 0.203 (0.165-0.240) | 0.232 (0.225-0.238) |
| LightGBM | 124.714 | Rank 126 | 0.659 (0.644-0.673) | 0.321 (0.266-0.375) | 0.578 (0.536-0.620) | 0.415 (0.347-0.484) | 0.696 (0.687-0.705) | 0.188 (0.146-0.229) | 0.228 (0.218-0.237) |
| DT | 124.857 | Rank 127 | 0.585 (0.543-0.627) | 0.379 (0.335-0.422) | 0.609 (0.574-0.643) | 0.555 (0.490-0.621) | 0.589 (0.532-0.646) | 0.183 (0.151-0.215) | 0.239 (0.233-0.246) |
| XGBoost | 124.857 | Rank 127 | 0.659 (0.629-0.689) | 0.330 (0.281-0.378) | 0.586 (0.543-0.630) | 0.433 (0.349-0.517) | 0.696 (0.672-0.720) | 0.171 (0.151-0.191) | 0.276 (0.253-0.299) |
| KNN | 128.143 | Rank 129 | 0.584 (0.557-0.610) | 0.305 (0.263-0.347) | 0.542 (0.494-0.591) | 0.428 (0.392-0.465) | 0.607 (0.579-0.634) | 0.162 (0.119-0.206) | 0.287 (0.268-0.306) |

**Supplementary Table 21.** Ranking of 129 machine learning models in the CHARLS validation set.

| **Model** | **Normalized Rank Score** | **Comprehensive Rank** | **Accuracy (95% CI)** | **F_Beta (95% CI)** | **ROC_AUC (95% CI)** | **Sensitivity (95% CI)** | **Specificity (95% CI)** | **PR_AUC (95% CI)** | **Brier (95% CI)** |
| --- | --- | --- | --- | --- | --- | --- | --- | --- | --- |
| RF+LightGBM+NaiveBayes+CatBoost | 6.429 | Rank 1 | 0.728 (0.709-0.748) | 0.407 (0.353-0.458) | 0.667 (0.626-0.709) | 0.529 (0.459-0.600) | 0.753 (0.733-0.774) | 0.246 (0.190-0.305) | 0.183 (0.174-0.191) |
| DT+RF+LightGBM | 18.286 | Rank 2 | 0.716 (0.696-0.735) | 0.379 (0.328-0.428) | 0.663 (0.624-0.704) | 0.496 (0.431-0.561) | 0.744 (0.723-0.764) | 0.224 (0.176-0.270) | 0.190 (0.182-0.198) |
| DT+RF+NaiveBayes+SVM+CatBoost | 22.857 | Rank 3 | 0.706 (0.686-0.726) | 0.379 (0.331-0.428) | 0.658 (0.616-0.699) | 0.505 (0.439-0.575) | 0.731 (0.710-0.752) | 0.219 (0.175-0.265) | 0.186 (0.178-0.195) |
| RF+LightGBM+NaiveBayes+SVM | 29.429 | Rank 4 | 0.705 (0.684-0.726) | 0.374 (0.322-0.422) | 0.650 (0.606-0.690) | 0.497 (0.429-0.566) | 0.731 (0.710-0.752) | 0.251 (0.193-0.301) | 0.192 (0.184-0.201) |
| RF+SVM+CatBoost | 29.857 | Rank 5 | 0.707 (0.687-0.728) | 0.374 (0.321-0.425) | 0.661 (0.620-0.703) | 0.496 (0.429-0.567) | 0.733 (0.713-0.754) | 0.211 (0.164-0.261) | 0.192 (0.183-0.200) |
| AdaBoost+RF+NaiveBayes+CatBoost | 32.000 | Rank 6 | 0.708 (0.688-0.730) | 0.369 (0.313-0.423) | 0.644 (0.602-0.683) | 0.486 (0.417-0.558) | 0.736 (0.715-0.758) | 0.236 (0.183-0.289) | 0.190 (0.180-0.198) |
| AdaBoost+DT+RF+SVM+CatBoost | 32.429 | Rank 7 | 0.700 (0.679-0.722) | 0.398 (0.345-0.447) | 0.661 (0.618-0.705) | 0.538 (0.471-0.605) | 0.721 (0.699-0.742) | 0.238 (0.184-0.293) | 0.198 (0.189-0.207) |
| CatBoost | 32.857 | Rank 8 | 0.705 (0.683-0.726) | 0.395 (0.341-0.444) | 0.651 (0.610-0.693) | 0.529 (0.457-0.600) | 0.727 (0.704-0.750) | 0.224 (0.172-0.279) | 0.196 (0.188-0.204) |
| AdaBoost+LightGBM+SVM+CatBoost | 36.143 | Rank 9 | 0.719 (0.699-0.739) | 0.358 (0.303-0.410) | 0.644 (0.602-0.688) | 0.461 (0.391-0.532) | 0.752 (0.732-0.772) | 0.213 (0.164-0.264) | 0.182 (0.173-0.190) |
| AdaBoost+DT+RF+LightGBM+NaiveBayes | 36.429 | Rank 10 | 0.708 (0.687-0.729) | 0.359 (0.304-0.410) | 0.656 (0.616-0.697) | 0.472 (0.400-0.543) | 0.738 (0.717-0.761) | 0.222 (0.171-0.270) | 0.193 (0.185-0.202) |
| AdaBoost+DT+RF+LightGBM | 36.571 | Rank 11 | 0.714 (0.693-0.735) | 0.367 (0.311-0.418) | 0.647 (0.603-0.690) | 0.479 (0.411-0.551) | 0.743 (0.721-0.765) | 0.204 (0.154-0.248) | 0.189 (0.181-0.198) |
| DT+SVM+CatBoost | 37.286 | Rank 12 | 0.717 (0.695-0.736) | 0.363 (0.303-0.417) | 0.645 (0.603-0.686) | 0.471 (0.397-0.543) | 0.748 (0.726-0.767) | 0.203 (0.157-0.249) | 0.185 (0.178-0.193) |
| AdaBoost+NaiveBayes | 39.000 | Rank 13 | 0.731 (0.710-0.751) | 0.365 (0.309-0.417) | 0.661 (0.620-0.699) | 0.463 (0.392-0.531) | 0.765 (0.744-0.786) | 0.196 (0.156-0.244) | 0.195 (0.190-0.201) |
| DT+RF+SVM+CatBoost | 41.714 | Rank 14 | 0.700 (0.680-0.722) | 0.364 (0.310-0.412) | 0.652 (0.613-0.693) | 0.486 (0.418-0.554) | 0.727 (0.707-0.750) | 0.204 (0.161-0.253) | 0.190 (0.181-0.198) |
| RF+LightGBM+NaiveBayes | 42.143 | Rank 15 | 0.705 (0.682-0.726) | 0.359 (0.307-0.406) | 0.653 (0.617-0.693) | 0.472 (0.408-0.539) | 0.734 (0.712-0.755) | 0.216 (0.164-0.266) | 0.196 (0.187-0.205) |
| AdaBoost+RF+LightGBM+NaiveBayes+SVM+CatBoost | 43.000 | Rank 16 | 0.700 (0.678-0.720) | 0.358 (0.304-0.407) | 0.655 (0.617-0.697) | 0.477 (0.408-0.549) | 0.727 (0.704-0.749) | 0.211 (0.161-0.260) | 0.192 (0.182-0.201) |
| RF+LightGBM+SVM | 43.143 | Rank 17 | 0.700 (0.679-0.721) | 0.353 (0.294-0.402) | 0.656 (0.616-0.696) | 0.469 (0.397-0.536) | 0.729 (0.708-0.751) | 0.226 (0.173-0.275) | 0.193 (0.184-0.201) |
| DT+RF+LightGBM+NaiveBayes+CatBoost | 44.000 | Rank 18 | 0.695 (0.675-0.717) | 0.363 (0.311-0.416) | 0.649 (0.608-0.692) | 0.487 (0.416-0.558) | 0.721 (0.700-0.743) | 0.227 (0.175-0.279) | 0.193 (0.184-0.201) |
| LightGBM+NaiveBayes+SVM+CatBoost | 44.286 | Rank 19 | 0.703 (0.681-0.724) | 0.398 (0.343-0.448) | 0.649 (0.608-0.691) | 0.537 (0.467-0.606) | 0.724 (0.702-0.748) | 0.197 (0.155-0.240) | 0.196 (0.187-0.206) |
| RF+SVM | 44.714 | Rank 20 | 0.691 (0.672-0.711) | 0.409 (0.357-0.457) | 0.665 (0.626-0.705) | 0.564 (0.493-0.635) | 0.708 (0.686-0.730) | 0.212 (0.166-0.262) | 0.203 (0.195-0.210) |
| AdaBoost+DT+LightGBM+CatBoost | 45.000 | Rank 21 | 0.723 (0.703-0.742) | 0.355 (0.298-0.407) | 0.638 (0.594-0.680) | 0.454 (0.380-0.527) | 0.756 (0.736-0.776) | 0.205 (0.159-0.253) | 0.186 (0.177-0.194) |
| DT+NaiveBayes+SVM+CatBoost | 45.857 | Rank 22 | 0.691 (0.669-0.712) | 0.393 (0.341-0.441) | 0.669 (0.631-0.708) | 0.539 (0.470-0.603) | 0.710 (0.687-0.732) | 0.204 (0.162-0.248) | 0.197 (0.188-0.205) |
| DT+NaiveBayes | 46.143 | Rank 23 | 0.747 (0.726-0.766) | 0.354 (0.302-0.404) | 0.678 (0.638-0.717) | 0.435 (0.369-0.500) | 0.786 (0.766-0.805) | 0.210 (0.164-0.255) | 0.213 (0.208-0.218) |
| DT+RF+NaiveBayes | 46.571 | Rank 24 | 0.692 (0.672-0.714) | 0.363 (0.311-0.413) | 0.657 (0.619-0.697) | 0.489 (0.422-0.559) | 0.718 (0.696-0.741) | 0.230 (0.176-0.287) | 0.198 (0.190-0.206) |
| DT+LightGBM+NaiveBayes | 46.714 | Rank 25 | 0.721 (0.701-0.742) | 0.337 (0.283-0.388) | 0.651 (0.613-0.693) | 0.427 (0.361-0.494) | 0.758 (0.738-0.780) | 0.205 (0.158-0.255) | 0.187 (0.179-0.194) |
| AdaBoost+DT+RF+SVM | 47.286 | Rank 26 | 0.693 (0.672-0.714) | 0.366 (0.311-0.415) | 0.657 (0.616-0.697) | 0.493 (0.426-0.558) | 0.718 (0.696-0.740) | 0.224 (0.166-0.274) | 0.201 (0.194-0.208) |
| AdaBoost+DT+CatBoost | 47.429 | Rank 27 | 0.711 (0.691-0.733) | 0.349 (0.292-0.401) | 0.652 (0.613-0.694) | 0.454 (0.384-0.522) | 0.744 (0.723-0.766) | 0.200 (0.157-0.246) | 0.187 (0.179-0.195) |
| AdaBoost+DT+LightGBM+NaiveBayes+SVM+CatBoost | 47.857 | Rank 28 | 0.690 (0.668-0.713) | 0.371 (0.317-0.422) | 0.651 (0.609-0.693) | 0.504 (0.434-0.574) | 0.714 (0.690-0.737) | 0.218 (0.162-0.264) | 0.196 (0.187-0.205) |
| AdaBoost+CatBoost | 48.286 | Rank 29 | 0.687 (0.667-0.707) | 0.395 (0.342-0.441) | 0.656 (0.613-0.697) | 0.546 (0.473-0.612) | 0.705 (0.684-0.727) | 0.211 (0.164-0.261) | 0.198 (0.189-0.206) |
| AdaBoost+DT+LightGBM+NaiveBayes+SVM | 49.714 | Rank 30 | 0.704 (0.685-0.726) | 0.358 (0.308-0.407) | 0.636 (0.597-0.679) | 0.472 (0.406-0.535) | 0.734 (0.714-0.755) | 0.220 (0.167-0.268) | 0.193 (0.185-0.200) |
| DT+LightGBM+SVM+CatBoost | 49.714 | Rank 30 | 0.694 (0.672-0.715) | 0.357 (0.302-0.407) | 0.658 (0.614-0.701) | 0.479 (0.405-0.547) | 0.721 (0.699-0.744) | 0.200 (0.156-0.245) | 0.191 (0.182-0.200) |
| DT+RF+LightGBM+NaiveBayes+SVM+CatBoost | 49.714 | Rank 30 | 0.720 (0.698-0.741) | 0.330 (0.278-0.378) | 0.647 (0.605-0.689) | 0.419 (0.353-0.485) | 0.757 (0.736-0.780) | 0.207 (0.157-0.260) | 0.181 (0.173-0.190) |
| RF+NaiveBayes | 49.857 | Rank 33 | 0.692 (0.671-0.714) | 0.357 (0.305-0.406) | 0.656 (0.618-0.694) | 0.479 (0.414-0.547) | 0.719 (0.698-0.741) | 0.225 (0.171-0.279) | 0.196 (0.188-0.205) |
| AdaBoost+RF+LightGBM+CatBoost | 50.429 | Rank 34 | 0.696 (0.675-0.716) | 0.353 (0.300-0.405) | 0.646 (0.609-0.687) | 0.472 (0.406-0.541) | 0.724 (0.701-0.744) | 0.219 (0.166-0.269) | 0.192 (0.183-0.202) |
| DT+NaiveBayes+CatBoost | 50.571 | Rank 35 | 0.701 (0.680-0.722) | 0.350 (0.296-0.400) | 0.654 (0.614-0.692) | 0.463 (0.395-0.532) | 0.731 (0.709-0.753) | 0.208 (0.164-0.254) | 0.193 (0.185-0.202) |
| AdaBoost+RF+LightGBM+NaiveBayes+SVM | 50.714 | Rank 36 | 0.712 (0.691-0.732) | 0.360 (0.308-0.414) | 0.638 (0.599-0.682) | 0.470 (0.397-0.544) | 0.742 (0.722-0.763) | 0.199 (0.154-0.247) | 0.192 (0.183-0.201) |
| AdaBoost+LightGBM+NaiveBayes+SVM+CatBoost | 51.286 | Rank 37 | 0.723 (0.702-0.742) | 0.326 (0.272-0.379) | 0.645 (0.605-0.687) | 0.412 (0.343-0.481) | 0.762 (0.740-0.783) | 0.207 (0.160-0.252) | 0.185 (0.176-0.194) |
| AdaBoost+RF | 52.000 | Rank 38 | 0.704 (0.682-0.725) | 0.366 (0.311-0.414) | 0.652 (0.615-0.695) | 0.486 (0.416-0.554) | 0.731 (0.709-0.753) | 0.199 (0.154-0.250) | 0.202 (0.193-0.211) |
| DT+RF | 52.000 | Rank 38 | 0.688 (0.667-0.710) | 0.376 (0.323-0.428) | 0.655 (0.615-0.693) | 0.512 (0.443-0.582) | 0.710 (0.689-0.732) | 0.209 (0.163-0.258) | 0.199 (0.190-0.207) |
| SVM+CatBoost | 52.429 | Rank 40 | 0.700 (0.679-0.721) | 0.338 (0.288-0.385) | 0.651 (0.607-0.693) | 0.445 (0.378-0.514) | 0.732 (0.710-0.753) | 0.212 (0.166-0.258) | 0.189 (0.180-0.197) |
| DT+RF+LightGBM+SVM | 52.857 | Rank 41 | 0.705 (0.684-0.726) | 0.363 (0.312-0.411) | 0.632 (0.592-0.671) | 0.479 (0.412-0.544) | 0.734 (0.712-0.756) | 0.202 (0.153-0.250) | 0.192 (0.183-0.202) |
| AdaBoost+RF+SVM+CatBoost | 54.286 | Rank 42 | 0.710 (0.690-0.731) | 0.349 (0.290-0.397) | 0.638 (0.597-0.677) | 0.455 (0.381-0.522) | 0.742 (0.721-0.763) | 0.201 (0.153-0.244) | 0.186 (0.178-0.194) |
| AdaBoost+SVM+CatBoost | 54.429 | Rank 43 | 0.669 (0.647-0.691) | 0.382 (0.333-0.431) | 0.669 (0.632-0.708) | 0.538 (0.471-0.605) | 0.685 (0.664-0.710) | 0.215 (0.172-0.263) | 0.201 (0.193-0.210) |
| RF+NaiveBayes+SVM | 54.429 | Rank 43 | 0.687 (0.666-0.708) | 0.375 (0.317-0.425) | 0.648 (0.605-0.689) | 0.512 (0.442-0.581) | 0.709 (0.688-0.729) | 0.231 (0.176-0.278) | 0.205 (0.197-0.213) |
| AdaBoost+LightGBM+NaiveBayes+SVM | 54.714 | Rank 45 | 0.685 (0.664-0.706) | 0.396 (0.346-0.447) | 0.662 (0.623-0.704) | 0.549 (0.481-0.618) | 0.702 (0.680-0.725) | 0.199 (0.159-0.245) | 0.199 (0.192-0.208) |
| AdaBoost+DT+RF | 55.286 | Rank 46 | 0.709 (0.688-0.731) | 0.349 (0.293-0.401) | 0.642 (0.602-0.685) | 0.455 (0.382-0.526) | 0.741 (0.720-0.762) | 0.205 (0.162-0.251) | 0.193 (0.185-0.200) |
| DT+RF+SVM | 56.000 | Rank 47 | 0.672 (0.651-0.693) | 0.371 (0.315-0.421) | 0.657 (0.615-0.697) | 0.517 (0.443-0.589) | 0.692 (0.669-0.713) | 0.241 (0.187-0.295) | 0.203 (0.195-0.212) |
| DT+RF+CatBoost | 56.286 | Rank 48 | 0.695 (0.673-0.716) | 0.347 (0.294-0.398) | 0.648 (0.608-0.689) | 0.463 (0.392-0.535) | 0.724 (0.702-0.745) | 0.215 (0.165-0.261) | 0.193 (0.184-0.201) |
| DT+RF+NaiveBayes+SVM | 56.286 | Rank 48 | 0.688 (0.667-0.708) | 0.364 (0.312-0.413) | 0.653 (0.616-0.692) | 0.494 (0.427-0.564) | 0.712 (0.691-0.733) | 0.215 (0.164-0.264) | 0.202 (0.194-0.210) |
| LightGBM+NaiveBayes | 57.714 | Rank 50 | 0.739 (0.719-0.760) | 0.321 (0.266-0.372) | 0.646 (0.608-0.685) | 0.394 (0.328-0.459) | 0.782 (0.763-0.803) | 0.194 (0.149-0.234) | 0.180 (0.174-0.187) |
| AdaBoost+RF+LightGBM+SVM+CatBoost | 58.714 | Rank 51 | 0.715 (0.696-0.736) | 0.334 (0.278-0.385) | 0.646 (0.606-0.684) | 0.429 (0.355-0.500) | 0.751 (0.732-0.772) | 0.185 (0.148-0.225) | 0.185 (0.177-0.193) |
| AdaBoost+RF+SVM | 58.857 | Rank 52 | 0.706 (0.686-0.726) | 0.330 (0.273-0.380) | 0.635 (0.596-0.675) | 0.429 (0.358-0.493) | 0.741 (0.720-0.762) | 0.222 (0.166-0.273) | 0.190 (0.182-0.199) |
| RF+LightGBM+NaiveBayes+SVM+CatBoost | 59.429 | Rank 53 | 0.711 (0.690-0.732) | 0.349 (0.292-0.400) | 0.644 (0.600-0.684) | 0.454 (0.382-0.520) | 0.744 (0.723-0.765) | 0.193 (0.151-0.239) | 0.193 (0.184-0.202) |
| RF+LightGBM+CatBoost | 59.571 | Rank 54 | 0.720 (0.699-0.741) | 0.335 (0.282-0.389) | 0.634 (0.596-0.675) | 0.428 (0.359-0.494) | 0.756 (0.737-0.777) | 0.198 (0.156-0.246) | 0.186 (0.178-0.194) |
| DT+LightGBM+CatBoost | 60.143 | Rank 55 | 0.696 (0.676-0.717) | 0.347 (0.296-0.396) | 0.646 (0.604-0.685) | 0.462 (0.394-0.526) | 0.726 (0.704-0.746) | 0.202 (0.162-0.247) | 0.191 (0.183-0.200) |
| AdaBoost+RF+CatBoost | 60.286 | Rank 56 | 0.693 (0.672-0.713) | 0.351 (0.303-0.397) | 0.638 (0.596-0.678) | 0.471 (0.408-0.536) | 0.720 (0.699-0.741) | 0.213 (0.163-0.258) | 0.192 (0.183-0.201) |
| LightGBM+SVM | 60.286 | Rank 56 | 0.690 (0.669-0.711) | 0.350 (0.298-0.401) | 0.650 (0.608-0.694) | 0.471 (0.403-0.540) | 0.717 (0.696-0.738) | 0.227 (0.174-0.275) | 0.198 (0.190-0.205) |
| AdaBoost+DT+RF+NaiveBayes | 61.571 | Rank 58 | 0.678 (0.656-0.699) | 0.376 (0.322-0.424) | 0.643 (0.601-0.682) | 0.522 (0.451-0.590) | 0.698 (0.675-0.719) | 0.228 (0.172-0.277) | 0.205 (0.196-0.213) |
| DT+RF+LightGBM+NaiveBayes | 61.714 | Rank 59 | 0.695 (0.673-0.716) | 0.336 (0.285-0.386) | 0.644 (0.604-0.685) | 0.445 (0.378-0.514) | 0.726 (0.704-0.749) | 0.227 (0.173-0.280) | 0.194 (0.185-0.203) |
| AdaBoost+DT+NaiveBayes+SVM+CatBoost | 61.857 | Rank 60 | 0.718 (0.697-0.738) | 0.347 (0.291-0.397) | 0.639 (0.598-0.679) | 0.446 (0.375-0.513) | 0.752 (0.733-0.772) | 0.176 (0.139-0.215) | 0.191 (0.184-0.199) |
| AdaBoost+DT+LightGBM+SVM+CatBoost | 62.429 | Rank 61 | 0.718 (0.697-0.739) | 0.336 (0.282-0.383) | 0.633 (0.593-0.672) | 0.428 (0.359-0.493) | 0.755 (0.734-0.775) | 0.190 (0.150-0.234) | 0.185 (0.177-0.194) |
| AdaBoost+RF+LightGBM+NaiveBayes+CatBoost | 62.429 | Rank 61 | 0.719 (0.699-0.739) | 0.314 (0.258-0.367) | 0.630 (0.589-0.671) | 0.396 (0.329-0.467) | 0.760 (0.740-0.781) | 0.207 (0.154-0.260) | 0.187 (0.178-0.195) |
| DT+LightGBM+NaiveBayes+SVM | 62.571 | Rank 63 | 0.708 (0.688-0.728) | 0.331 (0.282-0.384) | 0.647 (0.608-0.687) | 0.429 (0.364-0.500) | 0.743 (0.723-0.764) | 0.203 (0.160-0.251) | 0.195 (0.187-0.202) |
| AdaBoost+DT+NaiveBayes | 62.714 | Rank 64 | 0.677 (0.656-0.699) | 0.376 (0.322-0.427) | 0.666 (0.626-0.707) | 0.523 (0.453-0.597) | 0.696 (0.675-0.719) | 0.201 (0.158-0.247) | 0.210 (0.204-0.216) |
| AdaBoost+RF+NaiveBayes+SVM | 63.143 | Rank 65 | 0.683 (0.662-0.704) | 0.352 (0.301-0.403) | 0.649 (0.609-0.692) | 0.479 (0.411-0.548) | 0.709 (0.687-0.731) | 0.217 (0.167-0.264) | 0.197 (0.188-0.206) |
| AdaBoost+DT+RF+LightGBM+CatBoost | 63.286 | Rank 66 | 0.700 (0.677-0.718) | 0.354 (0.299-0.405) | 0.634 (0.592-0.676) | 0.470 (0.397-0.544) | 0.728 (0.706-0.749) | 0.195 (0.152-0.242) | 0.192 (0.184-0.200) |
| AdaBoost+DT+RF+NaiveBayes+CatBoost | 64.143 | Rank 67 | 0.696 (0.676-0.716) | 0.343 (0.292-0.393) | 0.644 (0.606-0.685) | 0.456 (0.392-0.529) | 0.726 (0.705-0.748) | 0.212 (0.165-0.259) | 0.197 (0.188-0.205) |
| AdaBoost+RF+NaiveBayes | 64.143 | Rank 67 | 0.680 (0.658-0.701) | 0.361 (0.312-0.410) | 0.645 (0.605-0.685) | 0.495 (0.429-0.562) | 0.704 (0.681-0.725) | 0.220 (0.167-0.268) | 0.201 (0.193-0.210) |
| RF+NaiveBayes+CatBoost | 64.429 | Rank 69 | 0.693 (0.674-0.713) | 0.356 (0.308-0.405) | 0.640 (0.601-0.683) | 0.478 (0.414-0.550) | 0.720 (0.699-0.742) | 0.208 (0.163-0.258) | 0.198 (0.189-0.206) |
| AdaBoost+DT+SVM+CatBoost | 65.571 | Rank 70 | 0.694 (0.672-0.715) | 0.352 (0.304-0.402) | 0.639 (0.599-0.684) | 0.471 (0.410-0.539) | 0.722 (0.699-0.743) | 0.211 (0.165-0.260) | 0.198 (0.190-0.206) |
| AdaBoost+RF+NaiveBayes+SVM+CatBoost | 65.571 | Rank 70 | 0.689 (0.667-0.710) | 0.327 (0.275-0.381) | 0.650 (0.613-0.689) | 0.435 (0.369-0.507) | 0.721 (0.699-0.742) | 0.216 (0.162-0.263) | 0.191 (0.182-0.199) |
| NaiveBayes | 65.857 | Rank 72 | 0.785 (0.766-0.804) | 0.293 (0.235-0.347) | 0.637 (0.595-0.681) | 0.327 (0.260-0.394) | 0.842 (0.825-0.860) | 0.186 (0.145-0.228) | 0.173 (0.158-0.189) |
| AdaBoost+DT+RF+LightGBM+NaiveBayes+SVM | 66.143 | Rank 73 | 0.700 (0.680-0.721) | 0.332 (0.285-0.379) | 0.637 (0.600-0.677) | 0.436 (0.375-0.500) | 0.733 (0.711-0.755) | 0.208 (0.158-0.251) | 0.192 (0.183-0.201) |
| AdaBoost+NaiveBayes+CatBoost | 67.571 | Rank 74 | 0.689 (0.669-0.708) | 0.376 (0.321-0.428) | 0.638 (0.597-0.683) | 0.513 (0.440-0.583) | 0.711 (0.689-0.732) | 0.185 (0.146-0.225) | 0.197 (0.189-0.205) |
| LightGBM+CatBoost | 68.857 | Rank 75 | 0.705 (0.685-0.725) | 0.324 (0.267-0.375) | 0.639 (0.602-0.676) | 0.420 (0.347-0.492) | 0.741 (0.719-0.762) | 0.197 (0.149-0.243) | 0.188 (0.181-0.196) |
| DT+RF+NaiveBayes+CatBoost | 69.143 | Rank 76 | 0.681 (0.661-0.704) | 0.350 (0.293-0.399) | 0.647 (0.604-0.688) | 0.478 (0.403-0.547) | 0.707 (0.686-0.729) | 0.221 (0.165-0.271) | 0.203 (0.193-0.213) |
| AdaBoost+DT+NaiveBayes+CatBoost | 69.857 | Rank 77 | 0.692 (0.671-0.713) | 0.340 (0.289-0.390) | 0.633 (0.590-0.675) | 0.455 (0.390-0.526) | 0.721 (0.699-0.744) | 0.205 (0.160-0.255) | 0.189 (0.181-0.197) |
| AdaBoost+NaiveBayes+SVM | 70.429 | Rank 78 | 0.666 (0.644-0.687) | 0.375 (0.326-0.423) | 0.657 (0.621-0.694) | 0.529 (0.464-0.592) | 0.683 (0.660-0.705) | 0.195 (0.157-0.236) | 0.218 (0.212-0.225) |
| AdaBoost+LightGBM+CatBoost | 70.714 | Rank 79 | 0.692 (0.671-0.713) | 0.346 (0.298-0.395) | 0.643 (0.604-0.679) | 0.462 (0.400-0.532) | 0.721 (0.699-0.742) | 0.202 (0.156-0.245) | 0.196 (0.188-0.205) |
| RF | 70.714 | Rank 79 | 0.690 (0.670-0.711) | 0.350 (0.295-0.399) | 0.639 (0.597-0.680) | 0.471 (0.400-0.538) | 0.718 (0.697-0.740) | 0.207 (0.156-0.258) | 0.198 (0.192-0.205) |
| AdaBoost+DT+RF+LightGBM+NaiveBayes+SVM+CatBoost | 71.000 | Rank 81 | 0.697 (0.676-0.718) | 0.325 (0.270-0.377) | 0.633 (0.588-0.674) | 0.429 (0.356-0.500) | 0.730 (0.710-0.752) | 0.215 (0.161-0.270) | 0.193 (0.184-0.201) |
| AdaBoost+NaiveBayes+SVM+CatBoost | 71.429 | Rank 82 | 0.691 (0.671-0.712) | 0.339 (0.290-0.393) | 0.639 (0.599-0.682) | 0.454 (0.382-0.525) | 0.721 (0.699-0.742) | 0.208 (0.161-0.256) | 0.193 (0.186-0.201) |
| AdaBoost+DT+RF+CatBoost | 71.714 | Rank 83 | 0.714 (0.690-0.735) | 0.334 (0.278-0.383) | 0.624 (0.581-0.666) | 0.430 (0.355-0.494) | 0.749 (0.725-0.770) | 0.181 (0.141-0.222) | 0.190 (0.181-0.200) |
| LightGBM+SVM+CatBoost | 71.714 | Rank 83 | 0.689 (0.668-0.709) | 0.350 (0.297-0.398) | 0.651 (0.612-0.690) | 0.470 (0.400-0.537) | 0.716 (0.695-0.739) | 0.192 (0.154-0.237) | 0.196 (0.188-0.205) |
| AdaBoost+DT+LightGBM+SVM | 71.857 | Rank 85 | 0.683 (0.661-0.706) | 0.373 (0.316-0.428) | 0.643 (0.605-0.687) | 0.513 (0.441-0.592) | 0.704 (0.682-0.727) | 0.194 (0.148-0.242) | 0.204 (0.196-0.212) |
| DT+SVM | 71.857 | Rank 85 | 0.672 (0.651-0.694) | 0.368 (0.317-0.417) | 0.653 (0.616-0.691) | 0.514 (0.444-0.583) | 0.691 (0.670-0.714) | 0.201 (0.156-0.242) | 0.229 (0.224-0.234) |
| NaiveBayes+SVM+CatBoost | 71.857 | Rank 85 | 0.672 (0.651-0.693) | 0.367 (0.318-0.414) | 0.645 (0.604-0.686) | 0.512 (0.446-0.580) | 0.692 (0.671-0.715) | 0.201 (0.159-0.244) | 0.198 (0.189-0.207) |
| AdaBoost+DT+RF+NaiveBayes+SVM+CatBoost | 72.000 | Rank 88 | 0.679 (0.658-0.701) | 0.354 (0.300-0.405) | 0.643 (0.607-0.684) | 0.485 (0.416-0.551) | 0.704 (0.682-0.726) | 0.196 (0.151-0.247) | 0.192 (0.184-0.200) |
| DT+LightGBM+NaiveBayes+SVM+CatBoost | 72.429 | Rank 89 | 0.714 (0.695-0.736) | 0.334 (0.283-0.382) | 0.620 (0.582-0.659) | 0.428 (0.360-0.494) | 0.751 (0.730-0.771) | 0.178 (0.139-0.220) | 0.189 (0.180-0.197) |
| AdaBoost+LightGBM+NaiveBayes+CatBoost | 73.000 | Rank 90 | 0.715 (0.695-0.736) | 0.316 (0.262-0.369) | 0.624 (0.584-0.666) | 0.403 (0.333-0.472) | 0.754 (0.734-0.776) | 0.189 (0.147-0.234) | 0.187 (0.178-0.196) |
| LightGBM+NaiveBayes+CatBoost | 74.571 | Rank 91 | 0.684 (0.662-0.706) | 0.341 (0.285-0.392) | 0.648 (0.608-0.687) | 0.461 (0.391-0.532) | 0.711 (0.690-0.734) | 0.209 (0.162-0.259) | 0.199 (0.189-0.208) |
| DT+LightGBM+SVM | 75.143 | Rank 92 | 0.708 (0.687-0.729) | 0.318 (0.262-0.370) | 0.627 (0.590-0.667) | 0.410 (0.342-0.479) | 0.745 (0.725-0.766) | 0.190 (0.147-0.233) | 0.189 (0.181-0.197) |
| AdaBoost+DT+NaiveBayes+SVM | 75.429 | Rank 93 | 0.677 (0.655-0.697) | 0.360 (0.309-0.408) | 0.655 (0.619-0.694) | 0.496 (0.429-0.568) | 0.700 (0.675-0.722) | 0.191 (0.151-0.234) | 0.218 (0.211-0.225) |
| AdaBoost+RF+LightGBM+SVM | 76.000 | Rank 94 | 0.693 (0.673-0.714) | 0.342 (0.290-0.390) | 0.627 (0.584-0.668) | 0.455 (0.385-0.524) | 0.723 (0.703-0.746) | 0.202 (0.154-0.246) | 0.194 (0.185-0.203) |
| AdaBoost+DT+RF+LightGBM+SVM+CatBoost | 76.286 | Rank 95 | 0.682 (0.661-0.703) | 0.342 (0.289-0.394) | 0.645 (0.605-0.686) | 0.463 (0.390-0.539) | 0.709 (0.686-0.730) | 0.196 (0.151-0.242) | 0.192 (0.183-0.201) |
| AdaBoost+DT+RF+NaiveBayes+SVM | 77.571 | Rank 96 | 0.670 (0.648-0.691) | 0.357 (0.304-0.407) | 0.641 (0.603-0.682) | 0.497 (0.429-0.569) | 0.692 (0.668-0.714) | 0.209 (0.160-0.255) | 0.209 (0.201-0.218) |
| RF+NaiveBayes+SVM+CatBoost | 77.571 | Rank 96 | 0.671 (0.652-0.691) | 0.358 (0.306-0.408) | 0.647 (0.609-0.685) | 0.497 (0.427-0.562) | 0.693 (0.673-0.715) | 0.196 (0.156-0.244) | 0.203 (0.195-0.211) |
| AdaBoost | 79.714 | Rank 98 | 0.626 (0.606-0.647) | 0.390 (0.341-0.434) | 0.643 (0.606-0.683) | 0.587 (0.522-0.653) | 0.631 (0.609-0.653) | 0.186 (0.145-0.225) | 0.218 (0.211-0.224) |
| AdaBoost+DT+LightGBM+NaiveBayes+CatBoost | 80.000 | Rank 99 | 0.694 (0.672-0.717) | 0.336 (0.280-0.386) | 0.628 (0.584-0.670) | 0.446 (0.373-0.514) | 0.726 (0.704-0.748) | 0.198 (0.154-0.242) | 0.195 (0.186-0.204) |
| AdaBoost+DT+SVM | 80.714 | Rank 100 | 0.644 (0.622-0.667) | 0.360 (0.310-0.407) | 0.653 (0.614-0.690) | 0.521 (0.449-0.590) | 0.660 (0.637-0.682) | 0.187 (0.148-0.227) | 0.221 (0.215-0.227) |
| SVM | 80.857 | Rank 101 | 0.636 (0.613-0.657) | 0.372 (0.319-0.418) | 0.639 (0.597-0.677) | 0.548 (0.480-0.615) | 0.647 (0.624-0.670) | 0.198 (0.152-0.241) | 0.228 (0.224-0.233) |
| DT+RF+LightGBM+SVM+CatBoost | 82.143 | Rank 102 | 0.697 (0.675-0.718) | 0.332 (0.281-0.378) | 0.629 (0.588-0.667) | 0.438 (0.370-0.500) | 0.729 (0.707-0.752) | 0.191 (0.146-0.236) | 0.196 (0.187-0.205) |
| NaiveBayes+CatBoost | 83.286 | Rank 103 | 0.696 (0.677-0.717) | 0.324 (0.274-0.370) | 0.628 (0.587-0.669) | 0.427 (0.361-0.488) | 0.730 (0.710-0.752) | 0.193 (0.149-0.239) | 0.193 (0.185-0.200) |
| AdaBoost+DT+RF+LightGBM+SVM | 84.429 | Rank 104 | 0.687 (0.665-0.707) | 0.350 (0.299-0.400) | 0.620 (0.582-0.662) | 0.473 (0.403-0.542) | 0.714 (0.691-0.734) | 0.194 (0.151-0.234) | 0.198 (0.190-0.207) |
| DT+LightGBM | 84.429 | Rank 104 | 0.680 (0.660-0.701) | 0.345 (0.292-0.391) | 0.639 (0.598-0.676) | 0.470 (0.400-0.535) | 0.707 (0.685-0.729) | 0.207 (0.158-0.260) | 0.207 (0.198-0.215) |
| DT+NaiveBayes+SVM | 85.143 | Rank 106 | 0.677 (0.655-0.699) | 0.345 (0.293-0.393) | 0.641 (0.602-0.682) | 0.473 (0.400-0.544) | 0.703 (0.680-0.725) | 0.205 (0.160-0.250) | 0.219 (0.211-0.226) |
| RF+LightGBM+SVM+CatBoost | 85.429 | Rank 107 | 0.677 (0.656-0.698) | 0.328 (0.273-0.376) | 0.648 (0.608-0.685) | 0.446 (0.375-0.514) | 0.706 (0.686-0.727) | 0.201 (0.152-0.250) | 0.196 (0.187-0.205) |
| NaiveBayes+SVM | 87.286 | Rank 108 | 0.649 (0.629-0.671) | 0.362 (0.310-0.411) | 0.640 (0.600-0.681) | 0.521 (0.451-0.592) | 0.665 (0.645-0.689) | 0.188 (0.147-0.229) | 0.230 (0.224-0.236) |
| LightGBM+NaiveBayes+SVM | 87.857 | Rank 109 | 0.697 (0.677-0.717) | 0.325 (0.271-0.376) | 0.621 (0.576-0.662) | 0.427 (0.360-0.500) | 0.731 (0.711-0.752) | 0.184 (0.142-0.228) | 0.195 (0.187-0.203) |
| AdaBoost+DT+LightGBM+NaiveBayes | 89.571 | Rank 110 | 0.705 (0.684-0.726) | 0.322 (0.271-0.370) | 0.616 (0.575-0.653) | 0.419 (0.348-0.486) | 0.741 (0.719-0.762) | 0.162 (0.129-0.195) | 0.197 (0.189-0.205) |
| AdaBoost+SVM | 89.714 | Rank 111 | 0.608 (0.585-0.629) | 0.372 (0.321-0.418) | 0.633 (0.591-0.672) | 0.570 (0.500-0.638) | 0.612 (0.588-0.636) | 0.174 (0.136-0.212) | 0.229 (0.221-0.237) |
| AdaBoost+DT+RF+LightGBM+NaiveBayes+CatBoost | 89.857 | Rank 112 | 0.691 (0.671-0.712) | 0.323 (0.272-0.373) | 0.625 (0.587-0.666) | 0.428 (0.360-0.500) | 0.724 (0.702-0.746) | 0.195 (0.149-0.240) | 0.196 (0.187-0.204) |
| AdaBoost+DT | 90.429 | Rank 113 | 0.621 (0.599-0.643) | 0.374 (0.324-0.421) | 0.630 (0.592-0.667) | 0.564 (0.493-0.635) | 0.629 (0.605-0.651) | 0.159 (0.128-0.190) | 0.236 (0.230-0.242) |
| AdaBoost+LightGBM+SVM | 91.714 | Rank 114 | 0.698 (0.678-0.718) | 0.326 (0.275-0.374) | 0.616 (0.575-0.656) | 0.428 (0.358-0.494) | 0.732 (0.711-0.754) | 0.160 (0.129-0.191) | 0.198 (0.190-0.206) |
| AdaBoost+LightGBM | 92.714 | Rank 115 | 0.671 (0.650-0.692) | 0.348 (0.297-0.393) | 0.638 (0.601-0.674) | 0.480 (0.412-0.545) | 0.695 (0.672-0.717) | 0.187 (0.147-0.227) | 0.205 (0.198-0.212) |
| DT+RF+LightGBM+CatBoost | 92.857 | Rank 116 | 0.687 (0.666-0.708) | 0.331 (0.274-0.383) | 0.616 (0.572-0.657) | 0.444 (0.370-0.515) | 0.717 (0.696-0.739) | 0.202 (0.153-0.253) | 0.201 (0.192-0.211) |
| RF+LightGBM | 92.857 | Rank 116 | 0.681 (0.658-0.702) | 0.331 (0.278-0.379) | 0.642 (0.601-0.679) | 0.446 (0.378-0.514) | 0.710 (0.687-0.733) | 0.187 (0.146-0.231) | 0.199 (0.191-0.208) |
| AdaBoost+LightGBM+NaiveBayes | 94.143 | Rank 118 | 0.675 (0.654-0.695) | 0.354 (0.303-0.403) | 0.623 (0.582-0.664) | 0.489 (0.425-0.561) | 0.699 (0.676-0.720) | 0.183 (0.141-0.223) | 0.208 (0.200-0.217) |
| DT+CatBoost | 94.571 | Rank 119 | 0.679 (0.658-0.701) | 0.334 (0.284-0.385) | 0.636 (0.595-0.677) | 0.454 (0.387-0.521) | 0.708 (0.686-0.731) | 0.182 (0.143-0.220) | 0.196 (0.188-0.203) |
| DT+LightGBM+NaiveBayes+CatBoost | 94.714 | Rank 120 | 0.684 (0.664-0.706) | 0.341 (0.285-0.394) | 0.629 (0.588-0.668) | 0.461 (0.389-0.531) | 0.712 (0.692-0.735) | 0.183 (0.144-0.228) | 0.202 (0.193-0.211) |
| AdaBoost+RF+LightGBM+NaiveBayes | 96.143 | Rank 121 | 0.687 (0.667-0.708) | 0.295 (0.238-0.342) | 0.624 (0.585-0.663) | 0.388 (0.317-0.456) | 0.725 (0.704-0.746) | 0.190 (0.146-0.234) | 0.194 (0.186-0.203) |
| DT+RF+LightGBM+NaiveBayes+SVM | 97.714 | Rank 122 | 0.680 (0.659-0.701) | 0.296 (0.246-0.343) | 0.623 (0.582-0.661) | 0.394 (0.328-0.458) | 0.716 (0.695-0.737) | 0.216 (0.165-0.267) | 0.204 (0.194-0.213) |
| RF+CatBoost | 97.714 | Rank 122 | 0.660 (0.639-0.681) | 0.347 (0.295-0.394) | 0.632 (0.592-0.672) | 0.488 (0.416-0.554) | 0.681 (0.659-0.703) | 0.179 (0.142-0.218) | 0.203 (0.194-0.211) |
| AdaBoost+DT+LightGBM | 102.857 | Rank 124 | 0.668 (0.646-0.689) | 0.339 (0.286-0.391) | 0.630 (0.589-0.671) | 0.469 (0.400-0.541) | 0.693 (0.670-0.714) | 0.184 (0.142-0.226) | 0.207 (0.200-0.215) |
| LightGBM | 104.857 | Rank 125 | 0.680 (0.657-0.702) | 0.308 (0.253-0.356) | 0.628 (0.584-0.665) | 0.413 (0.342-0.479) | 0.714 (0.689-0.736) | 0.198 (0.151-0.245) | 0.210 (0.199-0.222) |
| KNN | 112.000 | Rank 126 | 0.600 (0.577-0.622) | 0.327 (0.279-0.373) | 0.583 (0.541-0.624) | 0.496 (0.429-0.562) | 0.613 (0.589-0.635) | 0.149 (0.122-0.181) | 0.276 (0.262-0.290) |
| AdaBoost+RF+LightGBM | 116.857 | Rank 127 | 0.667 (0.647-0.690) | 0.307 (0.256-0.358) | 0.612 (0.575-0.653) | 0.419 (0.354-0.486) | 0.698 (0.677-0.722) | 0.185 (0.139-0.228) | 0.205 (0.196-0.214) |
| DT | 120.571 | Rank 128 | 0.658 (0.637-0.679) | 0.318 (0.268-0.367) | 0.598 (0.562-0.637) | 0.443 (0.373-0.508) | 0.685 (0.664-0.709) | 0.147 (0.121-0.173) | 0.237 (0.229-0.244) |
| XGBoost | 123.857 | Rank 129 | 0.665 (0.644-0.686) | 0.286 (0.238-0.330) | 0.566 (0.525-0.610) | 0.388 (0.324-0.456) | 0.700 (0.678-0.722) | 0.153 (0.121-0.187) | 0.272 (0.256-0.289) |

**Supplementary Table 22.** Standardized mean differences before and after IPTW adjustment in the HRS cohort.

| Variable | Type | SMD_Before_IPTW | SMD_After_IPTW |
| --- | --- | --- | --- |
| prop.score | Distance | 0.624 | 0.035 |
| ragender | Binary | 0.000 | 0.017 |
| ragey_b | Contin. | 0.351 | 0.013 |
| race_1 | Binary | 0.087 | 0.019 |
| race_2 | Binary | 0.040 | 0.032 |
| race_3 | Binary | 0.047 | 0.010 |
| race_4 | Binary | 0.000 | 0.003 |
| raedyrs | Contin. | 0.345 | 0.010 |
| atotb | Contin. | 0.322 | 0.143 |
| bmi | Contin. | 0.263 | 0.120 |
| hibpe | Binary | 0.059 | 0.036 |
| pa | Binary | 0.096 | 0.001 |
| drink | Binary | 0.101 | 0.021 |
| smokev | Binary | 0.021 | 0.040 |
| cancre | Binary | 0.048 | 0.001 |
| hipe | Binary | 0.015 | 0.003 |
| CVD | Binary | 0.073 | 0.038 |
| cesd | Contin. | 0.192 | 0.066 |
| tcog_z_z | Contin. | 0.407 | 0.029 |

**Supplementary Table 23.** Standardized mean differences before and after IPTW adjustment in the ELSA cohort.

| Variable | Type | SMD_Before_IPTW | SMD_After_IPTW |
| --- | --- | --- | --- |
| prop.score | Distance | 1.080 | 0.009 |
| ragender | Binary | 0.104 | 0.138 |
| ragey_b | Contin. | 1.159 | 0.009 |
| race | Binary | 0.002 | 0.007 |
| raedyrs_1 | Binary | 0.258 | 0.075 |
| raedyrs_3 | Binary | 0.076 | 0.042 |
| raedyrs_4 | Binary | 0.095 | 0.007 |
| raedyrs_5 | Binary | 0.088 | 0.027 |
| atotb | Contin. | 0.388 | 0.071 |
| bmi | Contin. | 0.033 | 0.098 |
| hibpe | Binary | 0.056 | 0.047 |
| pa | Binary | 0.238 | 0.018 |
| drink | Binary | 0.075 | 0.012 |
| smokev | Binary | 0.019 | 0.097 |
| cancre | Binary | 0.027 | 0.001 |
| hipe | Binary | 0.019 | 0.002 |
| CVD | Binary | 0.039 | 0.010 |
| cesd | Contin. | 0.268 | 0.033 |

**Supplementary Table 24.** Standardized mean differences before and after IPTW adjustment in the SHARE cohort.

| Variable | Type | SMD_Before_IPTW | SMD_After_IPTW |
| --- | --- | --- | --- |
| prop.score | Distance | 0.619 | 0.010 |
| ragender | Binary | 0.036 | 0.004 |
| ragey_b | Contin. | 0.519 | 0.022 |
| race | Binary | 0.015 | 0.002 |
| raedyrs_1 | Binary | 0.080 | 0.014 |
| raedyrs_2 | Binary | 0.045 | 0.003 |
| raedyrs_3 | Binary | 0.035 | 0.011 |
| bmi | Contin. | 0.239 | 0.189 |
| hibpe | Binary | 0.047 | 0.002 |
| pa | Binary | 0.152 | 0.003 |
| smokev | Binary | 0.024 | 0.004 |
| cancre | Binary | 0.004 | 0.008 |
| hipe | Binary | 0.019 | 0.021 |
| CVD | Binary | 0.006 | 0.029 |
| cesd | Contin. | 0.300 | 0.002 |

**Supplementary Table 25.** Standardized mean differences before and after IPTW adjustment in the CHARLS cohort.

| Variable | Type | SMD_Before_IPTW | SMD_After_IPTW |
| --- | --- | --- | --- |
| prop.score | Distance | 0.583 | 0.008 |
| ragender | Binary | 0.117 | 0.005 |
| ragey_b | Contin. | 0.460 | 0.087 |
| race | Binary | 0.073 | 0.010 |
| raedyrs_1 | Binary | 0.003 | 0.018 |
| raedyrs_2 | Binary | 0.017 | 0.004 |
| raedyrs_3 | Binary | 0.014 | 0.014 |
| atotb | Contin. | 0.070 | 0.069 |
| bmi | Contin. | 0.107 | 0.000 |
| hibpe | Binary | 0.068 | 0.008 |
| pa | Binary | 0.089 | 0.016 |
| drink | Binary | 0.048 | 0.018 |
| smokev | Binary | 0.179 | 0.033 |
| cancre | Binary | 0.000 | 0.001 |
| hipe | Binary | 0.001 | 0.001 |
| CVD | Binary | 0.015 | 0.021 |
| cesd | Contin. | 0.215 | 0.083 |
| tcog_z_z | Contin. | 0.433 | 0.038 |

**Supplementary Table 26.** Benchmarking analysis when the HRS and ELSA cohorts were used as training datasets.

| **Model** | **Accuracy** | **F Beta** | **Area under the ROC curve** | **Sensitivity** | **Specificity** | **Area under the PR curve** | **Brier** |
| --- | --- | --- | --- | --- | --- | --- | --- |
| AdaBoost | 0.635 (0.608-0.663) | 0.400 (0.379-0.422) | 0.680 (0.655-0.706) | 0.619 (0.543-0.695) | 0.639 (0.599-0.678) | 0.202 (0.180-0.224) | 0.223 (0.218-0.229) |
| DT | 0.582 (0.541-0.623) | 0.404 (0.383-0.426) | 0.643 (0.618-0.668) | 0.674 (0.601-0.746) | 0.572 (0.519-0.625) | 0.162 (0.146-0.178) | 0.233 (0.227-0.240) |
| Random Forest | 0.716 (0.702-0.730) | 0.372 (0.338-0.406) | 0.686 (0.666-0.705) | 0.499 (0.438-0.561) | 0.743 (0.721-0.765) | 0.201 (0.183-0.218) | 0.186 (0.182-0.190) |
| Light GBM | 0.729 (0.715-0.742) | 0.383 (0.359-0.406) | 0.671 (0.650-0.692) | 0.501 (0.469-0.534) | 0.756 (0.738-0.774) | 0.211 (0.180-0.242) | 0.185 (0.178-0.192) |
| KNN | 0.614 (0.595-0.633) | 0.344 (0.311-0.378) | 0.609 (0.582-0.636) | 0.528 (0.487-0.569) | 0.625 (0.605-0.644) | 0.159 (0.137-0.181) | 0.269 (0.257-0.281) |
| Naive Bayes | 0.402 (0.363-0.441) | 0.399 (0.381-0.417) | 0.661 (0.635-0.687) | 0.816 (0.776-0.856) | 0.352 (0.303-0.401) | 0.205 (0.182-0.227) | 0.354 (0.331-0.377) |
| SVM | 0.638 (0.615-0.661) | 0.377 (0.358-0.396) | 0.660 (0.639-0.681) | 0.570 (0.531-0.609) | 0.647 (0.620-0.674) | 0.197 (0.165-0.228) | 0.219 (0.212-0.227) |
| XGBoost | 0.697 (0.686-0.709) | 0.334 (0.300-0.368) | 0.634 (0.603-0.666) | 0.455 (0.395-0.514) | 0.727 (0.709-0.746) | 0.171 (0.155-0.186) | 0.241 (0.229-0.252) |
| CATBoost | 0.728 (0.715-0.741) | 0.352 (0.316-0.388) | 0.670 (0.642-0.698) | 0.459 (0.393-0.526) | 0.761 (0.744-0.779) | 0.196 (0.176-0.216) | 0.181 (0.175-0.186) |
| P | <.001^a^ | <.001^a^ | <.001^b^ | <.001^a^ | <.001^a^ | <.001^a^ | <.001^a^ |
| ^a^ANOVA test; ^b^Kruskal-Wallis | | |  |  |  |  |  |
|  | | | | | | |  |

**Supplementary Table 27.** Benchmarking analysis when the SHARE cohort was used as the validation dataset.

| **Model** | **Accuracy** | **F Beta** | **Area under the ROC curve** | **Sensitivity** | **Specificity** | **Area under the PR curve** | **Brier** |
| --- | --- | --- | --- | --- | --- | --- | --- |
| AdaBoost | 0.765 (0.749-0.780) | 0.276 (0.230-0.320) | 0.629 (0.595-0.662) | 0.319 (0.263-0.374) | 0.820 (0.805-0.835) | 0.161 (0.136-0.187) | 0.169 (0.163-0.176) |
| DT | 0.595 (0.575-0.613) | 0.376 (0.335-0.414) | 0.606 (0.571-0.642) | 0.589 (0.532-0.644) | 0.595 (0.575-0.616) | 0.156 (0.132-0.181) | 0.207 (0.200-0.214) |
| Random Forest | 0.811 (0.796-0.826) | 0.179 (0.132-0.222) | 0.622 (0.588-0.655) | 0.182 (0.133-0.225) | 0.890 (0.878-0.902) | 0.174 (0.139-0.208) | 0.152 (0.147-0.158) |
| Light GBM | 0.759 (0.742-0.776) | 0.232 (0.189-0.276) | 0.585 (0.553-0.616) | 0.264 (0.215-0.315) | 0.821 (0.806-0.837) | 0.140 (0.119-0.163) | 0.167 (0.159-0.176) |
| KNN | 0.635 (0.617-0.655) | 0.241 (0.201-0.282) | 0.524 (0.491-0.557) | 0.335 (0.283-0.394) | 0.673 (0.654-0.692) | 0.114 (0.099-0.130) | 0.247 (0.235-0.258) |
| Naive Bayes | 0.543 (0.524-0.561) | 0.368 (0.329-0.404) | 0.611 (0.579-0.646) | 0.616 (0.561-0.673) | 0.533 (0.514-0.553) | 0.186 (0.150-0.226) | 0.277 (0.269-0.285) |
| SVM | 0.759 (0.743-0.775) | 0.236 (0.189-0.277) | 0.595 (0.561-0.630) | 0.270 (0.218-0.320) | 0.820 (0.804-0.836) | 0.161 (0.129-0.195) | 0.171 (0.165-0.177) |
| XGBoost | 0.754 (0.738-0.770) | 0.234 (0.191-0.278) | 0.592 (0.558-0.627) | 0.269 (0.218-0.321) | 0.815 (0.800-0.831) | 0.149 (0.125-0.175) | 0.197 (0.184-0.209) |
| CATBoost | 0.798 (0.783-0.813) | 0.196 (0.150-0.234) | 0.604 (0.572-0.638) | 0.205 (0.157-0.248) | 0.872 (0.859-0.886) | 0.172 (0.137-0.203) | 0.150 (0.142-0.157) |
| P | <.001^a^ | <.001^a^ | <.001^b^ | <.001^a^ | <.001^a^ | <.001^a^ | <.001^a^ |
| ^a^ANOVA test; ^b^Kruskal-Wallis | | |  |  |  |  |  |
|  | | | | | | |  |

**Supplementary Table 28.** Benchmarking analysis when the CHARLS cohort was used as the testing dataset.

| **Model** | **Accuracy** | **F Beta** | **Area under the ROC curve** | **Sensitivity** | **Specificity** | **Area under the PR curve** | **Brier** |
| --- | --- | --- | --- | --- | --- | --- | --- |
| AdaBoost | 0.791 (0.781-0.801) | 0.298 (0.270-0.328) | 0.636 (0.615-0.657) | 0.315 (0.284-0.349) | 0.859 (0.850-0.868) | 0.213 (0.189-0.236) | 0.181 (0.177-0.184) |
| DT | 0.737 (0.726-0.748) | 0.342 (0.313-0.368) | 0.610 (0.590-0.630) | 0.402 (0.367-0.436) | 0.786 (0.775-0.796) | 0.194 (0.176-0.212) | 0.166 (0.161-0.171) |
| Random Forest | 0.788 (0.777-0.797) | 0.221 (0.193-0.247) | 0.596 (0.574-0.617) | 0.227 (0.199-0.254) | 0.868 (0.860-0.877) | 0.173 (0.154-0.190) | 0.181 (0.178-0.184) |
| Light GBM | 0.802 (0.792-0.813) | 0.212 (0.181-0.240) | 0.592 (0.570-0.613) | 0.212 (0.181-0.241) | 0.887 (0.879-0.896) | 0.178 (0.159-0.197) | 0.146 (0.141-0.152) |
| KNN | 0.585 (0.573-0.597) | 0.302 (0.276-0.326) | 0.524 (0.501-0.547) | 0.431 (0.397-0.468) | 0.607 (0.595-0.621) | 0.149 (0.133-0.166) | 0.279 (0.272-0.286) |
| Naive Bayes | 0.685 (0.673-0.696) | 0.297 (0.268-0.324) | 0.560 (0.536-0.581) | 0.370 (0.336-0.404) | 0.731 (0.718-0.742) | 0.161 (0.144-0.179) | 0.210 (0.204-0.215) |
| SVM | 0.791 (0.781-0.801) | 0.164 (0.140-0.187) | 0.557 (0.537-0.578) | 0.163 (0.139-0.188) | 0.882 (0.874-0.892) | 0.148 (0.133-0.163) | 0.173 (0.169-0.176) |
| XGBoost | 0.709 (0.697-0.720) | 0.259 (0.232-0.283) | 0.539 (0.516-0.561) | 0.307 (0.275-0.337) | 0.767 (0.756-0.778) | 0.153 (0.137-0.170) | 0.237 (0.228-0.246) |
| CATBoost | 0.744 (0.734-0.756) | 0.293 (0.265-0.320) | 0.590 (0.568-0.611) | 0.333 (0.301-0.366) | 0.804 (0.793-0.814) | 0.181 (0.162-0.200) | 0.182 (0.177-0.186) |
| P | <.001^a^ | <.001^a^ | <.001^b^ | <.001^a^ | <.001^a^ | <.001^a^ | <.001^a^ |
| ^a^ANOVA test; ^b^Kruskal-Wallis | | |  |  |  |  |  |
|  | | | | | | |  |

**Supplementary Table 29.** Ranking of 129 machine learning models when the HRS and ELSA cohorts were used as training datasets.

| **Model** | **Normalized Rank Score** | **Comprehensive Rank** | **Accuracy (95% CI)** | **F_Beta (95% CI)** | **ROC_AUC (95% CI)** | **Sensitivity (95% CI)** | **Specificity (95% CI)** | **PR_AUC (95% CI)** | **Brier (95% CI)** |
| --- | --- | --- | --- | --- | --- | --- | --- | --- | --- |
| AdaBoost+DT+RF+NaiveBayes+SVM | 5.000 | Rank 1 | 0.838 (0.831-0.845) | 0.748 (0.733-0.764) | 0.958 (0.954-0.963) | 0.964 (0.953-0.976) | 0.823 (0.815-0.830) | 0.716 (0.687-0.745) | 0.123 (0.120-0.126) |
| AdaBoost+RF+LightGBM | 6.286 | Rank 2 | 0.836 (0.828-0.843) | 0.741 (0.725-0.756) | 0.956 (0.951-0.960) | 0.954 (0.941-0.967) | 0.822 (0.813-0.830) | 0.709 (0.681-0.736) | 0.119 (0.116-0.122) |
| AdaBoost+DT+RF+LightGBM+NaiveBayes+SVM | 10.000 | Rank 3 | 0.831 (0.823-0.838) | 0.737 (0.720-0.754) | 0.955 (0.950-0.960) | 0.957 (0.945-0.970) | 0.816 (0.808-0.824) | 0.722 (0.694-0.749) | 0.122 (0.119-0.126) |
| AdaBoost+RF+LightGBM+SVM | 10.714 | Rank 4 | 0.836 (0.828-0.844) | 0.741 (0.725-0.756) | 0.952 (0.948-0.957) | 0.954 (0.941-0.966) | 0.822 (0.814-0.830) | 0.682 (0.653-0.711) | 0.122 (0.118-0.125) |
| DT+RF+SVM | 11.429 | Rank 5 | 0.834 (0.827-0.842) | 0.735 (0.720-0.751) | 0.954 (0.950-0.959) | 0.947 (0.934-0.960) | 0.820 (0.812-0.829) | 0.726 (0.699-0.751) | 0.125 (0.122-0.128) |
| RF+LightGBM+NaiveBayes+SVM | 11.571 | Rank 6 | 0.842 (0.835-0.849) | 0.746 (0.730-0.761) | 0.949 (0.944-0.954) | 0.952 (0.940-0.965) | 0.828 (0.821-0.836) | 0.668 (0.636-0.698) | 0.123 (0.119-0.126) |
| AdaBoost+DT+RF+NaiveBayes+SVM+CatBoost | 13.286 | Rank 7 | 0.840 (0.832-0.847) | 0.738 (0.720-0.753) | 0.946 (0.940-0.951) | 0.940 (0.927-0.955) | 0.828 (0.820-0.835) | 0.670 (0.640-0.701) | 0.120 (0.116-0.123) |
| DT+RF+LightGBM+NaiveBayes | 13.286 | Rank 7 | 0.838 (0.830-0.845) | 0.737 (0.720-0.752) | 0.948 (0.943-0.953) | 0.943 (0.929-0.956) | 0.825 (0.817-0.833) | 0.688 (0.657-0.718) | 0.124 (0.121-0.127) |
| AdaBoost+RF+LightGBM+NaiveBayes+SVM | 16.286 | Rank 9 | 0.829 (0.823-0.837) | 0.727 (0.710-0.744) | 0.950 (0.945-0.955) | 0.941 (0.926-0.956) | 0.816 (0.809-0.824) | 0.698 (0.670-0.728) | 0.121 (0.117-0.124) |
| AdaBoost+RF+LightGBM+NaiveBayes+CatBoost | 17.571 | Rank 10 | 0.834 (0.826-0.841) | 0.726 (0.708-0.743) | 0.945 (0.940-0.951) | 0.931 (0.915-0.946) | 0.822 (0.813-0.830) | 0.686 (0.656-0.717) | 0.122 (0.118-0.125) |
| DT+RF+LightGBM+NaiveBayes+SVM+CatBoost | 17.714 | Rank 11 | 0.833 (0.826-0.841) | 0.722 (0.705-0.739) | 0.947 (0.942-0.952) | 0.926 (0.910-0.942) | 0.822 (0.814-0.830) | 0.684 (0.652-0.715) | 0.120 (0.116-0.123) |
| DT+RF+LightGBM+SVM | 17.714 | Rank 11 | 0.829 (0.821-0.836) | 0.732 (0.714-0.748) | 0.951 (0.946-0.956) | 0.951 (0.938-0.965) | 0.814 (0.806-0.823) | 0.688 (0.659-0.719) | 0.125 (0.122-0.129) |
| AdaBoost+DT+RF | 18.571 | Rank 13 | 0.823 (0.815-0.830) | 0.727 (0.710-0.744) | 0.952 (0.947-0.957) | 0.954 (0.941-0.966) | 0.807 (0.799-0.815) | 0.706 (0.678-0.734) | 0.125 (0.121-0.128) |
| AdaBoost+DT+RF+LightGBM+SVM+CatBoost | 20.857 | Rank 14 | 0.833 (0.826-0.840) | 0.723 (0.706-0.738) | 0.944 (0.938-0.949) | 0.926 (0.911-0.942) | 0.822 (0.814-0.830) | 0.672 (0.642-0.699) | 0.123 (0.120-0.126) |
| AdaBoost+RF+NaiveBayes+SVM+CatBoost | 23.143 | Rank 15 | 0.825 (0.817-0.833) | 0.717 (0.700-0.733) | 0.949 (0.944-0.955) | 0.931 (0.916-0.946) | 0.812 (0.803-0.821) | 0.702 (0.671-0.732) | 0.125 (0.122-0.128) |
| AdaBoost+RF+LightGBM+SVM+CatBoost | 23.857 | Rank 16 | 0.830 (0.823-0.838) | 0.717 (0.700-0.734) | 0.942 (0.937-0.948) | 0.922 (0.906-0.939) | 0.819 (0.811-0.827) | 0.666 (0.633-0.697) | 0.121 (0.118-0.125) |
| RF+SVM | 24.714 | Rank 17 | 0.816 (0.808-0.823) | 0.727 (0.712-0.742) | 0.955 (0.950-0.959) | 0.965 (0.955-0.977) | 0.798 (0.790-0.806) | 0.712 (0.684-0.741) | 0.129 (0.126-0.132) |
| AdaBoost+DT+RF+CatBoost | 25.429 | Rank 18 | 0.831 (0.823-0.838) | 0.710 (0.691-0.728) | 0.941 (0.936-0.947) | 0.907 (0.891-0.925) | 0.822 (0.813-0.830) | 0.670 (0.638-0.699) | 0.121 (0.118-0.124) |
| AdaBoost+DT+RF+LightGBM+SVM | 25.714 | Rank 19 | 0.820 (0.812-0.828) | 0.724 (0.706-0.739) | 0.949 (0.944-0.955) | 0.953 (0.939-0.966) | 0.804 (0.796-0.812) | 0.688 (0.660-0.716) | 0.127 (0.123-0.130) |
| AdaBoost+DT+RF+LightGBM+NaiveBayes+CatBoost | 25.857 | Rank 20 | 0.831 (0.823-0.838) | 0.713 (0.696-0.730) | 0.941 (0.936-0.947) | 0.912 (0.895-0.929) | 0.821 (0.813-0.829) | 0.656 (0.628-0.685) | 0.123 (0.119-0.126) |
| AdaBoost+RF+NaiveBayes+SVM | 26.714 | Rank 21 | 0.817 (0.809-0.825) | 0.724 (0.707-0.740) | 0.951 (0.945-0.956) | 0.959 (0.947-0.971) | 0.800 (0.791-0.808) | 0.714 (0.686-0.741) | 0.130 (0.127-0.134) |
| DT+RF+NaiveBayes+SVM+CatBoost | 27.000 | Rank 22 | 0.833 (0.826-0.840) | 0.706 (0.688-0.723) | 0.937 (0.931-0.943) | 0.898 (0.880-0.916) | 0.825 (0.817-0.832) | 0.658 (0.629-0.686) | 0.123 (0.120-0.126) |
| RF+NaiveBayes+SVM | 27.143 | Rank 23 | 0.820 (0.813-0.828) | 0.723 (0.706-0.739) | 0.951 (0.946-0.956) | 0.951 (0.938-0.964) | 0.804 (0.796-0.813) | 0.697 (0.666-0.725) | 0.131 (0.128-0.134) |
| RF+LightGBM+SVM | 27.429 | Rank 24 | 0.824 (0.815-0.832) | 0.721 (0.703-0.737) | 0.945 (0.940-0.951) | 0.940 (0.927-0.955) | 0.810 (0.801-0.819) | 0.676 (0.645-0.705) | 0.128 (0.124-0.131) |
| AdaBoost+DT+RF+LightGBM | 27.571 | Rank 25 | 0.820 (0.813-0.828) | 0.728 (0.712-0.743) | 0.949 (0.944-0.954) | 0.961 (0.949-0.972) | 0.804 (0.796-0.812) | 0.666 (0.637-0.697) | 0.130 (0.127-0.134) |
| AdaBoost+DT+RF+SVM | 27.571 | Rank 25 | 0.820 (0.812-0.828) | 0.717 (0.700-0.733) | 0.951 (0.946-0.956) | 0.940 (0.926-0.956) | 0.806 (0.797-0.814) | 0.705 (0.678-0.732) | 0.128 (0.125-0.131) |
| AdaBoost+DT+RF+LightGBM+NaiveBayes | 28.143 | Rank 27 | 0.820 (0.812-0.827) | 0.713 (0.697-0.729) | 0.947 (0.941-0.952) | 0.933 (0.918-0.947) | 0.807 (0.798-0.815) | 0.697 (0.668-0.725) | 0.124 (0.121-0.128) |
| AdaBoost+RF+SVM | 28.286 | Rank 28 | 0.816 (0.808-0.823) | 0.724 (0.708-0.740) | 0.951 (0.946-0.956) | 0.962 (0.951-0.974) | 0.798 (0.790-0.806) | 0.707 (0.677-0.735) | 0.131 (0.127-0.134) |
| DT+RF+NaiveBayes | 28.429 | Rank 29 | 0.819 (0.812-0.827) | 0.717 (0.701-0.733) | 0.950 (0.945-0.955) | 0.942 (0.929-0.956) | 0.805 (0.797-0.813) | 0.699 (0.672-0.725) | 0.130 (0.126-0.133) |
| AdaBoost+RF+SVM+CatBoost | 29.429 | Rank 30 | 0.831 (0.824-0.839) | 0.705 (0.688-0.723) | 0.933 (0.928-0.939) | 0.898 (0.880-0.916) | 0.823 (0.816-0.831) | 0.634 (0.604-0.665) | 0.123 (0.120-0.126) |
| RF+LightGBM+NaiveBayes+SVM+CatBoost | 29.714 | Rank 31 | 0.827 (0.820-0.835) | 0.711 (0.694-0.728) | 0.938 (0.932-0.944) | 0.915 (0.899-0.932) | 0.817 (0.809-0.825) | 0.637 (0.607-0.670) | 0.124 (0.121-0.128) |
| AdaBoost+DT+RF+SVM+CatBoost | 30.143 | Rank 32 | 0.831 (0.823-0.839) | 0.706 (0.688-0.724) | 0.935 (0.929-0.941) | 0.901 (0.883-0.919) | 0.822 (0.814-0.830) | 0.626 (0.593-0.655) | 0.124 (0.121-0.128) |
| DT+RF+LightGBM+NaiveBayes+CatBoost | 30.143 | Rank 32 | 0.826 (0.819-0.833) | 0.710 (0.692-0.726) | 0.937 (0.931-0.943) | 0.917 (0.901-0.935) | 0.815 (0.807-0.822) | 0.668 (0.638-0.695) | 0.125 (0.122-0.128) |
| AdaBoost+RF+NaiveBayes | 32.429 | Rank 34 | 0.810 (0.802-0.818) | 0.722 (0.706-0.738) | 0.955 (0.950-0.960) | 0.968 (0.958-0.979) | 0.791 (0.783-0.799) | 0.708 (0.683-0.738) | 0.133 (0.129-0.137) |
| DT+RF+LightGBM+NaiveBayes+SVM | 32.857 | Rank 35 | 0.823 (0.815-0.831) | 0.722 (0.705-0.738) | 0.943 (0.938-0.948) | 0.944 (0.929-0.958) | 0.808 (0.800-0.817) | 0.621 (0.590-0.653) | 0.133 (0.129-0.136) |
| AdaBoost+RF+LightGBM+NaiveBayes+SVM+CatBoost | 38.429 | Rank 36 | 0.822 (0.815-0.830) | 0.696 (0.679-0.713) | 0.933 (0.926-0.939) | 0.898 (0.879-0.916) | 0.813 (0.805-0.822) | 0.639 (0.609-0.669) | 0.127 (0.124-0.130) |
| AdaBoost+RF+LightGBM+CatBoost | 38.571 | Rank 37 | 0.822 (0.814-0.829) | 0.698 (0.681-0.715) | 0.931 (0.925-0.937) | 0.902 (0.885-0.921) | 0.812 (0.804-0.820) | 0.628 (0.597-0.660) | 0.128 (0.125-0.132) |
| DT+RF+NaiveBayes+SVM | 39.000 | Rank 38 | 0.813 (0.805-0.821) | 0.714 (0.697-0.732) | 0.947 (0.942-0.953) | 0.949 (0.936-0.964) | 0.797 (0.789-0.805) | 0.683 (0.652-0.716) | 0.134 (0.130-0.137) |
| AdaBoost+DT+RF+LightGBM+NaiveBayes+SVM+CatBoost | 39.857 | Rank 39 | 0.819 (0.812-0.827) | 0.692 (0.673-0.709) | 0.933 (0.927-0.940) | 0.896 (0.878-0.915) | 0.810 (0.802-0.818) | 0.640 (0.610-0.672) | 0.126 (0.122-0.129) |
| AdaBoost+DT+RF+NaiveBayes | 40.571 | Rank 40 | 0.805 (0.797-0.813) | 0.709 (0.692-0.725) | 0.952 (0.947-0.957) | 0.952 (0.939-0.966) | 0.788 (0.779-0.796) | 0.724 (0.695-0.749) | 0.135 (0.131-0.138) |
| AdaBoost+RF+NaiveBayes+CatBoost | 40.571 | Rank 40 | 0.823 (0.816-0.831) | 0.685 (0.664-0.703) | 0.926 (0.919-0.932) | 0.877 (0.856-0.898) | 0.817 (0.809-0.826) | 0.619 (0.587-0.648) | 0.126 (0.122-0.129) |
| RF+LightGBM | 41.714 | Rank 42 | 0.818 (0.811-0.826) | 0.696 (0.677-0.715) | 0.933 (0.927-0.939) | 0.906 (0.888-0.923) | 0.808 (0.800-0.816) | 0.626 (0.595-0.656) | 0.130 (0.127-0.133) |
| DT+RF | 42.286 | Rank 43 | 0.807 (0.800-0.815) | 0.716 (0.700-0.731) | 0.947 (0.942-0.952) | 0.962 (0.951-0.973) | 0.789 (0.780-0.797) | 0.684 (0.655-0.714) | 0.135 (0.132-0.139) |
| RF+LightGBM+NaiveBayes | 43.714 | Rank 44 | 0.818 (0.811-0.826) | 0.699 (0.682-0.717) | 0.933 (0.927-0.939) | 0.911 (0.894-0.930) | 0.807 (0.799-0.815) | 0.618 (0.585-0.647) | 0.133 (0.130-0.136) |
| DT+RF+LightGBM+SVM+CatBoost | 44.000 | Rank 45 | 0.818 (0.811-0.826) | 0.698 (0.681-0.716) | 0.928 (0.921-0.935) | 0.909 (0.892-0.928) | 0.807 (0.799-0.815) | 0.611 (0.580-0.640) | 0.132 (0.128-0.135) |
| DT+RF+LightGBM | 44.714 | Rank 46 | 0.813 (0.806-0.821) | 0.711 (0.694-0.728) | 0.940 (0.935-0.945) | 0.942 (0.929-0.958) | 0.798 (0.789-0.806) | 0.634 (0.602-0.665) | 0.134 (0.130-0.138) |
| AdaBoost+DT+RF+LightGBM+CatBoost | 46.571 | Rank 47 | 0.816 (0.809-0.825) | 0.690 (0.672-0.707) | 0.929 (0.923-0.936) | 0.898 (0.880-0.916) | 0.806 (0.798-0.815) | 0.621 (0.588-0.651) | 0.131 (0.128-0.135) |
| RF+NaiveBayes+SVM+CatBoost | 47.143 | Rank 48 | 0.816 (0.808-0.824) | 0.687 (0.668-0.705) | 0.929 (0.922-0.935) | 0.894 (0.875-0.912) | 0.807 (0.798-0.815) | 0.610 (0.580-0.642) | 0.130 (0.126-0.133) |
| AdaBoost+RF | 47.571 | Rank 49 | 0.805 (0.798-0.814) | 0.707 (0.690-0.723) | 0.945 (0.939-0.951) | 0.949 (0.936-0.963) | 0.788 (0.780-0.797) | 0.686 (0.655-0.717) | 0.137 (0.133-0.140) |
| DT+RF+LightGBM+CatBoost | 48.429 | Rank 50 | 0.814 (0.807-0.823) | 0.686 (0.667-0.705) | 0.929 (0.923-0.936) | 0.894 (0.876-0.914) | 0.805 (0.796-0.813) | 0.619 (0.587-0.653) | 0.130 (0.127-0.134) |
| AdaBoost+RF+LightGBM+NaiveBayes | 49.143 | Rank 51 | 0.807 (0.798-0.814) | 0.706 (0.690-0.723) | 0.943 (0.938-0.949) | 0.946 (0.932-0.961) | 0.790 (0.781-0.798) | 0.665 (0.635-0.695) | 0.135 (0.132-0.139) |
| AdaBoost+LightGBM+NaiveBayes+SVM+CatBoost | 51.429 | Rank 52 | 0.826 (0.818-0.833) | 0.664 (0.644-0.682) | 0.903 (0.894-0.911) | 0.836 (0.813-0.858) | 0.824 (0.816-0.832) | 0.525 (0.494-0.559) | 0.134 (0.131-0.137) |
| AdaBoost+DT+LightGBM+SVM+CatBoost | 52.429 | Rank 53 | 0.829 (0.821-0.837) | 0.640 (0.619-0.660) | 0.899 (0.890-0.907) | 0.790 (0.766-0.814) | 0.834 (0.826-0.842) | 0.528 (0.496-0.560) | 0.130 (0.127-0.133) |
| RF+SVM+CatBoost | 53.857 | Rank 54 | 0.811 (0.803-0.819) | 0.682 (0.664-0.699) | 0.922 (0.916-0.929) | 0.893 (0.875-0.912) | 0.801 (0.793-0.810) | 0.609 (0.577-0.642) | 0.133 (0.129-0.136) |
| DT+LightGBM+SVM+CatBoost | 54.286 | Rank 55 | 0.822 (0.815-0.830) | 0.654 (0.633-0.672) | 0.901 (0.893-0.909) | 0.824 (0.801-0.846) | 0.822 (0.814-0.831) | 0.508 (0.475-0.543) | 0.133 (0.130-0.136) |
| DT+RF+CatBoost | 55.000 | Rank 56 | 0.814 (0.806-0.822) | 0.659 (0.639-0.678) | 0.912 (0.904-0.919) | 0.846 (0.825-0.868) | 0.811 (0.802-0.818) | 0.551 (0.517-0.587) | 0.132 (0.129-0.136) |
| RF+NaiveBayes | 56.571 | Rank 57 | 0.803 (0.795-0.811) | 0.697 (0.679-0.715) | 0.939 (0.933-0.945) | 0.936 (0.921-0.951) | 0.787 (0.779-0.796) | 0.651 (0.618-0.681) | 0.139 (0.135-0.143) |
| AdaBoost+RF+CatBoost | 56.857 | Rank 58 | 0.809 (0.801-0.817) | 0.679 (0.661-0.697) | 0.925 (0.919-0.932) | 0.892 (0.874-0.910) | 0.799 (0.790-0.808) | 0.589 (0.555-0.622) | 0.134 (0.130-0.137) |
| LightGBM+NaiveBayes+CatBoost | 58.143 | Rank 59 | 0.821 (0.813-0.828) | 0.642 (0.621-0.662) | 0.897 (0.888-0.905) | 0.807 (0.783-0.830) | 0.823 (0.815-0.831) | 0.528 (0.493-0.560) | 0.134 (0.131-0.137) |
| RF+LightGBM+CatBoost | 60.286 | Rank 60 | 0.810 (0.803-0.818) | 0.653 (0.632-0.672) | 0.909 (0.901-0.916) | 0.842 (0.822-0.865) | 0.807 (0.798-0.815) | 0.560 (0.526-0.592) | 0.134 (0.130-0.137) |
| RF+NaiveBayes+CatBoost | 61.000 | Rank 61 | 0.808 (0.800-0.816) | 0.656 (0.638-0.676) | 0.915 (0.908-0.922) | 0.853 (0.831-0.874) | 0.803 (0.794-0.811) | 0.586 (0.554-0.617) | 0.134 (0.131-0.138) |
| AdaBoost+DT+RF+NaiveBayes+CatBoost | 61.286 | Rank 62 | 0.805 (0.797-0.813) | 0.680 (0.662-0.697) | 0.923 (0.916-0.930) | 0.899 (0.881-0.917) | 0.794 (0.786-0.802) | 0.595 (0.560-0.626) | 0.136 (0.132-0.139) |
| DT+RF+SVM+CatBoost | 61.286 | Rank 62 | 0.804 (0.796-0.812) | 0.681 (0.662-0.698) | 0.924 (0.917-0.931) | 0.903 (0.885-0.920) | 0.792 (0.784-0.801) | 0.594 (0.560-0.628) | 0.135 (0.132-0.139) |
| DT+LightGBM+CatBoost | 62.429 | Rank 64 | 0.818 (0.810-0.826) | 0.643 (0.622-0.663) | 0.896 (0.887-0.904) | 0.814 (0.790-0.838) | 0.818 (0.810-0.826) | 0.490 (0.455-0.521) | 0.134 (0.130-0.137) |
| RF+LightGBM+SVM+CatBoost | 63.143 | Rank 65 | 0.807 (0.799-0.814) | 0.669 (0.651-0.687) | 0.914 (0.907-0.921) | 0.878 (0.859-0.898) | 0.798 (0.790-0.806) | 0.547 (0.516-0.577) | 0.137 (0.134-0.141) |
| RF+LightGBM+NaiveBayes+CatBoost | 67.143 | Rank 66 | 0.802 (0.794-0.809) | 0.665 (0.646-0.683) | 0.916 (0.909-0.923) | 0.878 (0.858-0.898) | 0.793 (0.784-0.801) | 0.553 (0.522-0.584) | 0.137 (0.133-0.141) |
| DT+RF+NaiveBayes+CatBoost | 67.429 | Rank 67 | 0.803 (0.795-0.811) | 0.653 (0.634-0.672) | 0.910 (0.902-0.918) | 0.854 (0.833-0.876) | 0.797 (0.789-0.805) | 0.588 (0.556-0.619) | 0.137 (0.133-0.140) |
| AdaBoost+LightGBM+SVM+CatBoost | 68.429 | Rank 68 | 0.809 (0.802-0.816) | 0.644 (0.623-0.666) | 0.898 (0.890-0.906) | 0.830 (0.809-0.854) | 0.806 (0.798-0.814) | 0.494 (0.460-0.527) | 0.139 (0.136-0.143) |
| LightGBM+NaiveBayes+SVM+CatBoost | 68.429 | Rank 68 | 0.810 (0.803-0.819) | 0.641 (0.621-0.659) | 0.893 (0.885-0.902) | 0.821 (0.799-0.843) | 0.809 (0.801-0.818) | 0.489 (0.458-0.522) | 0.137 (0.133-0.140) |
| AdaBoost+DT+LightGBM+NaiveBayes+SVM+CatBoost | 69.143 | Rank 70 | 0.803 (0.795-0.811) | 0.660 (0.641-0.678) | 0.905 (0.897-0.913) | 0.867 (0.847-0.886) | 0.795 (0.787-0.804) | 0.514 (0.480-0.547) | 0.137 (0.134-0.141) |
| AdaBoost+DT+LightGBM | 69.571 | Rank 71 | 0.809 (0.801-0.816) | 0.665 (0.646-0.684) | 0.902 (0.895-0.910) | 0.866 (0.846-0.888) | 0.802 (0.794-0.810) | 0.458 (0.426-0.489) | 0.143 (0.139-0.146) |
| DT+LightGBM | 69.571 | Rank 71 | 0.805 (0.797-0.813) | 0.681 (0.663-0.699) | 0.911 (0.904-0.918) | 0.901 (0.884-0.920) | 0.793 (0.785-0.802) | 0.489 (0.458-0.520) | 0.146 (0.143-0.150) |
| DT+LightGBM+NaiveBayes | 71.429 | Rank 73 | 0.807 (0.799-0.815) | 0.667 (0.649-0.685) | 0.905 (0.898-0.912) | 0.873 (0.852-0.893) | 0.799 (0.791-0.807) | 0.459 (0.427-0.494) | 0.146 (0.143-0.149) |
| LightGBM+SVM+CatBoost | 72.714 | Rank 74 | 0.807 (0.799-0.815) | 0.628 (0.605-0.648) | 0.894 (0.886-0.903) | 0.804 (0.780-0.828) | 0.808 (0.800-0.816) | 0.501 (0.463-0.533) | 0.139 (0.136-0.143) |
| AdaBoost+DT+LightGBM+NaiveBayes | 73.000 | Rank 75 | 0.809 (0.801-0.817) | 0.655 (0.635-0.674) | 0.895 (0.887-0.903) | 0.848 (0.826-0.871) | 0.804 (0.796-0.812) | 0.454 (0.425-0.484) | 0.144 (0.140-0.147) |
| RF+CatBoost | 76.429 | Rank 76 | 0.795 (0.787-0.803) | 0.651 (0.633-0.669) | 0.909 (0.901-0.916) | 0.865 (0.844-0.885) | 0.787 (0.778-0.795) | 0.555 (0.523-0.587) | 0.141 (0.137-0.144) |
| AdaBoost+LightGBM+CatBoost | 76.857 | Rank 77 | 0.804 (0.797-0.812) | 0.634 (0.615-0.654) | 0.891 (0.883-0.900) | 0.820 (0.797-0.844) | 0.803 (0.794-0.811) | 0.482 (0.450-0.516) | 0.139 (0.135-0.142) |
| AdaBoost+LightGBM+NaiveBayes+CatBoost | 78.143 | Rank 78 | 0.801 (0.793-0.809) | 0.639 (0.618-0.658) | 0.896 (0.888-0.905) | 0.833 (0.810-0.856) | 0.797 (0.788-0.806) | 0.501 (0.470-0.533) | 0.142 (0.138-0.145) |
| AdaBoost+LightGBM | 78.714 | Rank 79 | 0.799 (0.790-0.807) | 0.661 (0.642-0.679) | 0.903 (0.895-0.910) | 0.875 (0.855-0.895) | 0.790 (0.781-0.798) | 0.475 (0.443-0.505) | 0.147 (0.143-0.150) |
| DT+LightGBM+NaiveBayes+CatBoost | 79.000 | Rank 80 | 0.801 (0.793-0.809) | 0.637 (0.616-0.655) | 0.895 (0.887-0.903) | 0.830 (0.808-0.852) | 0.797 (0.789-0.806) | 0.484 (0.450-0.518) | 0.141 (0.138-0.145) |
| LightGBM+NaiveBayes | 79.714 | Rank 81 | 0.803 (0.795-0.811) | 0.653 (0.634-0.672) | 0.897 (0.890-0.905) | 0.855 (0.834-0.876) | 0.797 (0.788-0.805) | 0.450 (0.421-0.479) | 0.146 (0.142-0.149) |
| AdaBoost+DT+NaiveBayes+CatBoost | 80.429 | Rank 82 | 0.805 (0.797-0.813) | 0.599 (0.577-0.620) | 0.874 (0.864-0.885) | 0.760 (0.734-0.786) | 0.810 (0.802-0.819) | 0.474 (0.441-0.506) | 0.140 (0.137-0.143) |
| DT+NaiveBayes+CatBoost | 81.714 | Rank 83 | 0.802 (0.794-0.810) | 0.604 (0.582-0.625) | 0.878 (0.868-0.888) | 0.772 (0.746-0.798) | 0.805 (0.797-0.814) | 0.491 (0.458-0.525) | 0.142 (0.139-0.145) |
| AdaBoost+DT+LightGBM+SVM | 83.429 | Rank 84 | 0.805 (0.797-0.812) | 0.646 (0.626-0.665) | 0.887 (0.879-0.896) | 0.840 (0.817-0.863) | 0.801 (0.793-0.809) | 0.448 (0.412-0.480) | 0.148 (0.144-0.151) |
| DT+LightGBM+NaiveBayes+SVM+CatBoost | 85.000 | Rank 85 | 0.797 (0.789-0.806) | 0.632 (0.612-0.652) | 0.890 (0.881-0.898) | 0.828 (0.806-0.852) | 0.793 (0.785-0.802) | 0.487 (0.456-0.519) | 0.143 (0.139-0.146) |
| AdaBoost+DT+LightGBM+CatBoost | 86.714 | Rank 86 | 0.801 (0.794-0.809) | 0.633 (0.615-0.653) | 0.886 (0.877-0.895) | 0.824 (0.802-0.847) | 0.798 (0.790-0.807) | 0.435 (0.403-0.465) | 0.144 (0.141-0.148) |
| AdaBoost+LightGBM+SVM | 87.000 | Rank 87 | 0.797 (0.789-0.805) | 0.638 (0.617-0.657) | 0.894 (0.887-0.902) | 0.838 (0.815-0.859) | 0.792 (0.783-0.801) | 0.473 (0.440-0.505) | 0.148 (0.144-0.151) |
| DT+LightGBM+SVM | 87.143 | Rank 88 | 0.796 (0.788-0.804) | 0.644 (0.624-0.662) | 0.891 (0.883-0.899) | 0.850 (0.827-0.872) | 0.790 (0.781-0.798) | 0.471 (0.439-0.503) | 0.148 (0.144-0.151) |
| LightGBM+NaiveBayes+SVM | 87.571 | Rank 89 | 0.791 (0.783-0.799) | 0.650 (0.629-0.667) | 0.899 (0.891-0.907) | 0.868 (0.849-0.889) | 0.782 (0.773-0.791) | 0.469 (0.436-0.503) | 0.151 (0.147-0.154) |
| AdaBoost+LightGBM+NaiveBayes | 88.000 | Rank 90 | 0.800 (0.792-0.808) | 0.648 (0.628-0.666) | 0.890 (0.882-0.898) | 0.850 (0.829-0.873) | 0.794 (0.785-0.803) | 0.427 (0.396-0.458) | 0.150 (0.146-0.153) |
| AdaBoost+CatBoost | 88.143 | Rank 91 | 0.801 (0.793-0.809) | 0.596 (0.573-0.617) | 0.872 (0.862-0.881) | 0.760 (0.734-0.786) | 0.806 (0.797-0.814) | 0.450 (0.418-0.483) | 0.143 (0.140-0.147) |
| DT+LightGBM+NaiveBayes+SVM | 89.143 | Rank 92 | 0.793 (0.784-0.801) | 0.652 (0.634-0.671) | 0.895 (0.887-0.903) | 0.870 (0.850-0.891) | 0.783 (0.775-0.792) | 0.456 (0.422-0.488) | 0.151 (0.148-0.155) |
| LightGBM+CatBoost | 89.286 | Rank 93 | 0.795 (0.787-0.804) | 0.625 (0.604-0.643) | 0.886 (0.877-0.894) | 0.818 (0.793-0.842) | 0.792 (0.784-0.801) | 0.470 (0.435-0.501) | 0.143 (0.139-0.146) |
| AdaBoost+NaiveBayes+CatBoost | 90.571 | Rank 94 | 0.798 (0.791-0.806) | 0.616 (0.597-0.636) | 0.875 (0.866-0.885) | 0.799 (0.776-0.824) | 0.798 (0.790-0.807) | 0.449 (0.419-0.480) | 0.145 (0.142-0.149) |
| AdaBoost+SVM+CatBoost | 91.571 | Rank 95 | 0.799 (0.790-0.806) | 0.593 (0.572-0.613) | 0.867 (0.858-0.877) | 0.757 (0.733-0.784) | 0.804 (0.795-0.812) | 0.462 (0.433-0.494) | 0.145 (0.142-0.149) |
| AdaBoost+DT+LightGBM+NaiveBayes+CatBoost | 91.714 | Rank 96 | 0.791 (0.783-0.800) | 0.620 (0.598-0.640) | 0.888 (0.879-0.897) | 0.816 (0.793-0.840) | 0.788 (0.779-0.797) | 0.475 (0.444-0.508) | 0.145 (0.141-0.148) |
| DT+SVM+CatBoost | 91.857 | Rank 97 | 0.799 (0.791-0.807) | 0.593 (0.572-0.614) | 0.870 (0.860-0.880) | 0.758 (0.731-0.783) | 0.804 (0.795-0.813) | 0.447 (0.413-0.480) | 0.144 (0.140-0.147) |
| SVM+CatBoost | 91.857 | Rank 97 | 0.796 (0.788-0.805) | 0.608 (0.587-0.629) | 0.878 (0.868-0.887) | 0.787 (0.763-0.812) | 0.797 (0.789-0.806) | 0.456 (0.423-0.493) | 0.146 (0.142-0.149) |
| AdaBoost+LightGBM+NaiveBayes+SVM | 92.857 | Rank 99 | 0.792 (0.783-0.800) | 0.635 (0.615-0.655) | 0.892 (0.884-0.900) | 0.842 (0.820-0.865) | 0.786 (0.777-0.794) | 0.456 (0.425-0.489) | 0.149 (0.146-0.152) |
| NaiveBayes+CatBoost | 93.571 | Rank 100 | 0.795 (0.788-0.803) | 0.589 (0.568-0.610) | 0.868 (0.858-0.878) | 0.757 (0.732-0.783) | 0.800 (0.792-0.808) | 0.461 (0.429-0.494) | 0.143 (0.140-0.147) |
| LightGBM+SVM | 94.000 | Rank 101 | 0.784 (0.777-0.792) | 0.633 (0.615-0.653) | 0.891 (0.883-0.900) | 0.850 (0.829-0.872) | 0.776 (0.768-0.785) | 0.473 (0.440-0.506) | 0.153 (0.150-0.157) |
| AdaBoost+DT+SVM+CatBoost | 94.286 | Rank 102 | 0.797 (0.789-0.805) | 0.594 (0.572-0.615) | 0.867 (0.856-0.877) | 0.762 (0.737-0.788) | 0.801 (0.793-0.810) | 0.453 (0.421-0.484) | 0.146 (0.143-0.149) |
| AdaBoost+NaiveBayes+SVM+CatBoost | 95.000 | Rank 103 | 0.793 (0.785-0.801) | 0.593 (0.572-0.612) | 0.875 (0.865-0.885) | 0.765 (0.739-0.792) | 0.796 (0.788-0.804) | 0.475 (0.443-0.507) | 0.146 (0.143-0.150) |
| NaiveBayes+SVM+CatBoost | 95.000 | Rank 103 | 0.793 (0.785-0.801) | 0.597 (0.574-0.618) | 0.870 (0.859-0.880) | 0.773 (0.747-0.799) | 0.795 (0.787-0.804) | 0.469 (0.434-0.503) | 0.146 (0.143-0.149) |
| AdaBoost+DT+NaiveBayes+SVM+CatBoost | 95.429 | Rank 105 | 0.793 (0.785-0.801) | 0.601 (0.580-0.622) | 0.871 (0.861-0.881) | 0.779 (0.755-0.804) | 0.795 (0.787-0.803) | 0.461 (0.429-0.493) | 0.147 (0.143-0.150) |
| AdaBoost+DT+LightGBM+NaiveBayes+SVM | 99.714 | Rank 106 | 0.788 (0.780-0.796) | 0.618 (0.598-0.637) | 0.881 (0.872-0.890) | 0.817 (0.793-0.841) | 0.784 (0.776-0.792) | 0.436 (0.405-0.466) | 0.148 (0.145-0.151) |
| DT+CatBoost | 100.286 | Rank 107 | 0.787 (0.778-0.794) | 0.599 (0.577-0.618) | 0.872 (0.862-0.881) | 0.785 (0.759-0.809) | 0.787 (0.778-0.795) | 0.452 (0.420-0.485) | 0.146 (0.143-0.150) |
| DT+NaiveBayes+SVM+CatBoost | 102.000 | Rank 108 | 0.789 (0.781-0.797) | 0.584 (0.563-0.605) | 0.865 (0.855-0.876) | 0.756 (0.731-0.781) | 0.793 (0.784-0.802) | 0.450 (0.418-0.482) | 0.146 (0.142-0.149) |
| AdaBoost+DT+CatBoost | 106.571 | Rank 109 | 0.784 (0.776-0.792) | 0.592 (0.570-0.611) | 0.862 (0.852-0.873) | 0.777 (0.753-0.800) | 0.785 (0.777-0.794) | 0.413 (0.382-0.446) | 0.152 (0.149-0.156) |
| NaiveBayes+SVM | 114.429 | Rank 110 | 0.663 (0.654-0.673) | 0.478 (0.457-0.500) | 0.751 (0.735-0.765) | 0.726 (0.698-0.753) | 0.656 (0.646-0.665) | 0.236 (0.217-0.256) | 0.212 (0.208-0.216) |
| AdaBoost+NaiveBayes+SVM | 115.286 | Rank 111 | 0.719 (0.710-0.728) | 0.436 (0.415-0.458) | 0.724 (0.709-0.739) | 0.594 (0.565-0.622) | 0.734 (0.724-0.743) | 0.231 (0.211-0.253) | 0.182 (0.179-0.186) |
| AdaBoost+SVM | 115.286 | Rank 111 | 0.683 (0.674-0.693) | 0.448 (0.427-0.470) | 0.733 (0.719-0.748) | 0.650 (0.621-0.679) | 0.687 (0.678-0.697) | 0.236 (0.215-0.258) | 0.202 (0.199-0.206) |
| DT+NaiveBayes+SVM | 115.429 | Rank 113 | 0.651 (0.641-0.660) | 0.455 (0.434-0.476) | 0.741 (0.725-0.756) | 0.696 (0.669-0.723) | 0.645 (0.635-0.655) | 0.237 (0.217-0.258) | 0.211 (0.208-0.215) |
| DT+SVM | 115.429 | Rank 113 | 0.656 (0.646-0.665) | 0.480 (0.461-0.501) | 0.738 (0.724-0.752) | 0.738 (0.712-0.765) | 0.646 (0.636-0.656) | 0.233 (0.211-0.254) | 0.213 (0.209-0.216) |
| AdaBoost+DT+NaiveBayes+SVM | 115.571 | Rank 115 | 0.712 (0.704-0.721) | 0.450 (0.429-0.473) | 0.732 (0.717-0.748) | 0.624 (0.594-0.654) | 0.723 (0.713-0.732) | 0.231 (0.211-0.250) | 0.190 (0.186-0.193) |
| AdaBoost+DT+SVM | 115.571 | Rank 115 | 0.676 (0.667-0.686) | 0.453 (0.430-0.474) | 0.734 (0.719-0.749) | 0.665 (0.638-0.695) | 0.677 (0.668-0.688) | 0.233 (0.212-0.256) | 0.206 (0.203-0.210) |
| AdaBoost+DT+NaiveBayes | 117.571 | Rank 117 | 0.680 (0.671-0.689) | 0.435 (0.413-0.457) | 0.709 (0.694-0.726) | 0.630 (0.603-0.662) | 0.686 (0.676-0.695) | 0.224 (0.202-0.244) | 0.211 (0.208-0.215) |
| LightGBM | 118.000 | Rank 118 | 0.729 (0.715-0.742) | 0.383 (0.359-0.406) | 0.671 (0.650-0.692) | 0.501 (0.469-0.534) | 0.756 (0.738-0.774) | 0.211 (0.180-0.242) | 0.185 (0.178-0.192) |
| RF | 119.143 | Rank 119 | 0.716 (0.702-0.730) | 0.372 (0.338-0.406) | 0.686 (0.666-0.705) | 0.499 (0.438-0.561) | 0.743 (0.721-0.765) | 0.201 (0.183-0.218) | 0.186 (0.182-0.190) |
| CatBoost | 119.429 | Rank 120 | 0.728 (0.715-0.741) | 0.352 (0.316-0.388) | 0.670 (0.642-0.698) | 0.459 (0.393-0.526) | 0.761 (0.744-0.779) | 0.196 (0.176-0.216) | 0.181 (0.175-0.186) |
| AdaBoost+DT | 119.714 | Rank 121 | 0.631 (0.622-0.640) | 0.439 (0.417-0.460) | 0.701 (0.685-0.717) | 0.685 (0.655-0.713) | 0.625 (0.615-0.635) | 0.211 (0.192-0.230) | 0.222 (0.219-0.225) |
| NaiveBayes | 120.857 | Rank 122 | 0.402 (0.363-0.441) | 0.399 (0.381-0.417) | 0.661 (0.635-0.687) | 0.816 (0.776-0.856) | 0.352 (0.303-0.401) | 0.205 (0.182-0.227) | 0.354 (0.331-0.377) |
| DT+NaiveBayes | 121.714 | Rank 123 | 0.581 (0.571-0.591) | 0.421 (0.401-0.440) | 0.684 (0.668-0.699) | 0.700 (0.673-0.727) | 0.566 (0.556-0.577) | 0.213 (0.192-0.234) | 0.224 (0.221-0.227) |
| AdaBoost+NaiveBayes | 122.143 | Rank 124 | 0.609 (0.600-0.618) | 0.422 (0.400-0.442) | 0.685 (0.669-0.702) | 0.674 (0.645-0.702) | 0.602 (0.591-0.611) | 0.204 (0.183-0.225) | 0.234 (0.231-0.237) |
| AdaBoost | 122.571 | Rank 125 | 0.635 (0.608-0.663) | 0.400 (0.379-0.422) | 0.680 (0.655-0.706) | 0.619 (0.543-0.695) | 0.639 (0.599-0.678) | 0.202 (0.180-0.224) | 0.223 (0.218-0.229) |
| SVM | 123.286 | Rank 126 | 0.638 (0.615-0.661) | 0.377 (0.358-0.396) | 0.660 (0.639-0.681) | 0.570 (0.531-0.609) | 0.647 (0.620-0.674) | 0.197 (0.165-0.228) | 0.219 (0.212-0.227) |
| XGBoost | 124.143 | Rank 127 | 0.697 (0.686-0.709) | 0.334 (0.300-0.368) | 0.634 (0.603-0.666) | 0.455 (0.395-0.514) | 0.727 (0.709-0.746) | 0.171 (0.155-0.186) | 0.241 (0.229-0.252) |
| DT | 124.571 | Rank 128 | 0.582 (0.541-0.623) | 0.404 (0.383-0.426) | 0.643 (0.618-0.668) | 0.674 (0.601-0.746) | 0.572 (0.519-0.625) | 0.162 (0.146-0.178) | 0.233 (0.227-0.240) |
| KNN | 127.000 | Rank 129 | 0.614 (0.595-0.633) | 0.344 (0.311-0.378) | 0.609 (0.582-0.636) | 0.528 (0.487-0.569) | 0.625 (0.605-0.644) | 0.159 (0.137-0.181) | 0.269 (0.257-0.281) |

**Supplementary Table 30.** Ranking of 129 machine learning models when the SHARE cohort was used as the validation dataset.

| **Model** | **Normalized Rank Score** | **Comprehensive Rank** | **Accuracy (95% CI)** | **F_Beta (95% CI)** | **ROC_AUC (95% CI)** | **Sensitivity (95% CI)** | **Specificity (95% CI)** | **PR_AUC (95% CI)** | **Brier (95% CI)** |
| --- | --- | --- | --- | --- | --- | --- | --- | --- | --- |
| AdaBoost+DT+RF+LightGBM+NaiveBayes+CatBoost | 18.143 | Rank 1 | 0.822 (0.807-0.837) | 0.221 (0.175-0.265) | 0.631 (0.599-0.664) | 0.224 (0.178-0.272) | 0.896 (0.885-0.909) | 0.183 (0.149-0.219) | 0.137 (0.130-0.144) |
| NaiveBayes+CatBoost | 22.286 | Rank 2 | 0.818 (0.803-0.832) | 0.223 (0.178-0.266) | 0.633 (0.596-0.667) | 0.228 (0.180-0.274) | 0.892 (0.879-0.904) | 0.177 (0.143-0.210) | 0.141 (0.134-0.147) |
| AdaBoost+RF+LightGBM+SVM+CatBoost | 27.571 | Rank 3 | 0.815 (0.801-0.829) | 0.208 (0.164-0.252) | 0.630 (0.597-0.665) | 0.212 (0.165-0.259) | 0.890 (0.877-0.903) | 0.176 (0.145-0.206) | 0.135 (0.128-0.143) |
| AdaBoost+LightGBM+SVM+CatBoost | 29.571 | Rank 4 | 0.822 (0.808-0.836) | 0.216 (0.170-0.259) | 0.618 (0.582-0.653) | 0.218 (0.171-0.265) | 0.897 (0.885-0.909) | 0.169 (0.139-0.199) | 0.139 (0.132-0.147) |
| RF+SVM+CatBoost | 29.857 | Rank 5 | 0.816 (0.801-0.830) | 0.213 (0.167-0.253) | 0.633 (0.602-0.664) | 0.217 (0.168-0.261) | 0.891 (0.878-0.903) | 0.165 (0.139-0.193) | 0.140 (0.132-0.147) |
| DT+NaiveBayes+SVM+CatBoost | 31.571 | Rank 6 | 0.815 (0.800-0.829) | 0.207 (0.164-0.249) | 0.630 (0.595-0.665) | 0.211 (0.167-0.257) | 0.890 (0.878-0.904) | 0.172 (0.141-0.205) | 0.140 (0.132-0.147) |
| AdaBoost+DT+SVM+CatBoost | 33.857 | Rank 7 | 0.808 (0.793-0.823) | 0.241 (0.195-0.287) | 0.628 (0.595-0.661) | 0.253 (0.204-0.304) | 0.877 (0.864-0.890) | 0.164 (0.138-0.190) | 0.145 (0.137-0.153) |
| DT+NaiveBayes+CatBoost | 34.857 | Rank 8 | 0.817 (0.803-0.833) | 0.229 (0.183-0.274) | 0.615 (0.583-0.648) | 0.235 (0.186-0.284) | 0.889 (0.876-0.903) | 0.167 (0.139-0.198) | 0.143 (0.135-0.151) |
| DT+RF+SVM | 38.286 | Rank 9 | 0.814 (0.799-0.830) | 0.208 (0.161-0.254) | 0.618 (0.585-0.649) | 0.212 (0.165-0.262) | 0.889 (0.876-0.902) | 0.169 (0.139-0.202) | 0.141 (0.133-0.147) |
| AdaBoost+DT+RF+SVM+CatBoost | 38.714 | Rank 10 | 0.805 (0.790-0.821) | 0.210 (0.168-0.251) | 0.625 (0.594-0.657) | 0.218 (0.173-0.263) | 0.878 (0.866-0.892) | 0.169 (0.142-0.199) | 0.140 (0.132-0.147) |
| AdaBoost+DT+CatBoost | 39.143 | Rank 11 | 0.823 (0.809-0.837) | 0.207 (0.158-0.250) | 0.612 (0.580-0.644) | 0.208 (0.157-0.253) | 0.899 (0.888-0.911) | 0.163 (0.137-0.190) | 0.138 (0.130-0.145) |
| AdaBoost+RF | 39.714 | Rank 12 | 0.803 (0.788-0.818) | 0.240 (0.193-0.282) | 0.628 (0.596-0.662) | 0.254 (0.204-0.304) | 0.871 (0.858-0.885) | 0.165 (0.139-0.195) | 0.147 (0.139-0.155) |
| DT+RF+LightGBM+NaiveBayes+SVM | 39.857 | Rank 13 | 0.807 (0.793-0.822) | 0.230 (0.182-0.275) | 0.616 (0.583-0.652) | 0.241 (0.189-0.291) | 0.877 (0.865-0.891) | 0.171 (0.139-0.204) | 0.145 (0.138-0.152) |
| DT+LightGBM+CatBoost | 40.429 | Rank 14 | 0.833 (0.819-0.847) | 0.204 (0.156-0.248) | 0.600 (0.565-0.635) | 0.200 (0.150-0.246) | 0.912 (0.901-0.924) | 0.172 (0.140-0.207) | 0.134 (0.127-0.142) |
| AdaBoost+DT+LightGBM+NaiveBayes+SVM | 40.857 | Rank 15 | 0.802 (0.787-0.818) | 0.237 (0.191-0.283) | 0.621 (0.587-0.655) | 0.251 (0.202-0.300) | 0.871 (0.858-0.884) | 0.168 (0.138-0.200) | 0.145 (0.138-0.152) |
| RF+LightGBM+CatBoost | 42.143 | Rank 16 | 0.804 (0.789-0.819) | 0.225 (0.177-0.267) | 0.626 (0.591-0.658) | 0.235 (0.186-0.282) | 0.875 (0.861-0.889) | 0.160 (0.134-0.186) | 0.142 (0.134-0.149) |
| DT+SVM+CatBoost | 43.429 | Rank 17 | 0.822 (0.808-0.836) | 0.195 (0.150-0.239) | 0.599 (0.567-0.637) | 0.195 (0.149-0.241) | 0.901 (0.889-0.913) | 0.181 (0.143-0.219) | 0.136 (0.129-0.144) |
| RF+CatBoost | 44.571 | Rank 18 | 0.796 (0.780-0.811) | 0.216 (0.172-0.261) | 0.623 (0.590-0.654) | 0.229 (0.180-0.279) | 0.867 (0.853-0.880) | 0.181 (0.147-0.216) | 0.141 (0.134-0.149) |
| AdaBoost+RF+NaiveBayes+CatBoost | 44.714 | Rank 19 | 0.806 (0.790-0.821) | 0.209 (0.166-0.252) | 0.618 (0.585-0.652) | 0.217 (0.170-0.263) | 0.879 (0.866-0.892) | 0.167 (0.136-0.198) | 0.140 (0.132-0.148) |
| RF+LightGBM+NaiveBayes | 45.143 | Rank 20 | 0.799 (0.784-0.815) | 0.243 (0.197-0.286) | 0.619 (0.585-0.652) | 0.259 (0.208-0.308) | 0.866 (0.854-0.879) | 0.167 (0.139-0.197) | 0.147 (0.140-0.154) |
| AdaBoost+DT+LightGBM+CatBoost | 45.429 | Rank 21 | 0.812 (0.797-0.827) | 0.206 (0.160-0.250) | 0.604 (0.570-0.641) | 0.211 (0.163-0.262) | 0.887 (0.875-0.901) | 0.182 (0.145-0.219) | 0.138 (0.131-0.146) |
| DT+LightGBM+NaiveBayes+SVM+CatBoost | 46.143 | Rank 22 | 0.821 (0.807-0.836) | 0.161 (0.119-0.202) | 0.623 (0.587-0.659) | 0.159 (0.118-0.202) | 0.904 (0.892-0.917) | 0.166 (0.135-0.196) | 0.135 (0.128-0.143) |
| AdaBoost+DT+NaiveBayes+SVM+CatBoost | 47.429 | Rank 23 | 0.812 (0.798-0.827) | 0.191 (0.149-0.232) | 0.614 (0.581-0.648) | 0.195 (0.151-0.239) | 0.890 (0.877-0.903) | 0.177 (0.143-0.212) | 0.141 (0.134-0.149) |
| AdaBoost+DT+LightGBM | 48.571 | Rank 24 | 0.777 (0.762-0.793) | 0.254 (0.210-0.295) | 0.636 (0.604-0.668) | 0.284 (0.233-0.330) | 0.839 (0.824-0.854) | 0.183 (0.150-0.218) | 0.154 (0.147-0.161) |
| AdaBoost+SVM+CatBoost | 49.429 | Rank 25 | 0.796 (0.780-0.812) | 0.217 (0.171-0.260) | 0.623 (0.590-0.655) | 0.230 (0.181-0.277) | 0.866 (0.853-0.880) | 0.172 (0.138-0.203) | 0.147 (0.139-0.154) |
| AdaBoost+RF+LightGBM+CatBoost | 50.000 | Rank 26 | 0.822 (0.807-0.836) | 0.157 (0.115-0.196) | 0.614 (0.581-0.647) | 0.154 (0.112-0.196) | 0.905 (0.893-0.917) | 0.168 (0.138-0.196) | 0.131 (0.123-0.139) |
| AdaBoost+RF+SVM+CatBoost | 50.286 | Rank 27 | 0.800 (0.784-0.816) | 0.213 (0.168-0.257) | 0.618 (0.583-0.652) | 0.224 (0.176-0.272) | 0.872 (0.858-0.887) | 0.168 (0.137-0.200) | 0.144 (0.137-0.152) |
| AdaBoost+DT+NaiveBayes+SVM | 50.714 | Rank 28 | 0.789 (0.773-0.806) | 0.240 (0.195-0.281) | 0.634 (0.602-0.666) | 0.260 (0.210-0.308) | 0.856 (0.842-0.871) | 0.169 (0.137-0.201) | 0.160 (0.153-0.166) |
| AdaBoost+DT+LightGBM+SVM+CatBoost | 50.857 | Rank 29 | 0.825 (0.810-0.840) | 0.179 (0.136-0.220) | 0.613 (0.578-0.647) | 0.177 (0.134-0.218) | 0.906 (0.894-0.919) | 0.160 (0.132-0.188) | 0.133 (0.126-0.141) |
| DT+LightGBM+NaiveBayes+CatBoost | 51.000 | Rank 30 | 0.813 (0.799-0.829) | 0.191 (0.147-0.235) | 0.622 (0.586-0.658) | 0.194 (0.147-0.240) | 0.891 (0.878-0.903) | 0.157 (0.131-0.184) | 0.138 (0.131-0.146) |
| AdaBoost+DT+RF+LightGBM+SVM+CatBoost | 51.429 | Rank 31 | 0.810 (0.795-0.825) | 0.186 (0.143-0.226) | 0.616 (0.583-0.649) | 0.189 (0.144-0.232) | 0.888 (0.875-0.900) | 0.166 (0.138-0.196) | 0.138 (0.131-0.145) |
| AdaBoost+RF+SVM | 51.714 | Rank 32 | 0.800 (0.785-0.815) | 0.194 (0.151-0.237) | 0.628 (0.596-0.661) | 0.202 (0.158-0.250) | 0.875 (0.861-0.888) | 0.172 (0.141-0.204) | 0.143 (0.136-0.150) |
| DT+RF+LightGBM+SVM+CatBoost | 52.286 | Rank 33 | 0.802 (0.787-0.817) | 0.214 (0.169-0.257) | 0.621 (0.589-0.654) | 0.225 (0.176-0.271) | 0.874 (0.861-0.888) | 0.160 (0.134-0.189) | 0.144 (0.136-0.151) |
| LightGBM+NaiveBayes+SVM+CatBoost | 52.857 | Rank 34 | 0.808 (0.793-0.822) | 0.210 (0.166-0.252) | 0.610 (0.579-0.641) | 0.217 (0.170-0.262) | 0.881 (0.868-0.894) | 0.163 (0.134-0.191) | 0.143 (0.137-0.151) |
| AdaBoost+RF+LightGBM+NaiveBayes+SVM+CatBoost | 53.143 | Rank 35 | 0.799 (0.783-0.814) | 0.207 (0.164-0.249) | 0.623 (0.590-0.658) | 0.217 (0.173-0.265) | 0.871 (0.857-0.885) | 0.167 (0.137-0.198) | 0.146 (0.138-0.153) |
| AdaBoost+DT+NaiveBayes | 53.571 | Rank 36 | 0.756 (0.741-0.772) | 0.310 (0.267-0.352) | 0.645 (0.613-0.676) | 0.369 (0.317-0.424) | 0.804 (0.790-0.820) | 0.184 (0.152-0.216) | 0.172 (0.166-0.178) |
| RF+LightGBM+NaiveBayes+SVM+CatBoost | 53.714 | Rank 37 | 0.793 (0.779-0.809) | 0.220 (0.170-0.263) | 0.615 (0.582-0.646) | 0.235 (0.181-0.284) | 0.863 (0.849-0.876) | 0.172 (0.140-0.208) | 0.146 (0.138-0.154) |
| AdaBoost+NaiveBayes+CatBoost | 53.857 | Rank 38 | 0.805 (0.789-0.820) | 0.214 (0.166-0.257) | 0.611 (0.577-0.646) | 0.223 (0.173-0.272) | 0.877 (0.864-0.891) | 0.163 (0.136-0.194) | 0.146 (0.139-0.153) |
| AdaBoost+DT+NaiveBayes+CatBoost | 54.143 | Rank 39 | 0.813 (0.797-0.828) | 0.170 (0.129-0.211) | 0.612 (0.579-0.647) | 0.171 (0.129-0.213) | 0.893 (0.880-0.905) | 0.174 (0.143-0.206) | 0.141 (0.133-0.149) |
| LightGBM+NaiveBayes+CatBoost | 55.286 | Rank 40 | 0.817 (0.802-0.833) | 0.188 (0.145-0.231) | 0.597 (0.564-0.634) | 0.189 (0.145-0.236) | 0.895 (0.884-0.908) | 0.162 (0.134-0.194) | 0.137 (0.129-0.144) |
| DT+RF+CatBoost | 55.429 | Rank 41 | 0.811 (0.797-0.827) | 0.185 (0.141-0.227) | 0.613 (0.581-0.646) | 0.188 (0.143-0.234) | 0.889 (0.877-0.902) | 0.162 (0.135-0.193) | 0.138 (0.130-0.145) |
| RF+NaiveBayes+CatBoost | 56.286 | Rank 42 | 0.792 (0.777-0.808) | 0.221 (0.177-0.265) | 0.623 (0.589-0.656) | 0.236 (0.188-0.286) | 0.861 (0.848-0.875) | 0.160 (0.134-0.184) | 0.145 (0.137-0.153) |
| AdaBoost+DT+RF+NaiveBayes+SVM+CatBoost | 56.429 | Rank 43 | 0.814 (0.799-0.828) | 0.207 (0.160-0.251) | 0.607 (0.572-0.640) | 0.212 (0.163-0.257) | 0.889 (0.876-0.901) | 0.156 (0.130-0.184) | 0.141 (0.133-0.148) |
| RF | 56.857 | Rank 44 | 0.811 (0.796-0.826) | 0.179 (0.132-0.222) | 0.622 (0.588-0.655) | 0.182 (0.133-0.225) | 0.890 (0.878-0.902) | 0.174 (0.139-0.208) | 0.152 (0.147-0.158) |
| AdaBoost+DT+RF+SVM | 57.000 | Rank 45 | 0.812 (0.797-0.826) | 0.191 (0.146-0.232) | 0.611 (0.576-0.644) | 0.195 (0.149-0.240) | 0.888 (0.876-0.902) | 0.162 (0.132-0.192) | 0.143 (0.135-0.150) |
| DT+RF+LightGBM | 58.143 | Rank 46 | 0.814 (0.799-0.827) | 0.169 (0.124-0.210) | 0.609 (0.575-0.641) | 0.170 (0.125-0.212) | 0.894 (0.881-0.906) | 0.162 (0.134-0.193) | 0.137 (0.130-0.144) |
| RF+LightGBM | 58.571 | Rank 47 | 0.810 (0.794-0.826) | 0.185 (0.142-0.227) | 0.620 (0.588-0.653) | 0.188 (0.143-0.235) | 0.887 (0.875-0.899) | 0.158 (0.131-0.184) | 0.139 (0.132-0.147) |
| RF+LightGBM+NaiveBayes+CatBoost | 59.286 | Rank 48 | 0.820 (0.805-0.834) | 0.178 (0.137-0.217) | 0.609 (0.576-0.641) | 0.177 (0.136-0.220) | 0.900 (0.887-0.912) | 0.158 (0.132-0.187) | 0.140 (0.131-0.148) |
| AdaBoost+DT+RF+LightGBM+NaiveBayes+SVM+CatBoost | 60.429 | Rank 49 | 0.810 (0.796-0.826) | 0.200 (0.153-0.242) | 0.601 (0.567-0.636) | 0.205 (0.156-0.250) | 0.885 (0.874-0.898) | 0.160 (0.131-0.191) | 0.141 (0.134-0.149) |
| DT+RF+LightGBM+CatBoost | 60.571 | Rank 50 | 0.807 (0.792-0.822) | 0.184 (0.140-0.225) | 0.620 (0.588-0.651) | 0.189 (0.144-0.232) | 0.884 (0.871-0.898) | 0.157 (0.132-0.183) | 0.138 (0.130-0.146) |
| RF+LightGBM+SVM | 60.571 | Rank 50 | 0.769 (0.754-0.786) | 0.250 (0.207-0.293) | 0.630 (0.599-0.664) | 0.283 (0.233-0.333) | 0.830 (0.815-0.845) | 0.161 (0.137-0.185) | 0.158 (0.150-0.165) |
| RF+SVM | 61.000 | Rank 52 | 0.811 (0.795-0.826) | 0.196 (0.151-0.238) | 0.614 (0.580-0.645) | 0.200 (0.154-0.245) | 0.887 (0.874-0.900) | 0.157 (0.130-0.185) | 0.145 (0.138-0.152) |
| AdaBoost+LightGBM+SVM | 61.286 | Rank 53 | 0.803 (0.787-0.818) | 0.230 (0.183-0.273) | 0.606 (0.575-0.639) | 0.242 (0.192-0.292) | 0.872 (0.858-0.887) | 0.157 (0.130-0.185) | 0.147 (0.140-0.155) |
| LightGBM+SVM+CatBoost | 61.429 | Rank 54 | 0.790 (0.773-0.805) | 0.230 (0.184-0.271) | 0.622 (0.591-0.656) | 0.248 (0.196-0.294) | 0.857 (0.843-0.872) | 0.157 (0.133-0.182) | 0.149 (0.142-0.156) |
| AdaBoost | 61.571 | Rank 55 | 0.765 (0.749-0.780) | 0.276 (0.230-0.320) | 0.629 (0.595-0.662) | 0.319 (0.263-0.374) | 0.820 (0.805-0.835) | 0.161 (0.136-0.187) | 0.169 (0.163-0.176) |
| AdaBoost+RF+CatBoost | 61.857 | Rank 56 | 0.809 (0.794-0.824) | 0.152 (0.107-0.193) | 0.611 (0.578-0.643) | 0.153 (0.109-0.196) | 0.891 (0.879-0.904) | 0.163 (0.132-0.196) | 0.139 (0.132-0.147) |
| DT+CatBoost | 62.571 | Rank 57 | 0.808 (0.793-0.823) | 0.185 (0.139-0.226) | 0.610 (0.578-0.643) | 0.189 (0.140-0.233) | 0.886 (0.873-0.898) | 0.163 (0.131-0.193) | 0.142 (0.134-0.150) |
| AdaBoost+DT+RF+LightGBM | 63.286 | Rank 58 | 0.807 (0.792-0.822) | 0.189 (0.144-0.235) | 0.607 (0.573-0.641) | 0.194 (0.147-0.243) | 0.883 (0.871-0.896) | 0.166 (0.135-0.196) | 0.143 (0.136-0.151) |
| AdaBoost+SVM | 63.571 | Rank 59 | 0.739 (0.722-0.756) | 0.281 (0.238-0.324) | 0.631 (0.601-0.663) | 0.340 (0.288-0.393) | 0.789 (0.772-0.805) | 0.161 (0.135-0.187) | 0.172 (0.166-0.178) |
| LightGBM+NaiveBayes+SVM | 64.000 | Rank 60 | 0.800 (0.785-0.816) | 0.193 (0.151-0.233) | 0.621 (0.591-0.655) | 0.201 (0.157-0.245) | 0.875 (0.863-0.889) | 0.158 (0.132-0.184) | 0.144 (0.137-0.152) |
| NaiveBayes+SVM+CatBoost | 64.000 | Rank 60 | 0.810 (0.796-0.825) | 0.167 (0.124-0.207) | 0.609 (0.576-0.643) | 0.169 (0.126-0.212) | 0.890 (0.878-0.903) | 0.162 (0.135-0.194) | 0.141 (0.134-0.147) |
| AdaBoost+DT+RF+LightGBM+SVM | 64.714 | Rank 62 | 0.815 (0.800-0.831) | 0.165 (0.119-0.208) | 0.615 (0.582-0.649) | 0.165 (0.119-0.208) | 0.896 (0.885-0.908) | 0.155 (0.130-0.182) | 0.140 (0.133-0.147) |
| DT+RF+NaiveBayes+SVM | 65.429 | Rank 63 | 0.803 (0.788-0.818) | 0.166 (0.124-0.203) | 0.620 (0.589-0.651) | 0.170 (0.128-0.210) | 0.882 (0.869-0.895) | 0.162 (0.133-0.190) | 0.141 (0.135-0.148) |
| AdaBoost+CatBoost | 65.571 | Rank 64 | 0.794 (0.780-0.809) | 0.212 (0.165-0.252) | 0.619 (0.585-0.651) | 0.225 (0.176-0.269) | 0.866 (0.852-0.879) | 0.158 (0.131-0.182) | 0.149 (0.141-0.156) |
| AdaBoost+DT+LightGBM+NaiveBayes | 65.571 | Rank 64 | 0.774 (0.758-0.790) | 0.229 (0.184-0.271) | 0.623 (0.589-0.657) | 0.255 (0.202-0.304) | 0.839 (0.825-0.853) | 0.160 (0.132-0.191) | 0.157 (0.149-0.164) |
| NaiveBayes | 66.000 | Rank 66 | 0.543 (0.524-0.561) | 0.368 (0.329-0.404) | 0.611 (0.579-0.646) | 0.616 (0.561-0.673) | 0.533 (0.514-0.553) | 0.186 (0.150-0.226) | 0.277 (0.269-0.285) |
| DT+RF+LightGBM+NaiveBayes+CatBoost | 66.429 | Rank 67 | 0.795 (0.780-0.811) | 0.226 (0.180-0.271) | 0.593 (0.559-0.630) | 0.242 (0.191-0.292) | 0.864 (0.850-0.877) | 0.164 (0.132-0.196) | 0.152 (0.144-0.159) |
| AdaBoost+DT+RF+LightGBM+CatBoost | 66.714 | Rank 68 | 0.792 (0.776-0.808) | 0.189 (0.145-0.229) | 0.629 (0.596-0.661) | 0.200 (0.152-0.243) | 0.866 (0.853-0.880) | 0.158 (0.133-0.185) | 0.142 (0.135-0.150) |
| AdaBoost+LightGBM+NaiveBayes+CatBoost | 67.000 | Rank 69 | 0.807 (0.792-0.823) | 0.158 (0.118-0.194) | 0.616 (0.580-0.651) | 0.160 (0.119-0.198) | 0.888 (0.875-0.901) | 0.160 (0.133-0.187) | 0.141 (0.132-0.148) |
| AdaBoost+LightGBM+NaiveBayes+SVM | 67.286 | Rank 70 | 0.778 (0.762-0.794) | 0.234 (0.188-0.280) | 0.629 (0.599-0.662) | 0.258 (0.208-0.314) | 0.842 (0.829-0.857) | 0.155 (0.131-0.179) | 0.157 (0.149-0.165) |
| AdaBoost+DT+SVM | 68.429 | Rank 71 | 0.732 (0.716-0.749) | 0.284 (0.237-0.328) | 0.626 (0.591-0.659) | 0.348 (0.291-0.404) | 0.780 (0.763-0.796) | 0.158 (0.133-0.185) | 0.180 (0.174-0.186) |
| AdaBoost+NaiveBayes+SVM | 68.429 | Rank 71 | 0.777 (0.761-0.794) | 0.252 (0.206-0.296) | 0.619 (0.585-0.651) | 0.282 (0.229-0.333) | 0.839 (0.825-0.854) | 0.157 (0.132-0.183) | 0.161 (0.154-0.167) |
| AdaBoost+DT+RF | 68.857 | Rank 73 | 0.795 (0.781-0.809) | 0.192 (0.150-0.231) | 0.624 (0.592-0.656) | 0.201 (0.155-0.245) | 0.870 (0.856-0.884) | 0.160 (0.132-0.186) | 0.149 (0.143-0.156) |
| AdaBoost+RF+LightGBM+NaiveBayes | 69.143 | Rank 74 | 0.811 (0.797-0.826) | 0.208 (0.163-0.250) | 0.583 (0.550-0.617) | 0.213 (0.165-0.260) | 0.886 (0.873-0.899) | 0.153 (0.128-0.179) | 0.146 (0.138-0.153) |
| CatBoost | 69.143 | Rank 74 | 0.798 (0.783-0.813) | 0.196 (0.150-0.234) | 0.604 (0.572-0.638) | 0.205 (0.157-0.248) | 0.872 (0.859-0.886) | 0.172 (0.137-0.203) | 0.150 (0.142-0.157) |
| AdaBoost+LightGBM+NaiveBayes | 69.429 | Rank 76 | 0.792 (0.776-0.807) | 0.203 (0.159-0.244) | 0.632 (0.602-0.663) | 0.216 (0.170-0.265) | 0.863 (0.849-0.877) | 0.155 (0.132-0.178) | 0.150 (0.142-0.157) |
| DT+RF+NaiveBayes+SVM+CatBoost | 69.857 | Rank 77 | 0.803 (0.788-0.818) | 0.178 (0.135-0.218) | 0.615 (0.585-0.648) | 0.183 (0.136-0.225) | 0.880 (0.867-0.893) | 0.156 (0.131-0.183) | 0.140 (0.132-0.147) |
| AdaBoost+RF+NaiveBayes+SVM | 70.143 | Rank 78 | 0.789 (0.774-0.804) | 0.235 (0.190-0.277) | 0.609 (0.575-0.643) | 0.254 (0.206-0.303) | 0.856 (0.843-0.870) | 0.154 (0.130-0.180) | 0.150 (0.143-0.157) |
| RF+NaiveBayes+SVM+CatBoost | 71.143 | Rank 79 | 0.807 (0.792-0.822) | 0.173 (0.131-0.213) | 0.617 (0.584-0.651) | 0.176 (0.133-0.220) | 0.886 (0.873-0.899) | 0.149 (0.126-0.171) | 0.140 (0.132-0.147) |
| DT+LightGBM+NaiveBayes | 71.571 | Rank 80 | 0.804 (0.790-0.818) | 0.193 (0.148-0.234) | 0.601 (0.569-0.635) | 0.200 (0.151-0.244) | 0.879 (0.867-0.892) | 0.159 (0.132-0.188) | 0.146 (0.140-0.153) |
| DT+NaiveBayes+SVM | 71.571 | Rank 80 | 0.765 (0.748-0.780) | 0.258 (0.212-0.300) | 0.612 (0.578-0.645) | 0.295 (0.243-0.346) | 0.823 (0.808-0.838) | 0.159 (0.132-0.190) | 0.166 (0.160-0.172) |
| LightGBM+CatBoost | 71.857 | Rank 82 | 0.800 (0.785-0.815) | 0.187 (0.142-0.226) | 0.616 (0.586-0.648) | 0.194 (0.147-0.238) | 0.875 (0.862-0.888) | 0.157 (0.132-0.183) | 0.144 (0.136-0.152) |
| AdaBoost+DT+LightGBM+SVM | 72.000 | Rank 83 | 0.778 (0.762-0.793) | 0.214 (0.171-0.256) | 0.622 (0.589-0.653) | 0.234 (0.186-0.283) | 0.846 (0.832-0.861) | 0.159 (0.131-0.188) | 0.156 (0.149-0.163) |
| RF+LightGBM+NaiveBayes+SVM | 72.143 | Rank 84 | 0.789 (0.773-0.804) | 0.252 (0.205-0.298) | 0.605 (0.568-0.640) | 0.275 (0.222-0.330) | 0.853 (0.838-0.867) | 0.154 (0.128-0.182) | 0.153 (0.145-0.160) |
| DT+RF+LightGBM+SVM | 73.143 | Rank 85 | 0.782 (0.767-0.798) | 0.220 (0.175-0.264) | 0.607 (0.574-0.641) | 0.240 (0.190-0.289) | 0.850 (0.835-0.863) | 0.161 (0.130-0.192) | 0.154 (0.147-0.161) |
| AdaBoost+DT+LightGBM+NaiveBayes+CatBoost | 73.571 | Rank 86 | 0.810 (0.794-0.826) | 0.179 (0.133-0.222) | 0.603 (0.570-0.636) | 0.182 (0.136-0.226) | 0.888 (0.876-0.902) | 0.154 (0.129-0.179) | 0.141 (0.134-0.149) |
| AdaBoost+DT+RF+LightGBM+NaiveBayes+SVM | 73.714 | Rank 87 | 0.803 (0.788-0.818) | 0.218 (0.171-0.260) | 0.587 (0.553-0.620) | 0.229 (0.178-0.275) | 0.874 (0.861-0.887) | 0.143 (0.120-0.164) | 0.147 (0.139-0.154) |
| AdaBoost+NaiveBayes+SVM+CatBoost | 74.286 | Rank 88 | 0.800 (0.785-0.816) | 0.187 (0.141-0.226) | 0.616 (0.584-0.651) | 0.195 (0.144-0.236) | 0.876 (0.863-0.890) | 0.155 (0.129-0.182) | 0.146 (0.139-0.153) |
| RF+NaiveBayes | 74.286 | Rank 88 | 0.791 (0.775-0.806) | 0.203 (0.158-0.248) | 0.617 (0.587-0.650) | 0.216 (0.168-0.264) | 0.862 (0.849-0.876) | 0.158 (0.132-0.184) | 0.152 (0.144-0.159) |
| RF+LightGBM+SVM+CatBoost | 74.571 | Rank 90 | 0.790 (0.774-0.806) | 0.198 (0.156-0.243) | 0.627 (0.595-0.659) | 0.211 (0.165-0.264) | 0.862 (0.847-0.877) | 0.151 (0.128-0.174) | 0.147 (0.139-0.155) |
| AdaBoost+DT+RF+NaiveBayes+SVM | 74.714 | Rank 91 | 0.789 (0.774-0.805) | 0.182 (0.139-0.220) | 0.621 (0.590-0.654) | 0.193 (0.146-0.234) | 0.864 (0.850-0.878) | 0.163 (0.135-0.193) | 0.147 (0.139-0.155) |
| SVM+CatBoost | 74.857 | Rank 92 | 0.802 (0.787-0.817) | 0.194 (0.151-0.233) | 0.608 (0.574-0.641) | 0.201 (0.155-0.244) | 0.877 (0.863-0.890) | 0.155 (0.130-0.182) | 0.146 (0.139-0.154) |
| DT+RF+LightGBM+NaiveBayes+SVM+CatBoost | 75.714 | Rank 93 | 0.772 (0.756-0.788) | 0.208 (0.167-0.250) | 0.620 (0.588-0.654) | 0.229 (0.183-0.276) | 0.840 (0.826-0.855) | 0.159 (0.134-0.186) | 0.156 (0.147-0.163) |
| AdaBoost+RF+LightGBM | 76.286 | Rank 94 | 0.794 (0.779-0.809) | 0.210 (0.164-0.253) | 0.607 (0.575-0.640) | 0.223 (0.172-0.270) | 0.866 (0.852-0.879) | 0.153 (0.127-0.179) | 0.147 (0.139-0.154) |
| AdaBoost+RF+LightGBM+SVM | 76.429 | Rank 95 | 0.780 (0.765-0.797) | 0.235 (0.190-0.278) | 0.607 (0.573-0.641) | 0.259 (0.209-0.306) | 0.845 (0.830-0.860) | 0.156 (0.128-0.186) | 0.159 (0.152-0.166) |
| AdaBoost+DT+LightGBM+NaiveBayes+SVM+CatBoost | 76.571 | Rank 96 | 0.788 (0.772-0.803) | 0.238 (0.190-0.284) | 0.597 (0.560-0.632) | 0.259 (0.206-0.314) | 0.853 (0.839-0.867) | 0.154 (0.126-0.181) | 0.152 (0.144-0.159) |
| AdaBoost+LightGBM+NaiveBayes+SVM+CatBoost | 76.571 | Rank 96 | 0.797 (0.782-0.812) | 0.195 (0.155-0.238) | 0.595 (0.560-0.631) | 0.205 (0.162-0.252) | 0.871 (0.857-0.884) | 0.162 (0.133-0.193) | 0.147 (0.139-0.155) |
| AdaBoost+RF+NaiveBayes+SVM+CatBoost | 76.857 | Rank 98 | 0.795 (0.780-0.811) | 0.191 (0.146-0.233) | 0.617 (0.585-0.647) | 0.201 (0.151-0.245) | 0.869 (0.856-0.883) | 0.157 (0.132-0.183) | 0.148 (0.141-0.155) |
| DT+NaiveBayes | 77.143 | Rank 99 | 0.752 (0.735-0.769) | 0.239 (0.194-0.280) | 0.599 (0.564-0.633) | 0.276 (0.224-0.324) | 0.812 (0.796-0.828) | 0.166 (0.134-0.201) | 0.172 (0.166-0.177) |
| AdaBoost+RF+LightGBM+NaiveBayes+CatBoost | 78.429 | Rank 100 | 0.810 (0.795-0.825) | 0.168 (0.127-0.210) | 0.596 (0.565-0.629) | 0.170 (0.128-0.216) | 0.890 (0.877-0.903) | 0.151 (0.126-0.179) | 0.141 (0.134-0.149) |
| NaiveBayes+SVM | 78.571 | Rank 101 | 0.777 (0.761-0.792) | 0.224 (0.178-0.264) | 0.598 (0.564-0.632) | 0.247 (0.194-0.296) | 0.843 (0.829-0.859) | 0.161 (0.131-0.190) | 0.159 (0.153-0.165) |
| AdaBoost+DT | 79.143 | Rank 102 | 0.745 (0.728-0.762) | 0.266 (0.223-0.307) | 0.612 (0.579-0.648) | 0.317 (0.262-0.369) | 0.798 (0.781-0.815) | 0.154 (0.130-0.178) | 0.171 (0.163-0.177) |
| SVM | 80.857 | Rank 103 | 0.759 (0.743-0.775) | 0.236 (0.189-0.277) | 0.595 (0.561-0.630) | 0.270 (0.218-0.320) | 0.820 (0.804-0.836) | 0.161 (0.129-0.195) | 0.171 (0.165-0.177) |
| AdaBoost+DT+RF+CatBoost | 81.143 | Rank 104 | 0.796 (0.781-0.811) | 0.175 (0.136-0.215) | 0.604 (0.573-0.635) | 0.182 (0.142-0.225) | 0.873 (0.859-0.886) | 0.159 (0.131-0.187) | 0.143 (0.135-0.151) |
| AdaBoost+DT+RF+LightGBM+NaiveBayes | 81.143 | Rank 104 | 0.795 (0.780-0.809) | 0.196 (0.151-0.236) | 0.611 (0.579-0.643) | 0.207 (0.159-0.250) | 0.868 (0.855-0.881) | 0.150 (0.126-0.173) | 0.149 (0.142-0.156) |
| DT | 81.429 | Rank 106 | 0.595 (0.575-0.613) | 0.376 (0.335-0.414) | 0.606 (0.571-0.642) | 0.589 (0.532-0.644) | 0.595 (0.575-0.616) | 0.156 (0.132-0.181) | 0.207 (0.200-0.214) |
| DT+RF | 81.857 | Rank 107 | 0.780 (0.764-0.795) | 0.190 (0.144-0.232) | 0.618 (0.586-0.651) | 0.205 (0.155-0.250) | 0.852 (0.837-0.866) | 0.159 (0.131-0.186) | 0.150 (0.143-0.157) |
| AdaBoost+RF+NaiveBayes | 82.429 | Rank 108 | 0.785 (0.770-0.801) | 0.217 (0.172-0.264) | 0.605 (0.572-0.638) | 0.235 (0.185-0.288) | 0.854 (0.841-0.868) | 0.150 (0.128-0.175) | 0.155 (0.147-0.162) |
| AdaBoost+NaiveBayes | 84.429 | Rank 109 | 0.736 (0.721-0.753) | 0.264 (0.218-0.305) | 0.608 (0.574-0.639) | 0.318 (0.265-0.369) | 0.789 (0.772-0.806) | 0.148 (0.123-0.172) | 0.172 (0.166-0.177) |
| DT+LightGBM | 84.429 | Rank 109 | 0.790 (0.774-0.806) | 0.218 (0.172-0.261) | 0.580 (0.546-0.618) | 0.234 (0.183-0.282) | 0.860 (0.845-0.874) | 0.152 (0.126-0.182) | 0.153 (0.146-0.160) |
| DT+SVM | 84.571 | Rank 111 | 0.766 (0.748-0.783) | 0.220 (0.176-0.261) | 0.592 (0.559-0.626) | 0.247 (0.196-0.298) | 0.831 (0.814-0.847) | 0.161 (0.132-0.192) | 0.167 (0.161-0.173) |
| LightGBM+SVM | 85.857 | Rank 112 | 0.778 (0.762-0.794) | 0.209 (0.166-0.248) | 0.604 (0.571-0.636) | 0.229 (0.180-0.275) | 0.846 (0.832-0.862) | 0.157 (0.129-0.185) | 0.156 (0.149-0.163) |
| DT+RF+NaiveBayes+CatBoost | 86.429 | Rank 113 | 0.804 (0.789-0.819) | 0.168 (0.128-0.209) | 0.592 (0.560-0.625) | 0.172 (0.129-0.216) | 0.883 (0.870-0.896) | 0.150 (0.124-0.178) | 0.143 (0.136-0.151) |
| RF+NaiveBayes+SVM | 87.571 | Rank 114 | 0.767 (0.751-0.783) | 0.216 (0.173-0.253) | 0.611 (0.581-0.641) | 0.242 (0.196-0.289) | 0.832 (0.817-0.848) | 0.144 (0.124-0.164) | 0.160 (0.153-0.167) |
| LightGBM+NaiveBayes | 87.714 | Rank 115 | 0.761 (0.744-0.778) | 0.214 (0.172-0.257) | 0.609 (0.574-0.642) | 0.242 (0.191-0.292) | 0.825 (0.810-0.841) | 0.154 (0.130-0.180) | 0.161 (0.154-0.169) |
| DT+RF+NaiveBayes | 88.429 | Rank 116 | 0.783 (0.767-0.798) | 0.202 (0.161-0.243) | 0.599 (0.564-0.631) | 0.218 (0.171-0.264) | 0.853 (0.839-0.867) | 0.157 (0.127-0.186) | 0.155 (0.148-0.162) |
| AdaBoost+RF+LightGBM+NaiveBayes+SVM | 88.714 | Rank 117 | 0.781 (0.765-0.796) | 0.196 (0.150-0.240) | 0.611 (0.578-0.644) | 0.212 (0.162-0.263) | 0.852 (0.837-0.867) | 0.151 (0.126-0.178) | 0.152 (0.145-0.159) |
| AdaBoost+LightGBM | 91.571 | Rank 118 | 0.778 (0.762-0.793) | 0.210 (0.167-0.252) | 0.601 (0.568-0.637) | 0.229 (0.183-0.277) | 0.846 (0.831-0.861) | 0.147 (0.123-0.172) | 0.157 (0.150-0.163) |
| XGBoost | 91.857 | Rank 119 | 0.754 (0.738-0.770) | 0.234 (0.191-0.278) | 0.592 (0.558-0.627) | 0.269 (0.218-0.321) | 0.815 (0.800-0.831) | 0.149 (0.125-0.175) | 0.197 (0.184-0.209) |
| LightGBM | 92.714 | Rank 120 | 0.759 (0.742-0.776) | 0.232 (0.189-0.276) | 0.585 (0.553-0.616) | 0.264 (0.215-0.315) | 0.821 (0.806-0.837) | 0.140 (0.119-0.163) | 0.167 (0.159-0.176) |
| KNN | 94.571 | Rank 121 | 0.635 (0.617-0.655) | 0.241 (0.201-0.282) | 0.524 (0.491-0.557) | 0.335 (0.283-0.394) | 0.673 (0.654-0.692) | 0.114 (0.099-0.130) | 0.247 (0.235-0.258) |
| DT+LightGBM+NaiveBayes+SVM | 94.714 | Rank 122 | 0.789 (0.773-0.804) | 0.194 (0.151-0.233) | 0.585 (0.551-0.621) | 0.206 (0.160-0.250) | 0.861 (0.848-0.875) | 0.153 (0.125-0.182) | 0.153 (0.146-0.160) |
| AdaBoost+LightGBM+CatBoost | 95.143 | Rank 123 | 0.798 (0.782-0.812) | 0.159 (0.115-0.201) | 0.598 (0.565-0.630) | 0.164 (0.118-0.210) | 0.877 (0.864-0.891) | 0.146 (0.124-0.171) | 0.147 (0.139-0.154) |
| DT+LightGBM+SVM+CatBoost | 96.143 | Rank 124 | 0.796 (0.781-0.812) | 0.175 (0.131-0.215) | 0.582 (0.547-0.614) | 0.182 (0.137-0.227) | 0.873 (0.859-0.887) | 0.146 (0.122-0.173) | 0.147 (0.139-0.155) |
| AdaBoost+DT+RF+NaiveBayes+CatBoost | 98.143 | Rank 125 | 0.785 (0.770-0.801) | 0.187 (0.142-0.226) | 0.591 (0.557-0.625) | 0.199 (0.152-0.245) | 0.858 (0.844-0.872) | 0.153 (0.124-0.185) | 0.150 (0.142-0.158) |
| DT+RF+LightGBM+NaiveBayes | 98.571 | Rank 126 | 0.798 (0.781-0.813) | 0.165 (0.125-0.204) | 0.590 (0.559-0.622) | 0.171 (0.128-0.213) | 0.876 (0.861-0.889) | 0.145 (0.121-0.171) | 0.150 (0.142-0.157) |
| DT+RF+SVM+CatBoost | 99.286 | Rank 127 | 0.783 (0.767-0.799) | 0.173 (0.131-0.212) | 0.609 (0.577-0.641) | 0.184 (0.138-0.228) | 0.858 (0.844-0.872) | 0.147 (0.123-0.172) | 0.151 (0.143-0.158) |
| DT+LightGBM+SVM | 104.571 | Rank 128 | 0.765 (0.750-0.782) | 0.191 (0.144-0.231) | 0.599 (0.566-0.632) | 0.212 (0.159-0.259) | 0.834 (0.819-0.851) | 0.142 (0.120-0.166) | 0.160 (0.152-0.167) |
| AdaBoost+DT+RF+NaiveBayes | 106.714 | Rank 129 | 0.785 (0.769-0.800) | 0.129 (0.093-0.164) | 0.599 (0.568-0.629) | 0.135 (0.097-0.171) | 0.866 (0.853-0.880) | 0.149 (0.122-0.174) | 0.152 (0.145-0.159) |

**Supplementary Table 31.** Ranking of 129 machine learning models when the CHARLS cohort was used as the testing dataset.

| **Model** | **Accuracy (95% CI)** | **F_Beta (95% CI)** | **ROC_AUC (95% CI)** | **Sensitivity (95% CI)** | **Specificity (95% CI)** | **PR_AUC (95% CI)** | **Brier (95% CI)** | **Normalized Rank Score** | **Comprehensive Rank** |
| --- | --- | --- | --- | --- | --- | --- | --- | --- | --- |
| AdaBoost+NaiveBayes+SVM | 0.801 (0.791-0.810) | 0.300 (0.270-0.328) | 0.638 (0.616-0.659) | 0.313 (0.279-0.343) | 0.871 (0.863-0.880) | 0.203 (0.182-0.223) | 0.152 (0.149-0.156) | 17.571 | Rank 1 |
| RF+LightGBM+NaiveBayes+SVM | 0.807 (0.797-0.817) | 0.268 (0.238-0.296) | 0.611 (0.589-0.633) | 0.272 (0.240-0.301) | 0.885 (0.876-0.893) | 0.197 (0.174-0.219) | 0.150 (0.145-0.154) | 24.714 | Rank 2 |
| AdaBoost+LightGBM+NaiveBayes+CatBoost | 0.801 (0.791-0.811) | 0.258 (0.230-0.287) | 0.610 (0.589-0.632) | 0.264 (0.235-0.295) | 0.878 (0.869-0.888) | 0.194 (0.174-0.215) | 0.148 (0.142-0.154) | 30.286 | Rank 3 |
| AdaBoost+LightGBM+NaiveBayes+SVM+CatBoost | 0.800 (0.791-0.810) | 0.266 (0.238-0.295) | 0.609 (0.587-0.631) | 0.273 (0.244-0.304) | 0.876 (0.868-0.885) | 0.189 (0.169-0.209) | 0.149 (0.143-0.155) | 30.286 | Rank 3 |
| LightGBM+NaiveBayes+CatBoost | 0.789 (0.779-0.798) | 0.257 (0.226-0.286) | 0.628 (0.606-0.649) | 0.268 (0.235-0.299) | 0.864 (0.854-0.873) | 0.198 (0.176-0.221) | 0.149 (0.144-0.154) | 33.571 | Rank 5 |
| AdaBoost+DT+NaiveBayes+SVM | 0.796 (0.786-0.805) | 0.254 (0.226-0.282) | 0.641 (0.620-0.661) | 0.262 (0.232-0.291) | 0.873 (0.865-0.882) | 0.200 (0.179-0.223) | 0.159 (0.155-0.164) | 34.571 | Rank 6 |
| AdaBoost | 0.791 (0.781-0.801) | 0.298 (0.270-0.328) | 0.636 (0.615-0.657) | 0.315 (0.284-0.349) | 0.859 (0.850-0.868) | 0.213 (0.189-0.236) | 0.181 (0.177-0.184) | 35.143 | Rank 7 |
| AdaBoost+NaiveBayes | 0.800 (0.790-0.810) | 0.252 (0.221-0.281) | 0.627 (0.605-0.648) | 0.257 (0.225-0.288) | 0.879 (0.871-0.888) | 0.193 (0.170-0.212) | 0.159 (0.155-0.163) | 36.286 | Rank 8 |
| AdaBoost+SVM | 0.815 (0.805-0.824) | 0.208 (0.178-0.237) | 0.635 (0.615-0.657) | 0.203 (0.173-0.233) | 0.903 (0.895-0.911) | 0.194 (0.174-0.215) | 0.143 (0.139-0.147) | 37.143 | Rank 9 |
| AdaBoost+DT+RF+SVM | 0.790 (0.780-0.801) | 0.262 (0.232-0.291) | 0.616 (0.592-0.638) | 0.273 (0.241-0.305) | 0.865 (0.856-0.874) | 0.191 (0.170-0.210) | 0.157 (0.153-0.161) | 38.429 | Rank 10 |
| AdaBoost+DT+SVM | 0.810 (0.800-0.820) | 0.224 (0.194-0.252) | 0.640 (0.619-0.660) | 0.222 (0.192-0.250) | 0.895 (0.886-0.903) | 0.194 (0.174-0.213) | 0.155 (0.151-0.159) | 39.429 | Rank 11 |
| RF+SVM | 0.777 (0.766-0.788) | 0.282 (0.251-0.311) | 0.625 (0.605-0.646) | 0.304 (0.270-0.338) | 0.845 (0.836-0.855) | 0.190 (0.171-0.209) | 0.156 (0.151-0.161) | 39.714 | Rank 12 |
| AdaBoost+RF+LightGBM+SVM | 0.795 (0.785-0.805) | 0.250 (0.221-0.277) | 0.604 (0.581-0.626) | 0.257 (0.226-0.286) | 0.873 (0.864-0.881) | 0.186 (0.166-0.206) | 0.151 (0.147-0.156) | 40.714 | Rank 13 |
| AdaBoost+DT+LightGBM+NaiveBayes+SVM | 0.774 (0.764-0.784) | 0.264 (0.236-0.291) | 0.628 (0.607-0.648) | 0.284 (0.252-0.314) | 0.844 (0.835-0.854) | 0.202 (0.179-0.224) | 0.156 (0.151-0.161) | 41.571 | Rank 14 |
| AdaBoost+DT+LightGBM+CatBoost | 0.801 (0.791-0.811) | 0.230 (0.201-0.260) | 0.607 (0.585-0.629) | 0.232 (0.201-0.264) | 0.883 (0.875-0.892) | 0.184 (0.165-0.203) | 0.146 (0.141-0.152) | 43.429 | Rank 15 |
| AdaBoost+DT+NaiveBayes | 0.782 (0.771-0.792) | 0.277 (0.247-0.306) | 0.628 (0.607-0.649) | 0.295 (0.263-0.328) | 0.852 (0.842-0.862) | 0.191 (0.172-0.211) | 0.170 (0.166-0.174) | 45.143 | Rank 16 |
| RF+LightGBM+SVM | 0.765 (0.754-0.775) | 0.303 (0.275-0.331) | 0.607 (0.586-0.630) | 0.335 (0.303-0.370) | 0.827 (0.817-0.837) | 0.188 (0.167-0.208) | 0.161 (0.156-0.167) | 45.429 | Rank 17 |
| DT+CatBoost | 0.788 (0.778-0.798) | 0.241 (0.213-0.268) | 0.612 (0.591-0.635) | 0.250 (0.220-0.279) | 0.866 (0.857-0.875) | 0.184 (0.163-0.205) | 0.153 (0.148-0.158) | 48.000 | Rank 18 |
| LightGBM+SVM+CatBoost | 0.794 (0.784-0.804) | 0.246 (0.216-0.274) | 0.595 (0.574-0.616) | 0.252 (0.220-0.283) | 0.873 (0.863-0.882) | 0.189 (0.168-0.212) | 0.153 (0.148-0.159) | 48.000 | Rank 18 |
| DT+RF+NaiveBayes+SVM | 0.775 (0.764-0.785) | 0.257 (0.228-0.285) | 0.633 (0.611-0.652) | 0.275 (0.242-0.306) | 0.848 (0.837-0.857) | 0.187 (0.169-0.206) | 0.162 (0.157-0.166) | 48.714 | Rank 20 |
| AdaBoost+DT+RF | 0.791 (0.781-0.801) | 0.229 (0.200-0.254) | 0.621 (0.601-0.642) | 0.234 (0.205-0.262) | 0.872 (0.863-0.880) | 0.180 (0.163-0.199) | 0.148 (0.143-0.152) | 48.857 | Rank 21 |
| RF+LightGBM+NaiveBayes | 0.759 (0.749-0.770) | 0.294 (0.265-0.320) | 0.619 (0.597-0.642) | 0.327 (0.296-0.358) | 0.822 (0.811-0.832) | 0.199 (0.178-0.222) | 0.168 (0.163-0.173) | 48.857 | Rank 21 |
| AdaBoost+DT+RF+LightGBM+SVM | 0.762 (0.752-0.773) | 0.284 (0.256-0.311) | 0.614 (0.593-0.636) | 0.313 (0.281-0.344) | 0.827 (0.817-0.838) | 0.183 (0.164-0.203) | 0.160 (0.155-0.166) | 51.000 | Rank 23 |
| AdaBoost+DT | 0.742 (0.732-0.753) | 0.333 (0.304-0.361) | 0.652 (0.634-0.673) | 0.387 (0.352-0.422) | 0.793 (0.783-0.804) | 0.211 (0.189-0.233) | 0.180 (0.176-0.183) | 51.143 | Rank 24 |
| DT | 0.737 (0.726-0.748) | 0.342 (0.313-0.368) | 0.610 (0.590-0.630) | 0.402 (0.367-0.436) | 0.786 (0.775-0.796) | 0.194 (0.176-0.212) | 0.166 (0.161-0.171) | 51.571 | Rank 25 |
| RF+NaiveBayes+SVM | 0.801 (0.791-0.811) | 0.233 (0.205-0.260) | 0.597 (0.574-0.619) | 0.235 (0.206-0.263) | 0.883 (0.875-0.892) | 0.180 (0.161-0.198) | 0.152 (0.147-0.156) | 51.714 | Rank 26 |
| NaiveBayes+CatBoost | 0.781 (0.772-0.790) | 0.250 (0.218-0.278) | 0.600 (0.578-0.622) | 0.264 (0.230-0.293) | 0.856 (0.847-0.865) | 0.186 (0.164-0.208) | 0.155 (0.149-0.160) | 52.143 | Rank 27 |
| RF+NaiveBayes+SVM+CatBoost | 0.786 (0.776-0.796) | 0.229 (0.200-0.257) | 0.614 (0.591-0.635) | 0.237 (0.206-0.268) | 0.865 (0.856-0.874) | 0.187 (0.168-0.210) | 0.154 (0.149-0.159) | 52.429 | Rank 28 |
| NaiveBayes+SVM+CatBoost | 0.756 (0.746-0.767) | 0.309 (0.281-0.337) | 0.612 (0.591-0.633) | 0.349 (0.317-0.381) | 0.815 (0.805-0.826) | 0.192 (0.172-0.214) | 0.171 (0.166-0.177) | 52.571 | Rank 29 |
| AdaBoost+DT+LightGBM+NaiveBayes+CatBoost | 0.793 (0.783-0.803) | 0.221 (0.193-0.251) | 0.608 (0.586-0.630) | 0.225 (0.196-0.257) | 0.875 (0.866-0.883) | 0.180 (0.161-0.198) | 0.147 (0.142-0.153) | 52.857 | Rank 30 |
| DT+RF+NaiveBayes+CatBoost | 0.759 (0.749-0.770) | 0.320 (0.292-0.349) | 0.609 (0.587-0.631) | 0.360 (0.326-0.395) | 0.817 (0.807-0.828) | 0.185 (0.167-0.205) | 0.171 (0.166-0.176) | 54.000 | Rank 31 |
| AdaBoost+DT+CatBoost | 0.796 (0.786-0.806) | 0.229 (0.202-0.257) | 0.596 (0.574-0.618) | 0.233 (0.204-0.262) | 0.878 (0.869-0.886) | 0.182 (0.163-0.202) | 0.152 (0.147-0.157) | 54.429 | Rank 32 |
| AdaBoost+DT+LightGBM | 0.752 (0.741-0.763) | 0.293 (0.263-0.321) | 0.615 (0.592-0.637) | 0.330 (0.296-0.363) | 0.813 (0.803-0.823) | 0.192 (0.169-0.215) | 0.170 (0.164-0.175) | 54.857 | Rank 33 |
| RF+LightGBM+NaiveBayes+CatBoost | 0.771 (0.760-0.781) | 0.261 (0.234-0.289) | 0.610 (0.589-0.632) | 0.281 (0.253-0.313) | 0.841 (0.831-0.851) | 0.187 (0.166-0.208) | 0.164 (0.159-0.170) | 54.857 | Rank 33 |
| RF+NaiveBayes | 0.762 (0.751-0.772) | 0.291 (0.263-0.319) | 0.614 (0.592-0.636) | 0.322 (0.291-0.354) | 0.826 (0.815-0.835) | 0.189 (0.169-0.209) | 0.177 (0.173-0.181) | 55.286 | Rank 35 |
| AdaBoost+RF+LightGBM+NaiveBayes+SVM+CatBoost | 0.806 (0.796-0.816) | 0.218 (0.189-0.246) | 0.594 (0.570-0.617) | 0.216 (0.186-0.245) | 0.891 (0.883-0.899) | 0.179 (0.160-0.199) | 0.146 (0.141-0.152) | 56.286 | Rank 36 |
| SVM+CatBoost | 0.759 (0.748-0.770) | 0.302 (0.275-0.330) | 0.604 (0.582-0.626) | 0.338 (0.307-0.370) | 0.820 (0.810-0.831) | 0.186 (0.165-0.205) | 0.175 (0.170-0.181) | 57.857 | Rank 37 |
| AdaBoost+RF+NaiveBayes | 0.781 (0.769-0.791) | 0.241 (0.212-0.269) | 0.611 (0.587-0.633) | 0.253 (0.222-0.284) | 0.857 (0.847-0.866) | 0.180 (0.161-0.200) | 0.161 (0.156-0.165) | 58.143 | Rank 38 |
| DT+RF+NaiveBayes | 0.785 (0.775-0.796) | 0.230 (0.202-0.258) | 0.621 (0.601-0.643) | 0.239 (0.208-0.269) | 0.864 (0.855-0.873) | 0.182 (0.165-0.200) | 0.163 (0.159-0.167) | 58.286 | Rank 39 |
| AdaBoost+RF+NaiveBayes+SVM | 0.756 (0.746-0.768) | 0.294 (0.266-0.321) | 0.614 (0.591-0.635) | 0.328 (0.296-0.359) | 0.818 (0.808-0.829) | 0.178 (0.161-0.195) | 0.166 (0.161-0.171) | 58.429 | Rank 40 |
| DT+LightGBM+CatBoost | 0.727 (0.717-0.738) | 0.317 (0.289-0.342) | 0.616 (0.595-0.639) | 0.375 (0.341-0.407) | 0.778 (0.767-0.789) | 0.192 (0.169-0.214) | 0.185 (0.179-0.190) | 58.571 | Rank 41 |
| AdaBoost+DT+LightGBM+SVM+CatBoost | 0.788 (0.778-0.799) | 0.242 (0.212-0.269) | 0.597 (0.572-0.618) | 0.250 (0.218-0.281) | 0.866 (0.857-0.875) | 0.175 (0.157-0.193) | 0.151 (0.146-0.157) | 58.714 | Rank 42 |
| AdaBoost+DT+RF+LightGBM+NaiveBayes | 0.770 (0.760-0.781) | 0.253 (0.223-0.279) | 0.605 (0.583-0.628) | 0.271 (0.239-0.300) | 0.842 (0.833-0.852) | 0.181 (0.162-0.201) | 0.159 (0.154-0.163) | 58.714 | Rank 42 |
| AdaBoost+CatBoost | 0.759 (0.747-0.770) | 0.277 (0.249-0.307) | 0.609 (0.586-0.631) | 0.306 (0.273-0.341) | 0.824 (0.813-0.835) | 0.186 (0.167-0.206) | 0.167 (0.161-0.173) | 58.857 | Rank 44 |
| LightGBM | 0.802 (0.792-0.813) | 0.212 (0.181-0.240) | 0.592 (0.570-0.613) | 0.212 (0.181-0.241) | 0.887 (0.879-0.896) | 0.178 (0.159-0.197) | 0.146 (0.141-0.152) | 59.000 | Rank 45 |
| AdaBoost+RF | 0.791 (0.781-0.801) | 0.232 (0.206-0.259) | 0.606 (0.585-0.628) | 0.238 (0.210-0.267) | 0.871 (0.862-0.880) | 0.178 (0.161-0.196) | 0.161 (0.157-0.165) | 60.000 | Rank 46 |
| DT+LightGBM+SVM+CatBoost | 0.727 (0.716-0.738) | 0.335 (0.305-0.362) | 0.606 (0.582-0.628) | 0.399 (0.364-0.434) | 0.775 (0.763-0.786) | 0.194 (0.172-0.217) | 0.191 (0.185-0.197) | 61.429 | Rank 47 |
| AdaBoost+DT+RF+LightGBM+SVM+CatBoost | 0.788 (0.777-0.798) | 0.243 (0.214-0.271) | 0.583 (0.560-0.607) | 0.252 (0.221-0.283) | 0.865 (0.856-0.874) | 0.180 (0.160-0.202) | 0.153 (0.147-0.158) | 62.000 | Rank 48 |
| RF+SVM+CatBoost | 0.765 (0.755-0.775) | 0.263 (0.233-0.290) | 0.603 (0.580-0.625) | 0.286 (0.254-0.316) | 0.835 (0.824-0.844) | 0.184 (0.163-0.205) | 0.168 (0.163-0.174) | 62.000 | Rank 48 |
| LightGBM+NaiveBayes+SVM+CatBoost | 0.768 (0.757-0.778) | 0.251 (0.223-0.278) | 0.606 (0.585-0.628) | 0.271 (0.239-0.301) | 0.839 (0.829-0.849) | 0.179 (0.160-0.199) | 0.160 (0.155-0.167) | 62.143 | Rank 50 |
| DT+LightGBM+NaiveBayes+SVM | 0.762 (0.752-0.772) | 0.278 (0.250-0.307) | 0.600 (0.580-0.622) | 0.306 (0.273-0.337) | 0.828 (0.819-0.838) | 0.180 (0.161-0.199) | 0.166 (0.162-0.171) | 62.286 | Rank 51 |
| AdaBoost+DT+RF+LightGBM+NaiveBayes+SVM+CatBoost | 0.764 (0.753-0.775) | 0.293 (0.264-0.322) | 0.595 (0.571-0.619) | 0.323 (0.290-0.357) | 0.827 (0.818-0.838) | 0.181 (0.162-0.200) | 0.170 (0.164-0.176) | 63.286 | Rank 52 |
| AdaBoost+LightGBM+SVM+CatBoost | 0.777 (0.768-0.788) | 0.262 (0.233-0.292) | 0.588 (0.567-0.611) | 0.279 (0.247-0.314) | 0.849 (0.840-0.859) | 0.180 (0.160-0.199) | 0.163 (0.157-0.168) | 63.429 | Rank 53 |
| AdaBoost+RF+NaiveBayes+CatBoost | 0.809 (0.799-0.819) | 0.158 (0.132-0.182) | 0.596 (0.574-0.618) | 0.152 (0.127-0.176) | 0.904 (0.896-0.912) | 0.171 (0.154-0.188) | 0.141 (0.135-0.146) | 63.429 | Rank 53 |
| DT+SVM | 0.822 (0.812-0.832) | 0.163 (0.137-0.191) | 0.598 (0.576-0.620) | 0.154 (0.129-0.181) | 0.918 (0.911-0.926) | 0.172 (0.155-0.190) | 0.154 (0.151-0.158) | 63.429 | Rank 53 |
| AdaBoost+LightGBM | 0.760 (0.750-0.771) | 0.277 (0.248-0.306) | 0.600 (0.579-0.625) | 0.306 (0.271-0.339) | 0.826 (0.816-0.836) | 0.183 (0.163-0.205) | 0.169 (0.163-0.173) | 63.571 | Rank 56 |
| AdaBoost+DT+RF+NaiveBayes | 0.773 (0.763-0.784) | 0.248 (0.218-0.276) | 0.599 (0.577-0.619) | 0.265 (0.233-0.296) | 0.847 (0.837-0.857) | 0.183 (0.163-0.202) | 0.164 (0.160-0.168) | 63.714 | Rank 57 |
| AdaBoost+LightGBM+NaiveBayes | 0.780 (0.770-0.791) | 0.249 (0.221-0.277) | 0.594 (0.572-0.616) | 0.262 (0.233-0.294) | 0.855 (0.845-0.864) | 0.179 (0.160-0.198) | 0.158 (0.152-0.163) | 63.714 | Rank 57 |
| RF+CatBoost | 0.785 (0.775-0.795) | 0.245 (0.215-0.272) | 0.595 (0.573-0.617) | 0.255 (0.223-0.285) | 0.862 (0.852-0.871) | 0.175 (0.157-0.193) | 0.156 (0.151-0.161) | 63.714 | Rank 57 |
| AdaBoost+DT+LightGBM+NaiveBayes | 0.761 (0.751-0.772) | 0.294 (0.261-0.320) | 0.584 (0.559-0.606) | 0.326 (0.291-0.357) | 0.824 (0.814-0.834) | 0.193 (0.170-0.214) | 0.171 (0.166-0.176) | 63.857 | Rank 60 |
| AdaBoost+DT+RF+LightGBM+NaiveBayes+CatBoost | 0.781 (0.771-0.791) | 0.242 (0.214-0.269) | 0.602 (0.582-0.624) | 0.254 (0.223-0.284) | 0.857 (0.848-0.866) | 0.175 (0.156-0.192) | 0.158 (0.153-0.163) | 63.857 | Rank 60 |
| AdaBoost+DT+RF+NaiveBayes+SVM+CatBoost | 0.771 (0.760-0.782) | 0.252 (0.223-0.281) | 0.603 (0.582-0.624) | 0.270 (0.238-0.302) | 0.844 (0.835-0.854) | 0.179 (0.161-0.197) | 0.163 (0.157-0.168) | 63.857 | Rank 60 |
| AdaBoost+RF+SVM+CatBoost | 0.771 (0.760-0.781) | 0.253 (0.222-0.283) | 0.597 (0.574-0.620) | 0.271 (0.237-0.304) | 0.843 (0.833-0.853) | 0.179 (0.159-0.198) | 0.158 (0.152-0.164) | 64.143 | Rank 63 |
| AdaBoost+NaiveBayes+CatBoost | 0.766 (0.755-0.776) | 0.280 (0.250-0.311) | 0.592 (0.570-0.615) | 0.306 (0.274-0.339) | 0.832 (0.822-0.842) | 0.181 (0.162-0.200) | 0.167 (0.162-0.173) | 64.429 | Rank 64 |
| AdaBoost+NaiveBayes+SVM+CatBoost | 0.789 (0.780-0.800) | 0.237 (0.208-0.265) | 0.586 (0.564-0.608) | 0.245 (0.214-0.275) | 0.868 (0.859-0.878) | 0.179 (0.159-0.198) | 0.156 (0.151-0.161) | 64.429 | Rank 64 |
| DT+NaiveBayes+SVM | 0.810 (0.801-0.820) | 0.156 (0.131-0.179) | 0.595 (0.571-0.616) | 0.150 (0.125-0.173) | 0.906 (0.898-0.914) | 0.172 (0.155-0.189) | 0.149 (0.145-0.152) | 64.857 | Rank 66 |
| DT+RF+LightGBM+NaiveBayes+SVM | 0.766 (0.756-0.777) | 0.260 (0.233-0.287) | 0.608 (0.587-0.630) | 0.282 (0.252-0.313) | 0.836 (0.826-0.846) | 0.179 (0.162-0.198) | 0.170 (0.166-0.175) | 65.000 | Rank 67 |
| RF+LightGBM+CatBoost | 0.808 (0.797-0.817) | 0.187 (0.159-0.213) | 0.586 (0.564-0.607) | 0.182 (0.156-0.210) | 0.899 (0.890-0.906) | 0.172 (0.154-0.189) | 0.146 (0.141-0.152) | 65.000 | Rank 67 |
| DT+NaiveBayes | 0.821 (0.812-0.831) | 0.187 (0.159-0.213) | 0.575 (0.551-0.597) | 0.179 (0.151-0.206) | 0.914 (0.907-0.921) | 0.173 (0.157-0.190) | 0.143 (0.138-0.147) | 65.143 | Rank 69 |
| AdaBoost+RF+NaiveBayes+SVM+CatBoost | 0.784 (0.774-0.794) | 0.220 (0.196-0.246) | 0.601 (0.580-0.624) | 0.228 (0.201-0.256) | 0.864 (0.855-0.874) | 0.180 (0.162-0.199) | 0.157 (0.151-0.162) | 66.286 | Rank 70 |
| RF+NaiveBayes+CatBoost | 0.754 (0.743-0.765) | 0.295 (0.266-0.323) | 0.593 (0.571-0.617) | 0.331 (0.297-0.363) | 0.815 (0.805-0.825) | 0.187 (0.166-0.208) | 0.178 (0.174-0.183) | 66.714 | Rank 71 |
| AdaBoost+RF+SVM | 0.761 (0.750-0.772) | 0.286 (0.258-0.313) | 0.600 (0.577-0.622) | 0.317 (0.285-0.348) | 0.825 (0.814-0.835) | 0.179 (0.162-0.197) | 0.172 (0.167-0.177) | 66.857 | Rank 72 |
| DT+RF+SVM | 0.774 (0.763-0.785) | 0.244 (0.217-0.274) | 0.603 (0.582-0.627) | 0.260 (0.229-0.293) | 0.848 (0.838-0.857) | 0.175 (0.157-0.194) | 0.163 (0.159-0.167) | 67.714 | Rank 73 |
| AdaBoost+SVM+CatBoost | 0.762 (0.752-0.773) | 0.261 (0.233-0.289) | 0.597 (0.575-0.621) | 0.285 (0.253-0.317) | 0.831 (0.821-0.841) | 0.176 (0.158-0.197) | 0.162 (0.156-0.167) | 68.143 | Rank 74 |
| RF+LightGBM | 0.781 (0.771-0.792) | 0.241 (0.212-0.268) | 0.603 (0.582-0.624) | 0.253 (0.221-0.284) | 0.858 (0.849-0.868) | 0.172 (0.155-0.189) | 0.163 (0.158-0.168) | 68.143 | Rank 74 |
| AdaBoost+RF+LightGBM+NaiveBayes | 0.780 (0.770-0.791) | 0.229 (0.200-0.257) | 0.597 (0.575-0.618) | 0.239 (0.209-0.269) | 0.859 (0.850-0.868) | 0.178 (0.159-0.198) | 0.157 (0.152-0.162) | 68.286 | Rank 76 |
| AdaBoost+LightGBM+NaiveBayes+SVM | 0.751 (0.740-0.762) | 0.274 (0.246-0.301) | 0.602 (0.579-0.625) | 0.305 (0.274-0.340) | 0.816 (0.805-0.826) | 0.186 (0.165-0.207) | 0.176 (0.171-0.180) | 68.571 | Rank 77 |
| AdaBoost+DT+RF+NaiveBayes+CatBoost | 0.796 (0.786-0.806) | 0.214 (0.188-0.244) | 0.587 (0.565-0.609) | 0.217 (0.188-0.247) | 0.880 (0.871-0.889) | 0.169 (0.153-0.186) | 0.151 (0.145-0.156) | 68.714 | Rank 78 |
| DT+LightGBM+NaiveBayes+SVM+CatBoost | 0.757 (0.746-0.767) | 0.270 (0.241-0.299) | 0.601 (0.578-0.623) | 0.298 (0.265-0.331) | 0.823 (0.813-0.833) | 0.184 (0.164-0.203) | 0.172 (0.167-0.178) | 68.714 | Rank 78 |
| DT+RF+LightGBM+SVM+CatBoost | 0.741 (0.729-0.751) | 0.299 (0.273-0.327) | 0.610 (0.590-0.632) | 0.344 (0.312-0.377) | 0.798 (0.787-0.808) | 0.175 (0.159-0.192) | 0.181 (0.176-0.186) | 68.857 | Rank 80 |
| AdaBoost+DT+NaiveBayes+CatBoost | 0.763 (0.752-0.774) | 0.273 (0.243-0.301) | 0.596 (0.573-0.618) | 0.299 (0.265-0.332) | 0.830 (0.819-0.840) | 0.176 (0.158-0.195) | 0.169 (0.163-0.175) | 70.429 | Rank 81 |
| AdaBoost+DT+RF+CatBoost | 0.793 (0.783-0.803) | 0.192 (0.168-0.217) | 0.587 (0.567-0.609) | 0.193 (0.168-0.220) | 0.880 (0.871-0.888) | 0.170 (0.153-0.188) | 0.147 (0.142-0.152) | 70.571 | Rank 82 |
| AdaBoost+DT+RF+LightGBM+NaiveBayes+SVM | 0.753 (0.742-0.764) | 0.273 (0.243-0.301) | 0.603 (0.581-0.625) | 0.304 (0.271-0.337) | 0.818 (0.808-0.830) | 0.178 (0.159-0.198) | 0.171 (0.166-0.176) | 71.714 | Rank 83 |
| DT+RF+LightGBM+NaiveBayes+SVM+CatBoost | 0.732 (0.721-0.743) | 0.293 (0.264-0.321) | 0.599 (0.576-0.621) | 0.340 (0.304-0.374) | 0.789 (0.778-0.799) | 0.182 (0.163-0.202) | 0.188 (0.182-0.193) | 71.857 | Rank 84 |
| AdaBoost+LightGBM+SVM | 0.752 (0.741-0.763) | 0.272 (0.241-0.300) | 0.598 (0.574-0.621) | 0.303 (0.269-0.335) | 0.817 (0.807-0.827) | 0.184 (0.162-0.205) | 0.176 (0.171-0.181) | 72.286 | Rank 85 |
| RF+LightGBM+SVM+CatBoost | 0.786 (0.776-0.797) | 0.229 (0.200-0.256) | 0.587 (0.565-0.608) | 0.237 (0.208-0.268) | 0.865 (0.856-0.875) | 0.173 (0.154-0.191) | 0.156 (0.151-0.162) | 72.286 | Rank 85 |
| DT+LightGBM+NaiveBayes+CatBoost | 0.793 (0.783-0.803) | 0.216 (0.188-0.243) | 0.581 (0.556-0.603) | 0.219 (0.190-0.249) | 0.876 (0.868-0.885) | 0.171 (0.153-0.191) | 0.152 (0.146-0.158) | 72.429 | Rank 87 |
| RF+LightGBM+NaiveBayes+SVM+CatBoost | 0.766 (0.756-0.776) | 0.246 (0.215-0.274) | 0.597 (0.572-0.619) | 0.265 (0.230-0.296) | 0.838 (0.828-0.847) | 0.175 (0.157-0.193) | 0.166 (0.160-0.171) | 72.857 | Rank 88 |
| DT+RF+LightGBM+NaiveBayes+CatBoost | 0.787 (0.777-0.797) | 0.216 (0.188-0.245) | 0.577 (0.555-0.598) | 0.222 (0.192-0.253) | 0.868 (0.860-0.878) | 0.176 (0.157-0.195) | 0.152 (0.147-0.158) | 73.857 | Rank 89 |
| DT+RF+LightGBM+SVM | 0.804 (0.794-0.814) | 0.208 (0.179-0.235) | 0.570 (0.550-0.592) | 0.207 (0.178-0.236) | 0.890 (0.882-0.899) | 0.169 (0.150-0.188) | 0.154 (0.149-0.159) | 74.286 | Rank 90 |
| CatBoost | 0.744 (0.734-0.756) | 0.293 (0.265-0.320) | 0.590 (0.568-0.611) | 0.333 (0.301-0.366) | 0.804 (0.793-0.814) | 0.181 (0.162-0.200) | 0.182 (0.177-0.186) | 74.429 | Rank 91 |
| DT+RF+LightGBM | 0.752 (0.742-0.763) | 0.272 (0.243-0.300) | 0.607 (0.585-0.628) | 0.304 (0.270-0.337) | 0.817 (0.807-0.827) | 0.172 (0.156-0.189) | 0.170 (0.165-0.174) | 74.571 | Rank 92 |
| AdaBoost+RF+LightGBM | 0.790 (0.781-0.800) | 0.213 (0.184-0.240) | 0.581 (0.558-0.604) | 0.217 (0.186-0.247) | 0.873 (0.864-0.882) | 0.172 (0.154-0.192) | 0.154 (0.150-0.159) | 74.857 | Rank 93 |
| DT+RF+NaiveBayes+SVM+CatBoost | 0.805 (0.795-0.816) | 0.163 (0.137-0.188) | 0.559 (0.536-0.582) | 0.159 (0.132-0.183) | 0.899 (0.891-0.908) | 0.157 (0.141-0.173) | 0.147 (0.141-0.152) | 74.857 | Rank 93 |
| AdaBoost+DT+RF+LightGBM+CatBoost | 0.787 (0.776-0.797) | 0.197 (0.169-0.225) | 0.588 (0.564-0.609) | 0.201 (0.172-0.230) | 0.872 (0.863-0.880) | 0.172 (0.154-0.192) | 0.153 (0.148-0.159) | 75.429 | Rank 95 |
| DT+NaiveBayes+SVM+CatBoost | 0.739 (0.728-0.749) | 0.285 (0.253-0.313) | 0.595 (0.572-0.617) | 0.326 (0.290-0.359) | 0.798 (0.788-0.809) | 0.181 (0.160-0.202) | 0.182 (0.176-0.187) | 75.429 | Rank 95 |
| DT+RF+SVM+CatBoost | 0.776 (0.765-0.787) | 0.246 (0.218-0.274) | 0.584 (0.561-0.607) | 0.261 (0.230-0.291) | 0.851 (0.841-0.860) | 0.177 (0.157-0.198) | 0.166 (0.161-0.172) | 75.571 | Rank 97 |
| DT+RF+LightGBM+CatBoost | 0.731 (0.720-0.742) | 0.292 (0.266-0.318) | 0.602 (0.580-0.623) | 0.340 (0.308-0.372) | 0.788 (0.778-0.799) | 0.174 (0.157-0.191) | 0.185 (0.180-0.190) | 76.857 | Rank 98 |
| AdaBoost+RF+LightGBM+NaiveBayes+CatBoost | 0.789 (0.778-0.800) | 0.206 (0.178-0.233) | 0.592 (0.569-0.615) | 0.210 (0.181-0.237) | 0.872 (0.864-0.882) | 0.168 (0.150-0.185) | 0.155 (0.150-0.160) | 77.000 | Rank 99 |
| AdaBoost+DT+NaiveBayes+SVM+CatBoost | 0.706 (0.695-0.717) | 0.320 (0.292-0.347) | 0.596 (0.575-0.619) | 0.392 (0.356-0.425) | 0.751 (0.740-0.762) | 0.172 (0.155-0.190) | 0.200 (0.194-0.205) | 78.857 | Rank 100 |
| AdaBoost+DT+SVM+CatBoost | 0.745 (0.735-0.756) | 0.271 (0.242-0.299) | 0.589 (0.567-0.611) | 0.306 (0.272-0.338) | 0.808 (0.798-0.819) | 0.181 (0.161-0.203) | 0.178 (0.173-0.183) | 78.857 | Rank 100 |
| AdaBoost+DT+LightGBM+NaiveBayes+SVM+CatBoost | 0.779 (0.769-0.790) | 0.231 (0.203-0.257) | 0.585 (0.563-0.608) | 0.242 (0.211-0.270) | 0.857 (0.848-0.867) | 0.170 (0.153-0.187) | 0.158 (0.152-0.163) | 79.286 | Rank 102 |
| AdaBoost+LightGBM+CatBoost | 0.775 (0.765-0.786) | 0.237 (0.209-0.263) | 0.585 (0.565-0.607) | 0.250 (0.220-0.280) | 0.851 (0.842-0.861) | 0.170 (0.153-0.188) | 0.158 (0.153-0.164) | 79.429 | Rank 103 |
| AdaBoost+DT+RF+LightGBM | 0.791 (0.781-0.801) | 0.211 (0.184-0.239) | 0.571 (0.548-0.594) | 0.215 (0.187-0.245) | 0.874 (0.866-0.883) | 0.166 (0.150-0.185) | 0.154 (0.149-0.159) | 79.571 | Rank 104 |
| AdaBoost+RF+CatBoost | 0.727 (0.717-0.739) | 0.299 (0.269-0.327) | 0.590 (0.567-0.612) | 0.350 (0.316-0.384) | 0.782 (0.771-0.793) | 0.174 (0.156-0.194) | 0.190 (0.184-0.195) | 80.000 | Rank 105 |
| AdaBoost+RF+LightGBM+SVM+CatBoost | 0.746 (0.735-0.756) | 0.282 (0.254-0.311) | 0.598 (0.577-0.619) | 0.319 (0.287-0.352) | 0.808 (0.797-0.818) | 0.168 (0.152-0.186) | 0.174 (0.169-0.179) | 80.429 | Rank 106 |
| DT+RF | 0.779 (0.769-0.790) | 0.240 (0.209-0.268) | 0.585 (0.562-0.609) | 0.252 (0.221-0.284) | 0.855 (0.846-0.865) | 0.171 (0.153-0.189) | 0.163 (0.158-0.168) | 80.571 | Rank 107 |
| RF | 0.788 (0.777-0.797) | 0.221 (0.193-0.247) | 0.596 (0.574-0.617) | 0.227 (0.199-0.254) | 0.868 (0.860-0.877) | 0.173 (0.154-0.190) | 0.181 (0.178-0.184) | 81.143 | Rank 108 |
| AdaBoost+RF+LightGBM+CatBoost | 0.778 (0.768-0.788) | 0.232 (0.205-0.260) | 0.581 (0.559-0.604) | 0.243 (0.213-0.275) | 0.855 (0.846-0.864) | 0.171 (0.153-0.190) | 0.159 (0.154-0.164) | 81.286 | Rank 109 |
| AdaBoost+DT+LightGBM+SVM | 0.778 (0.767-0.788) | 0.236 (0.207-0.264) | 0.586 (0.566-0.606) | 0.248 (0.217-0.279) | 0.854 (0.844-0.864) | 0.168 (0.151-0.183) | 0.161 (0.156-0.167) | 81.429 | Rank 110 |
| LightGBM+SVM | 0.780 (0.770-0.791) | 0.224 (0.197-0.251) | 0.582 (0.560-0.603) | 0.234 (0.204-0.263) | 0.859 (0.849-0.869) | 0.174 (0.155-0.193) | 0.163 (0.158-0.168) | 82.429 | Rank 111 |
| AdaBoost+DT+RF+NaiveBayes+SVM | 0.787 (0.776-0.797) | 0.221 (0.193-0.248) | 0.582 (0.558-0.606) | 0.228 (0.198-0.257) | 0.867 (0.858-0.877) | 0.171 (0.153-0.191) | 0.165 (0.161-0.170) | 82.571 | Rank 112 |
| DT+SVM+CatBoost | 0.790 (0.780-0.801) | 0.157 (0.133-0.183) | 0.567 (0.546-0.589) | 0.156 (0.132-0.183) | 0.882 (0.873-0.890) | 0.154 (0.140-0.169) | 0.153 (0.148-0.159) | 83.143 | Rank 113 |
| AdaBoost+RF+LightGBM+NaiveBayes+SVM | 0.763 (0.752-0.774) | 0.243 (0.216-0.270) | 0.585 (0.564-0.609) | 0.263 (0.232-0.294) | 0.835 (0.825-0.846) | 0.172 (0.154-0.190) | 0.166 (0.161-0.172) | 83.571 | Rank 114 |
| DT+RF+LightGBM+NaiveBayes | 0.762 (0.752-0.774) | 0.243 (0.215-0.271) | 0.585 (0.563-0.608) | 0.263 (0.231-0.295) | 0.834 (0.825-0.845) | 0.174 (0.155-0.194) | 0.169 (0.164-0.174) | 84.857 | Rank 115 |
| DT+LightGBM+SVM | 0.771 (0.761-0.782) | 0.244 (0.215-0.271) | 0.583 (0.562-0.604) | 0.260 (0.229-0.291) | 0.845 (0.836-0.855) | 0.169 (0.151-0.186) | 0.168 (0.162-0.173) | 85.429 | Rank 116 |
| NaiveBayes+SVM | 0.793 (0.784-0.803) | 0.164 (0.136-0.189) | 0.566 (0.543-0.587) | 0.163 (0.136-0.189) | 0.884 (0.876-0.893) | 0.154 (0.138-0.171) | 0.163 (0.159-0.166) | 85.571 | Rank 117 |
| DT+LightGBM+NaiveBayes | 0.747 (0.736-0.757) | 0.269 (0.240-0.297) | 0.584 (0.561-0.606) | 0.302 (0.269-0.336) | 0.811 (0.800-0.822) | 0.174 (0.154-0.194) | 0.174 (0.169-0.179) | 87.143 | Rank 118 |
| LightGBM+CatBoost | 0.770 (0.759-0.782) | 0.231 (0.202-0.259) | 0.585 (0.563-0.607) | 0.246 (0.214-0.276) | 0.846 (0.836-0.856) | 0.169 (0.151-0.186) | 0.164 (0.158-0.169) | 87.429 | Rank 119 |
| LightGBM+NaiveBayes | 0.742 (0.731-0.753) | 0.284 (0.254-0.311) | 0.581 (0.559-0.603) | 0.323 (0.289-0.356) | 0.802 (0.792-0.813) | 0.170 (0.151-0.188) | 0.178 (0.173-0.183) | 88.286 | Rank 120 |
| DT+LightGBM | 0.723 (0.712-0.734) | 0.290 (0.263-0.318) | 0.585 (0.563-0.607) | 0.342 (0.309-0.374) | 0.778 (0.767-0.789) | 0.168 (0.149-0.186) | 0.186 (0.180-0.191) | 89.429 | Rank 121 |
| LightGBM+NaiveBayes+SVM | 0.749 (0.738-0.759) | 0.263 (0.235-0.288) | 0.585 (0.563-0.607) | 0.293 (0.261-0.323) | 0.815 (0.805-0.825) | 0.169 (0.152-0.186) | 0.173 (0.168-0.178) | 90.429 | Rank 122 |
| SVM | 0.791 (0.781-0.801) | 0.164 (0.140-0.187) | 0.557 (0.537-0.578) | 0.163 (0.139-0.188) | 0.882 (0.874-0.892) | 0.148 (0.133-0.163) | 0.173 (0.169-0.176) | 92.857 | Rank 123 |
| NaiveBayes | 0.685 (0.673-0.696) | 0.297 (0.268-0.324) | 0.560 (0.536-0.581) | 0.370 (0.336-0.404) | 0.731 (0.718-0.742) | 0.161 (0.144-0.179) | 0.210 (0.204-0.215) | 93.000 | Rank 124 |
| KNN | 0.585 (0.573-0.597) | 0.302 (0.276-0.326) | 0.524 (0.501-0.547) | 0.431 (0.397-0.468) | 0.607 (0.595-0.621) | 0.149 (0.133-0.166) | 0.279 (0.272-0.286) | 93.571 | Rank 125 |
| AdaBoost+DT+RF+SVM+CatBoost | 0.763 (0.752-0.773) | 0.229 (0.199-0.256) | 0.577 (0.555-0.600) | 0.246 (0.213-0.278) | 0.837 (0.827-0.848) | 0.167 (0.148-0.185) | 0.167 (0.162-0.173) | 95.857 | Rank 126 |
| DT+RF+CatBoost | 0.757 (0.746-0.767) | 0.244 (0.217-0.272) | 0.559 (0.538-0.582) | 0.267 (0.236-0.298) | 0.827 (0.817-0.837) | 0.159 (0.143-0.176) | 0.174 (0.168-0.179) | 97.286 | Rank 127 |
| DT+NaiveBayes+CatBoost | 0.761 (0.750-0.771) | 0.228 (0.200-0.258) | 0.571 (0.550-0.593) | 0.246 (0.216-0.278) | 0.835 (0.826-0.845) | 0.165 (0.148-0.183) | 0.169 (0.164-0.174) | 99.143 | Rank 128 |
| XGBoost | 0.709 (0.697-0.720) | 0.259 (0.232-0.283) | 0.539 (0.516-0.561) | 0.307 (0.275-0.337) | 0.767 (0.756-0.778) | 0.153 (0.137-0.170) | 0.237 (0.228-0.246) | 103.286 | Rank 129 |

**Supplementary Table 32.** Baseline characteristics of included and excluded participants across four cohorts

| Variable | HRS Excluded | HRS Included | ELSA Excluded | ELSA Included | SHARE Excluded | SHARE Included | CHARLS Excluded | CHARLS Included |
| --- | --- | --- | --- | --- | --- | --- | --- | --- |
| Sample size, n | 15869 | 2600 | 6389 | 3043 | 28883 | 1533 | 14158 | 3550 |
| Age, mean (SD) | 67.06 (11.41) | 73.64 (6.61) | 63.73 (11.16) | 69.81 (7.30) | 63.06 (10.19) | 78.02 (6.89) | 56.75 (9.85) | 67.28 (6.38) |
| CES-D score, mean (SD) | 1.62 (2.06) | 1.04 (1.61) | 1.76 (2.06) | 1.20 (1.67) | 2.35 (2.28) | 2.45 (2.17) | 8.56 (6.44) | 7.84 (5.87) |
| Body mass index (BMI), mean (SD) | 28.04 (6.01) | 26.64 (4.60) | 28.45 (5.21) | 27.16 (4.27) | 26.40 (4.33) | 25.76 (3.98) | 24.34 (37.18) | 23.64 (31.48) |
| Wealth, mean (SD) | 481840.50 (1282130.47) | 631722.60 (1123474.95) | 250320.47 (402746.49) | 291229.54 (367475.57) | / | / | 24687.45 (53249.04) | 20853.46 (30188.75) |
| Sex (male), n (%) | 6442 (40.6) | 1144 (44.0) | 2755 (43.1) | 1371 (45.1) | 12847 (44.5) | 668 (43.6) | 6593 (46.6) | 1886 (53.1) |
| Education level, n (%) |  |  |  |  |  |  |  |  |
| 1 | 3970 (25.1) | 529 (20.3) | 2750 (46.9) | 1312 (47.5) | 14606 (50.6) | 1046 (68.2) | / | / |
| 2 | 8518 (53.8) | 1501 (57.7) | 1119 (19.1) | 510 (18.5) | 8749 (30.3) | 308 (20.1) | / | / |
| 3 | 3352 (21.2) | 570 (21.9) | 1195 (20.4) | 571 (20.7) | 5528 (19.1) | 179 (11.7) | / | / |
| 4 | / | / | 804 (13.7) | 367 (13.3) | / | / | / | / |
| Smoking status = yes, n (%) | 9035 (57.4) | 1427 (55.4) | 4082 (63.9) | 1869 (61.4) | 13633 (47.5) | 625 (40.8) | 5360 (38.3) | 1571 (44.3) |
| Drinking status = yes, n (%) | 7759 (48.9) | 1409 (54.2) | 4718 (88.4) | 2543 (90.7) | / | / | 5357 (38.3) | 1466 (41.3) |
| Hypertension = yes, n (%) | 8712 (54.9) | 1403 (54.0) | 2645 (41.4) | 1204 (39.6) | 8927 (31.1) | 635 (41.4) | 3529 (25.3) | 1006 (28.4) |
| Cancer = yes, n (%) | 2112 (13.3) | 450 (17.3) | 419 (6.6) | 256 (8.4) | 1478 (5.1) | 95 (6.2) | 144 (1.0) | 23 (0.6) |
| Race/ Native, n (%) |  |  |  |  |  |  |  |  |
| 1 | 11543 (72.7) | 2206 (84.8) | 6202 (97.1) | 3006 (98.8) | 28156 (97.7) | 1512 (98.6) | 9824 (91.9) | 2694 (93.6) |
| 2 | 2352 (14.8) | 216 (8.3) | / | / | / | / | / | / |
| 3 | 1568 (9.9) | 142 (5.5) | / | / | / | / | / | / |
| 4 | 404 (2.5) | 36 (1.4) | / | / | / | / | / | / |
| Cardiovascular disease = yes, n (%) | 4549 (28.7) | 688 (26.5) | 342 (5.4) | 102 (3.4) | 4038 (14.1) | 367 (23.9) | 1982 (14.2) | 520 (14.7) |
| Physical activity = yes, n (%) | 10803 (68.1) | 2116 (81.4) | 4505 (72.0) | 2553 (83.9) | 24427 (85.0) | 1248 (81.4) | 1884 (34.8) | 477 (31.8) |
| Hip fracture = yes, n (%) | 427 (4.9) | 56 (2.2) | 103 (3.3) | 22 (0.7) | / | / | 213 (1.7) | 49 (1.4) |

| **Supplementary Table 33.** Associations of single-domain muscle strength and walking speed trajectories with incident diabetes across the HRS, ELSA, SHARE, and CHARLS cohorts. | | | | | | | | |
| --- | --- | --- | --- | --- | --- | --- | --- | --- |
| Exposure Variable | Model 1 | | Model 2 | | Model 3 | | Model 4 | |
|  | HR (95% CI) | P-value | HR (95% CI) | P-value | HR (95% CI) | P-value | HR (95% CI) | P-value |
| HRS |  |  |  |  |  |  |  |  |
| Muscle strength trajectory | | | |  |  |  |  |  |
| High−increasing muscle strength | 1.00 (Reference) | - | 1.00 (Reference) | - | 1.00 (Reference) | - | 1.00 (Reference) | - |
| Low−declining muscle strength | 0.90 (0.63 ~ 1.29) | 0.573 | 0.99 (0.69 ~ 1.43) | 0.952 | 0.92 (0.65 ~ 1.32) | 0.663 | 1.00 (0.69 ~ 1.45) | 0.979 |
| Walking speed trajectory | |  |  |  |  |  |  |  |
| Low−increasing walking speed | 1.00 (Reference) | - | 1.00 (Reference) | - | 1.00 (Reference) | - | 1.00 (Reference) | - |
| High−increasing walking speed | 1.05 (0.68 ~ 1.61) | 0.838 | 1.10 (0.70 ~ 1.72) | 0.687 | 1.04 (0.67 ~ 1.61) | 0.869 | 1.09 (0.68 ~ 1.73) | 0.726 |
| ELSA |  |  |  |  |  |  |  |  |
| Muscle strength trajectory | | | |  |  |  |  |  |
| Low level decline muscle strength | 1.00 (Reference) | - | 1.00 (Reference) | - | 1.00 (Reference) | - | 1.00 (Reference) | - |
| High level decline muscle strength | 1.07 (0.84 ~ 1.37) | 0.573 | 1.35 (0.92 ~ 1.98) | 0.131 | 1.19 (0.91 ~ 1.54) | 0.203 | 1.38 (0.92 ~ 2.09) | 0.120 |
| Walking speed trajectory | |  |  |  |  |  |  |  |
| Low level stable walking speed | 1.00 (Reference) | - | 1.00 (Reference) | - | 1.00 (Reference) | - | 1.00 (Reference) | - |
| High level improving walking speed | 0.62 (0.43 ~ 0.90) | 0.012* | 1.13 (0.75 ~ 1.70) | 0.570 | 0.64 (0.44 ~ 0.93) | 0.019* | 1.11 (0.73 ~ 1.68) | 0.625 |
| SHARE |  |  |  |  |  |  |  |  |
| Muscle strength trajectory | | |  |  |  |  |  |  |
| Low level decline muscle strength | 1.00 (Reference) | - | 1.00 (Reference) | - | 1.00 (Reference) | - | 1.00 (Reference) | - |
| High level decline muscle strength | 0.91 (0.67 ~ 1.22) | 0.521 | 1.23 (0.82 ~ 1.84) | 0.324 | 1.11 (0.78 ~ 1.57) | 0.555 | 1.24 (0.82 ~ 1.88) | 0.313 |
| Walking speed trajectory | |  |  |  |  |  |  |  |
| Low stable walking speed | 1.00 (Reference) | - | 1.00 (Reference) | - | 1.00 (Reference) | - | 1.00 (Reference) | - |
| High increasing walking speed | 0.68 (0.49 ~ 0.96) | 0.026* | 0.74 (0.52 ~ 1.05) | 0.095 | 0.71 (0.51 ~ 1.00) | 0.052 | 0.74 (0.52 ~ 1.05) | 0.093 |
| CHARLS |  |  |  |  |  |  |  |  |
| Muscle strength trajectory | | | |  |  |  |  |  |
| Moderate level stable muscle strength | 1.00 (Reference) | - | 1.00 (Reference) | - | 1.00 (Reference) | - | 1.00 (Reference) | - |
| High level rapid decline muscle strength | 1.00 (0.74 ~ 1.35) | 0.987 | 0.96 (0.71 ~ 1.30) | 0.801 | 0.98 (0.73 ~ 1.33) | 0.921 | 0.95 (0.70 ~ 1.29) | 0.743 |
| Walking speed trajectory | |  |  |  |  |  |  |  |
| High level decline walking speed | 1.00 (Reference) | - | 1.00 (Reference) | - | 1.00 (Reference) | - | 1.00 (Reference) | - |
| Low level decline walking speed | 0.90 (0.71 ~ 1.13) | 0.353 | 0.92 (0.72 ~ 1.17) | 0.486 | 0.89 (0.71 ~ 1.13) | 0.358 | 0.91 (0.71 ~ 1.17) | 0.466 |
| Model 1: Crude | | | | | | | | |
| Model 2: Adjust: Age, Sex, Race/ Native, Education, Wealth (Without SHARE), BMI, Hypertension, Drinking status (Without SHARE), Smoking status, Cancer, Cardiovascular disease, Hip fracture, Physical activity, CESD, Cognitive score (Without ELSA) | | | | | | | | |
| Model 3: IPTW-Crude | | | | | | | | |
| Model 4: IPTW- Adjust: Age, Sex, Race/ Native, Education, Wealth (Without SHARE), BMI, Hypertension, Drinking status (Without SHARE), Smoking status, Cancer, Cardiovascular disease, Hip fracture, Physical activity, CESD, Cognitive score (Without ELSA) | | | | | | | | |
| Significance: * p < 0.05, ** p < 0.01, *** p < 0.001 | | | | | | | | |
